# An updated checklist of the ants of Thailand (Hymenoptera, Formicidae)

**DOI:** 10.3897/zookeys.998.54902

**Published:** 2020-11-26

**Authors:** Salinee Khachonpisitsak, Seiki Yamane, Patchara Sriwichai, Weeyawat Jaitrong

**Affiliations:** 1 Department of Biology, Faculty of Science, Burapha University, 169 Long Hard Bangsaen Road, Sanesuk, Mueang, Chon Buri, 20131 Thailand Burapha University Chon Buri Thailand; 2 Kagoshima University Museum, Korimoto 1-21-30, Kagoshimashi, 890-0065 Japan Kagoshima University Kagoshima Japan; 3 Department of Medical Entomology, Faculty of Tropical Medicine, Mahidol University, 420/6 Ratchawithi Road, Ratchathewi, Bangkok, 10400 Thailand Mahidol University Bangkok Thailand; 4 Thailand Natural History Museum, National Science Museum, Technopolis, Khlong 5, Khlong Luang, Pathum Thani, 12120 Thailand Thailand Natural History Museum Pathum Thani Thailand

**Keywords:** Distribution, new records, updated nomenclature, taxonomy

## Abstract

Thailand has a great diversity of ant fauna as a zoogeographical crossroads and biodiversity hotspot. The last publication presenting a Thai ant checklist was published in 2005. In the present paper, based on an examination of museum specimens and published records, a comprehensive and critical species list of Thai ants is synthesized. Currently, 529 valid species and subspecies in 109 genera among ten subfamilies are known from Thailand with their diversity and distribution within 77 provinces presented and assigned to six geographical regions. Furthermore, Thailand is the type locality for 81 ant species. Forty-one species are here newly recorded for Thailand with photographs illustrating these species. The checklist provides information on distribution and a comprehensive bibliography. This study will also serve as a guide for the upper northeast and central Thailand, which are poorly sampled; a comprehensive reference list relating to endemic taxa and localities where conservation is an important priority, thus an essential resource for policy makers and conservation planners concerned with the management of insect diversity in Thailand; and a list of exotic ant species found in Thailand, which could possibly impact the ecological balance.

## Introduction

Thailand, with an area of 513,120 km^2^, is located in the central portion of Southeast Asia and extend from 5°45'N to 20°30'N and from 97°30'E to 105°45'E. One thousand and five hundreds kilometers separate the northern from the southern parts of the country, while the maximum width is ca. 800 km. Thailand is bordered to the north by Myanmar and Laos, to the east by Laos and Cambodia, to the west by Myanmar and the Andaman Sea, and to the south by Malaysia and the Gulf of Thailand. Thailand has been divided into six geographical regions: northern, northeastern, central, eastern, western, and southern; with each region being unique in terms of geography, human population size, and available resources (Royal Forest Department 1985). Thailand has been described as a natural gateway or zoogeographical crossroads to Indochina in the north and the Sundaic region in the south (Luangjame et al. 1997) and is considered as a biodiversity hotspot due to its rich diversity, high endemism, and great habitat loss (Myers et al. 2000; Plant 2014; The Sustainability Consortium WRI 2019). Ant diversity in various countries of Southeast Asia, including countries surrounding Thailand, has been summarized in several publications in the past two decades (e.g., China: Guénard and Dunn 2012; Yunnan: Liu et al. 2015; Vietnam: Eguchi et al. 2011; Loas: Jaitrong et al. 2016; Cambodia: Hosoishi et al. 2013; Borneo: Pfeiffer et al. 2011), as well as in online tools and databases (antmaps.org, Janicki et al. 2016, Guénard et al. 2017). If the diversity of ants has also been studied in Thailand (Jaitrong and Nabhitabhata 2005), the data remain scattered (but see antmap.org).

The first ant records in Thailand, *Camponotus exiguoguttatus* (Smith, 1857) and *Camponotus irritans* Forel, 1886 were presented in Forel (1892). Since that time, several other foreign researchers have contributed to taxonomic and faunistic studies (e.g., Bingham 1903, 1906, Wilson 1964, Baroni Urbani 1977a, Bolton 1977, 2000, 2007, Rigato 1994, Dorrow 1995, Ward 2001, Lattke 2004, Wang 2003, Baroni Urbani and Andrade 2003). More recently, Thai and other myrmecologists have joined taxonomic activities, publishing many important articles, in addition to those written by foreigners (Jaitrong and Nabhitabhata 2005, Dumpert 2006, Dumpert et al. 2006, Kohout 2006, LaPolla 2009, Jaitrong 2010, 2015, Jaitrong and Yamane 2011, 2012, 2013, 2018, Jaitrong and Hashimoto 2012, Jaitrong et al. 2011b, 2012, 2016, Jaitrong and Ruangsittichai 2018, Jaitrong et al. 2013, 2018a, 2018b, 2019a, 2019b, 2020, Jaitrong and Wiwatwitaya 2015, Hosoishi 2015, Hosoishi et al. 2015, Phengsi et al. 2018, Tanansathaporn et al. 2018, Wang et al. 2018, Jaitrong and Asanok 2018, Zettel et al, 2018, Yamane and Jaitrong 2019, Okido et al, 2020). These studies have added many new species and records, but the information is scattered across multiple many taxonomic publications, making it difficult to obtain a full overview of the diversity of Thai ants.

The present species checklist of Thai ants presents a synthesis of the currently recognized 529 species, including 41 new records, a compilation of recent distribution information, and a comprehensive bibliography, allowing the ant fauna of Thailand to be examined in one publication and in a broader regional context.

## Materials and methods

**Figure 1.**
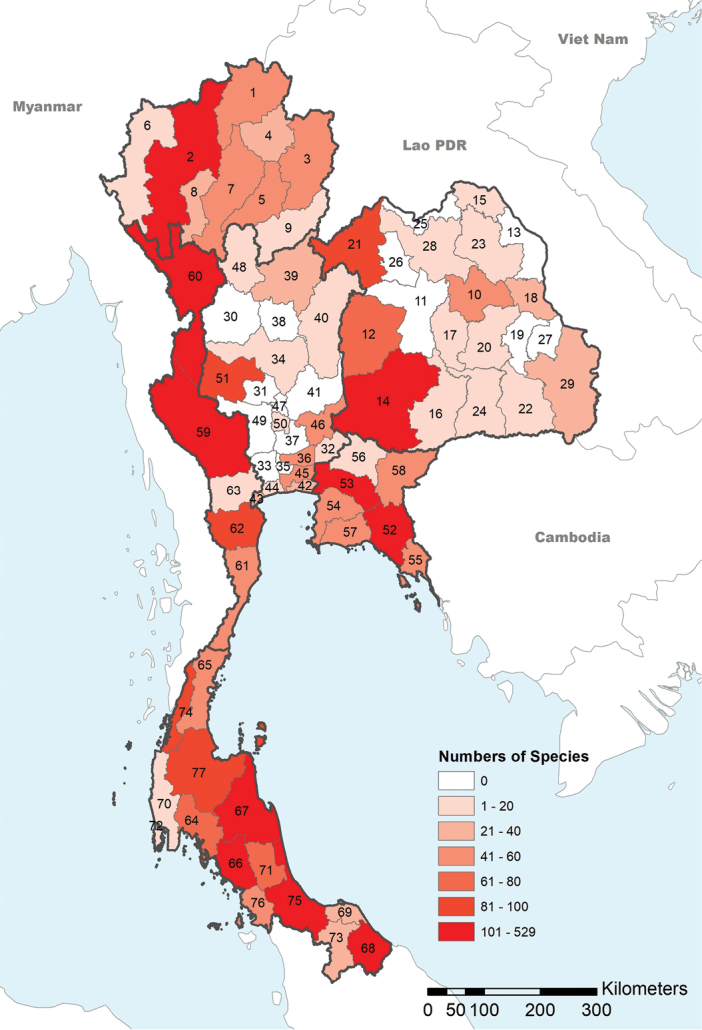
Map of the known species diversity in 77 Thai provinces. Shades indicate the number of species from low (light) to high (darker colors). Dark and light grey bold line indicate borders of geopolitical regions and provinces, respectively. **Northern**: 1 Chiang Rai; 2 Chiang Mai; 3 Nan; 4 Phayao; 5 Phrae; 6 Mae Hong Son; 7 Lampang; 8 Lamphun; 9 Uttaradit. **Northeastern**: 10 Kalasin; 11 Khon Kaen; 12 Chaiyaphum; 13 Nakhon Phanom; 14 Nakhon Ratchasima; 15 Bueng Kan; 16 Buri Ram; 17 Maha Sarakham; 18 Mukdahan; 19 Yasothon; 20 Roi Et; 21 Loei; 22 Si Sa Ket; 23 Sakon Nakhon; 24 Surin; 25 Nong Khai; 26 Nong Bua Lam Phu; 27 Amnat Charoen; 28 Udon Thani; 29 Ubon Ratchathani. **Central**: 30 Kamphaeng Phet; 31 Chai Nat; 32 Nakhon Nayok; 33 Nakhon Pathom; 34 Nakhon Sawan; 35 Nonthaburi; 36 Pathum Thani; 37 Phra Nakhon Si Ayutthaya; 38 Phichit; 39 Phitsanulok; 40 Phetchabun; 41 Lop Buri; 42 Samut Prakan; 43 Samut Songkhram; 44 Samut Sakhon; 45 Bangkok; 46 Saraburi; 47 Sing Buri; 48 Sukhothai; 49 Suphan Buri; 50 Ang Thong; 51 Uthai Thani. **Eastern**: 52 Chanthaburi; 53 Chachoengsao; 54 Chon Buri; 55 Trat; 56 Prachin Buri; 57 Rayong; 58 Sa Kaeo. **Western**: 59 Kanchanaburi; 60 Tak; 61 Prachuap Khiri Khan; 62 Phetchaburi; 63 Ratchaburi. **Southern**: 64 Krabi; 65 Chunphon; 66 Trang; 67 Nakhon Si Thammarat; 68 Narathiwat; 69 Pattani; 70 Phang–nga; 71 Phatthalung; 72 Phuket; 73 Yala; 74 Ranong; 75 Songkhla; 76 Satun; 77 Surat Thani. The black borders represent the province available the endemic species restricted in Thailand.

An updated checklist was compiled on the data mined from museum records, literature, and collections made during field trips from 1997 to 2020 and covering six geographical regions and 77 provinces of Thailand (northern region: 9 provinces, northeastern region: 20 provinces, central region: 22 provinces, eastern region: 7 provinces, western region: 5 provinces, and southern region: 14 provinces; Fig. 1). This list presents all known Thai ants described and recorded from 1892 to 2020 covering current valid names, distributions, and references.

Extensive collections made from various localities in Thailand during the last 20 years and now deposited in the Natural History Museum of National Science Museum, Thailand (**THNHM**), Ant Museum of Kasetsart University, Thailand (**AMK**) and Seiki Yamane’s Collection at Kitakyushu Museum of Natural History and Human History, Japan (**SKYC**) have been examined. Most specimens were collected by W. Jaitrong and Sk. Yamane. Specimens deposited in several University collections in Thailand (e.g., Faculty of Agriculture of Chiang Mai University, Faculty of Science of Burapa University, Faculty of Agriculture of Kasetsart University) were also examined. Specimens were identified mainly by W. Jaitrong and Sk. Yamane using taxonomic keys for Southeast Asian ants (e.g., Ward 2001, Bolton 2007, Eguchi 2008, Hosoishi and Ogata 2009, Jaitrong and Yamane 2011, Jaitrong et al. 2011a, b, Schmidt and Shattuck 2014, Jaitrong and Wiwatwitaya 2015, Hosoishi and Ogata 2016,Jaitrong et al. 2016, 2018a, b, 2019a, b, Phengsi et al. 2018, Jaitrong and Yamane 2018, Tanansathaporn et al. 2018, Wang et al. 2018, Jaitrong and Asanok 2018, Jaitrong et al. 2019a, b, Yamane and Jaitrong 2019, Jaitrong et al. 2020, Okido et al. 2020).

The data compilation also includes records from the GABI database (Guénard et al. 2017), which has accumulated information from more than 10,000 publications and other databases (e.g., Antweb 2019), with species distributions available for visualization through the website antmaps.org (Janicki et al. 2016). Separately, information provided in other websites (e.g., Antwiki: www.antwiki.org, Antweb: www.antweb.org) are also added here.

Presumed biological species (most might be undescribed are not included in the list. Species validity, spelling, and authorship follow Bolton’s Synopsis of the Formicidae and Catalogue of Ants of the World (Bolton 2019). The present list is arranged alphabetically by subfamily, genus, and species. Binominal species names are followed by author name(s) and publication year. Brief synonymic list including the history of taxonomic treatments and distribution are also given. Material examined and images are provided for the species newly recorded for the country.

All known localities for each species are shown according to six geopolitical regions; starting from the north, to northeast, west, center, east, and lastly to the south. Geopolitical regions are denoted in bold type followed by provinces (**Prov**) and names of national parks (**NP**), wildlife sanctuaries (**WS**), botanical gardens (**BG**), districts (**Dist**) or subdistricts (**Subdist**) in parentheses. Type locality of species described from Thailand is marked with an asterisk (*).

## Results and discussion

**Figures 2–11.**
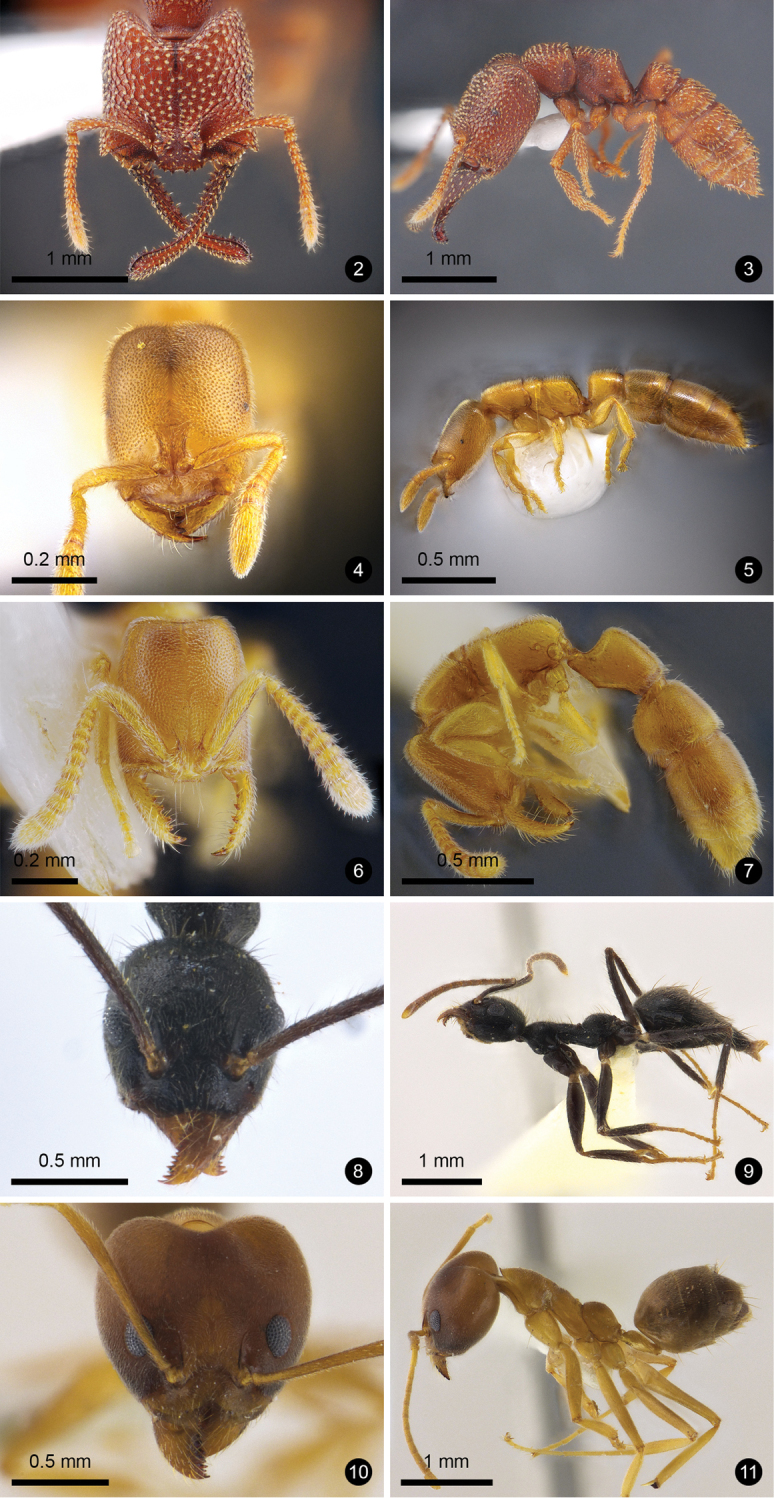
Ant species new to Thailand. **2, 3***Mystrium camillae***4, 5***Prionopelta kraepelin***6, 7***Xymmer phungi***8, 9***Technomyrmex grandis***10, 11***Technomyrmex lisae*.

**Figures 12–21.**
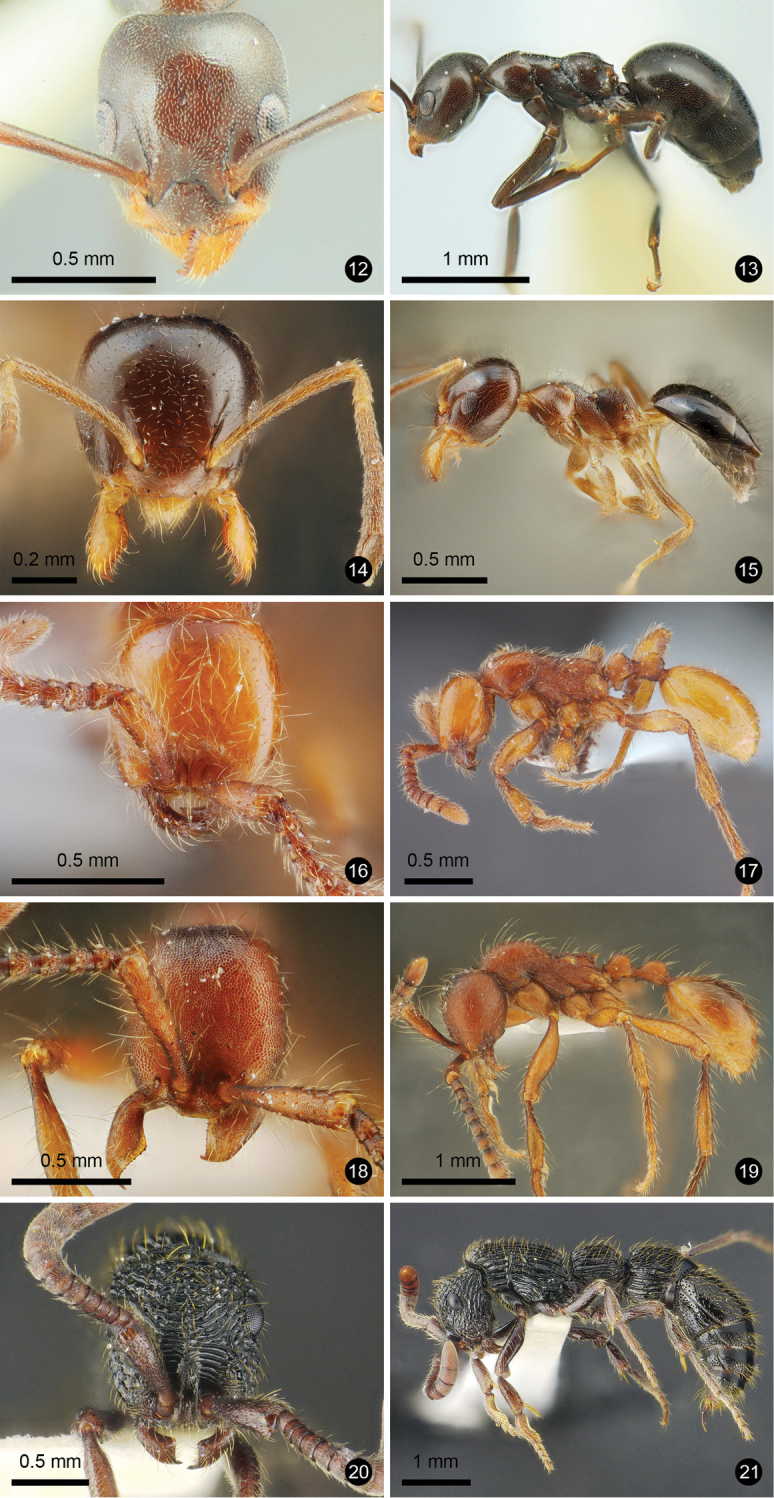
Ant species new to Thailand. **12, 13***Technomyrmex reductus***14, 15***Technomyrmex strenuus***16, 17***Aenictus brevipodus***18, 19***Aenictus yamanei***20, 21***Chrysapace costatus*.

**Figures 22–31.**
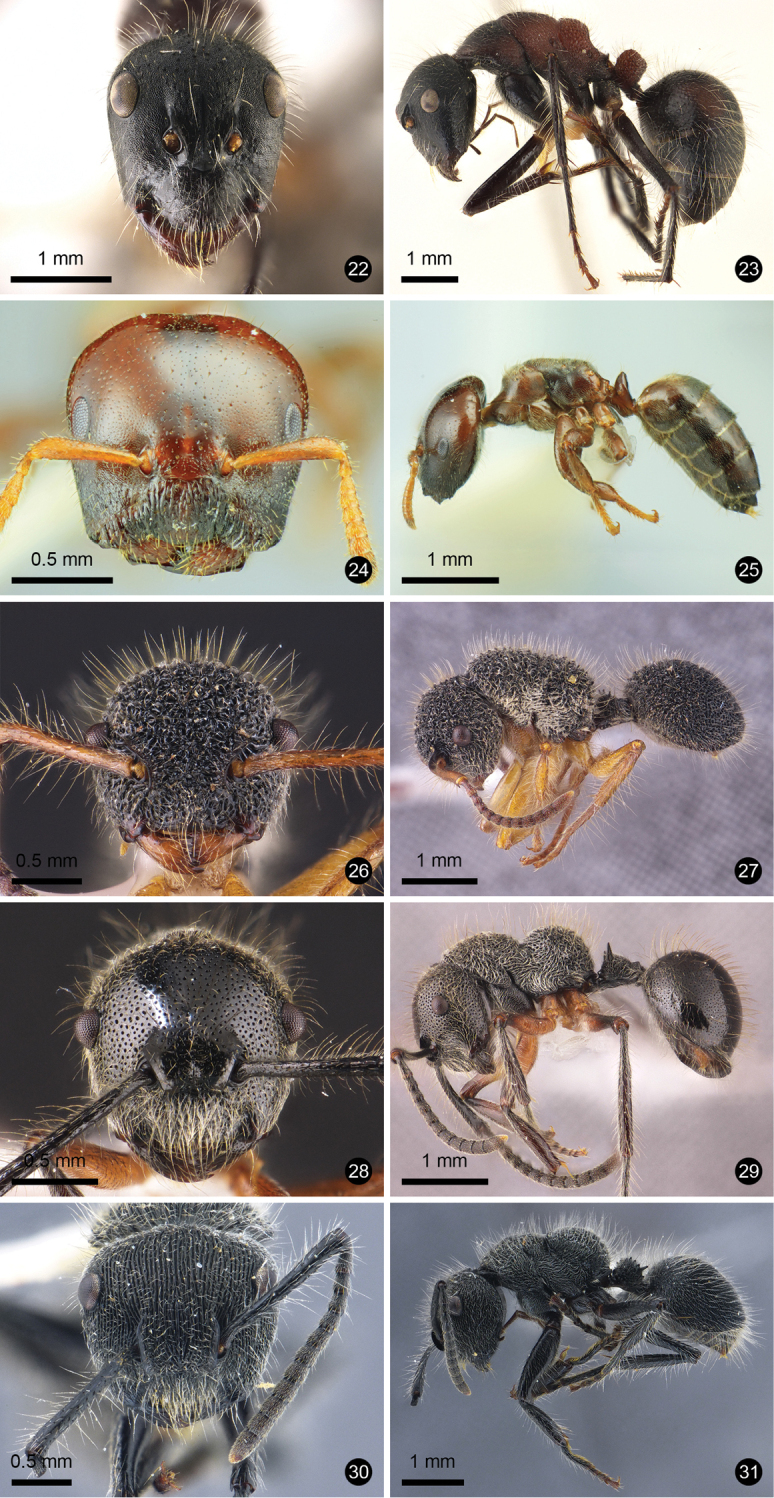
Ant species new to Thailand. **22, 23***Camponotus mutilarius***24, 25***Cladomyrma petalae***26, 27***Echinopla cherapunjiensis***28, 29***Echinopla fisheri***30, 31***Echinopla lineata*.

This updated checklist contains 529 ant species and subspecies from Thailand belonging to 109 genera and ten subfamilies; among them, 283 species were cited previously in Jaitrong and Nabhitabhata (2005). This checklist includes 41 species newly recorded in Thailand (Figs 2–81). Fifteen names (*Aenictus ceylonicus*, *Aenictus fergusoni*, *Aenictus javanus*, *Calyptomyrmex emeryi*, *Cardiocondyla nuda*, *Diacamma rugosum*, *Diacamma sculpturata*, *Diacamma vargens*, *Pheidole nodifera, Pheidole bugi*, *Pheidole incense*, *Pheidole tsailuni*, *Polyrhachis bellicosa*, *Polyrhachis sumatraensis*, and *Pristomyrmex pungens*) have been removed from Jaitrong and Nabhitabhata (2005) as they were either previously misidentified or have been synonymized with other species (Table 1). For instance, *Aenictus ceylonicus* was previously reported to be widespread in Southeast Asia including Thailand (Willson 1964), but most recent work now considers it to be restricted to India and Sri Lanka (Jaitrong and Yamane 2013). *Calyptomyrmex emeryi* was synonymized with *Calyptomyrmex beccarii* and *Pristomyrmex pungens* with *Pristomyrmex punctatus* (see remarks under each species below as well as Table 1). Images of a *Diacamma sculpturata* specimen available in Antweb (CASENT0173639) is considered to be a misidentification of *D. orbiculatum*, here corrected in the present study.

**Table 1.** List of species excluded from the previous ant check list by Jaitrong and Nabhitabhata (2005).

Excluded species	Remarks
*Aenictus ceylonicus* Mayr, 1866	Restricted to Sri Lanka and southern India (Jaitrong and Yamane 2013). Several species of the species group found in Thailand were previously identified as this species.
*Aenictus fergusoni* Forel, 1901	All Thai specimens cited in Jaitrong and Nabhitabhata (2005) were reidentified as *Aenictus hodgsoni* Forel, 1901 by Jaitrong and Yamane (2011).
*Aenictus javanus* Emery, 1896	Distributed in Java and Borneo (Jaitrong and Yamane 2012). All Thai specimens cited in Jaitrong and Nabhitabhata (2005) are reidentified as *Aenictus longinodus* Jaitrong & Yamane, 2012.
*Calyptomyrmex emeryi* Forel, 1901	A junior synonym of *Calyptomyrmex beccarii* (Forel, 1901).
*Cardiocondyla nuda* Mayr, 1866	Reidentified as *Cardiocondyla itsukii* Seifert, Okita & Heinze, 2017 and *Cardiocondyla kagutsuchi* Terayama, 1999 in this paper.
*Diacamma rugosum* Le Guillou, 1842	All specimens in Jaitrong and Nabhitabhata (2005) are reidentified as *D. orbiculatum* Santschi, 1932 in the present paper.
*Diacamma sculpturata* Smith, 1859	All specimens in Jaitrong and Nabhitabhata (2005) are reidentified as *D. violaceum* Forel, 1900 in the present paper. The species *D. sculpturata* is a junior synonym of *D. rugosum*.
*Diacamma vagans* Smith, 1860	Restricted to the Moluccas, Bacan Island. Other records from literature probably refer to different species (Laciny et al. 2015).
*Pheidole bugi* Wheeler, 1919	A junior synonym of *Pheidole parva* Wheeler, 1919).
*Pheidole incensa* Wheeler, 1928	A junior synonym of *Pheidole pieli* Santschi, 1925.
*Pheidole nodifera* Smith, 1858	Reidentified as *Pheidole tumida* Eguchi, 2008.
*Pheidole tsailuni* Wheeler, 1929	A junior synonym of *Peidole rabo* Forel, 1913.
*Polyrhachis bellicosa* Smith, 1859	All Thai specimens in Jaitrong and Nabhitabhata (2005) should be reidentified as *P. olybria* Forel, 1912 (sensu Kohout, 2013).
*Polyrhachis sumatrensis hamulata* Emery, 1887	A junior synonym of *Polyrhachis sculpturata* Smith, 1860.
*Pristomyrmex pungens* Mayr, 1866	A junior synonym of *Pristomyrmex punctatus* (Smith, 1860).

The most speciose subfamily, Myrmicinae comprises 36.70% of all genera and 40.83% of all species, followed by Formicinae (17.43% and 25.14%), Ponerinae (17.43% and 10.59%), Dorylinae (11.01% and 9.83%), and Dolichoderinae (6.42% and 6.62%), respectively. The rest, the smaller subfamilies collectively constitute 11.01% and 6.99% of all genera and species known from Thailand respectively (4.59% and 1.51% for Amblyoponinae, 2.75% and 0.95% for Proceratiinae, 1.83% and 0.19% for Leptanillinae, 0.92% and 1.32% for Ectatomminae, 0.92% and 3.02% for Pseudomyrmecinae, Table 2). Worldwide, 17 subfamilies, 337 genera, and 15,621 extant species and subspecies are recognized in Formicidae, excluding fossil taxa (Antwiki 2019). Thus, to date the number of species and subspecies known from Thailand accounts for 3.39% of the global total and 32.34% of the Southeast Asian species. Known ant fauna in Thailand seem relatively poor when compared to those of China, India, Indonesia, and Malaysia (ca. 1,500 species for all these countries). This may be because intensive surveys have started only recently, some of those countries are larger, or are in closer proximity to the equator, where biodiversity is greater. In nearby countries such as Laos, Cambodia, Myanmar, and Vietnam, only approximately to 300 species have been reported (Antweb 2019). While knowledge on ant diversity in Thailand is still incomplete, knowledge in nearby regions is probably even more limited with less than 130 species recorded in Cambodia and Laos, ca. 350 species in Myanmar, and ca. 440 species recoded in Vietnam (antmaps.org, Janicki et al. 2016).

**Table 2.** Numbers and percentages of known genera and species recorded for each subfamily in Thailand.

Subfamilies	Genus richness	% Genus	Species richness	% Species
Myrmicinae	40	36.70	216	40.83
Formicinae	19	17.43	133	25.14
Ponerinae	19	17.43	56	10.59
Dorylinae	12	11.01	52	9.83
Dolichoderinae	7	6.42	35	6.62
Amblyoponinae	5	4.59	8	1.51
Proceratiinae	3	2.75	5	0.95
Leptanillinae	2	1.83	1	0.19
Ectatomminae	1	0.92	7	1.32
Pseudomyrmecinae	1	0.92	16	3.02
**Total**	**109**	**100**	**529**	**100**

The most speciose ant genera in Thailand are *Polyrhachis* (62 species, 11.72%), *Pheidole* (51 species, 9.64%), *Aenictus* (39 species, 7.37%), *Strumigenys* (32 species, 6.05%), *Crematogaster* (29 species, 5.48%), *Camponotus* (26 species, 4.91%) and *Tetramorium* (26 species, 4.91%, Suppl. material 1: Table S1). The majority of genera (36) are species-poor (2–7 species) or known in Thailand from a single species (47 genera, Suppl. material 1: Table S1). It should be noted, however, that the examination of the collection of specimens deposited in THNHM and SKYC present several unidentified species which, following their identification or description, should increase the known richness of some genera in Thailand, such as *Camponotus*, *Carebara*, *Crematogaster*, *Leptogenys*, *Monomorium, Pheidole*, *Polyrhachis*, *Stigmatomma*, and *Tetramorium*. In other genera, there are specimens that have only been identified at generic level and thus the number of species is likely to greatly increase once these are identified. For thirteen genera, the specimens collected have not been identified at the species level and are thus only known at the generic level (*Aphaenogaster*, *Chronoxenus*, *Discothyrea*, *Gesomyrmex*, *Hypoponera*, *Lordomyrma*, *Nylanderia*, *Ooceraea*, *Plagiolepis*, *Ponera*, *Protanilla*, *Temnothorax*, and *Vombisidris*, Suppl. material 1: Table S1). In the collections of THNHM and SKYC, we recognized more than 200 unidentified species from Thailand, many of which seem to be new to science; with several species of *Cerapachys*, *Ponera*, and *Ooceraea* currently under investigation.

Species richness varies substantially among the geopolitical regions of Thailand (Fig. 1). The southern region has the most diverse ant fauna with 306 species and 72 genera recorded, followed by the western and northern regions with 250 species in 68 genera and 241 species in 69 genera respectively while the central and northeastern parts appear to be less studied with 144 species in 58 genera and 211 species in 67 genera, respectively (Suppl. material 1: Table S2). In the northeast part, the faunal studies appear to have been well-done only in few provinces (Nakhon Ratchasrima, Chaiyaphum, and Loei) while the other provinces are possibly poorly explored. The detailed data for the regional gap of ant collection in Thailand are summarized in Suppl. material 1: Table S3.

The current diversity pattern observed between the different Thai provinces shows important differences. Ten provinces have over a hundred species recorded (Fig. 1), with the highest diversity thus far recorded within the Chiang Mai (225 species), Tak (195 species), Nakhon Ratchasima (179 species), Chanthaburi (165 species), and Chachoengsao (153 species) provinces. In contrast, 46 provinces have less than 40 species recorded, including 17 provinces with fewer than ten recorded species, seven provinces with only a single species recorded and 13 provinces without any records. High priority areas with more than 100 species (see red-shaded areas in Fig. 1) are distributed in almost all regions except the central part of Thailand.

Thailand is the type locality for 81 species, including 20 species currently considered as endemic; however, as most species have been recently described, it cannot be dismissed that future sampling or material revision from surrounding countries may extend their known distribution in the future. For ten species, the endemic status to Thailand may remain, considering the peculiar habitats or localities where they have been collected. (Suppl. material 1: Table S4 and Fig. 82). For instance, *Aenictus siamensis* is only known at this time from an isolated population in the high mountains in the central part of Thailand. *Camponotus aureus* is found in the interior but confined to the deep forest. Independent of their future classification, the current endemic status of these species should encourage political actions to preserve their habitats in order to maintain viable populations, which are now under increasing pressure as a result of the changes affecting potential suitable habitats.

On the other hand, 14 species are here considered as exotic species to Thailand, and have been collected from 1997 to 2015 (Deyrup et al. 2000, Wetterer and Vargo 2003, Heterick and Shattuck 2011, Vonshak and Ionescu-Hirsch 2009, Wetterer 2009, 2010a, 2010b, 2010c, Yamane and Jaitrong 2011, Wetterer 2013, Wetterer et al. 2015) (Table 3). To this point, *Ochetellus glaber*, *Lioponera longitarsus*, *Pheidole megacephala*, and *Vollenhovia emeryi* appear to have a more limited regional distribution in Thailand while other species are widely distributed throughout the country. Many of these species have putative native range in the Indo–Pacific, Middle East, or African regions. The exotic species can influence to the native ecological system. For instance, *Technomyrmex albipes* can adapt to a variety of habitats from urban to forests which harbor the native species, which can enable this species to possibly outcompete other species. *Solenopsis geminata* is an exotic venomous ant species in Thailand, causing serious reactions in hypersensitive people by anaphylaxis that accounts for more than 30% of the Thai ant allergic patients (Potiwat et al. 2018). Cases of stings by this ant have been frequently reported on many Asian islands, including those in Indonesia and Taiwan (Hoffman 2010). Although no detailed studies are available in Thailand, this species may compete with native open land ant species for nesting sites and food.

**Table 3.** Exotic ant list and distribution in Thailand. Abbreviations: +, presence; N, North; W, West; NE, North East; C, Central; E, East; S, South.

Species	Origin	Distribution						References
		N	W	NE	C	E	S	
Dolichoderinae								
*Iridomyrmex anceps*	Indo–Pacific	+	+	+	+	+	+	Heterick and Shattuck 2011
*Ochetellus glaber*	Indo–Pacific	+			+			Deyrup et al. 2000
*Tapinoma melanocephalum*	Indo–Pacific	+	+	+	+	+	+	Wetterer 2009
*Technomyrmex albipes*	Indo–Pacific	+	+	+	+	+	+	Wetterer 2009
*Technomyrmex difficilis*	Unknown	+	+	+		+	+	Wetterer 2013
Formicinae								
*Anoplolepis gracilipes*	Asia or Africa	+	+	+	+	+	+	Wetterer 2005
Myrmicinae								
*Monomorium floricola*	Asia	+	+	+	+	+	+	Wetterer 2010a
*Monomorium pharaonis*	Asia or Africa	+	+	+	+	+	+	Wetterer 2010b
*Pheidole megacephala*	Africa				+		+	Wetterer and Vargo 2003
*Solenopsis geminata*	Neotropic	+	+	+	+	+	+	Wetterer 2011
*Tetramorium kheperra*	Unknown	+	+	+	+	+	+	Yamane and Jaitrong 2011
*Tetramorium lanuginosum*	Unknown	+	+	+	+	+	+	Wetterer 2010c
*Trichomyrmex destructor*	Indian subcontinent	+	+	+	+	+	+	Deyrup et al. 2000, Wetterer 2009
*Vollenhovia emeryi*	Japan							Wetterer et al. 2015

### Conclusions

In this work, based on an examination of museum specimens and published records represent approximately 1,000 species, only half of them are known species. This study presents an updated checklist of current ant species and subspecies with their distribution in Thailand. We identified many data gaps in taxonomic and spatial records among genera and regions. Currently, 529 valid species and subspecies in 109 genera among ten subfamilies are known from Thailand.

This study serves as: 1) a guide for collection in the poorly sampled northeast and central Thailand; 2) a comprehensive reference list with the endemic taxa and localities where conservation is an important priority of the resource for policy makers, conservation planners, and management of insect diversity in Thailand; and 3) a list of exotic ant species found in Thailand, which could possibly impact the ecological balance.

We encourage myrmecologists holding additional data on systematically collected ant assemblages to produce an updated data set. Also, new intensive surveys of Thai ants are being conducted, as well as unknown specimens being continuously identified to species level.

### Checklist of species

#### Subfamily Amblyoponinae [5 genera, 8 species]

***Myopopone castanea* (Smith, 1860)**

*Amblyopone castanea* Smith, 1860a: 105, pl. 1, fig. 6.

**Distribution. *Western***: Tak (Thung Yai Naresuan East WS, Umphang WS). ***Central***: Pitsanulok (Thung Salaeng Luang NP), Nakhon Nayok (Nang Rong). ***Eastern***: Chachoengsao (Khao Ang Reu Nai WS), Chanthaburi (Khao Soi Dao, Khlung Dist.), Trat (Ko Chang). ***Southern***: Nakhon Si Thammarat (Khao Nan NP, Khao Luang NP), Trang (Khao Chong BG), Songkhla (Ton Nga Chang WS).

**References.**Jaitrong and Nabhitabhata (2005), Jaitrong (2011).

***Mystrium camillae* Emery, 1889**

Figs 2, 3

*Mystrium camillae* Emery, 1889a: 491, pl. 10, figs 1–3.

**Distribution. *Northern***: Chiang Rai (Mae Fa Luang), Chiang Mai (Doi Suthep–Pui NP), Lampang (Tham Pha Thai NP), Mae Hong Son (Phang Ma Pha). ***Northeastern***: Loei (Phu Luang WS), Bueng Kan (Seka). ***Eastern***: Prachin Buri (Mueang Prachin Buri), Sakaeo (Pang Sida NP), Chanthaburi (Khao Soi Dao WS). ***Central***: Uthai Thani (Haui Kha Khaeng WS), Nakhon Nayok.

**Remarks.** New record.

**Material examined.** N Thailand, Chiang Rai Prov, Mae Fa Luang Dist, Doi Tung, 23.X.2017, W. Jaitrong leg., WJT230917–11 (THNHM); same locality and collector, 22.X.2018, WJT221018–02 (THNHM); N Thailand, Lampang Prov, Ngao Dist, Tham Pha Thai NP, 20.XII.2001 (THNHM); N Thailand, Mae Hong Son Prov, Phang Ma Pha Dist, 9.III.2008, W. Jaitrong leg. (THNHM); N Thailand, Chiang Mai Prov, Doi Suthep–Pui NP, S. Hasin leg. (SKYC); NE Thailand, Loei Prov, Phu Luang WS, 10.IV.2008, W. Jaitrong leg., TH08–WJT–501 (THNHM); same loc., 11.IV.2008, Sk. Yamane leg., TH08–SKY–87 (SKYC); NE Thailand, Bueng Kan Prov, Seka Dist, Sang Subdist, rubber plantation, 20.VI.2018, W. Jaitrong leg., WJT200618–17 (THNHM); same locality, date and collector, WJT200618–18 (THNHM); E Thailand, Prachin Buri, Mueang Prachin Buri Dist, Ban Nuen Hom, 21.X.2017, W. Jaitrong leg., WJT211017–1 (THNHM); E Thailand, Sa Kaeo Prov, Pang Sida NP, 11.IV.2008, unknown collector (THNHM); E Thailand, Chanthaburi Prov, Khao Soi Dao, 6.V.2006, Watana leg. (THNHM); C Thailand, Uthai Thani Prov, Ban Rai Dist, Huai Kha Khaeng WS, 6.II.2013, W. Jaitrong leg., TH13–WJT–017 (THNHM); C Thailand, Nakhon Nayok Prov, Nang Rong Temple, 10.VIII.2018, W. Jaitrong leg., WJT100818–2 (THNHM).

***Prionopelta kraepelini* Forel, 1905**

Figs 4, 5

*Prionopelta kraepelini* Forel, 1905: 3.

**Distribution. *Southern***: Surat Thani (Ratchaprapa Dam), Satun (near Phupha Phet Cave).

**Remarks.** New record.

**Material examined.** S Thailand, Surat Thani Prov, Ban Takhun Dist, Ratchaprapa Dam, Khlong Pae, 1.III.2019, W. Jaitrong leg., WJT010319–09 (THNHM); S Thailand, Satun Prov, Manang Dist, Ban Palm Pattana, near Phupha Phet Cave, 23.XII.2018, W. Jaitrong leg., WJT231218–24 (THNHM).

***Stigmatomma crenatum* (Xu, 2001)**

*Amblyopone crenata* Xu, 2001: 553, figs 18–20.

**Distribution. *Southern***: Trang (Khao Chong BG)

**References.**Antweb (2019).

***Stigmatomma quadratum* Karavaiev, 1935**

*Stigmatomma quadratum* Karavaiev, 1935: 58.

**Distribution.** Thailand (unknown locality).

**References.**Chapman and Capco (1951), Brown (1960).

***Stigmatomma reclinatum* (Mayr, 1879)**

*Amblyopone reclinata* Mayr, 1879: 667.

**Distribution. *Northeastern***: Chaiyaphum (Phu Khiao WS), Nakhon Ratchasima (Sakaerat, Khao Yai NP), Mukdahan (Phu Sithan WS). ***Central***: Saraburi (Phu Kae BG). ***Eastern***: Chachoengsao (Khao Ang Reu Nai WS), Chanthaburi (Khao Soi Dao WS). ***Southern***: Trang (Khao Chong BG).

**References.**Jaitrong and Nabhitabhata (2005, cited as *Amblyopone reclinata* Mayr, 1879), Jaitrong (2011, cited as *Amblyopone reclinata* Mayr, 1879)

***Stigmatomma rothneyi* (Forel, 1900)**

Amblyopone (Stigmatomma) rothneyi Forel, 1900b: 56.

**Distribution.** Northern: Chiang Mai (Doi Inthanon NP).

**References.**Antweb (2019).

***Xymmer phungi* Satria, Sasaki, Bui, Oguri, Syoji, Fisher, Yamane & Eguchi, 2016**

Figs 6, 7

*Xymmer phungi*Satria et al., 2016: 143, figs 1–11.

**Distribution. *Eastern***: Chachoengsao (Khao Ang Reu Nai WS).

**Remarks.** New record.

**Material examined.** E Thailand, Chachoengsao Prov, Thatakiab Dist, Khao Ang Reu Nai WS, Lumchangwat Station, 28.XII.2002, W. Jaitrong leg., WJT281202–01 (THNHM).

#### Subfamily Dolichoderinae [7 genera, 35 species]

***Dolichoderus affinis* Emery, 1889**

*Dolichoderus affinis* Emery, 1889a: 508, pl. 11, fig. 20.

**Distribution. *Northern***: Chiang Rai (Doi Tung), Chiang Mai (Khun Chang Khian, Pa Miang Village, Mae Taeng, Doi Suthep–Pui NP, Pha Hom Pok NP, Huai Nam Dang NP), Nan (Doi Phu Kha NP). ***Northeastern***: Loei (Phu Luang WS), Mukdahan (Phu Sithan WS).

**References.**Dill (2002), Onishi et al. (2016).

***Dolichoderus beccarii* Emery, 1887**

*Dolichoderus beccarii* Emery, 1887b: 253.

**Distribution. *Southern***: Narathiwat (Hala–Bala WS).

**References.**Jaitrong and Nabhitabhata (2005).

***Dolichoderus butteli* Forel, 1913**

Dolichoderus (Hypoclinea) butteli Forel, 1913: 89.

**Distribution. *Northern***: Chiang Mai (Doi Suthep–Pui NP, Doi Inthanon NP, Hod, Khun Chang Khian). ***Northeastern***: Loei (Phu Luang WS). ***Western***: Tak (Thung Yai Naresuan East WS, Umphang WS), Phetchaburi (KaeTao ng Kachan NP). ***Eastern***: Rayong (Khao Ang Reu Nai WS), Chanthaburi (Khao Soi Dao WS).

**References.**Onishi et al. (2016).

***Dolichoderus cuspidatus* (Smith, 1857)**

*Polyrhachis cuspidatus* Smith, 1857: 63.

**Distribution. *Southern***: Ranong (Khlong Na Kha WS), Trang (Khao Chong BG), Pattani (Nong Chik), Narathiwat (Hala–Bala WS, Toh Daeng).

**References.**Bingham (1906), Jaitrong and Nabhitabhata (2005).

***Dolichoderus erectilobus* Santschi, 1920**

Dolichoderus (Hypoclinea) erectilobus Santschi, 1920: 171, fig. 2.

**Distribution. *Northern***: Chiang Mai, Chiang Rai (Doi Mae Tho, Fang), Lampang (Doi Khun Tan), Phayao (Ban Pak Pok). ***Northeastern***: Loei (Phu Ruea).

**References.**Dill (2002).

***Dolichoderus feae* Emery, 1889**

*Dolichoderus feae* Emery, 1889a: 509, pl. 11, fig. 21.

**Distribution. *Northern***: Chiang Mai (Doi Ang Khang, Chiang Dao WS, Doi Suthep–Pui NP, Doi Inthanon NP, Nong Hoi), Chiang Rai (Mae Fa Luang, Doi Mae Tho), Nan (Doi Phukha NP), Mae Hong Son (Sop Pong). ***Western***: Tak (Umphang WS; Thung Yai Naresuan East WS), Prachuap Khiri Khan (Kaeng Krachan NP). ***Central***: Uthai Thani (Huai Kha Khaeng WS).

**References.**Dill (2002).

***Dolichoderus laotius* Santschi, 1920**

Dolichoderus (Hypoclinea) laotius Santschi, 1920: 170.

**Distribution. *Northeastern***: Ubon Ratchathani (Khong Chiam), Nakhon Ratchasima (Khao Yai NP; Sakaerat); Mukdahan (Phu Sithan WS). ***Eastern***: Chachoengsao (Khao Ang Reu Nai WS), Chanthaburi (Khao Soi Dao WS; Namtok Phlio NP).

**References.**Dill (2002).

***Dolichoderus semirugosus* (Mayr, 1870)**

*Hypoclinea semirugosus* Mayr, 1870: 956 (diagnosis in key).

**Distribution. *Northeastern***: Nakhon Ratchasima (Khao Yai NP). ***Eastern***: Chachoengsao (Khao Ang Reu Nai WS). ***Southern***: Yala.

**References.**Bingham (1906), Jaitrong and Nabhitabhata (2005).

***Dolichoderus siggii* Forel, 1895**

*Dolichoderus siggii* Forel, 1895: 465.

**Distribution. *Central***: unknown locality. ***Eastern***: Chachoengs (Khao Ang Reu Nai WS).

**References.**Forel (1895), Bolton (1995), Dill (2002).

***Dolichoderus sulcaticeps* (Mayr, 1870)**

*Hypoclinea sulcaticeps* Mayr, 1870: 957.

**Distribution. *Western***: Tak (Thung Yai Naresuan East WS, Huai Kha Khaeng WS). ***Northeastern***: Nakhon Ratchasima (Khao Yai NP). ***Central***: Uthai Thani (Huai Kha Khaeng WS), Bangkok*. ***Eastern***: Chachoengsao (Khao Ang Reu Nai WS). ***Southern***: Songkhla (Hat Yai).

**References.**Jaitrong and Nabhitabhata (2005).

***Dolichoderus taprobanae* (Smith, 1858)**

*Formica taprobane* Smith, 1858: 13.

**Distribution. *Northern***: Chiang Mai (Pha Hom Pok NP, Doi Chiang Dao WS), Nan (Doi Phu Kha NP). ***Western***: Tak (Thung Yai Naresuan East WS), Ratchaburi (Khao Lam). ***Northeastern***: Loei (Phu Luang WS). ***Central***: Uthai Thani (Huai Kha Khaeng WS).

**References.** Antweb (2019)

***Dolichoderus taprobanae siamensis* Forel, 1911**

Dolichoderus taprobanae var. siamensis Forel, 1911a: 46.

**Distribution. *Northern***: Chiang Mai*. ***Western***: Tak (Umphang WS), Phetchaburi (Kaeng Krachan NP). ***Northeastern***: Loei (Phu Luang WS).

**References.**Dill (2002).

***Dolichoderus thoracicus* (Smith, 1860)**

*Tapinoma thoracica* Smith, 1860b: 69.

**Distribution. *Northern***: Chiang Mai (Omkoi). ***Western***: Tak (Thung Yai Naresuan East WS, Umphang WS), Kanchanaburi (Thong Pha Phum NP), Prachuap Khiri Khan (Tab Sakae), Phetchaburi (Kaeng Krachan NP). ***Northeastern***: Loei (Phu Luang WS), Mukdahan (Phu Sithan WS), Nakhon Ratchasima (Sakaerat, Khao Yai NP, Forestry Camp). ***Central***: Uthai Thani (Huai Kha Khaeng WS), Saraburi (Phu Kae BG, Namtok Sam Lan NP), Pathum Thani (Khlong Luang, Nong Suar), Samut Prakan (Bang Krachao). ***Eastern***: Sa Kaeo (Pang Sida NP), Chachoengsao (Khao Ang Reu Nai WS), Chon Buri (Khao Ang Reu Nai WS), Rayong (Khao Ang Reu Nai WS), Chanthaburi (Khao Soi Dao WS, Pheao NP), Trat (Ko Kut). ***Southern***: Chumphon (Krom Luang Chumphon WS), Ranong (Khlong Na Kha WS), Surat Thani (Khlong Yan WS, Tai Rom Yen WS, Khlong Saeng WS), Nakhon Si Thammarat (Khao Nan NP, Khao Luang NP), Phuket (Thalang), Krabi (Ko Lanta NP), Trang (Ton Tae Waterfall, Palian, Khao Chong BG), Phatthalung (Khao Pu–Khao Ya NP), Songkhla (Ton Nga Chang WS, Songkhlanakarin University, Khao Nam Khang NP), Narathiwat (Hala–Bala WS).

**References.**Dill (2002), Jaitrong and Nabhitabhata (2005), Sakchoowong et al. (2008), Sakchoowong et al. (2009), Suwannaphak et al. (2016).

***Iridomyrmex anceps* (Roger, 1863)**

*Formica anceps* Roger, 1863: 164.

**Distribution. *Northern***: Chiang Rai (Doi Tung), Chiang Mai (Doi Pha Hom Pok NP, Chiang Dao WS, Mae Taeng, Doi Suthep–Pui NP, Doi Inthanon NP, Mae Chaem, Omkoi), Lampang (Tham Pha Thai NP). ***Western***: Tak (Thung Yai Naresuan East WS, Umphang WS), Kanchanaburi (Thong Pha Phum), Prachuap Khiri Khan (Kui Buri). ***Northeastern***: Loei (Phu Luang NP), Ubon Ratchathani (Khong Chiam), Kalasin (Phu Sithan WS), Chaiyaphum (Phu Khiao WS), Nakhon Ratchasima (Sakaerat, Khao Yai). ***Central***: Nakhon Sawan (Bueng Boraphet), Uthai Thani (Haui Kha Khaeng WS), Saraburi (Phu Kae BG, Namtok Namtok Sam Lan NP), Pathum Thani (Khlong Luang), Samut Prakan (Bang Krachao), Samut Songkhram (Mueang Samut Songkhram). ***Eastern***: Sa Kaeo (Pang Sida), Chachoengsao (Khao Ang Reu Nai WS), Rayong (Khao Ang Reu Nai WS), Chanthaburi (Khlung), Trat (Ko Kut, Ko Chang). ***Southern***: Chumphon (Krom Luang Chumphon WS), Phuket (Thalang), Ranong (Khlong Na Kha WS), Nakhon Si Thammarat (Khao Nan NP, Khao Luang NP), Surat Thani (Tai Rom Yen WS, Khlong Saeng WS), Krabi (Ko Lanta NP), Trang (Palian, Khao Chong BG), Phatthalung (Khao Pu–Khao Ya NP), Satun (Tarutao NP), Songkhla (Ton Nga Chang WS, Songkhlanakarin University, Khao Nam Khang NP), Narathiwat (Hala–Bala WS).

**References.**Jaitrong and Nabhitabhata (2005), Jaitrong and Ting–nga (2005), Jaitrong (2011), Jaitrong and Jeenthong (2014a), Suwannaphak et al. (2016).

***Ochetellus glaber* (Mayr, 1862)**

*Hypoclinea glaber* Mayr, 1862: 705.

**Distribution. *Northern***: Chiang Mai (Chiang Dao WS; Doi Inthanon NP). ***Central***: Pathum Thani (Khlong Luang), Bangkok (Bang Khen).

**References.**Jaitrong (2011), Suwannaphak et al. (2016).

***Philidris cordata* (Smith, 1859)**

*Formica cordata* Smith, 1859: 137.

**Distribution. *Northern***: Chiang Mai (unknown locality).

**References.**Antweb (2019).

***Philidris myrmecodiae* (Emery, 1887)**

Iridomyrmex cordatus var. myrmecodiae Emery, 1887a: 249.

**Distribution. *Northern***: Chiang Mai (unknown locality).

**References.**Forel (1911d), Forel (1911h).

***Tapinoma indicum* Forel, 1895**

*Tapinoma indicum* Forel, 1895: 472.

**Distribution. *Northern***: Nan (Si Nan NP). ***Western***: Kanchanaburi (Thong Pha Phum).

**References.**Chantarasawat et al. (2013), Torchote et al. (2010).

***Tapinoma melanocephalum* (Fabricius, 1793)**

*Formica melanocephalum* Fabricius, 1793: 353.

**Distribution. *Northern***: Chiang Rai (Doi Tung), Chiang Mai (Pa Miang Village, Doi Pha Hom Pok NP, Chiang Dao WS, Mae Taeng, Doi Suthep–Pui NP, Doi Inthanon NP, Mae Chaem, Omkoi), Lampang (Tham Pha Thai NP). ***Western***: Tak (Thung Yai Naresuan East WS, Umphang WS), Kanchanaburi (Thong Pha Phum), Prachuap Khiri Khan (Kui Buri). ***Northeastern***: Loei (Phu Luang NP, Phu Ruea Dist.), Ubon Ratchathani (Khong Chiam), Kalasin (Phu Sithan WS), Chaiyaphum (Phu Khiao WS), Nakhon Ratchasima (Sakaerat, Khao Yai). ***Central***: Bangkok, Uthai Thani (Haui Kha Khaeng WS), Saraburi (Phu Kae BG, Namtok Sam Lan NP), Pathum Thani (Khlong Luang), Samut Prakan (Bang Krachao), Samut Songkhram (Mueang Samut Songkhram). ***Eastern***: Sa Kaeo (Pang Sida), Chachoengsao (Khao Ang Reu Nai WS), Rayong (Khao Ang Reu Nai WS), Chanthaburi (Khlung, Namtok Phlio NP), Trat (Ko Kut, Ko Chang). ***Southern***: Chumphon (Krom Luang Chumphon WS), Ranong (Khlong Na Kha WS), Nakhon Si Thammarat (Khao Nan NP, Khao Luang NP), Surat Thani (Tai Rom Yen WS, Khlong Saeng WS), Phuket (Thalang), Krabi (Ko Lanta NP), Trang (Palian, Khao Chong BG), Phatthalung (Khao Pu–Khao Ya NP), Satun (Tarutao NP), Songkhla (Ton Nga Chang WS, Songkhlanakarin University, Khao Nam Khang NP), Pattani (Nong Chik), Narathiwat (Hala–Bala WS).

**References.**Bingham (1906), Jaitrong and Nabhitabhata (2005), Sakchoowong et al. (2009), Jaitrong (2011), Jaitrong and Jeenthong (2014a), Suwannaphak et al. (2016), Onishi et al. (2016).

***Technomyrmex albipes* (Smith, 1861)**

Formica (Tapinoma) albipes Smith, 1861: 38.

**Distribution. *Northern***: Chiang Mai (Pa Miang Village, Doi Suthep–Pui NP, Pha Hom Pok NP, Doi Ang Khang), Mae Hong Son (Pang Mapha), Lampang (Ngao), Lamphun (Mae Li Forest Plantation). ***Western***: Tak (Thung Yai Naresuan East WS, Tukasu Resort, Umphang WS), Phetchaburi (Kaeng Krachan NP), Prachuap Khiri Khan (Kui Buri). ***Northeastern***: Loei (Phu Luang WS), Kalasin (Phu Sithan WS), Mukdahan (Phu SithanWS), Bueng Kan (Bueng Kan Forest Plantation). ***Central***: Phetchabun (Bueng Sam Pan), Uthai Thani (Huai Kha Khaeng WS), Pathum Thani (Khlong Luang), Samut Prakan (Bang Krachao). ***Eastern***: Rayong (Mu Ko Man), Chanthaburi (Pheao NP, Khao Soi Dao WS), Trat (Trat Agroforestry Research Station, Ko Kut). ***Southern***: Phuket (Thalang), Krabi (Ko Lanta), Trang (Thung Khai BG), Nakhon Si Thammarat (Khao Nan NP, Phrom Khiri), Songkhla (Songkhlanakarin University), Pattani (Nong Chik).

**References.**Jaitrong and Nabhitabhata (2005), Jaitrong and Jeenthong (2014a), Suwannaphak et al. (2016), Onishi et al. (2016).

***Technomyrmex butteli* Forel, 1913**

*Technomyrme butteli* Forel, 1913: 97, fig. C.

**Distribution. *Western***: Phechaburi (Kaeng Krachan NP), Tak (Umphang WS). ***Eastern***: Chanthaburi (Khao Soi Dao WS). ***Southern***: Nakhon Si Thammarat (Khao Nan NP), Songkhla (Ton Nga Chang WS), Narathiwat (Hala–Bala WS).

**References.**Jaitrong and Nabhitabhata (2005).

***Technomyrmex brunneus* Forel, 1895**

Technomyrmex albipes r. brunneus Forel, 1895: 467.

**Distribution.** Northern: Lumphun (Mae Li Forest Plantation).

**References.**Collingwood (1962).

***Technomyrmex difficilis* Forel, 1892**

*Technomyrmex difficilis* Forel, 1892b: 242.

**Distribution. *Northern***: Chiang Rai (Doi Tung), Chiang Mai (Chiang Dao WS). ***Western***: Phetchaburi (Kaeng Krachan NP). ***Northeastern***: Kalasin (Phu Sithan WS), Nakhon Ratchasima (Sakaerat). ***Eastern***: Rayong (Khao Ang Reu Nai WS). ***Southern***: Ranong (Ngao), Krabi (Ko Lanta), Trang (Khao Chong BG), Songkhla (Ton Nga Chang WS).

**References.**Bolton (2007), Wetterer (2013), Jaitrong and Jeenthong (2014a).

***Technomyrmex elatior* Forel, 1902**

Technomyrmex modiglianii r. elatior Forel, 1902a: 293.

**Distribution. *Northern***: Chiang Mai (Chiang Dao WS, Doi Suthep–Pui NP). ***Western***: Tak (Umphang WS, Thung Yai Naresuan East WS), Kanchanaburi (Thong Pha Phum NP), Phetchaburi (Kaeng Krachan NP). ***Northeastern***: Loei (Phu Luang WS), Mukdahan (Phu Sithan WS), Nakhon Ratchasima (Khao Yai NP, Wang Nam Khiao). ***Central***: Phetchabun (Bueng Sam Pan). ***Eastern***: Chanthaburi (Khao Soi Dao WS), Rayong (Khao Ang Reu Nai WS). ***Southern***: Nakhon Si Thammarat (Khao Nan NP), Trang (Khao Chong BG).

**References.**Bolton (2007).

***Technomyrmex grandis* Emery, 1887**

Figs 8, 9

*Technomyrmex grandis* Emery, 1887b: 248.

**Distribution. *Southern***: Surat Thani (Tai Rom Yen NP), Phatthalung (Khao Pu–Khao Ya NP).

**Remarks.** New record.

**Material examined.** S Thailand, Surat Thani Prov, Ban Lumphun, Tai Rom Yen NP, 11.X.2011, W. Jaitrong leg., TH11–WJT–33 (THNHM); Phatthalung Prov, Si Banphot Dist, Khao Pu–Khao Ya NP, Riang Thong Waterfall, 28.IX.2007, W. Jaitrong leg., WJT07–TH2059 (THNHM).

***Technomyrmex horni* Forel, 1912**

*Technomyrmex horni* Forel, 1912d: 71.

**Distribution. *Western***: Tak (Thung Yai Naresuan East WS). ***Eastern***: Sa Kaeo (Pang Sida NP), Rayong (Mu Ko Man), Chachoengsao (Khao Ang Reu Nai WS), Chanthaburi (Khao Soi Dao WS). ***Southern***: Chumphon (Krom Luang Chumphon WS), Phuket (Thalang), Trang (Khao Chong BG), Songkhla (Ton Nga Chang WS).

**References.**Bolton (2007).

***Technomyrmex kraepelini* Forel, 1905**

*Technomyrmex kraepelini* Forel, 1905: 23.

**Distribution. *Northern***: Chiang Mai (Doi Suthep–Pui NP). ***Western***: Tak (Umphang WS, Thung Yai Naresuan East WS). ***Northeastern***: Loei (Phu Luang WS), Nakhon Ratchasima (Khao Yai WS). ***Southern***: Chumphon (Krom Luang Chumphon WS), Krabi (Ko Lanta), Satun (Tarutao NP), Pattani (Namtok Sai Khao NP).

**References.**Jaitrong and Nabhitabhata (2005), Bolton (2007), Sakchoowong et al. (2008), Jaitrong and Jeenthong (2014a).

***Technomyrmex lisae* Forel, 1913**

Figs 10, 11

*Technomyrmex lisae* Forel, 1913: 94, fig. D.

**Distribution. *Southern***: Surat Thani (Khlong Saeng WS), Trang (Khao Chong BG), Songkhla (Ton Nga Chang WS).

**Remarks.** New record.

**Material examined.** S Thailand, Surat Thani Prov, Khlong Saeng WS, 15.X.2011, Sk. Yamane leg., TH11–SKY–120 (SKYC, THNHM); S Thailand, Trang Prov, Khao Chong BG, 10.VIII.2009, WJT09–TH2030 (THNHM); S Thailand, Songkhla Prov, Ton Nga Chang WS, 25.VII.2004, WJT04–S019 (THNHM).

***Technomyrmex modiglianii* Emery, 1900**

*Technomyrmex modiglianii* Emery, 1900b: 696, fig. 12.

**Distribution. *Northern***: Chiang Rai (Doi Tung), Chiang Mai (Khun Chang Khian, Pa Miang Village, Omkoi), Nan (Nakhon Nan Forest Plantation). ***Western***: Tak (Um Piam, Thung Yai Naresuan East WS, Umphang WS), Kanchanaburi (Thong Pha Phum NP), Prachuap Khiri Khan (Pala–U Waterfall). ***Northeastern***: Loei (Phu Luang WS), Nakhon Ratchasima (Khao Yai WS, Wang Nam Khiao). ***Central***: Uthai Thani (Huai Kha Khaeng WS), Nakhon Nayok (Khao Yai NP). ***Eastern***: Chanthaburi (Pheao NP). ***Southern***: Chumphon (Krom Luang Chumphon WS), Surat Thani (Tai Rom Yen WS, Khlong Saeng WS), Nakhon Si Thammarat (Tapi Watershed Research Station, Khao Nan NP), Trang (Khao Chong BG), Songkhla.

**References.**Jaitrong and Nabhitabhata (2005), Bolton (2007), Onishi et al. (2016).

***Technomyrmex obscurior* Wheeler, 1928**

Technomyrmex schimmeri var. obscurior Wheeler, 1928c: 31.

**Distribution. *Northern***: Chiang Mai (Mae Chaem, Doi Suthep–Pui NP, Chiang Dao WS, Doi Ang Khang, Omkoi), Mae Hong Son (Phang Ma Pha). ***Western***: Tak (Thung Yai Naresuan East WS, Umphang WS), Phetchaburi (Kaeng Krachan NP). ***Northeastern***: Nakhon Ratchasima (Khao Yai WS). ***Central***: Uthai Thani (Huai Kha Khaeng WS). ***Eastern***: Chachoengsao (Khao Ang Reu Nai WS), Chanthaburi (Khao Soi Dao WS). ***Southern***: Nakhon Si Thammarat (Khao Nan NP).

**References.**Bolton (2007).

***Technomyrmex pratensis* (Smith, 1860)**

*Tapinoma pratensis* Smith, 1860a: 97.

**Distribution. *Northern***: Chiang Rai (Doi Tung), Chiang Mai (Pa Miang Village, Doi Suthep–Pui NP, Chiang Dao WS, Pha Hom Pok NP, Doi Ang Khang, Omkoi), Mae Hong Son (Tham Lot Forest Park). ***Western***: Tak (Thung Yai Naresuan East WS), Kanchanaburi (Khuean Srinagarindra NP, Thong Pha Phum NP), Phetchaburi (Kaeng Krachan NP). ***Northeastern***: Loei (Phu Luang WS). ***Central***: Uthai Thani (Haui Kha Khaeng WS). ***Eastern***: Prachin Buri (Khao Yai NP), Chon Buri (Ko Samaesarn), Chachoengsao (Khao Ang Reu Nai WS), Chanthaburi (Khlung, Pheao NP, Khao Soi Dao WS). ***Southern***: Surat Thani (rubber plantation).

**References.**Bolton (2007), Onishi et al. (2016).

***Technomyrmex reductus* Bolton, 2007**

Figs 12, 13

*Technomyrmex reductus* Bolton, 2007: 98, fig. 56.

**Distribution. *Western***: Phetchaburi (Kaeng Krachan WS). ***Southern***: Nakhon Si Thammarat (Khao Nan NP).

**Remarks.** New record.

**Material examined.** W Thailand, Phetchaburi Prov, Kaeng Krachan NP, 370 m alt., 24.VI.2014, Sk. Yamane & M. Maruyama leg., TH14–SKY–18 (SKYC, THNHM); S Thailand, Nakhon Si Thammarat Prov, Khao Nan NP, 16.IV.2007, W. Jaitrong leg., WJT07–TH661 (THNHM).

***Technomyrmex strenuus* Mayr, 1872**

Figs 14, 15

*Technomyrmex strenua* Mayr, 1872: 147.

**Distribution. *Western***: Phetchaburi (Kaeng Krachan WS).

**Remarks.** New record.

**Material examined.** W Thailand, Phetchaburi Prov, Kaeng Krachan NP, 370 m alt., 25.VI.2014, Sk. Yamane & M. Maruyama leg., TH14–SKY–43A (SKYC, THNHM).

***Technomyrmex vitiensis* Mann, 1921**

Technomyrmex albipes var. vitiensis Mann, 1921: 473.

**Distribution. *Northern***: Chiang Mai (Doi Suthep–Pui NP, Omkoi), Mae Hong Son (Phang Mapha). ***Western***: Kanchanaburi (Mae Khlong Watershed Research Station). ***Northeastern***: Nakhon Ratchasima (Sakaerat), Si Sa Ket (La–or Forest Plantation), Buri Ram (Mueang Buri Ram). ***Central***: Uthai Thani (Huai Kha Khaeng WS). ***Eastern***: Chanthaburi (Khao Soi Dao WS). ***Southern***: Nakhon Si Thammarat (Khao Nan NP), Songkhla (Kuan Khao Wang Forest Park).

**References.**Bolton (2007).

***Technomyrmex yamanei* Bolton, 2007**

*Technomyrmex yamanei* Bolton, 2007: 107, figs 47, 67.

**Distribution. *Northern***: Chiang Rai (Doi Tung), Chiang Mai (Pa Miang Village, Chiang Dao WS*, Pha Hom Pok NP), Lampang (Tham Pha Thai NP), Nan (Doi Phu Kha NP, Wiang Sa). ***Western***: Prachuap Khiri Khan. ***Northeastern***: Loei (Phu Luang WS), Chaiyaphum (Phu Khiao WS). ***Central***: Phetchabun (Bueng Sam Pan).

**References.**Bolton (2007), Onishi et al. (2016).

#### Subfamily Dorylinae [12 genera, 52 species]

***Aenictus artipus* Wilson, 1964**

*Aenictus artipus* Wilson, 1964: 449, fig. 60 (w).

**Distribution. *Northern***: Chiang Mai (Doi Ang Khang, Chiang Dao WS, Doi Suthep–Pui NP*, Doi Inthanon NP). ***Western***: Tak (Umphang WS). ***Northeastern***: Loei (Phu Luang WS), Chaiyaphum (Phu Khiao WS), Nakhon Ratchasima (Sakaerat). ***Eastern***: Prachin Buri (Khao Yai NP).

**References.** Jaitrong and Wiwatitaya (2006), Wilson (1964), Bolton (1995), Jaitrong and Nabhitabhata (2005), Jaitrong et al. (2010), Jaitrong (2013, 2015).

***Aenictus binghamii* Forel, 1900**

*Aenictus binghaniri* (sic) Forel, 1900a: 76 (w).

**Distribution. *Northern***: Chiang Mai (Doi Ang Khang, Chiang Dao WS, Mae Taeng, Doi Suthep–Pui NP, Doi Inthanon NP, Mae Chaem, Omkoi, Doi Luang NP, Mae Ai), Nan (Si Nan NP), Phrae (Wang Chin). ***Western***: Tak (Thung Yai Naresuan East WS, Umphang WS, Chiang Thong Forest Plantation), Kanchanaburi (Mae Khlong Watershed Research Station), Phetchaburi (Kaeng Krachan NP). ***Northeastern***: Mukdahan (Phu Sithan WS), Kalasin (Phu Sithan WS), Loei (Phu Luang WS), Chaiyaphum (Phu Khiao WS), Nakhon Ratchasima (Sakaerat, Khao Yai NP, Forestry Camp). ***Central***: Phitsanulok (Thung Salaeng Luang NP, Phu Soi Dao NP), Uthai Thani (Haui Kha Khaeng WS). ***Eastern***: Sa Kaeo (Pang Sida NP), Chachoengsao (Khao Ang Reu Nai WS), Chanthaburi (Pheao NP, Khao Soi Dao WS), Chon Buri (Khao Kheow), Rayong (Khao Chamao–Khao Wong NP), Trat (Ko Kut). ***Southern***: Nakhon Si Thammarat (Tapi Watershed Research Station, Khao Nan NP), Trang (Palian, Khao Chong BG), Songkhla (Khao Kho Hong), Narathiwat (Hala–Bala WS).

**References.** Jaitrong and Wiwatitaya (2006), Terayama and Kubota (1993), Jaitrong and Nabhitabhata (2005), Jaitrong and Ting–nga (2005), Jaitrong and Yamane (2011), Jaitrong et al. (2011a), Jaitrong (2013, 2015).

***Aenictus brevipodus* Jaitrong & Yamane, 2013**

Figs 16, 17

*Aenictus brevipodus* Jaitrong & Yamane, 2013: 176, figs 3A–C.

**Distribution. *Northern***: Chiang Mai (Omkoi).

**Remarks.** New record.

**Material examined.** N Thailand, Chiang Mai Prov, Omkoi Dist, Omkoi Forest, Dry Dipterocarp Forest (DDF), 16.VII.2016, W. Jaitrong leg., TH16–WJT–860 (THNHM).

***Aenictus camposi* Wheeler & Chapman, 1925**

*Aenictus camposi* Wheeler & Chapman, 1925: 48, pl. 1, figs 3, 4.

**Distribution. *Western***: Prachuap Kiri Khan (Pala–U waterfall), Phetchaburi (Kaeng Krachan NP). ***Central***: Phitsanulok (Thung Salaeng Luang NP), Uthai Thani (Haui Kha Khaeng WS). ***Northeastern***: Nakhon Ratchasima (Khao Yai NP). ***Eastern***: Sa Kaeo (Huai Nam Yen), Chachoengsao (Khao Ang Reu Nai WS), Chanthaburi (Khao Soi Dao WS).

**References.** Jaitrong and Wiwatitaya (2006), Jaitrong and Nabhitabhata (2005), Jaitrong et al. (2010), Jaitrong (2013, 2015).

***Aenictus changmaianus* Terayama & Kubota, 1993**

*Aenictus changmaianus* Terayama & Kubota, 1993: 68, figs 2–4.

**Distribution. *Northern***: Chiang Mai (Mae Taeng, Doi Suthep–Pui NP*, Chiang Dao WS, Chiang Mai University, Omkoi). ***Western***: Tak (Umphang WS). ***Northeastern***: Loei (Phu Luang WS), Nakhon Ratchasima (Sakaerat). ***Eastern***: Sa Kaeo (Pang Sida NP).

**References.**Terayama and Kubota (1993), Bolton (1995), Jaitrong and Hashimoto (2012), Jaitrong (2013, 2015).

***Aenictus concavus* Jaitrong & Yamane, 2013**

*Aenictus concavus* Jaitrong & Yamane, 2013: 178, fig. 4A–C.

**Distribution. *Northeastern***: Nakhon Ratchasima (Khao Yai NP*). ***Eastern***: Chathaburi (Pong Nam Ron).

**References.**Jaitrong and Yamane (2013), Jaitrong (2015).

***Aenictus cornutus* Forel, 1900**

*Aenictus cornutus* Forel, 1900a: 75.

**Distribution. *Southern***: Narathiwat (Wang).

**References.**Jaitrong (2015).

***Aenictus cylindripetiolus* Jaitrong & Yamane, 2013**

*Aenictus cylindripetiolus* Jaitrong & Yamane, 2013: 180, fig. 5A–G.

**Distribution. *Southern***: Phang–nga (Ton Chang Fa Waterfall, Khao Lak NP), Nakhon Si Thammarat (Khao Nan NP), Trang (Khao Chong BG*), Songkhla (Sadao).

**References.**Jaitrong (2015), Jaitrong and Yamane (2013).

***Aenictus dentatus* Forel, 1911**

Aenictus aitkeni var. dentatus Forel, 1911b: 383 (w).

**Distribution. *Southern***: Narathiwat (Hala–Bala WS).

**References.** Jaitrong and Wiwatitaya (2006), Jaitrong and Nabhitabhata (2005), Jaitrong et al. (2012), Jaitrong (2013, 2015).

***Aenictus doydeei* Jaitrong & Yamane, 2011**

*Aenictus doydeei* Jaitrong & Yamane, in Jaitrong et al. 2011a: 319, figs 7–9.

**Distribution. *Northern***: Chiang Mai (Chiang Dao WS, Omkoi). ***Western***: Tak (Thung Yai Naresuan East WS, Umphang WS), Kanchanaburi (Si Sawat). ***Northeastern***: Loei (Phu Luang WS), Chaiyaphum (Phu Khiao WS), Nakhon Ratchasima (Sakaerat). ***Eastern***: Chachoengsao (Khao Ang Reu Nai WS).

**References.**Jaitrong et al. (2011a); Jaitrong and Yamane (2012); Jaitrong (2013, 2015).

***Aenictus duengkaei* Jaitrong & Yamane, 2012**

*Aenictus duengkaei* Jaitrong & Yamane, 2012: 55, figs 2, 7A.

**Distribution. *Northern***: Lampang (Mae Moh Forest Plantation). ***Eastern***: Chachoengsao (Khao Ang Reu Nai WS*), Chon Buri (Si Racha).

**References.**Jaitrong and Yamane (2012); Jaitrong (2013, 2015).

***Aenictus fuchuanensis* Zhou, 2001**

*Aenictus fuchuanensis* Zhou, 2001: 231, figs 74, 75.

**Distribution. *Northern***: Chiang Rai (Doi Tung). ***Western***: Kanchanaburi (Thong Pha Phum NP). ***Northeastern***: Chaiyaphum (Phu Khiao WS), Nakhon Ratchasima (Sakaerat, Forestry Camp). ***Central***: Uthai Thani (Ban Rai), Nakhon Nayok (Nang Rong Waterfall). ***Eastern***: Sa Kaeo (Pang Sida NP), Chachoengsao (Khao Ang Reu Nai WS), Chanthaburi (Pheao NP, Khao Soi Dao WS), Trat (Ko Kut). ***Southern***: Ranong (Khlong Na Kha WS), Surat Thani (Tai Rom Yen NP), Nakhon Si Thammarat (Khao Nan NP), Trang (Khao Chong BG), Songkhla (Ton Nga Chang WS).

**References.**Jaitrong and Yamane (2013), Jaitrong (2013, 2015).

***Aenictus fulvus* Jaitrong & Yamane, 2011**

*Aenictus fulvus* Jaitrong & Yamane, 2011: 34, figs 28–30.

**Distribution. *Southern***: Nakhon Si Thammarat (Khao Nan NP*), Trang (Palian).

**References.**Jaitrong and Yamane (2011), Jaitrong (2013, 2015).

***Aenictus gracilis* Emery, 1893**

*Aenictus gracilis* Emery, 1893a: 187, pl. 8, fig. 1 (w).

**Distribution. *Western***: Tak (Thung Yai Naresuan East WS, Umphang WS), Kanchanaburi (Thong Pha Phum NP, Khuean Srinagarindra NP). ***Central***: Uthai Thani (Huai Kha Khaeng WS). ***Southern***: Ranong (Khlong Na Kha WS), Satun (Tarutao NP), Songkhla (Khao Nam Khang NP), Narathiwat (Toh Daeng).

**References.**Jaitrong and Yamane (2011), Jaitrong (2013, 2015).

***Aenictus hodgsoni* Forel, 1901**

Aenictus fergusoni var. hodgsoni Forel, 1901a: 474.

**Distribution. *Northern***: Chiang Mai (Doi Pha Hom Pok NP, Doi Ang Khang, Doi Inthanon NP), Mae Hong Son (Tham Lot Forest Park), Nan (Doi Phu Kha NP). ***Western***: Tak (Thung Yai Naresuan East WS, Umphang WS), Kanchanaburi (Si Sawat, Thong Pha Phum NP), Phetchaburi (Kaeng Krachan NP). ***Northeastern***: Loei (Phu Luang WS), Chaiyaphum (Phu Khiao WS), Nakhon Ratchasima (Sakaerat, Khao Yai NP). ***Central***: Pitsanulok (Thung Salaeng Luang NP), Uthai Thani (Haui Kha Khaeng WS), Saraburi (Khao Yai NP). ***Eastern***: Sa Kaeo (Pang Sida NP), Chachoengsao (Khao Ang Reu Nai WS), Chanthaburi (Khao Soi Dao WS), Rayong (Khao Chamao–Khao Wong NP). ***Southern***: Trang (Palian), Songkhla (Khao Nam Khang NP).

**References.**Jaitrong and Yamane (2011), Jaitrong et al. (2011a), Jaitrong (2013, 2015).

**Remarks.** All Thai specimens cited as *Aenictus fergusoni* Forel, 1901a in Jaitrong and Nabhitabhata (2005) should be reidentified as *A. hodgsoni* (sensu Jaitrong et al. 2011a, see also Jaitrong and Yamane 2011).

***Aenictus hottai* Terayama & Yamane, 1989**

*Aenictus hottai* Terayama & Yamane, 1989: 598, figs 1, 2 (w).

**Distribution. *Southern***: Nakhon Si Thammarat (Lansaka), Songkhla (Khao Kho Hong), Narathiwat (Hala–Bala WS).

**References.**Jaitrong and Nabhitabhata (2005); Wiwatwitaya and Jaitrong (2011); Jaitrong (2013, 2015).

***Aenictus jarujini* Jaitrong & Yamane, 2010**

*Aenictus jarujini* Jaitrong & Yamane, 2010: 329, figs 1, 2.

**Distribution. *Northern***: Chiang Mai (Omkoi), Mae Hong Son (Haui Nam Dang NP*).

**References.**Jaitrong and Yamane (2010); Jaitrong (2013, 2015).

***Aenictus khaoyaiensis* Jaitrong & Yamane, 2013**

*Aenictus khaoyaiensis* Jaitrong & Yamane, 2013: 194, fig. 10A–C.

**Distribution. *Western***: Tak (Thung Yai Naresuan East WS, Umphang WS). ***Northeastern***: Nakhon Ratchasima (Sakaerat, Khao Yai NP*).

**References.**Jaitrong (2015), Jaitrong and Yamane (2013).

***Aenictus laeviceps* (Smith, 1857)**

*Typhlatta laeviceps* Smith, 1857: 79 (w).

**Distribution. *Western***: Tak (Thung Yai Naresuan East WS, Umphang WS), Kanchanaburi (Thong Pha Phum NP). ***Eastern***: Sa Kaeo (Pang Sida NP), Chon Buri (Khao Ang Reu Nai WS), Rayong (Khao Ang Reu Nai WS), Chanthaburi (Pheao NP, Khao Soi Dao WS, Pong Nam Ron, Khao Khitchakut NP), Trat (Ko Kut). ***Southern***: Phang–nga (Khao Lak), Ranong (Khlong Na Kha WS), Surat Thani (Khlong Saeng WS), Nakhon Si Thammarat (Khao Nan NP), Krabi (Ko Lanta NP), Trang (Khao Chong BG, Thung Khai BG), Songkhla (Ton Nga Chang WS, Songkhlanakarin Campus), Narathiwat (Hala–Bala WS).

**References.**Jaitrong and Wiwatwitaya (2006), Jaitrong and Nabhitabhata (2005), Jaitrong and Yamane (2011), Jaitrong and Jeenthong (2014a), Jaitrong (2013, 2015).

***Aenictus leptotyphlatta* Jaitrong & Eguchi, 2010**

*Aenictus leptotyphlatta* Jaitrong & Eguchi, 2010: 14, figs 1, 2.

**Distribution. *Northern***: Chiang Mai (Chiang Mai University*). ***Western***: Kanchanaburi (Huai Mae Kha Min waterfall).

**References.**Jaitrong and Eguchi (2010), Jaitrong (2013, 2015).

***Aenictus longinodus* Jaitrong & Yamane, 2012**

*Aenictus longinodus* Jaitrong & Yamane, 2012: 59, figs 4, 7.

**Distribution. *Southern***: Nakhon Si Thammarat (Khao Luang NP), Trang (Palian, Khao Chong BG*), Songkhla (Ton Nga Chang WS, Songkhlanakarin University Campus).

**References.**Jaitrong and Yamane (2012), Jaitrong (2013, 2015).

***Aenictus maneerati* Jaitrong & Yamane, 2013**

*Aenictus maneerati* Jaitrong & Yamane, 2013: 201, fig. 13A–C.

**Distribution. *Northern***: Chiang Mai (Mae Yod). ***Western***: Tak (Umphang WS, Thung Yai Naresuan East WS*).

**References.**Jaitrong (2015), Jaitrong and Yamane (2013).

***Aenictus minutulus* Terayama & Yamane, 1989**

*Aenictus minutulus* Terayama & Yamane, 1989

**Distribution. *Southern***: Trang (Khao Chong BG), Narathiwat (Hala–Bala WS).

**References.**Jaitrong (2015).

***Aenictus nishimurai* Terayama & Kubota, 1993**

*Aenictus nishimurai* Terayama & Kubota, 1993: 70, figs 9, 10.

**Distribution. *Northern***: Chiang Mai (Doi Ang Khang, Doi Suthep–Pui NP*, Omkoi). ***Western***: Tak (Thung Yai Naresuan East WS), Kanchanaburi (Sai Yok NP). ***Eastern***: Chachoengsao (Khao Ang Reu Nai WS). ***Central***: Phitsanulok (Thung Salaeng Luang NP), Saraburi (Phu Kae BG).

**References.**Jaitrong and Wiwatwitaya (2006), Terayama and Kubota (1993), Jaitrong et al. (2011a), Jaitrong and Yamane (2012), Jaitrong (2013, 2015).

***Aenictus nuchiti* Jaitrong & Ruangsittichai, 2018**

*Aenictus nuchiti* Jaitrong & Ruangsittichai, 2018: 106, figs 1, 2, 4.

**Distribution. *Northern***: Chiang Mai (Omkoi National Forest*, near Maejo University Campus).

**References.**Jaitrong and Ruangsittichai (2018).

***Aenictus paradentatus* Jaitrong & Yamane, 2012**

*Aenictus paradentatus* Jaitrong & Yamane, in Jaitrong et al. 2012: 136, figs 6–12.

**Distribution. *Northern***: Chiang Mai (Doi Pha Hom Pok NP, Doi Ang Khang, Chiang Dao WS, Doi Suthep–Pui NP*), Mae Hong Son (Pang Mapha), Nan (Si Nan NP). ***Western***: Tak (Umphang WS, Thung Yai Naresuan East WS, Chiang Thong Forest Plantation), Prachuap Khiri Khan (Tab Sakae). ***Northeastern***: Chaiyaphum (Phu Khiao WS), Loei (Phu Luang WS), Nakhon Ratchasima (Sakaerat). ***Central***: Phitsanulok (Phu Soi Dao), Uthai Thani (Haui Kha Khaeng WS). ***Eastern***: Chachoengsao (Khao Ang Reu Nai WS), Chanthaburi (Pheao NP, Khao Soi Dao WS, Khlung).

**References.**Jaitrong et al. (2012), Jaitrong (2013, 2015).

***Aenictus parahuonicus* Jaitrong & Yamane, 2011**

*Aenictus parahuonicus* Jaitrong & Yamane, 2011: 19, figs 17–19.

**Distribution. *Northern***: Chiang Mai (Omkoi), Phrae (Wang Chin). ***Western***: Tak (Umphang WS). ***Northeastern***: Chaiyaphum (Phu Khiao WS), Nakhon Ratchasima (Khao Yai NP). ***Eastern***: Chachoengsao (Khao Ang Reu Nai WS), Chanthaburi (Khao Soi Dao WS). ***Southern***: Trang (Thung Khai BG*).

**References.**Jaitrong and Wiwatwitaya (2006), Jaitrong and Yamane (2011), Jaitrong (2013, 2015).

**Remarks.** All Thai specimens cited as *Aenictus huonicus* Wilson, 1964 in Jaitrong and Nabhitabhata (2005) and Jaitrong and Wiwatwitaya (2006) should be reidentified as *A. parahuonicus* in the present paper sensu, Jaitrong and Yamane (2011).

***Aenictus peguensis* Emery, 1895**

*Aenictus peguensis* Emery, 1895: 452.

**Distribution. *Northern***: Chiang Mai (Omkoi). ***Eastern***: Chon Buri (Nong Ta Yu Aboretum).

**References.** Khachonpisitsak and Lopwichan (2016)

***Aenictus pinkaewi* Jaitrong & Yamane, 2013**

*Aenictus pinkaewi* Jaitrong & Yamane, 2013: 207, fig. 16A–C.

**Distribution. *Northern***: Chiang Mai (Mueang Chiang Mai Dist*, Doi Ang Khang). ***Western***: Tak (Umphang WS, Thung Yai Naresuan East WS). ***Northeastern***: Chaiyaphum (Phu Khiao WS).

**References.**Jaitrong (2015), Jaitrong and Yamane (2013).

***Aenictus samungi* Jaitrong & Ruangsittichai, 2018**

*Aenictus samungi* Jaitrong & Ruangsittichai, 2018: 109, figs 3, 5.

**Distribution. *Western***: Tak (Thung Yai Naresuan East WS*).

**References.**Jaitrong and Ruangsittichai (2018).

***Aenictus siamensis* Jaitrong & Yamane, 2011**

*Aenictus siamensis* Jaitrong & Yamane, 2011: 42, figs 35–37.

**Distribution. *Northern***: Chiang Mai (Mae Taeng, Doi Suthep–Pui NP). ***Northeastern***: Chaiyaphum (Phu Khiao WS*), Loei (Phu Luang WS). ***Central***: Phitsanulok (Phu Soi Dao NP).

**References.**Jaitrong and Yamane (2011), Jaitrong (2013, 2015).

***Aenictus sonchaengi* Jaitrong & Yamane, 2011**

*Aenictus soncheangi* Jaitrong & Yamane, 2011: 43, figs 38–40.

**Distribution. *Southern***: Surat Thani (Ratchaprapa Dam), Nakhon Si Thammarat (Khao Nan NP*), Songkhla (Khao Kho Hong), Narathiwat (Hala–Bala WS).

**References.**Jaitrong and Yamane (2011), Jaitrong (2013, 2015).

***Aenictus stenocephalus* Jaitrong & Yamane, 2010**

*Aenictus stenocephalus* Jaitrong & Yamane, in Jaitrong et al. 2010: 41, figs 13–15.

**Distribution. *Northeastern***: Chaiyaphum (Phu Khiao WS*).

**References.**Jaitrong et al. (2010), Jaitrong (2013, 2015).

***Aenictus thailandianus* Terayama & Kubota, 1993**

*Aenictus thailandianus* Terayama & Kubota, 1993: 71, figs 11–13 (w).

**Distribution. *Northern***: Chiang Mai (Doi Ang Khang, Doi Suthep–Pui NP*).

**References.**Terayama and Kubota (1993), Jaitrong (2013, 2015), Jaitrong and Yamane (2013).

***Aenictus vieti* Jaitrong & Yamane, 2010**

*Aenictus vieti* Jaitrong & Yamane, in Jaitrong et al. 2010: 44, figs 11, 12, 15.

**Distribution. *Western***: Tak (Umphang WS).

**References.**Jaitrong et al. (2010), Jaitrong (2015).

***Aenictus watanasiti* Jaitrong & Yamane, 2013**

*Aenictus watanasiti* Jaitrong & Yamane, 2013: 213, fig. 18A–D.

**Distribution. *Northern***: Chiang Mai, (Khun Chang Khian, Doi Suthep–Pui NP*, Chiang Dao WS). ***Western***: Tak (Umphang WS, Thung Yai Naresuan East WS). ***Northeastern***: Nakhon Ratchasima (Khao Yai NP).

**References.**Jaitrong (2015), Jaitrong and Yamane (2013), Onishi et al. (2016).

***Aenictus wilaiae* Jaitrong & Yamane, 2013**

*Aenictus wilaiae* Jaitrong & Yamane, 2013: 215, fig. 19A–C.

**Distribution. *Northern***: Chiang Mai (Chiang Dao WS). ***Western***: Kanchanaburi (Sai Yok NP, Si Sawat), Phetchaburi (Kaeng Krachan NP). ***Northeastern***: Chaiyaphum (Phu Khiao WS), Nakhon Ratchasima (Sakaerat, Khao Yai NP), Trat (Ko Kut). ***Central***: Uthai Thani (Ban Rai), Samut Songkhram (Mueang Samut Songkhram). ***Eastern***: Chachoengsao (Khao Ang Reu Nai WS*), Chanthaburi (Khao Soi Dao WS). ***Southern***: Nakhon Si Thammarat (Khao Nan NP), Trang (Palian), Songkhla (Ton Nga Chang WS, Khao Kho Hong).

**References.**Jaitrong (2015), Jaitrong and Yamane (2013).

***Aenictus wiwatwitayai* Jaitrong & Yamane, 2013**

*Aenictus wiwatwitayai* Jaitrong & Yamane, 2013: 218, fig. 20A–C.

**Distribution. *Northeastern***: Nakhon Ratchasima (Sakaerat*). ***Eastern***: Chachoengsao (Khao Ang Reu Nai WS), Chanthaburi (Khao Soi Dao WS).

**References.**Jaitrong (2015), Jaitrong and Yamane (2013).

***Aenictus yamanei* Wiwatwitaya & Jaitrong, 2011**

Figs 18, 19

*Aenictus yamanei* Wiwatwitaya & Jaitrong, 2011: 562, figs 2A–D, 3.

**Distribution. *Western***: Phetchaburi (Kaeng Krachan WS), Prachuap Khiri Khan (Huai Sat Yai). ***Southern***: Surat Thani (Khlong Yan WS).

**Remarks.** New record.

**Material examined.** W Thailand, Phetchaburi Prov, Kaeng Krachan NP, 26.XII.2007, I. Chama (THNHM); S Thailand, Prachuap Khiri Khan Prov, Huai Sat Yai, upland rice field, 12.IX.2015, S. Chinkangsadarn leg., CFor003SSP1Q3 (THNHM); S Thailand, Surat Thani Prov, Vibhavadi Dist, Khlong Yan WS, 29.XII.2001, W. Jaitrong leg., WJT01–TH–14 (THNHM).

***Cerapachys sulcinodis* Emery, 1889**

*Cerapachys sulcinodis* Emery, 1889a: 493.

**Distribution. *Northern***: Chiang Mai (Chiang Dao WS, Mae Taeng, Doi Suthep–Pui NP), Tak (Thung Yai Naresuan East WS), Chaiyaphum (Phu Khiao WS). ***Northeastern***: Nakhon Ratchasima (Sakaerat, Khao Yai NP). ***Central***: Uthai Thani (Huai Kha Khaeng WS). ***Eastern***: Chachoengsao (Khao Ang Reu Nai WS), Chanthaburi (Khao Soi Dao WS). ***Southern***: Trang (Khao Chong BG), Narathiwat (Hala–Bala WS).

**References.**Jaitrong and Nabhitabhata (2005).

***Chrysapace costatus* (Bharti & Wachkoo, 2013)**

Figs 20, 21

*Cerapachys costatus* Bharti & Wachkoo, 2013: 1191, figs 4–6.

**Distribution. *Northern***: Chiang Mai (Chiang Dao WS). ***Western***: Tak (Umphang WS, Thung Yai Naresuan East WS), Kanchanaburi (Khuean Srinagarindra NP).

**Remarks.** New record.

**Material examined.** N Thailand, Chiang Mai Prov, Chiang Dao Dist, Chiang Dao WS, 21.IX.2013, W. Jaitrong leg., WJT210913–28 (THNHM); W. Jaitrong leg. (THNHM); Thailand, Tak Prov, Umphang Dist, Thung Yai Naresuan East WS, Unai Forest Ranger Station, 21.VI.20; W Thailand, Tak Prov, Mae Khlong Yai Village, Umphang WS, Grassland, 1050 m alt., 12.IX.2004, W. Jaitrong leg. (THNHM); W Thailand, Kanchanaburi Prov, Khuean Srinagarindra NP, Huai Mae Kamin Waterfall, 6.V.2014 [20104], W. Jaitrong leg., WJT060514–4 (THNHM).

***Dorylus laevigatus* (Smith, 1857)**

*Typhlopone laevigats* Smith, 1857: 70.

**Distribution. *Northern***: Chiang Mai (Huai Nam Dang NP, Doi Suthep–Pui NP, Doi Chiang Dao), Nan (Doi Phu Kha NP), Mae Hong Son (Pang Ma Pha). ***Western***: Tak (Umphang WS, Thung Yai Naresuan East WS). ***Central***: Uthai Thani (Huai Kha Khaeng WS). ***Southern***: Chumphon (Krom Luang Chumphon WS), Nakhon Si Thammarat (Khao Nan NP, Tha Sala), Krabi (Ko Lanta), Trang (Khao Chong BG, Thung Khai BG), Songkhla (Hat Yai), Narathiwat (Hala–Bala WS).

**References.**Jaitrong and Nabhitabhata (2005), Jaitrong et al. (2011b), Jaitrong and Jeenthong (2014a).

***Dorylus orientalis* Westwood, 1835**

*Dorylus orientalis* Westwood, 1835: 72.

**Distribution. *Northern***: Chiang Mai (Mueang Chiang Mai, Chom Thong, Doi Suthep–Pui NP, Doi Inthanon NP, Omkoi), Lampang (Ngao), Uttaradit (Nam Pad). ***Western***: Tak (Umphang WS). ***Northeastern***: Loei (Phu Luang WS), Chaiyaphum (Phu Khiao WS), Maha Sarakham (Borabue), Roi Et (unknown locality), Nakhon Ratchasima (Khao Yai NP). ***Central***: Pathum Thani (Khlong Luang). ***Eastern***: Chanthaburi (Pong Nam Ron, Khao Soi Dao WS). ***Southern***: Narathiwat (Hala–Hala WS), Ranong (Suk Samran).

**References.**Jaitrong and Nabhitabhata (2005), Jaitrong et al. (2011b), Suwannaphak et al. (2016).

***Dorylus vishnui* Wheeler, 1913**

*Dorylus vishnui* Wheeler, 1913: 233.

**Distribution. *Northern***: Chiang Mai (Omkoi, Doi Inthanon NP). ***Western***: Tak (Thung Yai Naresuan East WS), Phetchaburi (Kaeng Krachan WS). ***Northeastern***: Loei (Phu Luang WS), Nakhon Ratchasima (Pak Chong, Sakaerat). ***Eastern***: Sa Kaeo (Khao Ang Reu Nai WS), Chachoengsao (Khao Ang Reu Nai WS), Chanthaburi (Pong Nam Ron).

**References.**Jaitrong and Nabhitabhata (2005), Jaitrong et al. (2011b).

***Eusphinctus furcatus* Emery, 1893**

*Eusphinctus furcatus* Emery, 1893a: 275. Combination in *Eusphinctus*: Borowiec, 2016: 144.

**Distribution. *Northern***: Chiang Mai (Omkoi). ***Southern***: Trang (Khao Chong BG).

**References.**Jaitrong et al. (2016, cited as *Sphinctomyrmex furcatus* (Emery, 1893)).

***Lioponera longitarsus* Mayr, 1879**

*Lioponera longitarsus* Mayr, 1879: 667. Combination in *Cerapachys*: Brown, 1975: 23; in *Lioponera*: Borowiec, 2016: 164.

**Distribution. *Northeastern***: Nakhon Ratchasima (Khao Yai NP). ***Eastern***: Chanthaburi (Khao Soi Dao WS).

**References.**Jaitrong and Nabhitabhata (2005, cited as *Cerapachys longitarsus* (Mayr, 1879)), Jaitrong (2011, cited as *Cerapachys longitarsus* (Mayr, 1879)).

***Parasyscia dohertyi* (Emery, 1902)**

*Cerapachys dohertyi* Emery, 1902: 25. Combination in *Parasyscia*: Borowiec, 2016: 204.

**Distribution. *Western***: Tak (Thung Yai Naresuan East WS). ***Northeastern***: Nakhon Ratchasima (Khao Yai NP). ***Eastern***: Chachoengsao (Khao Ang Reu Nai WS), Chanthaburi (Khao Soi Dao WS), Trat (Ko Kut).

**References.**Jaitrong and Nabhitabhata (2005, cited as *Cerapachys dohertyi* Emery, 1902), Jaitrong (2011, cited as *Cerapachys dohertyi* Emery, 1902).

***Simopone oculata* Radchenko, 1993**

*Simopone oculata* Radchenko, 1993: 45, figs 4–6.

**Distribution. *Northern***: Chiang Mai (Chiang Mai University). ***Northeastern***: Loei (Phu Kradueng NP).

**References.**Antweb (2019, two specimens collected from Loei Prov, Phu Kradueng NP).

***Syscia chaladthanyakiji* Jaitrong, Wiwatwitaya & Yamane, 2020**

*Syscia chaladthanyakiji* Jaitrong, Wiwatwitaya & Yamane, 2020: 3, figs 1–6, 11.

**Distribution.**Westhern: Tak (Thung Yai Naresuan East WS). Central: Nakhonnayok (Nang Rong Temple).

**References.**Jaitrong et al. (2020).

***Syscia reticularis* Jaitrong, Wiwatwitaya & Yamane, 2020**

*Syscia reticularis* Jaitrong, Wiwatwitaya & Yamane, 2020: 6, figs 7–10.

**Distribution.** Southern: Nakhon Si Thammarat (Khao Luang NP).

**References.**Jaitrong et al. (2020).

***Yunodorylus sexspinus* Xu, 2000**

*Yunodorylus sexspinus* Xu, 2000: 298, figs 1–6. Combination in *Cerapachys*: Bolton, 2003: 269; in *Yunodorylus*: Borowiec, 2016: 237.

**Distribution. *Northern***: Chiang Mai (Mae Chaem). ***Western***: Tak (Thung Yai Naresuan East WS).

**References.**Jaitrong and Nabhitabhata (2005, cited as *Cerapachys sexspenus* (Xu, 2000), [misspelling]), Borowiec (2009).

***Zasphinctus siamensis* (Jaitrong, 2016)**

*Sphinctomyrmex siamensis* Jaitrong, in Jaitrong et al. 2016: 6, figs 5–8. Combination in *Zasphinctus*: Borowiec 2016: 243.

**Distribution. *Northern***: Chiang Mai (Mae Taeng*).

**References.**Jaitrong et al. (2016).

#### Subfamily Ectatomminae [1 genus, 7 species]

***Gnamptogenys bicolor* (Emery, 1889)**

Ectatomma (Stictoponera) bicolor Emery, 1889a: 493.

**Distribution. *Northern***: Chiang Mai (Pa Miang Village, Chiang Dao WS, Doi Suthep–Pui NP, Doi Inthanon NP, Mae Wang NP, Omkoi). ***Northeastern***: Loei (Phu Luang WS), Kalasin (Phu Sithan WS). ***Western***: Tak (Umphang WS, Thung Yai Naresuan East WS), Kanchanaburi (River Kwai Resort Hotel), Phetchaburi (Khang Krachan WS), Prachuap Khiri Khan (Kui Buri NP). ***Central***: Uthai Thani (Haui Kha Khaeng WS; Ban Rai Dist.). ***Eastern***: Chanthaburi (Khao Soi Dao WS, Pheao NP, Khao Ang Rue Nai WS). ***Southern***: Chumphon (Krom Luang Chumphon WS), Nakhon Si Thammarat (Khao Nan NP, Tapi Watershed Research Station), Trang (Khao Chong BG, Thung Khai BG, Ton Tae Waterfall).

**References.**Jaitrong and Nabhitabhata (2005), Lattke (2004), Onishi et al. (2016).

***Gnamptogenys binghamii* (Forel, 1900)**

Ectatomma (Stictoponera) binghamii Forel, 1900c: 317.

**Distribution. *Northern***: Chiang Mai (Doi Suthep–Pui NP, Doi Chiang Dao). ***Western***: Tak (Thung Yai Naresuan East WS, Umphang WS), Kanchanaburi (Sai Yok NP), Phetchaburi (Kaeng Krachan NP).

**Chetral**: Pathum Thani (Khlong Luang), Samut Prakan (Bang Krachao). ***Eastern***: Chachoengsao (Khao Ang Reu Nai WS), Chanthaburi (Khao Soi Dao, Namtok Phlio NP). ***Southern***: Krabi (Ko Lanta NP), Trang (Khao Chong BG, Palian, Thung Khai BG).

**References.**Jaitrong and Nabhitabhata (2005), Lattke (2004), Jaitrong and Jeenthong (2014a), Suwannaphak et al. (2016).

***Gnamptogenys chapmani* Brown, 1958**

*Gnamptogenys chapmani* Brown, 1958: 305.

**Distribution. *Western***: Phetchaburi (Kaeng Krachan NP).

**References.**Lattke (2004).

***Gnamptogenys coxalis* (Roger, 1860)**

*Ponera coxalis* Roger, 1860: 308.

**Distribution. *Northern***: Chiang Mai (Dao Chiang Dao WS). ***Western***: Tak (Umphang WS, Thung Yai Naresuan East WS). ***Northeastern***: Loei (Phu Luang WS), Nakhon Ratchasima (Khao Yai NP). ***Eastern***: Chachoengsao (Khao Ang Reu Nai WS), Chanthaburi (Khao Soi Dao WS). ***Southern***: Trang (Khao Chong BG).

**References.**Jaitrong and Nabhitabhata (2005), Lattke (2004).

REMARKS: Jaitrong and Nabhitabhata (2005) and Lattke (2004) identified specimens from Thailand as *Gnamptogenys costata* (Emery, 1889). It was later synonymized with *G. coxalis* by Lattke and Delsinne (2016).

***Gnamptogenys cribrata* (Emery, 1900)**

*Rhopalopone cribrata* Emery, 1900c: 311.

**Distribution. *Southern***: Trang (Khao Chong BG).

**References.**Lattke (2004).

***Gnamptogenys menadensis* (Mayr, 1887)**

Ectatomma (Stictoponera) menadensis Mayr, 1887: 539.

**Distribution. *Southern***: Ranong (Khlong Na Kha WS), Surat Thani (Khlong Yan WS, Khlong Saeng WS, Ratchaprapha Dam), Nakhon Si Thammarat (Khao Luang NP, Khao Nan NP, Lansaka), Trang (Khao Chong BG), Narathiwat (Hala–Bala WS).

**References.**Jaitrong and Nabhitabhata (2005).

***Gnamptogenys ortostoma* Lattke, 2004**

*Gnamptogenys ortostoma* Lattke, 2004: 139, fig. 31.

**Distribution. *Northern***: Chiang Mai (unknown locality name).

**References.**Lattke (2004).

#### Subfamily Formicinae [19 genera, 133 species]

***Acropyga acutiventris* Roger, 1862**

*Acropyga acutiventris* Roger, 1862: 243.

**Distribution. *Northern***: Chiang Rai (Doi Tung), Chiang Mai (Chiang Dao WS, Mae Taeng, Doi Suthep–Pui NP, Doi Inthanon NP, Mae Chaem), Lampang (Tham Pha Thai NP). ***Western***: Kanchanaburi (Sai Yok NP), Tak (Thung Yai Naresuan East WS, Umphang WS). ***Northeastern***: Loei (Na Duang, Phu Luang WS), Chaiyaphum (Phu Khiao WS), Nakhon Ratchasima (Sakaerat, Khao Yai NP), Ubon Ratchathani (Pha Taem NP). ***Central***: Nakhon Nayok (Nang Rong), Uthai Thani (Huai Kha Khaeng WS). ***Eastern***: Chachoengsao (Khao Ang Reu Nai WS), Chanthaburi (Pheao NP, Khao Soi Dao WS), Trat (Ko Kut). ***Southern***: Chumphon (Krom Luang Chumphon WS), Surat Thani (Khlong Saeng WS), Krabi (Ko Lanta), Trang (Khao Chong BG, Thung Khai BG), Phatthalung (Khao Pu–Khao Ya NP, Khao Bantad WS), Satun (Tarutao NP), Nakhon Si Thammarat (Kao Nan NP), Songkhla (Hat Yai, Ton Nga Chang NP).

**References.**Jaitrong and Nabhitabhata (2005), Jaitrong and Ting–nga (2005), LaPolla (2004), Jaitrong (2011), Jaitrong and Jeenthong (2014a).

***Acropyga butteli* Forel, 1912**

*Acropyga butteli* Forel, 1912b: 772.

**Distribution. *Northeastern***: Nakhon Ratchasima (Khao Yai NP). ***Central***: Saraburi (Phukae BG). ***Eastern***: Chachoengsao (Khao Ang Rue Nai WS), Chanthaburi (Khao Soi Dao WS). ***Southern***: Krabi (Ko Lanta NP).

**References.**Jaitrong and Jeenthong (2014a).

***Anoplolepis gracilipes* (Smith, 1857)**

*Formica gracilipes* Smith, 1857: 55.

**Distribution. *Northern***: Chiang Rai (Doi Tung), Chiang Mai (Khun Chang Khian, Pa Miang Village, Doi Ang Khang, Chiang Dao WS, Mae Taeng, Doi Suthep–Pui NP, Chiang Mai University Campus, Doi Inthanon NP, Mae Chaem), Phayao (Mae Ka), Lampang (Haui Tak, Tham Pha Thai NP), Phrae (Wang Chin Forest Plantation, Maorest Plantation), Nan (Doi Phu Kha NP, Nakhon Nan Forest Plantation). ***Western***: Tak (Umphang WS, Thung Yai Naresuan East WS, Lansang NP), Kanchanaburi (Thong Pha Phum NP), Phetchaburi (Kaeng Krachan NP), Prachuap Khiri Khan (Kui Buri). ***Northeastern***: Kalasin (Phu SithanWS), Chaiyaphum (Phu Khiao WS), Loei (Phu Luang WS), Nakhon Ratchasima (Sakaerat, Khao Yai NP, Buer Yai), Ubon Ratchathani (Pha Taem NP). ***Central***: Uthai Thani (Huai Kha Khaeng WS), Saraburi (Phukae BG, Namtok Sam Lan NP), Bangkok (Bang Khen, Chatuchak), Chachoengsao (Khao Ang Reu Nai WS), Pathum Thani (Khlong Luang), Samut Prakan (Bang Krachao), Samut Songkhram (Mueang Samut Songkhram). ***Eastern***: Chon Buri (Si Racha, Khao Kheow, Ko Samaesarn), Chanthaburi (Khao Soi Dao WS, Pheao NP, Khlung Dist.), Trat (Agricutural Research Station, Ko Kut). ***Southern***: Chumphon (Krom Luang Chumphon NP), Ranong (Khlong Na Kha WS), Surat Thani (Tai Rom Yen NP, Khlong Yan WS, Khlong Saeng WS), Nakhon Si Thammarat (Tapi Watershed Research Station, Khao Nan NP, Khao Luang NP, Krung Ching Waterfall), Krabi (Ko Lanta), Trang (Khao Chong BG, Thung Khai BG, Palian), Phatthalung (Khao Pu–Khao Ya NP, Khao Bantad WS), Satun (Tarutao NP), Songkhla (Khao Kho Hong, Khao Nam Khang NP), Narathiwat (Hala–Bala WS).

**References.**Jaitrong and Nabhitabhata (2005), Jaitrong and Ting–nga (2005), Sakchoowong et al. (2008), Sakchoowong et al. (2009), Jaitrong (2011), Jaitrong and Jeenthong (2014a), Suwannaphak et al. (2016), Onishi et al. (2016).

***Camponotus angusticollis* (Jerdon, 1851)**

*Formica angusticollis* Jerdon, 1851: 120.

**Distribution. *Southern***: Pattani (Nong Chik).

**References.**Bingham (1906).

***Camponotus arrogans* (Smith, 1858)**

*Formica arrogans* Smith, 1858: 23.

**Distribution. *Eastern***: Chachoengsao (Khao Ang Reu Nai WS). ***Southern***: Songkhla (Khao Nam Khang NP), Narathiwat (Hala–Bala WS).

**References.**Jaitrong and Nabhitabhata (2005).

***Camponotus aureus* Dumpert, 2006**

*Camponotus aureus* Dumpert, in Dumpert et al., 2006: 70, figs 1–4.

**Distribution. *Southern***: Surat Thani (Khao Sok NP*).

**References.**Dumpert et al. (2006).

***Camponotus auriventris* Emery, 1889**

*Camponotus auriventris* Emery, 1889b: 514.

**Distribution. *Northern***: Chiang Mai (Mae Chaem). ***Western***: Tak (Thung Yai Naresuan East WS), Phetchaburi (Kaeng Krachan NP). ***Northeastern***: Nakhon Ratchasima (Khao Yai NP), Loei (Phu Luang WS). ***Central***: Uthai Thani (Huai Kha Khaeng WS). ***Eastern***: Chachoengsao (Khao Ang Reu Nai WS), Trat (Ko Kut). ***Southern***: Ranong (Khlong Na Kha WS), Nakhon Si Thammarat (Khao Nan NP), Pattani (Nong Chik).

**References.**Bingham (1906), Jaitrong and Nabhitabhata (2005).

***Camponotus camelinus* (Smith, 1857)**

*Formica camelinus* Smith, 1857: 57.

**Distribution. *Eastern***: Chanthaburi (Pong Nam Ron Dist.). ***Southern***: Pattani (Nong Chik).

**References.**Bingham (1906), Jaitrong and Nabhitabhata (2005).

***Camponotus concurrens* Zettel & Laciny, 2018**

*Camponotus concurrens* Zettel & Laciny, in Zettel et al. 2018: 141–143, figs 22–31.

**Distribution. *Northeastern***: Nakhon Ratchasima (Khao Yai NP), Loei (Phu Luang WS). ***Eastern***: Chanthaburi (Pheao NP).

**References.** Zettel et al. (2018)

***Camponotus dolichoderoides* Forel, 1911**

*Camponotus dolichoderoides* Forel, 1911a: 51.

**Distribution. *Southern***: Narathiwat (Hala–Bala WS).

**References.**Jaitrong and Nabhitabhata (2005).

***Camponotus exiguoguttatus* Forel, 1886**

Camponotus sexguttatus subsp. exiguoguttatus Forel, 1886: 239.

**Distribution. *Central***: Bangkok (unknown locality).

**References.** Forel, 1892a.

***Camponotus irritabilis* (Smith, 1857)**

*Formica irritabilis* Smith, 1857: 56.

**Distribution. *Central***: Uthai Thani (Ban Rai). ***Southern***: Krabi (Ko Lanta NP), Trang (Khao Chong BG, Thung Khai BG), Satun (Tarutao NP), Songkhla (Ton Nga Chang WS).

**References.**Jaitrong and Jeenthong (2014a).

***Camponotus irritans* (Smith, 1857)**

*Formica irritans* Smith, 1857: 55

**Distribution. *Central***: Bangkok.

**References.** Forel, 1892a.

***Camponotus khaosokensis* Dumpert, 2006**

*Camponotus khaosokensis* Dumpert, 2006: 89. Replacement name for *Camponotus hoelldobleri* Dumpert in Dumpert et al. 2006: 71, figs 5–8.

**Distribution. *Southern***: Surat Thani (Khao Sok NP*, Khlong Yan WS).

**References.**Dumpert (2006), Dumpert et al. (2006).

***Camponotus lasiselene* Wang & Wu, 1994**

*Camponotus lasiselene* Wang & Wu, 1994: 24, fig. 3.

**Distribution. *Northern***: Chiang Mai (Pa Miang Village, Chiang Dao WS, Doi Suthep–Pui NP, Mae Chaem, Doi Inthanon NP), Lampang (Tham Pha Thai NP, Haui Tak), Nan (Doi Phu Kha NP). ***Western***: Tak (Thung Yai Naresuan East WS, Doi Mu Sur Market, Umphang WS). ***Northeastern***: Loei (Phu Luang WS), Chaiyaphum (Phu Khiao WS), Nakhon Ratchasima (Sakaerat, Khao Yai NP). ***Central***: Bangkok (Bang Khen). ***Eastern***: Chachoengsao (Khao Ang Reu Nai WS), Chon Buri (Si Racha), Chanthaburi (Khao Soi Dao WS, Pheao NP).

**References.**Jaitrong and Nabhitabhata (2005), Onishi et al. (2016).

***Camponotus mitis* (Smith, 1858)**

*Formica mitis* Smith, 1858: 20.

**Distribution. *Southern***: Yala.

**References.**Bingham (1906).

***Camponotus mutilarius* Emery, 1893**

Figs 22–23

Camponotus wasmanni var. mutilarius Emery, 1893b: 224. Revised to species by Wachkoo 2015: 383.

**Distribution. *Northern***: Nan (Doi Phu Kha NP).

**Remarks.** New record.

**Material examined.** N Thailand, Nan Prov, Doi Phu Kha NP, 1300m, 29.V.2004, W. Jaitrong leg. (THNHM).

***Camponotus nicobarensis* Mayr, 1865**

*Camponotus nicobarensis* Mayr, 1865: 31, pl. 1, fig. 1.

**Distribution. *Northern***: Chiang Mai (Pa Miang Village, Chiang Dao WS, Omkoi), Mae Hong Son (Tham Lot Forest Park). ***Western***: Tak (Umphang WS, Thung Yai Naresuan East WS), Prachuap Khiri Khan (Kaeng Krachan NP). ***Northeastern***: Loei (Phu Luang WS), Nakhon Ratchasima (Khao Yai NP). ***Central***: Uthai Thani (Huai Kha Khaeng WS), Phathum Thani (Khlong Luang). ***Eastern***: Chachoengsao (Khao Ang Rue Nai WS), Chanthaburi (Khao Soi Dao WS, Khao Ang Rue Nai WS). ***Southern***: Chumphon (Krom Luang Chumphon WS), Krabi (Ko Lanta NP), Nakhon Si Thammarat (Khao Nan NP), Trang (Khao Chong BG), Narathiwat (Hala–Bala WS).

**References.**Jaitrong and Nabhitabhata (2005), Jaitrong and Jeenthong (2014a), Suwannaphak et al. (2016), Onishi et al. (2016).

***Camponotus oblongus* (Smith, 1858)**

*Formica oblonga* Smith, 1858b: 21

**Distribution.** Unknown.

**References.**Chhotani and Maiti (1977).

***Camponotus paraleonardi* Zettel & Yamane, 2018**

*Camponotus paraleonardi* Zettel & Yamane, in Zettel et al. 2018: 169, figs 64–67.

**Distribution. *Southern***: Phang–nga (Khao Lak NP*)

**References.** Zettel et al. (2018)

***Camponotus parius* Emery, 1889**

Camponotus micans r. paria Emery, 1889b: 513.

**Distribution. *Southern***: Yala.

**References.** Binghami (1906).

***Camponotus rufifemur* Emery, 1900**

*Camponotus rufifemur* Emery, 1900b: 705.

**Distribution. *Western***: Tak (Umphang WS, Thung Yai Naresuan East WS). ***Southern***: Nakhon Si Thammarat (Khao Nan NP), Trang (Thung Khai BG), Satun (Tarutao NP), Narathiwat (Hala–Bala WS).

**References.**Jaitrong and Nabhitabhata (2005).

***Camponotus rufoglaucus* (Jerdon, 1851)**

*Formica rufoglaucus* Jerdon, 1851: 124.

**Distribution. *Northern***: Chiang Rai (Doi Tung), Chiang Mai (Doi Ang Khang, Doi Luang Chiang Dao, Chiang Dao WS, Mae Taeng, Doi Suthep–Pui NP, Chiang Mai University Campus, Doi Inthanon NP, Mae Chaem, Omkoi), Phayao (Mae Ka), Lampang (Haui Tak, Tham Pha Thai NP), Phrae (Wang Chin Plantation), Lamphun (Mae Li Forest Plantation), Nan (Doi Phu Kha NP, Nakhon Nan Forest Plantation). ***Western***: Tak (Umphang WS, Thung Yai Naresuan East WS, Lansang NP, Thung Yai Naresuan East WS), Kanchanaburi (Thong Pha Phum), Phetchaburi (Kaeng Krachan NP), Prachuap Khiri Khan (Kui Buri). ***Northeastern***: Kalasin (Phu Sithan WS), Chaiyaphum (Phu Khiao WS), Loei (Phu Luang WS), Nakhon Ratchasima (Sakaerat, Khao Yai NP, Buer Yai), Ubon Ratchathani (Pha Taem NP). ***Central***: Saraburi (Phukae BG, Namtok Sam Lan NP), Bangkok (Bang Khen, Chatuchak), Uthai Thani (Huai Kha Khaeng WS), Pathum Thani (Khlong Luang Dist.), Samut Prakan (Bang Krachao), Samut Songkhram (Mueang Samut Songkhram). ***Eastern***: Chachoengsao (Khao Ang Reu Nai WS), Chon Buri (Si Racha, Khao Kheow), Chanthaburi (Khao Soi Dao WS, Pheao NP), Trat (Ko Kut). ***Southern***: Chumphon (Krom Luang Chumphon WS), Ranong (Khlong Na Kha WS), Surat Thani (Tai Rom Yen NP, Khlong Yan WS, Khlong Saeng WS), Nakhon Si Thammarat (Khao Nan NP, Khao Luang NP, Krung Ching Waterfall), Krabi (Ko Lanta), Trang (Khao Chong BG, Thung Khai BG, Palian), Phatthalung (Khao Pu–Khao Ya NP, Khao Bantad WS), Satun (Tarutao NP), Songkhla (Khao Kho Hong, Khao Nam Khang NP), Narathiwat (Hala–Bala WS).

**References.**Jaitrong and Nabhitabhata (2005), Sakchoowong et al. (2009), Jaitrong and Jeenthong (2014a), Suwannaphak et al. (2016).

**Remarks.** No taxonomic revision is available on the *C. rufoglaucus* group (*Myrmosericus*) in continental Asia. According to Bingham (1903), *Camponotus dolendus* Forel, 1892a is a highland species, while *C. rufoglaucus* and *C. parius* occur in lower elevations and found together. We have applied the name *C. rufoglaucus* to the populations of continental Asia, adopting a wider concept of *C. rufoglaucus*. Careful comparison of all the castes and sexes is needed to evaluate the status of these three forms.

***Camponotus schoedli* Dumpert, 2006**

*Camponotus schoedli* Dumpert, in Dumpert et al. 2006: 74, figs 14, 15.

**Distribution. *Southern***: Surat Thani (Khao Sok NP*).

**References.**Dumpert et al. (2006).

***Camponotus schulzianus* Zettel & Balàka, 2018**

*Camponotus schulzianus* Zettel & Balàka, in Zettel et al. 2018: 134, figs 11–14.

**Distribution. *Southern***: Phang–nga (Khao Lak NP*).

**References.** Zettle et al. (2018).

***Camponotus sericeus* (Fabricius, 1798)**

*Formica cericea* Fabricius, 1798: Supplementum Entomologiae Systematical: 279, (w.).

**Distribution. *Northern***: Chiang Mai (Doi Suthep–Pui), Lampang (Tham Pha Thai, Ngao) ***Northeastern***: Nakhon Ratchasima (Khao Yai, Sakaerat)

**References.**Jaitrong and Nabhitabhata (2005).

***Camponotus singularis* (Smith, 1858)**

*Formica singularis* Smith, 1858: 27.

**Distribution. *Northern***: Chiang Rai (Mae Fa Luang), Chiang Mai (Chiang Dao WS, Mae Taeng, Mae Chaem, Omkoi), Nan (Nakhon Nan Forest Plantation). ***Northeastern***: Nakhon Ratchasima (Khao Yai NP). ***Southern***: Ranong (Khlong Na Kha WS), Phatthalung (Khao Pu–Khao Ya NP), Narathiwat (Hala–Bala WS).

**References.**Jaitrong and Nabhitabhata (2005).

***Camponotus sophiae* Zettel & Balàka, 2018**

*Camponotus sophiae* Zettel & Balàka, in Zettle et al. 2018: 149, figs 36–39.

**Distribution. *Southern***: Phang–nga (Khao Lak NP*)

**References.** Zettel et al. (2018)

***Camponotus weiserti* Zettel & Laciny, 2018**

*Camponotus weiserti* Zettel & Laciny, in Zettle et al. 2018: 144, figs 32–35.

**Distribution. *Southern***: Phang–nga (Khao Lak NP*)

**References.** Zettel et al. (2018)

***Cladomyrma petalae* Agosti, 1991**

Figs 24, 25

*Cladomyrma petalae* Agosti, 1991: 308, figs 13, 16, 20, 25, 26.

**Distribution. *Southern***: Narathiwat (Hala–Bala WS).

**Remarks.** New record.

**Material examined.** S Thailand, Narathiwat Prov, Hala–Bala WS, 15.IX.2000, S. Hasin leg. (THNHM).

***Cladomyrma sirindhornae* Jaitrong, Laedprathom & Yamane, 2013**

*Cladomyrma sirindhornae*Jaitrong et al. 2013: 15, figs 1–4.

**Distribution. *Northeastern***: Nakhon Ratchasima (Sakaerat). ***Eastern***: Chachoengsao (Khao Ang Reu Nai WS), Rayong (Khao Ang Reu Nai WS), Chanthaburi (Khao Soi Dao WS, Pheao NP, Ban Ang–Ed Commutity Forest development Project*), Trat (Agroforestry Research Station). ***Central***: Nakhon Nayok (Nang Rong Waterfall), Saraburi (Kok E–dok Waterfall).

**References.**Jaitrong et al. (2013).

***Colobopsis badia* (Smith, 1857)**

*Formica badia*Smith. 1857: 54. Combination in *Colobopsis*: Ward et al. 2016: 350.

**Distribution. *Southern***: Trang (Khao Chong BG).

**References.** Laciny and Zettel (2018), Zettel et al. (2018).

***Colobopsis cylindrica* (Fabricius, 1798)**

*Formica cylindrica* Fabricius, 1798: 280. Combination in *Colobopsis*: Ward et al. 2016: 350.

**Distribution. *Southern***: Ranong (Khlong Na Kha WS), Nakhon Si Thammarat (Khao Nan NP).

**References.**Jaitrong and Nabhitabhata (2005, cited as *Camponotus cylindricus* (Fabricius, 1798)).

***Colobopsis explodens* Laciny & Zettel, 2018**

*Colobopsis explodens* Laciny & Zettel, in Laciny et al. 2018: 10, figs 2–7.

**Distribution. *Southern***: Chumphon (Krom Luang Chumphon*).

**References.**Laciny et al. (2018).

***Colobopsis leonardi* (Emery, 1889)**

*Camponotus leonardi* Emery, 1889b: 515, pl. 11, figs 22, 23. Combination in *Colobopsis*: Ward et al., 2016: 350.

**Distribution. *Northern***: Chiang Mai (Pa Miang Village, Chiang Dao WS, Doi Suthep–Pui NP, Doi Inthanon NP, Obluang NP, Omkoi), Lampang (Tham Pha Thai NP, Mae Yod), Nan (Nakhon Nan Forest Plantation). ***Western***: Tak (Thung Yai Naresuan East WS, Umphang WS). ***Northeastern***: Kalasin (Phu Sithan WS), Loei (Phu Luang WS), Nakhon Ratchasima (Sakaerat, Khao Yai NP). ***Central***: Pitsanulok (Thung Salaeng Luang NP), Uthai Thani (Huai Kha Khaeng WS). ***Eastern***: Chachoengsao (Khao Ang Reu Nai WS), Chanthaburi (Khao Soi Dao WS), Trat (Ko Kut). ***Southern***: Surat Thani (Tai Rom Yen NP), Nakhon Si Thammarat (Khao Nan NP), Krabi (Ko Lanta), Trang (Khao Chong BG, Thung Khai BG), Songkhla (Khao Nam Khang NP), Pattani, Narathiwat (Hala–Bala WS).

**References.**Bingham (1906, cited as *Colobopsis pubescens* Mayr, 1862, junior synonym of *C. leonardi*), Jaitrong and Nabhitabhata (2005, cited as *Camponotus leonadi* Emery, 1889 [misspelling]), Jaitrong and Jeenthong (2014a), Onishi et al. (2016), Zettel et al. (2018).

***Colobopsis markli* Dumpert, 2004**

*Camponotus markli* Dumpert, in Maschwitz et al. 2004: 44. Type locality: THAILAND (Eastern Thailand). Combination in *Colobopsis*: Ward et al., 2016: 350.

**Distribution. *Eastern***: Chanthaburi (Khao Chamao–Khao Wong NP*).

**References.**Maschwitz et al. (2004).

***Colobopsis saundersi* (Emery, 1889)**

*Camponotus saundersi* Emery, 1889b: 516. Combination in *Colobopsis*: Ward et al., 2016: 350.

**Distribution. *Western***: Tak (Umphang WS). ***Northeastern***: Chaiyaphum (Phu Khiao WS), Nakhon Ratchasima (Khao Yai NP, Buer Yai). ***Eastern***: Chachoengsao (Khao Ang Reu Nai WS), Chanthaburi (Khao Soi Dao WS, Pheao NP) ***Southern***: Nakhon Si Thammarat (Khao Luang NP, Khao Nan NP), Krabi (Ko Lanta), Trang (Khao Chong BG), Narathiwat (Hala–Bala WS).

**References.**Jaitrong and Nabhitabhata (2005, cited as *Camponotus saundersi* Emery, 1889), Jaitrong and Jeenthong (2014, cited as *Camponotus saundersi* Emery, 1889).

***Colobopsis vitrea* (Smith, 1860)**

*Formica vitrea* Smith, 1860a: 94. Combination in *Colobopsis*: Ward et al., 2016: 350.

**Distribution. *Eastern***: Chanthaburi (Khao Soi Dao WS, Kung Krabaen). ***Southern***: Songkhla (Ton Nga Chang WS).

**References.**Jaitrong and Nabhitabhata (2005, cited as *Camponotus vitreus* (Smith, 1860)).

***Colobopsis vitrea praerufa* (Emery, 1900)**

Camponotus vitreus var. praerufa Emery, 1900b: 707.

**Distribution. *Northeastern***: Nakhon Ratchasima (Khao Yai NP).

**References.**Suriyapong (2003).

***Dinomyrmex gigas* (Latreille, 1802)**

*Formica gigas* Latreille, 1802: 105, pl. 2, fig. 6. Combination in *Dinomyrmex*: Ward et al., 2016: 355.

**Distribution. *Southern***: Nakhon Si Thammarat (Khao Nan NP, Khao Luang NP, Krung Ching Waterfall), Trang (Khao Chong BG, Thung Khai BG, Ton Tae Waterfall, Sai Rung Waterfall), Ranong (Khlong Na Kha WS), Songkhla (Ton Nga Chang WS, Khao Nam Khang NP), Pattani (Namtok Sai Khao NP), Narathiwat (Toh Daeng, Hala–Bala WS).

**References.**Bingham (1906), Jaitrong and Nabhitabhata (2005, cited as *Camponotus gigas* (Latreille, 1802)), Jaitrong & Ting–Nga (2005, cited as *Camponotus gigas* (Latreille, 1802)).

***Echinopla charernsomi* Tanansathaporn & Jaitrong, 2018**

*Echinopla charernsomi* Tanansathaporn & Jaitrong, in Tanansathaporn et al. 2018: 3, figs 1–3.

**Distribution. *Western***: Tak (Thung Yai Naresuan East WS). ***Northeastern***: Nakhon Ratchasima (Sakaerat Environmental Research Station*). ***Central***: Nakhon Nayok (Mueang Nakhon Nayok).

**References.**Tanansathaporn et al. (2018).

***Echinopla cherapunjiensis* Bharti & Gul, 2012**

Figs 26, 27

*Echinopla cherapunjiensis* Bharti & Gul, 2012: e–53, figs 1–3.

**Distribution. Northesatern**: Loei (Phu Luang), Pitsanulok (Phu Soi Dao NP). ***Western***: Tak (Umphang), Phechaburi (Kaeng Krachan NP), Tak (Tung Yai WS). ***Southern***: Nakhon Si Thammarat (Khao Nan NP), Narathiwat (Toh Daeng Swamp Forest).

**Remarks.** New record.

**Material examined.** Loei Prov, Phu Luang WS, 15.V.2007, S. Hasin leg., SH07–TH–101 (THNHM); same locality, and collection, 9.V.2007, SH07–TH–15 (THNHM); same locality, date and collector, SH07–TH–9 THNHM); Pitsanulok Prov, Phu Soi Dao NP, 8.VII.2006, D. Wiwatwitaya leg., (THNHM); Phetchaburi Prov, Kaeng Krachan NP, 900 m alt., 29.VI.2014, Sk. Yamane & M. Maruyama leg.: same locality, 28.XI.2006, W. Sanguansombat leg., WS280906–1 (THNHM); Tak Prov, Tung Yai WS, DEF, 25.V.2000, W. Jaitrong leg. (SKYC, THNHM): same locality and collector, 19.II.2015, TH15–WJT–383 (THNHM); same locality and collector, 23.VI.2015 (THNHM); Nakhon Si Thammarat Prov, Khao Nan NP, San Yen, hill evergreen forest (HEF), 22.IV.2007, W. Jaitrong leg. (THNHM); same locality, 500–900 m a.s.l., evergreen forest, 21.VII.2005, N. Noon–anant leg. (PSU); Narathiwat Prov, Su–ngai Kolok Dist, Toh Dang Swamp Forest, 12.X.2000, S. Hasin leg. (THNHM).

***Echinopla fisheri* Zettel & Laciny, 2015**

Figs 28, 29

*Echinopla fisheri* Zettel & Laciny, 2015: 108, figs 17–20.

**Distribution. *Eastern***: Chanthaburi (Pheao NP). ***Southern***: Phang–nga (Ton Pariwat waterfall), Trang (Ton Tae Waterfall), Songkhla (Khao Nam Khang NP), Narathiwat (Hala–Bala WS).

**Remarks.** New record.

**Material examined.** Chanthaburi Prov, Pheao NP, evergreen forest, 24.XII.2003, W. Jaitrong leg. (THNHM); Phang–nga Prov, Ton Wariat WS, 300–380 m alt., 20.IV.2005, N. Noon–anant leg. (SKYC); Trang Prov, Palian Dist, Ton Tae Waterfall, 200–300 m alt., 28.III.2005, W. Jaitrong leg. (THNHM); Songkhla Prov, Khao Nam Khang NP, evergreen forest, 27.II.2005, N. Noon–anant leg. (PSU); Narathiwat Prov, Wang Dist, Bala Forest, 0–200 m alc., 26.IX.2001, N. Noon–anant leg. (PSU).

***Echinopla jeenthongi* Tanansathaporn & Jaitrong, 2018**

*Echinopla jeenthongi* Tanansathaporn & Jaitrong, in Tanansathaporn et al. 2018: 6, figs 4–9.

**Distribution. *Southern***: Nakhon Si Thammarat (Khao Nan NP*), Surat Thani (Ban Na San), Phang–nga (Mueang Phang–nga), Narathiwat (Hala–Bala WS).

**References.**Tanansathaporn et al. (2018).

***Echinopla lineata* Mayr, 1862**

Figs 30, 31

*Echinopla lineata* Mayr, 1862: 689 (w.). Type locality: INDONESIA (Java).

**Distribution. *Southern***: Pattani (Yaring).

**Remarks.** New record.

**Material examined.** Pattani Prov, Yaring Dist, mangrove forest, 19.IV.2002, C. Bourmas leg. (THNHM).

***Echinopla madli* Zettel & Laciny, 2015**

*Echinopla madli* Zettel & Laciny, 2015: 103, figs 1–4.

**Distribution. *Southern***: Surat Thani (Khlong Saeng WS), Ranong (Khlong Na Kha WS), Satun (Thale Ban NP*), Narathiwat (Hala–Bala WS).

**References.**Zettel and Laciny (2015), Tanansathaporn et al. (2018).

***Echinopla melanarctos* Smith, 1857**

*Echinopla melanarctos* Smith, 1857: 79, pl. 1, figs 25–29.

**Distribution. *Southern***: Pattani (Nong Chik), Yala (Betong), Narathiwat (Hala–Bala WS).

**References.**Bingham (1906), Jaitrong and Nabhitabhata (2005), Tanansathaporn et al. (2018).

***Echinopla pallipes* Smith, 1857**

*Echinopla pallipes* Smith, 1857: 80.

**Distribution. *Southern***: Narathiwat (Hala–Bala WS).

**References.**Jaitrong and Nabhitabhata (2005), Tanansathaporn et al. (2018).

***Echinopla striata* Smith, 1857**

Figs 32, 33

*Echinopla striata* Smith, 1857: 80.

**Distribution. *Eastern***: Chanthaburi (Pheao NP). ***Southern***: Ranong (Khlong Na Kha WS), Nakhon Si Thamarat (Khao Nan NP, Tapi watershed), Patthalung (Tamot waterfall), Surat Thani (Tai Rom Yen NP, Khlong Yan WS), Trang (Thung Khai BG), Narathiwat (Hala–Bala WS).

**Remarks.** New record.

**Material examined.** Chanthaburi, Namtok Trok Nong, tropical rain forest, 23.XI.2003, D. Wiwatwitaya leg. (THNHM); Nakhon Si Thamarat, Khao Nan NP, Yod Nam waterfall, dead twig on tree, Sk. Yamane leg., TH08–SKY–193 (SKYC); Nakhon Si Thamarat, Phipun, Tapi watershed, 13.X.2011, Sk. Yamane leg. (SKYC); Phat Thalun [Patthalung Prov], Tamot waterfall, dead twig on tree, 27.IX.2008, Sk. Yamane, TH08–SKY–154; Nakhon Si Thammarat Prov, Khao Nan NP, 12.XII.2007, W. Jaitrong leg., WJT07–TH–1909 (THNHM); Surat Thani Prov, Tai Rom Yen NP, 400–500 m alt., dead twig, Sk. Yamane leg., TH11–SKY–41X (SKYC); same locality, 11.X.2011, W. Jaitrong leg., TH11–WJT–39 (THNHM); same locality and collector, 12.VII.2009, WJT09–TH–2060 (THNHM); Surat Thani Prov, Vibhavadi Dist, Khlong Yan WS, 30.XII.2001, W. Jaitrong leg. (THNHM); Surat Thani Prov, Khlong Saeng WS, 100–300 m alc., evergreen forest, 1994, L. Label leg. (PSU); Trang Prov, Yan Takhao Dist, Thung Khai B.G., 13.XI.2017, W. Jaitrong leg. (THNHM); Songkhla Prov, Ton Nga Chang WS, 13.IX.2004, evergreen forest (EF), W. Jaitrong leg. (THNHM); Narathiwat Prov, Wang Dist, Hala–Bala WS, tropical rain forest, 6.VI.2001, S. Hasin leg. (THNHM); same locality and collector, 30.VI.2018, WJT300618–2 (THNHM).

**Figures 32–41.**
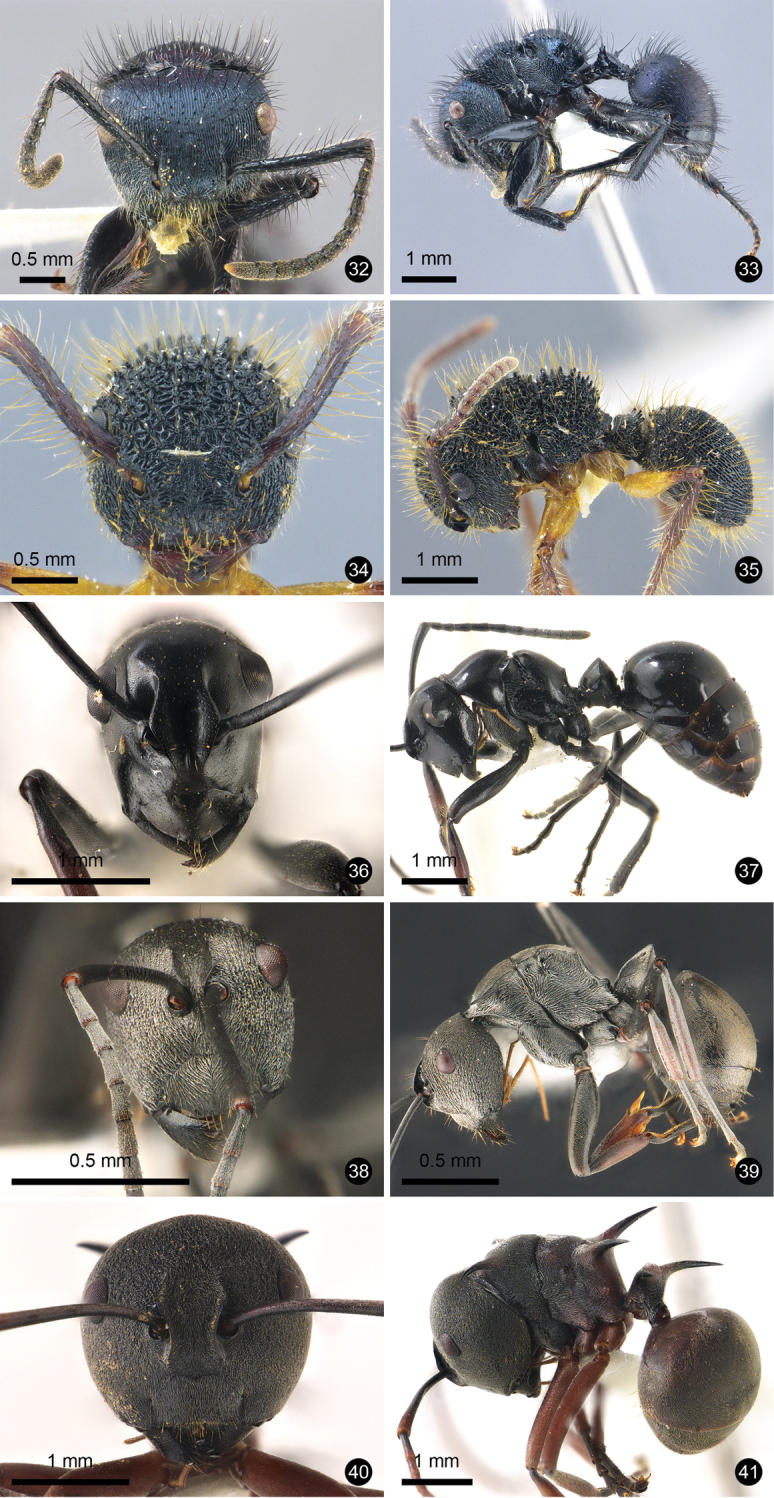
Ant species new to Thailand. **32, 33***Echinopla striata***34, 35***Echinopla tritschleri***36, 37***Polyrhachis boltoni***38, 39***Polyrhachis carbonaria***40, 41***Polyrhachis cephalotes*.

***Echinopla tritschleri* Forel, 1901**

Figs 34, 35

*Echinopla tritschleri* Forel, 1901b: 74 (w.). Type locality: INDONESIA (Sumatra).

**Distribution. *Southern***: Nakhon Si Thammarat (Khao Luang).

**Remarks.** New record.

**Material examined.** Nakhon Si Thammarat Prov, Khao Luang, Noppitam, 20.V.2008, W. Jaitrong leg., TH03–WJT–321 (THNHM).

***Echinopla tunkuabduljalilii* Laciny, Zettel, Maryati & Noor–Izwan, 2019**

*Echinopla tunkuabduljalilii*Laciny et al., 2019: 251, figs 1–5. Type locality: MALAYSIA.

**Distribution. *Southern***: Nakhon Si Thammarat (Noppitum), Phang–nga (Ton Pariwat waterfall, Si Phang–nga), Yala (Betong).

**References.**Laciny et al. (2019).

***Euprenolepis procera* (Emery, 1900)**

*Prenolepis procera* Emery, 1900b: 699.

**Distribution. *Southern***: Nakhon Si Thammarat (Khao Nan NP), Trang (Khao Chong BG), Phatthalung (Khao Pu–Khao Ya NP), Narathiwat (Hala–Bala WS).

**References.**Jaitrong and Nabhitabhata (2005).

***Euprenolepis wittei* LaPolla, 2009**

*Euprenolepis wittei* LaPolla, 2009: 20, figs 12A–D, 13A–I.

**Distribution. *Southern***: Nakhon Si Thammarat (Khao Nan NP).

**References.**LaPolla (2009).

***Lepisiota watsonii* (Forel, 1894)**

Plagiolepis rothneyi r. watsonii Forel, 1894.

**Distribution. Distribution.** Bangkok (unknown locality).

**References.**Forel (1894).

***Myrmoteras binghamii* Forel, 1893**

*Myrmoteras binghamii* Forel, 1893a: 608.

**Distribution. *Northern***: Chiang Mai (Pa Miang Village, Mae Rim, Chiang Dao WS, Doi Suthep–Pui NP), Nan (Doi Phu Kha NP). ***Western***: Tak (Thung Yai Naresuan East WS, Umphang WS), Kanchanaburi (Thong Pha Phum).

**References.**Agosti (1992), Jaitrong and Nabhitabhata (2005), Bui et al. (2013), Jaitrong (2011), Onishi et al. (2016).

***Myrmoteras concolor* Bui, Eguchi & Yamane, 2013**

*Myrmoteras concolor* Bui, Eguchi & Yamane, 2013: 550, figs 1C, 4.

**Distribution. *Western***: Tak (Umphang WS). ***Northeastern***: Loei (Phu Luang WS), Nakhon Ratchasima (Khao Yai NP). ***Eastern***: Chachoengsao (Khao Ang Reu Nai WS), Chanthaburi (Khao Soi Dao WS*), Trat (Ko Kut).

**References.**Bui et al. (2013).

***Myrmoteras cuneonodus* Xu, 1998**

*Myrmoteras cuneonodus* Xu, 1998: 125.

**Distribution. *Western***: Tak (Thung Yai Naresuan East WS), Kanchanaburi (Thong Pha Phum NP) ***Northeastern***: Nakhon Ratchasima (Sakaerat, Khao Yai NP). ***Central***: Uthai Thani (Huai Kha Khaeng WS). ***Eastern***: Chanthaburi (Khao Soi Dao WS, Khao Khitchakut NP).

**References.**Bui et al. (2013).

***Myrmoteras jaitrongi* Bui, Eguchi & Yamane, 2013**

*Myrmoteras jaitrongi* Bui, Eguchi & Yamane, 2013: 551, figs 1B, 5.

**Distribution. *Southern***: Narathiwat (Hala–Bala WS*).

**References.**Bui et al. (2013).

***Myrmoteras opalinum* Bui, Eguchi & Yamane, 2013**

*Myrmoteras opalinum* Bui, Eguchi & Yamane, 2013: 553, fig. 7.

**Distribution. *Central***: Uthai Thani (Huai Kha Khaeng WS). ***Southern***: Krabi (Ko Lanta NP), Surat Thani (Tai Rom Yen NP*), Nakhon Si Thammarat (Khao Nan NP).

**References.**Bui et al. (2013), Jaitrong and Jeenthong (2014a).

***Myrmoteras tomimasai* Bui, Eguchi & Yamane, 2013**

*Myrmoteras tomimasai* Bui, Eguchi & Yamane, 2013: 556, fig. 8.

**Distribution. *Western***: Tak (Thung Yai Naresuan East WS). ***Northeastern***: Loei (Phu Luang WS).

**References.**Bui et al. (2013).

***Oecophylla smaragdina* (Fabricius, 1775)**

*Formica smaragdina* Fabricius, 1775: 828.

**Distribution. *Northern***: Chiang Rai (Doi Tung), Chiang Mai (Pa Miang Village, Doi Ang Khang, Doi Luang Chiang Dao, Chiang Dao WS, Mae Taeng, Doi Suthep–Pui NP, Chiang Mai University Campus, Doi Inthanon NP, Mae Chaem, Omkoi), Phayao (Mae Ka), Lampang (Haui Tak, Tham Pha Thai NP), Lamphun (Mae Li Forest Plantation), Phrae (Wang Chin Plantation, Maejo Phrae Campus), Nan (Doi Phu Kha NP, Nakhon Nan Forest Plantation). ***Western***: Tak (Umphang WS, Thung Yai Naresuan East WS, Lansang NP, Doi Mu Sur Market), Kanchanaburi (Thong Pha Phum), Prachuap Khiri Khan (Kui Buri). ***Northeastern***: Kalasin (Phu SithanWS), Chaiyaphum (Phu Khiao WS, Bam Netnarong), Loei (Phu Luang WS), Nakhon Ratchasima (Sakaerat, Khao Yai NP, Buer Yai), Ubon Ratchathani (Pha Taem NP). ***Central***: Uthai Thani (Huai Kha Khaeng WS), Saraburi (Phukae BG, Namtok Sam Lan NP), Bangkok (Bang Khen, Chatuchak), Pathum Thani (Khlong Luang), Phetchaburi (Kaeng Krachan NP), Samut Prakan (Bang Krachao), Samut Songkhram (Mueang Samut Songkhram). ***Eastern***: Chachoengsao (Khao Ang Reu Nai WS), Chon Buri (Si Racha, Khao Kheow, Ko Samaesarn), Chanthaburi (Khao Soi Dao WS, Pheao NP), Trat (Ko Kut). ***Southern***: Chumphon (Krom Luang Chumphon NP), Ranong (Khlong Na Kha WS), Surat Thani (Tai Rom Yen NP, Khlong Yan WS, Khlong Saeng WS), Nakhon Si Thammarat (Khao Nan NP, Khao Luang NP, Krung Ching Waterfall), Krabi (Ko Lanta), Trang (Khao Chong BG, Thung Khai BG, Palian), Phatthalung (Khao Pu–Khao Ya NP, Khao Bantad WS), Satun (Tarutao NP), Songkhla (Khao Kho Hong, Khao Nam Khang NP, Ton Nga Chang WS), Yala, Pattani (Nong Chik), Narathiwat (Hala–Bala WS, Toh Daeng).

**References.**Tongjerm et al. (2003), Jaitrong and Nabhitabhata (2005), Azuma et al. (2006), Jaitrong (2011), Jaitrong and Jeenthong (2014a), Suwannaphak et al. (2016), Onishi et al. (2016).

***Paraparatrechina opaca* (Emery, 1887)**

Prenolepis clandestina var. opaca Emery, 1887b: 243. Combination in *Paraparatrechina*: LaPolla et al. 2010: 128.

**Distribution. *Northern***: Chiang Rai (Doi Tung), Chiang Mai (Khun Chang Khian, Doi Ang Khang, Doi Luang Chiang Dao, Chiang Dao WS, Mae Taeng, Doi Suthep–Pui NP, Chiang Mai University Campus, Doi Inthanon NP, Mae Chaem), Phayao (Mae Ka), Lampang (Haui Tak, Tham Pha Thai NP), Phrae (Wang Chin Plantation), Nan (Doi Phu Kha NP). ***Western***: Tak (Umphang WS, Thung Yai Naresuan East WS, Lansang NP), Kanchanaburi (Thong Pha Phum), Phetchaburi (Kaeng Krachan NP). ***Northeastern***: Kalasin (Phu SithanWS), Chaiyaphum (Phu Khiao WS), Loei (Phu Luang WS), Nakhon Ratchasima (Sakaerat, Khao Yai NP, Buer Yai), Ubon Ratchathani (Pha Taem NP). ***Central***: Saraburi (Phukae BG), Bangkok (Bang Khen, Chatuchak). ***Eastern***: Chachoengsao (Khao Ang Reu Nai WS), Chon Buri (Si Racha, Khao Kheow), Chanthaburi (Khao Soi Dao WS, Pheao NP), Trat (Ko Kut). ***Southern***: Chumphon (Krom Luang Chumphon NP), Ranong (Khlong Na Kha WS), Surat Thani (Tai Rom Yen NP, Khlong Yan WS, Khlong Saeng WS), Nakhon Si Thammarat (Khao Nan NP, Khao Luang NP, Krung Ching Waterfall), Trang (Khao Chong BG, Thung Khai BG, Palian), Phatthalung (Khao Po Khao Ya NP, Khao Bantad WS), Satun (Tarutao NP), Songkhla (Khao Kho Hong, Khao Nam Khang NP), Narathiwat (Hala–Bala WS).

**References.**Jaitrong and Nabhitabhata (2005, cited as *Paratrechina opaca* Emery, 1887), Onishi et al. (2016).

***Paratrechina longicornis* (Latreille, 1802)**

*Formica longicornis* Latreille, 1802: 113.

**Distribution. *Northern***: Chiang Rai (Doi Tung), Chiang Mai (Doi Ang Khang, Doi Luang Chiang Dao, Chiang Dao WS, Mae Taeng, Doi Suthep–Pui NP, Chiang Mai University Campus, Doi Inthanon NP, Mae Chaem), Phayao (Mae Ka), Lampang (Haui Tak, Tham Pha Thai NP), Phrae (Wang Chin Plantation), Nan (Doi Phu Kha NP). ***Western***: Tak (Umphang WS, Thung Yai Naresuan East WS, Lansang NP), Kanchanaburi (Thong Pha Phum), Phetchaburi (Kaeng Krachan NP), Prachuap Khiri Khan (Kui Buri). ***Northeastern***: Kalasin (Phu SithanWS), Chaiyaphum (Phu Khiao WS), Loei (Phu Luang WS), Nakhon Ratchasima (Sakaerat, Khao Yai NP, Buer Yai), Ubon Ratchathani (Pha Taem NP). ***Central***: Uthai Thani (Huai Kha Khaeng WS), Saraburi (Phukae BG, Namtok Sam Lan NP), Bangkok (Bang Khen, Chatuchak), Pathum Thani (Khlong Luang), Samut Prakan (Bang Krachao). ***Eastern***: Chachoengsao (Khao Ang Reu Nai WS), Chon Buri (Si Racha, Khao Kheow, Ko Samaesarn), Chanthaburi (Khao Soi Dao WS, Pheao NP), Trat (Ko Kut, Ko Chang, Trat Agoforestry Research Station). ***Southern***: Chumphon (Krom Luang Chumphon NP), Ranong (Khlong Na Kha WS), Surat Thani (Tai Rom Yen NP, Khlong Yan WS, Khlong Saeng WS), Nakhon Si Thammarat (Khao Nan NP, Khao Luang NP, Krung Ching Waterfall), Krabi (Ko Lanta), Trang (Khao Chong BG, Thung Khai BG, Palian), Phatthalung (Khao Pu–Khao Ya NP, Khao Bantad WS), Satun (Tarutao NP), Songkhla (Khao Kho Hong, Khao Nam Khang NP), Narathiwat (Hala–Bala WS).

**References.**Jaitrong and Nabhitabhata (2005), Jaitrong (2011), Jaitrong and Jeenthong (2014a).

***Polyrhachis abdominalis* Smith, 1858**

*Polyrhachis abdominalis* Smith, 1858: 63.

**Distribution. *Northern***: Chiang Mai (Omkoi), Phrae (Wang Chin). ***Western***: Tak (Thung Yai Naresuan East WS, Umphang WS). ***Eastern***: Chachoengsao (Khao Ang Reu Nai WS), Chanthaburi (Khao Soi Dao WS, Namtok Phlio NP, Pheao NP). ***Central***: Uthai Thani (Huai Kha Khaeng WS). ***Southern***: Chumphon (Krom Luang Chumphon WS), Surat Thani (Tai Rom Yen NP), Narathiwat (Hala–Bala WS).

**References.**Jaitrong and Nabhitabhata (2005).

***Polyrhachis alatisquamis* Forel, 1893**

Polyrhachis pubescens var. alatisquamis Forel, 1893a: 17. Revised to species by Kohout (2013).

**Distribution.** Cited as Siam, on further data. ***Western***: Tak (Umphang WS, Thung Yai WS, Mae Sot).

**References.**Kohout (2013).

***Polyrhachis arachne* Emery, 1896**

*Polyrhachis arachne* Emery, 1896: 249.

**Distribution. *Northern***: Chiang Mai (Omkoi). ***Western***: Kanchanaburi (Pha Tad Watershed). ***Eastern***: Rayong (Khao Ang Reu Nai WS). ***Southern***: Songkhla (Ton Nga Chang WS).

**References.**Emery (1896, 1898, cited as *Polyrhachis uncinata* André, 1896, junior synonym of *P. arachne*), André (1896).

***Polyrhachis arcuata* (Le Guillou, 1842)**

*Formica arcuata* Le Guillou, 1842: 315.

**Distribution. *Central***: Bangkok (Bang Khen), Pathum Thani (Khlong Luang). ***Southern***: Narathiwat (Hala–Bala WS).

**References.**Jaitrong and Nabhitabhata (2005).

***Polyrhachis armata* (Le Guillou, 1842)**

*Formica armata* Le Guillou, 1842: 313.

**Distribution. *Northern***: Chiang Rai (Doi Tung), Chiang Mai (Pa Miang Village, Doi Ang Khang, Doi Luang Chiang Dao, Chiang Dao WS, Mae Taeng, Doi Suthep–Pui NP, Chiang Mai University Campus, Doi Inthanon NP, Mae Chaem, Omkoi), Phayao (Mae Ka), Lamphun (Mae Li Forest Plantation), Lampang (Haui Tak, Tham Pha Thai NP), Phrae (Wang Chin Plantation), Nan (Doi Phu Kha NP, Nakhon Nan Forest Plantation) ***Western***: Tak (Umphang WS, Thung Yai Naresuan East WS, Lansang NP), Kanchanaburi (Khuean Srinagarindra NP, Sai Yok NP, Thong Pha Phum), Phetchaburi (Kaeng Krachan NP). ***Northeastern***: Kalasin (Phu SithanWS), Chaiyaphum (Phu Khiao WS), Loei (Phu Luang WS), Nakhon Ratchasima (Sakaerat, Khao Yai NP, Buer Yai), Ubon Ratchathani (Pha Taem NP). ***Central***: Uthai Thani (Huai Kha Khaeng WS), Saraburi (Phukae BG, Namtok Sam Lan NP), Bangkok (Bang Khen, Chatuchak), Pathum Thani (Khlong Luang). ***Eastern***: Chachoengsao (Khao Ang Reu Nai WS), Chon Buri (Si Racha, Khao Kheow), Chanthaburi (Khao Soi Dao WS, Namtok Phlio NP, Pheao NP), Trat (Ko Kut, Trat Agoforestry Research Station). ***Southern***: Chumphon (Krom Luang Chumphon NP), Ranong (Khlong Na Kha WS), Surat Thani (Tai Rom Yen NP, Khlong Yan WS), Nakhon Si Thammarat (Tapi Watershed Research Station, Khao Nan NP, Khao Luang NP, Krung Ching Waterfall), Phuket (Thalang), Krabi (Ko Lanta), Trang (Khao Chong BG, Thung Khai BG, Palian), Phatthalung (Khao Pu–Khao Ya NP, Khao Bantad WS), Satun (Tarutao NP), Songkhla (Khao Kho Hong, Khao Nam Khang NP), Narathiwat (Hala–Bala WS).

**References.**Jaitrong and Nabhitabhata (2005), Suwannaphak et al. (2016); Onishi et al. (2016).

***Polyrhachis bicolor* Smith, 1858**

*Polyrhachis bicolor* Smith, 1858: 65.

**Distribution. *Western***: Kanchanaburi (Pha Tad Watershed). ***Eastern***: Chachoengsao (Khao Ang Reu Nai WS), Chanthaburi (Khao Soi Dao WS, Khao Ang Reu Nai WS). ***Southern***: Krabi (Ko Lanta), Trang (Palian), Songkhla (Ton Nga Chang WS), Narathiwat (Hala–Bala WS, Toh Daeng), Nakhon Si Thammarat (Khao Nan NP).

**References.**Jaitrong and Nabhitabhata (2005), Tongjerm et al. (2003), Jaitrong and Jeenthong (2014a).

***Polyrhachis bihamata* (Drury, 1773)**

*Formica bihamata* Drury, 1773: 73, pl. 38, figs 7, 8.

**Distribution. *Northern***: Chiang Mai (Omkoi, Chiang Dao WS, Doi Suthep–Pui NP), Lampang (Tham Pha Thai NP). ***Western***: Tak (Thung Yai Naresuan East WS, Umphang WS), Kanchanaburi (Thong Pha Phum). ***Northeastern***: Chaiyaphum (Phu Khiao WS), Loei (Phu Luang WS), Nakhon Ratchasima (Sakaerat, Khao Yai NP). ***Central***: Uthai Thani (Huai Kha Khaeng WS). ***Eastern***: Sa Kaeo (Pang Sida NP), Chachoengsao (Khao Ang Reu Nai WS), Chanthaburi (Khao Soi Dao WS), Trat (Ko Kut). ***Southern***: Songkhla (Ton Nga Chang WS), Yala, Pattani (Nong Chik), Narathiwat (Hala–Bala WS), Surat Thani (Tai Rom Yen NP).

**References.**Bingham (1906), Jaitrong and Nabhitabhata (2005), Kohout (2014).

***Polyrhachis boltoni* Dorow & Kohout, 1995**

Figs 36, 37

*Polyrhachis boltoni* Dorow & Kohout, 1995: 96, fig. 1.

**Distribution. *Southern***: Nakhon Si Thammarat (Krung Ching Waterfall), Songkhla (Ton Nga Chang WS), Narathiwat (Hala–Bala WS).

**Remarks.** New record.

**Material examined.** S Thailand, Nakhon Si Thammarat Prov, Krung Ching Waterfall, 14.IV.2005, N. Noon–anant leg. (THNHM); S Thailand, Songkhla Prov, Ton Nga Chang WS, 24.III.2004, N. Noon–anant leg. (THNHM); S Thailand, Narathiwat Prov, Hala–Bala WS, 20.X.2003, Y. Sittimul leg. (AMK).

***Polyrhachis calypso* Forel, 1911**

Polyrhachis spinosa subsp. calypso Forel, 1911b: 394.

**Distribution. *Northeastern***: Sakon Nakhon (Akat Aumnuai). ***Western***: Kanchanaburi (Thong Pha Phum NP). ***Southern***: Satun (Tarutao NP), Songkhla (Ton Nga Chang WS), Narathiwat (Hala–Bala WS), Patthalung (Tamot waterfall), Nakhon Si Thammarat (Khao Nan NP)

**References.**Jaitrong and Nabhitabhata (2005).

***Polyrhachis carbonaria* Smith, 1857**

Figs 38, 39

*Polyrhachis* (sic) *carbonarius* Smith, 1857.

**Distribution. *Southern***: Songkhla (Ton Nga Chang NP).

**Remarks.** New record.

**Material examined.** S Thailand, Songkhla Prov, Ton Nga Chang NP, 24.vii.1997, Sk. Yamane (SKYC).

***Polyrhachis cephalotes* Emery, 1893**

Figs 40, 41

*Polyrhachis cephalotes* Emery, 1893b: 199, pl. 8, fig. 6.

**Distribution. *Southern***: Ranong (Khlong Na Kha WS).

**Remarks.** New record.

**Material examined.** S Thailand, Ranong Prov, Khlong Na Kha WS, 12.VII.2009, W. Jaitrong leg. (THNHM).

***Polyrhachis chalybea* Smith, 1857**

Figs 42, 43

*Polyrhachis chalybea* Smith, 1857: 61.

**Distribution. *Northern***: Chiang Mai (Doi Chiang Dao).

**Remarks.** New record.

**Material examined.** Chiang Mai Prov, Doi Chiang Dao, 3.IV.2005, Sk. Yamane leg. (SKYC).

**Figures 42–51.**
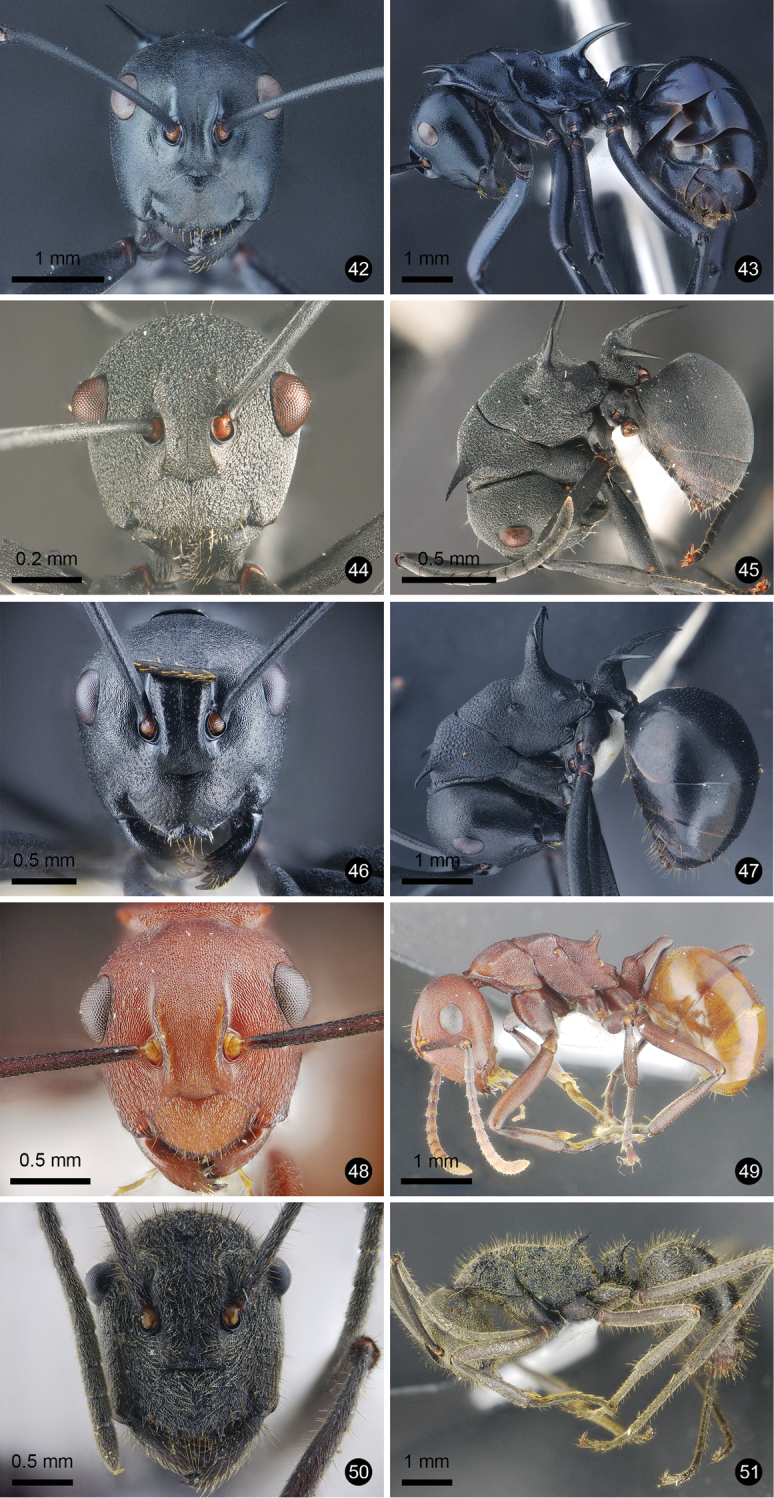
Ant species new to Thailand. **42, 43***Polyrhachis chalybea***44, 45***Polyrhachis fortis***46, 47***Polyrhachis hodgsoni***48, 49***Polyrhachis javanica***50, 51***Polyrhachis lama*.

***Polyrhachis craddocki* Bingham, 1903**

*Polyrhachis craddocki* Bingham, 1903: 403, fig. 138.

**Distribution. *Northern***: Chiang Mai (Omkoi).

**References.**Jaitrong et al. (2018b).

***Polyrhachis cryptoceroides* Emery, 1887**

*Polyrhachis cryptoceroides* Emery, 1887a: 228, pl. 3, fig. 14.

**Distribution. *Western***: Prachuap Khiri Khan (Sai Kru Waterfall). ***Eastern***: Chanthaburi (Khao Soi Dao WS).

**References.**Kohout (2006).

***Polyrhachis dives* Smith, 1857**

*Polyrhachis dives* Smith, 1857: 64.

**Distribution. *Northern***: Chiang Mai (Pa Miang Village, Doi Ang Khang, Mae Yod, Doi Pha Hom Pok NP), Nan (Doi Phu Kha NP, Nakhon Nan Forest Plantation), Lampang (Ngao, Mae Chang Forest Plantation), Phrae (Wang Chin Forest Plantation), Lamphun (Mae Li Forest Plantation). ***Western***: Tak (Thung Yai Naresuan East WS, Umphang WS), Kanchanaburi (Thong Pha Phum NP), Ratchaburi (unknown locality), Prachuap Khiri Khan (Kui Buri). ***Northeastern***: Loei (Phu Luang WS), Chaiyaphum (Phu Khiao WS), Nakhon Ratchasima (Khao Yai NP, Sakaerat), Kalasin (Phu Sithan WS), Ubon Rachatahi (Kong Chiam). ***Eastern***: Chanthaburi (Khao Khitchakut NP, Khao Soi Dao WS, Khlung, Pheao NP), Chon Buri (Kasetsart Si Racha Campus), Trat (Ko Kut). ***Central***: Bangkok (Bang Khen), Pathum Thani (Khlong Luang), Nakhon Nayok (Nang Rong Waterfall), Saraburi (Phukae BG, Namtok Sam Lan NP), Samut Songkhram (Mueang Samut Songkhram). ***Southern***: Chumphon (Krom Luang Chumphon NP), Ranong (Ngao, Khlong Na Kha WS), Surat Thani (Tai Rom Yen NP, Khlong Yan WS), Nakhon Si Thammarat (Khao Nan NP, Khao Luang NP, Krung Ching Waterfall), Phuket (Thalang), Trang (Khao Chong BG, Thung Khai BG, Palian), Phatthalung (Khao Pu–Khao Ya NP, Khao Bantad WS), Songkhla (Khao Kho Hong, Khao Nam Khang NP), Narathiwat (Hala–Bala WS).

**References.**Jaitrong and Nabhitabhata (2005), Suwannaphak et al. (2016), Onishi et al. (2016).

***Polyrhachis dolomedes* Smith, 1863**

*Polyrhachis dolomedes* Smith, 1863: 14.

**Distribution. *Eastern***: Chachoengsao (Khao Ang Reu Nai).

**References.**Jaitrong and Nabhitabhata (2005).

***Polyrhachis flavicornis* Smith, 1857**

*Polyrhachis flavicornis* Smith, 1857: 63.

**Distribution. *Northern***: Chiang Mai (Doi Suthep). ***Western***: Tak (Thung Yai Naresuan East WS). ***Central***: Uthai Thai (Huai Kha Khaeng WS). ***Eastern***: Chachoengsao (Khao Ang Rue Nai WS), Chanthaburi (Khao Soi Dao WS). ***Southern***: Songkhla (Ton Nga Chang WS).

**References.**Jaitrong and Nabhitabhata (2005).

***Polyrhachis flavoflagellata* Karavaiev, 1927**

Polyrhachis (Myrmhopla) flavoflagellata Karavaiev, 1927: 35, fig. 16.

**Distribution. *Southern***: Songkhla (Ton Nga Chang WS).

**References.** Watanasit & Noon–anant (2005).

***Polyrhachis fortis* Emery, 1893**

Figs 44, 45

*Polyrhachis fortis* Emery, 1893b: 228, pl. 8, fig. 5.

**Distribution.** Northern: Chiang Mai (Doi Pui).

**Remarks.** New record.

**Material examined.** Chiang Mai Prov, Doi Pui, 23.xii.1997, F. Yamane leg. (identified by R. Kohout; SKYC).

***Polyrhachis furcata* Smith, 1858**

*Polyrhachis furcata* Smith, 1858: 64, pl. 4, fig. 20.

**Distribution. *Northern***: Phrae (Wang Chin). Western: Tak (Thung Yai Naresuan East WS, Umphang WS), Kanchanaburi (Thong Pha Phum), Phetchaburi (Kaeng Krachan NP). ***Northeastern***: Chaiyaphum (Phu Khiao WS), Nakhon Ratchasima (Khao Yai NP, Sakaerat). ***Eastern***: Chachoengsao (Khao Ang Reu Nai WS), Chanthaburi (Pheao NP, Khao Soi Dao, Namtok Phlio NP). ***Southern***: Chumphon (Krom Luang Chumphon WS), Surat Thani (Tai Rom Yen NP), Phatthalung (Khao Pappha), Ranong (Khlong Na Kha WS), Nakhon Si Thammarat (Khao Luang NP, Khao Nan NP), Trang (Khao Chong BG, Ton Tae Waterfall), Narathiwat (Hala–Bala WS), Songkhla (Hat Yai).

**References.**Jaitrong and Nabhitabhata (2005).

***Polyrhachis halidayi* Emery, 1889**

*Polyrhachis halidayi* Emery, 1889b: 517.

**Distribution. *Northern***: Chiang Mai (Khun Chang Khian, Doi Suthep–Pui NP, Doi Ang Khang, Mae Chaem, Omkoi, Doi Inthanon NP), Phrae (Wang Chin). ***Western***: Tak (Thung Yai Naresuan East WS, Umphang WS), Kanchanaburi (Thong Pha Phum NP, Maeklong Watershed), Prachuap Khiri Khan (Kaeng Krachan NP). ***Northeastern***: Loei (Phu Luang WS), Chaiyaphum (Phu Khiao WS), Nakhon Ratchasima (Khao Yai NP, Sakaerat). ***Central***: Phitsalulok (Thung Salaeng Luang NP), Uthai Thani (Huai Kha Khaeng WS), Pathum Thani (Khlong Luang). ***Eastern***: Chanthaburi (Khao Soi Dao WS), Chachoengsao (Khao Ang Reu Nai WS). ***Southern***: Ranong (Khlong Na Kha WS), Trang (Khao Chong BG).

**References.**Jaitrong and Nabhitabhata (2005), Jaitrong et al. (2007), Sakchoowong et al. (2008), Sakchoowong et al. (2009), Suwannaphak et al. (2016), Onishi et al. (2016).

***Polyrhachis hauxwelli* Bingham, 1903**

*Polyrhachis hauxwelli* Bingham, 1903: 394, fig. 133.

**Distribution. *Southern***: Trang (Palian).

**References.**Jaitrong and Nabhitabhata (2005).

***Polyrhachis hector* Smith, 1857**

*Polyrhachis hector* Smith, 1857: 62.

**Distribution. *Eastern***: Chachoengsao (Khao Ang Reu Nai WS). ***Southern***: Trang (Khao Chong BG).

**References.**Antweb (2019).

***Polyrhachis hemiopticoides* Mukerjee, 1930**

*Polyrhachis hemiopticoides* Mukerjee, 1930: 161, fig. 5.

**Distribution. *Western***: Tak (Umphang WS, Thung Yai Naresuan East WS). ***Southern***: Surat Thani (Khlong Saeng WS).

**References.**Kohout (2013).

***Polyrhachis hippomanes* Smith, 1861**

*Polyrhachis hippomanes* Smith, 1861: 43, pl. 1, fig. 21.

**Distribution. *Northeastern***: Kalasin (Phu Sithan WS), Chaiyaphum (Phu Khiao WS), Nakhon Ratchasima (Khao Yai NP, Sakaerat). ***Eastern***: Chachoengsao (Khao Ang Reu Nai WS), Chanthaburi (Khao Soi Dao WS, Phaeo NP). ***Southern***: Songkhla (Khao Nam Khang NP), Narathiwat (Toh Daeng).

**References.**Jaitrong and Nabhitabhata (2005).

***Polyrhachis hodgsoni* Forel, 1902**

Figs 46, 47

*Polyrhachis hodgsoni* Forel, 1902a: 289.

**Distribution. *Western***: Phetchaburi (Kaeng Krachan NP).

**Remarks.** New record.

**Material examined.** Phetchaburi Prov, Kaeng Krachan NP, 29.VI.2014, Sk. Yamane and M. Maruyama (SKYC).

***Polyrhachis illaudata* Walker, 1859**

*Polyrhachis illaudatus* Walker, 1859: 373.

**Distribution. *Northern***: Chiang Mai (Omkoi, Chiang Dao WS, Doi Inthanon NP). ***Western***: Tak (Umphang WS, Thung Yai Naresuan East WS), Kanchanaburi (Pha Tad). ***Northeastern***: Nakhon Ratchasima (Khao Yai NP), Loei (Phu Luang WS). ***Eastern***: Chachoengsao (Khao Ang Reu nai WS), Chanthaburi (Pheao NP, Khao Soi Dao WS, Khlung, Namtok Phlio NP), Trat (Ko Kut). ***Southern***: Ranong (Khlong Na Kha WS), Surat Thani (Tai Rom Yen NP, Khlong Yan WS), Phuket (Thalang), Krabi (Ko Lanta), Phatthalung (Khao Pappha), Trang (Khao Chong BG), Satun (Tarutao NP), Songkhla (Hat Yai, Ton Nga Chang NP), Pattani (Nong Chik).

**References.**Bingham (1906, cited as *Polyrhachis mayri* Roger, 1862, junior synonym of *P. illaudata*), Jaitrong and Nabhitabhata (2005), Jaitrong et al. (2007), Jaitrong and Jeenthong (2014a).

***Polyrhachis illaudata intermedia* Forel, 1886**

Polyrhachis mayri subsp. intermedia Forel, 1886: 242.

**Distribution. *Northern***: Chiang Mai (Doi Suthep–Pui NP).

**References.**Antweb (2019).

***Polyrhachis inermis* Smith, 1858**

*Polyrhachis inermis* Smith, 1858: 68, pl. 4, figs 25, 26.

**Distribution. *Eastern***: Chachoengsao (Khao Ang Reu Nai WS). ***Southern***: Narathiwat (Hala–Bala WS).

**References.**Jaitrong and Nabhitabhata (2005).

***Polyrhachis javanica* Mayr, 1867**

Figs 48, 49

Polyrhachis thrinax var. javanica Mayr, 1867: 52.

**Distribution. *Eastern***: Chanthaburi (Khao Ang Reu Nai WS).

**Remarks.** New record.

**Material examined.** Chanthaburi Prov, Khao Ang Reu Nai WS, Lumchangwat stn., 22.VIII.2003, Sk. Yamane leg., TH03–SKY–81 (SKYC).

***Polyrhachis laevigata* Smith, 1857**

*Polyrhachis laevigatus*Smith. 1857: 62.

**Distribution. *Eastern***: Chanthaburi (Khao Soi Dao WS).

**References.**Antweb (2019).

***Polyrhachis laevissima* Smith, 1858**

*Polyrhachis laevissimus* Smith, 1858: 64, pl. 4, fig. 42.

**Distribution. *Northern***: Chiang Rai (Doi Tung), Chiang Mai (Pa Miang Village, Doi Ang Khang, Doi Luang Chiang Dao, Chiang Dao WS, Mae Taeng, Doi Suthep–Pui NP, Chiang Mai University Campus, Doi Inthanon NP, Mae Chaem), Phayao (Mae Ka), Lamphun (Mae Li Forest Plantation), Lampang (Haui Tak, Tham Pha Thai NP), Phrae (Wang Chin Plantation), Nan (Doi Phu Kha NP, Nakhon Nan Forest Plantation) ***Western***: Tak (Umphang WS, Thung Yai Naresuan East WS, Lansang NP, Taksin Maharat NP), Kanchanaburi (Thong Pha Phum NP, Pha Tad Watershed Management Station, Sai Yok NP), Ratchaburi (unknown locality), Phetchaburi (Kaeng Krachan NP), Prachuap Khiri Khan (Kui Buri). ***Northeastern***: Kalasin (Phu SithanWS), Chaiyaphum (Phu Khiao WS), Loei (Phu Luang WS), Nakhon Ratchasima (Sakaerat, Khao Yai NP, Buer Yai), Ubon Ratchathani (Pha Taem NP). ***Central***: Phitsanulok (Thung Salaeng Luang NP), Saraburi (Phukae BG, Namtok Sam Lan NP), Bangkok (Bang Khen, Chatuchak), Pathum Thani (Khlong Luang), Samut Prakan (Bang Krachao), Samut Songkhram (Mueang Samut Songkhram). ***Eastern***: Chachoengsao (Khao Ang Reu Nai WS), Chon Buri (Si Racha, Khao Kheow), Chanthaburi (Khao Soi Dao WS, Pheao NP, Khao Ang Reu Nai WS), Trat (Ko Kut, Trat Agroforestry Research Station). ***Southern***: Chumphon (Krom Luang Chumphon WS), Ranong (Khlong Na Kha WS), Surat Thani (Khlong Yan WS), Nakhon Si Thammarat (Khao Nan NP, Khao Luang NP, Krung Ching Waterfall), Krabi (Ko Lanta), Trang (Khao Chong BG, Thung Khai BG, Palian), Phatthalung (Khao Pu–Khao Ya NP, Khao Bantad WS), Satun (Tarutao NP), Songkhla (Khao Kho Hong, Khao Nam Khang NP), Yala, Pattani, Narathiwat (Hala–Bala WS Toh Daeng).

**References.**André (1896), Bingham (1906), Jaitrong and Nabhitabhata (2005), Jaitrong and Jeenthong (2014a), Suwannaphak et al. (2016).

***Polyrhachis lama* Kohout, 1994**

Figs 50, 51

*Polyrhachis lama* Kohout, 1994: 137, fig. 1.

**Distribution. *Northeastern***: Loei (Phu Luang WS), Nakhon Ratchasima (Sakaerat). ***Eastern***: Trat (Agroforestry Research Station).

**Remarks.** New record.

**Material examined.** NE Thailand, Loei Prov, Phu Luang Wildlife Research Station, 11.IV.2008, P. Kosonpanyapiwat leg. (THNHM); NE Thailand, Nakhon Ratchasima Prov, Sakaerat Environmental Research Station, Dry Dipterocarp Forest, 3.VIII.2008, S. Hasin leg. (THNHM); E Thailand, Trat Prov, Mueang Trat, Agroforestry Research Station, 28.I.2014, W. Jaitrong. (THNHM).

***Polyrhachis latona* Wheeler, 1909**

*Polyrhachis latona* Wheeler, 1909: 337.

**Distribution. *Northeastern***: Nakhon Ratchasima (Khao Yai NP).

**References.**Jaitrong and Nabhitabhata (2005).

***Polyrhachis mitrata* Menozzi, 1932**

Polyrhachis (Myrmhopla) mitrata Menozzi, 1932: 303.

**Distribution. *Southern***: Songkhla (Klong U–Tapao Basin).

**References.**Watanasit et al. (2007).

***Polyrhachis moesta* Emery, 1887**

Figs 52, 53

Polyrhachis hippomanes var. moesta Emery, 1887a: 237 (w.). Type locality: INDONESIA (Sumatra).

**Distribution. *Western***: Kanchanaburi (Pha Tad Watershed).

**Remarks.** New record.

**Material examined.** W Thailand, Kanchanaburi Prov, Pha Tad Watershed Conservation & Management, 28.XI.2003, Sk. Yamane leg. (THNHM).

**Figures 52–61.**
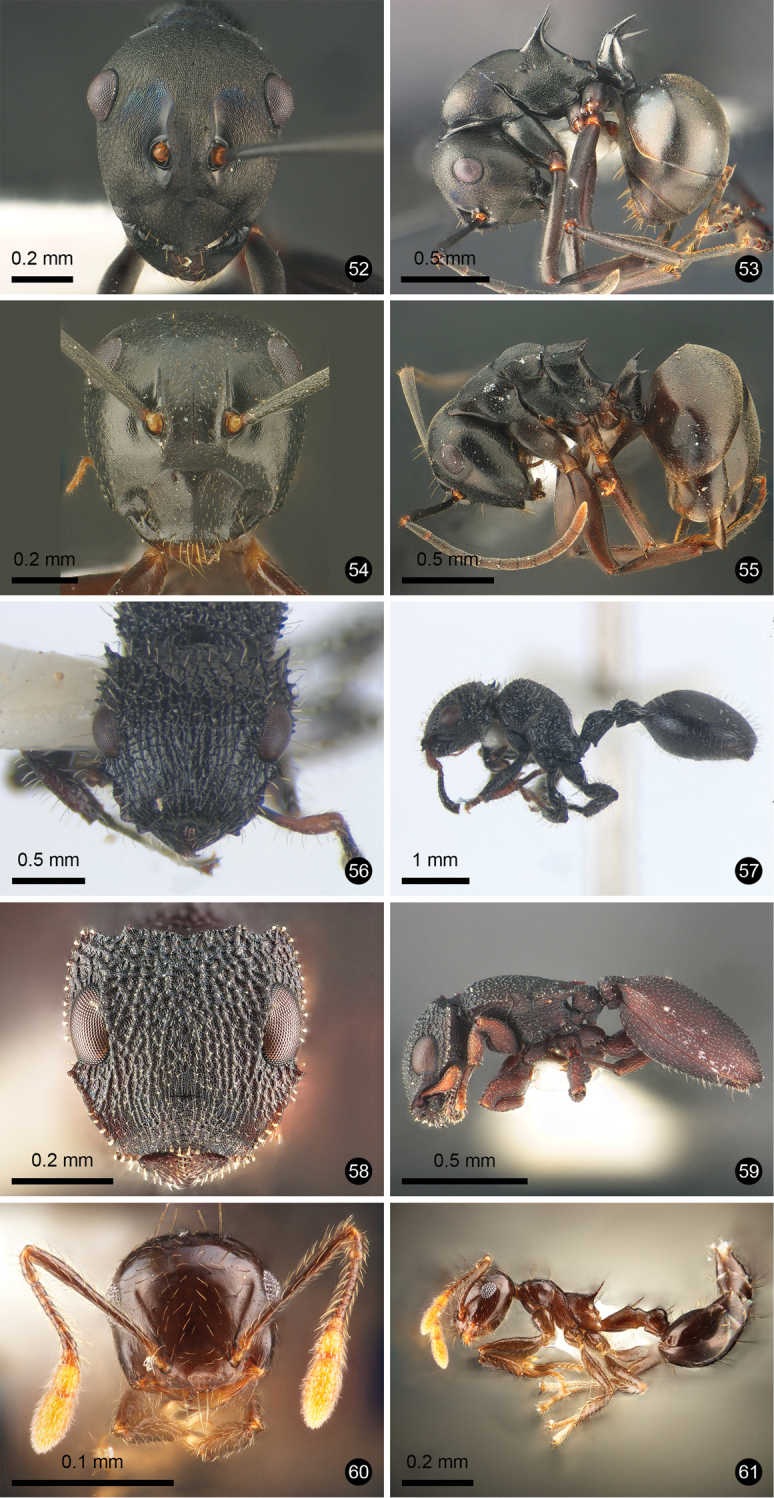
Ant species new to Thailand. **52, 53***Polyrhachis moesta***54, 55***Polyrhachis shixingensis***56, 57***Cataulacus muticus***58, 59***Cataulacus praetextus***60, 61***Crematogaster baduvi*.

***Polyrhachis muelleri* Forel, 1893**

*Polyrhachis muelleri* Forel, 1893a: 32.

**Distribution. *Western***: Tak (Umphang WS, Thung Yai Naresuan East WS). ***Eastern***: Chachoengsao (Khao Ang Reu Nai WS), Chanthaburi (Khao Soi Dao WS), Rayong (Khao Ang Reu Nai WS). ***Southern***: Songkhla (Ton Nga Chang WS).

**References.**Jaitrong and Nabhitabhata (2005), Jaitrong et al. (2007).

***Polyrhachis nigropilosa* Mayr, 1872**

*Polyrhachis nigropilosa* Mayr, 1872: 141.

**Distribution. *Southern***: Yala, Pattani, Nakhon Si Thammarat (Khao Nan NP).

**References.**Bingham (1906).

***Polyrhachis noonananti* Kohout, 2013**

*Polyrhachis noonananti* Kohout, 2013: 150, figs 12, 17, 18.

**Distribution. *Southern***: Surat Thani (Khlong Saeng WS*).

**References.**Kohout (2013).

***Polyrhachis ochracea* Karavaiev, 1927**

Polyrhachis (Myrmhopla) ochracea Karavaiev, 1927: 30.

**Distribution. *Southern***: Phatthalung (unknown locality).

**References.**Antweb (2019).

***Polyrhachis olybria* Forel, 1912**

*Polyrhachis olybrius* Forel, 1912c: 73.

**Distribution. *Western***: Tak (Umphang WS, Thung Yai Naresuan East WS). ***Northeastern***: Chaiyaphum (Phu Khiao WS), Nakhon Ratchasima (Sakaerat, Khao Yai NP). ***Eastern***: Chachoengsao (Khao Ang Reu Nai WS). ***Southern***: Chumphon (Krom Luang Chumphon WS), Ranong (Khlong Na Kha WS), Nakhon Si Thammarat (Khao Nan WS), Trang (Khao Chong BG), Narathiwat (Hala–Bala WS, Toh Daeng).

**References.**Jaitrong and Nabhitabhata (2005), Kohout (2013), Jaitrong et al. (2018b).

**Remarks.** All Thai specimens cited as *Polyrhachis bellicosa* Smith, 1859 in Jaitrong and Nabhitabhata (2005) should be reidentified as *P. olybria* (sensu Kohout, 2013).

***Polyrhachis phalerata* Menozzi, 1926**

Polyrhachis (Myrmatopa) phalerata Menozzi, 1926: 102.

**Distribution. *Northern***: Chiang Mai (Doi Pui). ***Western***: Kanchanaburi (Maeklong Watershed). ***Eastern***: Chachoengsao (Khao Ang Reu Nai WS), Chanthaburi (Khao Soi Dao). ***Southern***: Songkhla (Hat Yai).

**References.**Jaitrong and Nabhitabhata (2005).

***Polyrhachis piliventris* Smith, 1858**

*Polyrhachis piliventris* Smith, 1858: 60, pl. 4, fig. 24.

**Distribution. *Southern***: Pattani.

**References.**Bingham (1906).

***Polyrhachis proxima* Roger, 1863**

*Polyrhachis proxima* Roger, 1863: 155.

**Distribution. *Northern***: Chiang Rai (Doi Tung), Chiang Mai (Doi Ang Khang, Doi Luang Chiang Dao, Chiang Dao WS, Mae Taeng, Doi Suthep–Pui NP, Chiang Mai University Campus, Doi Inthanon NP, Mae Chaem, Omkoi), Phayao (Mae Ka), Lamphun (Mae Li Forest Plantation), Lampang (Haui Tak, Tham Pha Thai NP), Phrae (Wang Chin Plantation), Nan (Doi Phu Kha NP, Nakhon Nan Forest Plantation) ***Western***: Tak (Umphang WS, Thung Yai Naresuan East WS, Lansang NP, Taksin Maharat NP), Kanchanaburi (Thong Pha Phum NP, Pha Tad Watershed Management station), Phetchaburi (Kaeng Krachan NP). ***Northeastern***: Kalasin (Phu SithanWS), Chaiyaphum (Phu Khiao WS), Loei (Phu Luang WS), Nakhon Ratchasima (Sakaerat, Khao Yai NP, Buer Yai), Ubon Ratchathani (Pha Taem NP). ***Central***: Saraburi (Phukae BG), Bangkok (Bang Khen, Chatuchak), Pathum Thani (Khlong Luang). ***Eastern***: Chachoengsao (Khao Ang Reu Nai WS), Chon Buri (Si Racha, Khao Kheow), Chanthaburi (Khao Soi Dao WS, Pheao NP, Khao Ang Reu Nai WS), Trat (Ko Kut). ***Southern***: Chumphon (Krom Luang Chumphon NP), Ranong (Khlong Na Kha WS), Surat Thani (Tai Rom Yen NP, Khlong Yan WS), Nakhon Si Thammarat (Tapi Watershed Research Station, Khao Nan NP, Khao Luang NP, Krung Ching Waterfall), Krabi (Ko Lanta), Trang (Khao Chong BG, Thung Khai BG, Palian), Phatthalung (Khao Po Khao Ya NP, Khao Bantad WS), Songkhla (Khao Kho Hong, Khao Nam Khang NP), Narathiwat (Hala–Bala WS).

**References.**André (1896), Jaitrong and Nabhitabhata (2005), Jaitrong et al. (2007), Jaitrong and Jeenthong (2014a), Suwannaphak et al. (2016).

***Polyrhachis pubescens* Mayr, 1879**

*Polyrhachis pubescens* Mayr, 1879: 657.

**Distribution. *Southern***: Yala (Betong).

**References.**Bingham (1906), Kohout (2013).

***Polyrhachis rastellata* (Latreille, 1802)**

*Formica rastellata* Latreille, 1802: 130.

**Distribution. *Northern***: Chiang Mai (Doi Inthanon NP), Phrae (Wang Chin). ***Western***: Tak (Thung Yai Naresuan East WS, Umphang WS), Kanchanaburi (Thong Pha Phum NP). ***Southern***: Ranong (Khlong Na Kha WS).

**References.**Jaitrong and Nabhitabhata (2005).

***Polyrhachis rixosa* Smith, 1858**

*Polyrhachis rixosus* Smith, 1858: 68.

**Distribution. *Eastern***: Chachoengsao (Khao Ang Reu Nai WS).

**References.**Jaitrong and Nabhitabhata (2005).

***Polyrhachis rufipes* Smith, 1858**

*Polyrhachis rufipes* Smith, 1858: 66, pl. 4, fig. 28.

**Distribution. *Northern***: Chiang Mai (Omkoi). ***Western***: Prachuap Khiri Khan (Kui Buri NP). ***Eastern***: Chanthaburi (Pheao NP, Namtok Phlio NP). ***Southern***: Chumphon (Krom Luang Chumphon WS), Trang (Khao Chong BG), Songkhla (Ton Nga Chang WS), Yala, Pattani (Namtok Sai Khao NP), Nakhon Si Thamarat (Khao Nan NP).

**References.**Bingham (1906).

***Polyrhachis saevissima kerri* Forel, 1911**

Polyrhachis acantha var. kerri Forel, 1911c: 286.

**Distribution. *Northern***: Chiang Mai*.

**References.**Forel (1911c), Emery (1925), Bolton (1995).

***Polyrhachis sculpturata* Smith, 1860**

*Polyrhachis sculpturatus* Smith, 1860b: 70.

**Distribution.** Thailand (unknown locality).

**References.**André (1896), Viehmeyer (1912).

***Polyrhachis sculpturata siamensis* Mayr, 1879**

Polyrhachis sculpturata var. siamensis Mayr, 1879: 657.

**Distribution.** Thailand*.

**References.** Jaitrong and Nabhitabha (2005, cited as *Polyrhachis sumatrensis* Smith, 1858, junior synonym of *P. sculpturata*), Mayr (1879), Emery (1925), Bolton (1995).

***Polyrhachis shixingensis* Wu & Wang, 1995**

Figs 54, 55

*Polyrhachis shixingensis* Wu & Wang, 1995: 166, figs 334, 348, 351.

**Distribution. *Northeastern***: Mukdahan (Phu Sithan WS).

**Remarks.** New record.

**Material examined.** NE Thailand, Mukdahan Prov, Phu Sithan WS, Keang Chang Niam Station, 3.IX.2007, P. Kosonpanyapiwat leg. (THNHM).

***Polyrhachis striata* Mayr, 1862**

*Polyrhachis striatus* Mayr, 1862: 686, pl. 19, fig. 8.

**Distribution. *Northeastern***: Nakhon Ratchasima (Kao Yai NP, Sakaerat) ***Eastern***: Chachoengsao (Khao Ang Reu Nai WS), Chanthaburi (Khao Soi Dao WS). ***Southern***: Nakhon Si Thamarat (Khao Nan NP), Songkhla (Ton Nga Chang NP).

**References.**Jaitrong and Nabhitabhata (2005).

***Polyrhachis textor* Smith, 1857**

*Polyrhachis textor* Smith, 1857: 60.

**Distribution. *Eastern***: Chachoengsao (Khao Ang Reu Nai WS).

**References.**Jaitrong and Nabhitabhata (2005).

***Polyrhachis thailandica* Kohout, 2006**

*Polyrhachis thailandica* Kohout, 2006: 146, figs 3, 7, 11.

**Distribution. *Western***: Kanchanaburi (Mae Klong River*). ***Norteastern***: Chaiyaphum (Phu Khiao WS).

**References.**Kohout (2006).

***Polyrhachis thrinax* Roger, 1863**

*Polyrhachis thrinax* Roger, 1863: 152.

**Distribution. *Northern***: Chiang Mai (Doi Chiang Dao). Western: Tak (Umphang WS). ***Eastern***: Chachoengsao (Khao Ang Reu Nai WS), Chanthaburi (Khao Soi Dao WS).

**References.**Jaitrong and Nabhitabhata (2005).

***Polyrhachis tibialis* Smith, 1858**

*Polyrhachis tibialis* Smith, 1858: 63.

**Distribution. *Western***: Tak (Thung Yai Naresuan East WS), Kanchanaburi (Pha Tad Watershed). ***Northern***: Phrae (Wang Chin). ***Northeastern***: Loei (Phu Luang WS), Chaiyaphum (Phu Khiao WS), Nakhon Ratchasima (Khao Yai). ***Eastern***: Chachoengsao (Khao Ang Reu Nai), Chon Buri (Khao Ang Reu Nai), Chanthaburi (Khao Soi Dao WS, Pheao NP, Nam Tok Pheao). ***Southern***: Satun (Tarutao NP), Pattani (Yaring), Narathiwat (Hala–Bala WS, Toh Daeng), Songkhla (Khao Nam Khang NP, Natawatee).

**References.**Jaitrong and Nabhitabhata (2005).

***Polyrhachis varicolor* Viehmeyer, 1916**

*Polyrhachis fruhstorferi* spp. *varicolor* Viehmeyer, 1916: 163.

**Distribution. *Western***: Prachuap Khiri Khan (Ka Oon Waterfall). ***Southern***: Surat Thani (Khlong Saeng WS), Nakhon Si Thammarat (Khiriwong), Songkhla (Ton Nga Chang WS).

**References.**Kohout (2008), Noon–Anant et al. (2009).

***Polyrhachis venus* Forel, 1893**

*Polyrhachis venus* Forel, 1893a: 31.

**Distribution. *Northern***: Chiang Mai (Doi Suthep–Pui NP).

**References.**Antweb (2019).

***Polyrhachis villipes* Smith, 1857**

*Polyrhachis villipes* Smith, 1857: 61.

**Distribution. *Southern***: Narathiwat (Hala–Bala WS).

**References.** Jaitrong and Nabhitabha (2005, cited as *Polyrhachis sumatraensis* Smith, 1858, junior synomym of *P. villipes*).

***Polyrhachis watanasiti* Kohout, 2013**

*Polyrhachis watanasiti* Kohout, 2013: 40, figs 57, 58, 61, 62.

**Distribution. *Southern***: Ranong (Ngao*).

**References.**Kohout (2013).

***Polyrhachis ypsilon* Emery, 1887**

*Polyrhachis ypsilon* Emery, 1887a: 239.

**Distribution. *Southern***: Ranong (Khlong Na Kha WS), Nakhon Si Thammarat (Khao Nan NP), Songkhla (Ton Nga Chang WS), Narathiwat (Hala–Bala WS).

**References.**Jaitrong and Nabhitabhata (2005).

***Prenolepis darlena* Williams & LaPolla, 2016**

*Prenolepis darlena* Williams & LaPolla, 2016: 219, figs 63–65.

**Distribution. *Northern***: Chiang Mai (Doi Inthanon NP).

**References.**Williams and LaPolla (2016).

***Prenolepis fustinoda* Williams & LaPolla, 2016**

*Prenolepis fustinoda* Williams & LaPolla, 2016: 222, figs 69–71.

**Distribution. *Northern***: Chiang Mai (Doi Inthanon NP*).

**References.**Williams and LaPolla (2016).

***Prenolepis jacobsoni* Crawley, 1923**

*Prenolepis jacobsoni* Crawley, 1923: 30.

**Distribution. *Southern***: Surat Thani (Khlong Saeng WS), Narathiwat (Hala–Bala WS).

**References.**Jaitrong and Nabhitabhata (2005).

***Prenolepis jerdoni* Emery, 1893**

*Prenolepis jerdoni* Emery, 1893b: 222, pl. 8, fig. 20.

**Distribution. *Northern***: Chiang Mai (unknown locality).

**References.**Antmaps (2020).

***Prenolepis melanogaster* Emery, 1893**

*Prenolepis melanogaster* Emery, 1893b: 223.

**Distribution. *Northern***: Chiang Mai (Doi Ang Khang, Mae Taeng, Mae Chaem, Chiang Dao WS, Doi Suthep)

**References.**Jaitrong and Nabhitabhata (2005).

***Prenolepis naoroji* Forel, 1902**

*Prenolepis naoroji* Forel, 1902a: 290.

**Distribution. *Northeastern***: Nakhon Ratchasima (Khao Yai NP, Phu Sithan WS). ***Western***: Tak (Umphnang WS). ***Southern***: Songkhla (Hat Yai), Nakhon Si Thammarat (Khao Nan NP), Trang (Khao Chong).

**References.**Williams and LaPolla (2016).

***Prenolepis shanialena* Williams & LaPolla, 2016**

*Prenolepis shanialena* Williams & LaPolla, 2016: 239, figs 115–117.

**Distribution. *Northern***: Chiang Mai (Doi Inthanon NP).

**References.**Williams and LaPolla (2016).

***Pseudolasius silvestrii* Wheeler, 1927**

*Pseudolasius silvestrii* Wheeler, 1927a: 102, fig. 8.

**Distribution. *Northern***: Chiang Rai (Mae Sai).

**References.**Ran and Zhou (2013).

#### Subfamily Leptanillinae [2 genera, 1 species]

***Leptanilla thai* Baroni Urbani, 1977**

*Leptanilla thai* Baroni Urbani, 1977a: 454, figs 21, 23.

**Distribution. *Southern***: Krabi (Ko Lanta), Trang (Khao Chong BG*).

**References.**Baroni Urbani (1977a), Bolton (1995), Jaitrong and Nabhitabhata (2005), Jaitrong (2011), Jaitrong and Jeenthong (2014a).

#### Subfamily Myrmicinae [40 genera, 216 species]

***Acanthomyrmex ferox* Emery, 1893**

*Acanthomyrmex ferox* Emery, 1893c: 245, pl. 6, fig. 11.

**Distribution. *Southern***: Surat Thani (Khlong Yan WS), Nakhon Si Thammarat (Khao Luang NP, Khao Nan NP), Trang (Khao Chong BG), Songkhla (Ton Nga Chang WS), Narathiwat (Hala–Bala WS).

**References.**Jaitrong and Nabhitabhata (2005), Jaitrong (2011), Jaitrong and Asanok (2019).

***Acanthomyrmex malikuli* Jaitrong & Asanok, 2019**

*Acanthomyrmex malikuli* Jaitrong & Asanok, 2019: 116, figs 1–3.

**Distribution. *Western***: Tak (Umphang WS, Thung Yai Naresuan East WS*).

**References.**Jaitrong and Asanok (2019).

***Acanthomyrmex mizunoi* Jaitrong & Asanok, 2019**

*Acanthomyrmex mizunoi* Jaitrong & Asanok, 2019: 124, figs 4–6. Type locality: THAILAND, Nakhon Nayok province, Mueang district, Ban Hin Tang.

**Distribution. *Northern***: Chiang Rai (Huai Pong Coffee Plantation). ***Northeastern***: Nakhon Ratchasima (Pak Chong). ***Central***: Nakhon Nayok (Ban Hin Tang*).

**References.**Jaitrong and Asanok (2019).

***Acanthomyrmex thailandensis* Terayama, 1995**

*Acanthomyrmex thailandensis* Terayama, 1995: 551, figs 1–9.

**Distribution. *Northern***: Chiang Mai (Doi Suthep–Pui NP*, Omkoi).

**References.**Terayama (1995), Jaitrong and Asanok (2019).

***Anillomyrma decamera* (Emery, 1901)**

*Monomorium decamerum* Emery, 1901: 117.

**Distribution. *Northern***: Chiang Mai (Omkoi). ***Western***: Kanchanaburi (Ban Sahakorn Nikhom).

**References.**Yamane and Jaitrong (2019).

***Calyptomyrmex beccarii* Emery, 1887**

*Calyptomyrmex beccarii* Emery, 1887c: 472, pl. 2, fig. 23.

**Distribution. *Southern***: Narathiwat (Hala–Bala WS).

**References.**Jaitrong and Nabhitabhata (2005, cited as *Calyptomyrmex emeryi* Forel, 1901b, junior synonym of *C. beccarii*.), Jaitrong and Yamane (2018).

***Calyptomyrmex rectopilosus* Dlussky & Radchenko, 1990**

*Calyptomyrmex rectopilosus* Dlussky & Radchenko, 1990: 124, figs 7, 8.

**Distribution. *Northern***: Chiang Mai (Doi Suthep–Pui NP). ***Western***: Tak (Umphang WS), Kanchanaburi (Thong Pha Phum NP, Khuean Srinagarindra NP). ***Northeastern***: Nakhon Ratchasima (Forestry Camp, Khao Yai NP). ***Eastern***: Chachoengsao (Khao Ang Reu Nai WS), Chanthaburi (Khao Soi Dao WS) ***Central***: Saraburi (Korkod waterfall), Nakhon Nayok (Nang Rong waterfall). ***Southern***: Ranong (Khlong Na Kha WS), Nakhon Si Thammarat (Tapi Watershed Research Station, Khao Luang NP).

**References.**Jaitrong (2011), Jaitrong and Yamane (2018).

***Cardiocondyla emeryi* Forel, 1881**

*Cardiocondyla emeryi* Forel, 1881: 5.

**Distribution. *Northern***: Chiang Mai (Mae Chaem, Mae Taeng). ***Northeastern***: Nakhon Ratchasima (Khao Yai NP). ***Central***: Bangkok (Kasetsart University). ***Southern***: Trang (Khao Chong BG), Songkhla (Khao Nam Khang), Narathiwat (Hala–Bala WS).

**References.**Jaitrong and Nabhitabhata (2005).

***Cardiocondyla itsukii* Seifert, Okita & Heinze, 2017**

*Cardiocondyla itsukii* Seifert, Okita & Heinze, 2017: 339–344, figs 10–12.

**Distribution. *Western***: Tak (Umphang WS), Kanchanaburi (Thong Pha Phum NP). ***Eastern***: Trat (Ko Chang)

**References.** Seifert et al. (2017)

***Cardiocondyla kagutsuchi* Terayama, 1999**

*Cardiocondyla kagutsuchi* Terayama, 1999: 100.

**Distribution. *Northern***: Chiang Mai (Mae Chaem, Mae Taeng, Ob Luang NP, Doi Suthep–Pui NP), Mae Hong Son (Mae Sariang). ***Northeastern***: Nakhon Ratchasima (Sakaerat, Khao Yai NP). ***Central***: Pathum Thani (Khlong Luang). ***Southern***: Phatthalung (Khao Lak), Songkhla (Khao Nam Khang).

**References.** Seifert et al. (2017)

***Cardiocondyla wroughtonii* (Forel, 1890)**

*Emeryia wroughtonii* Forel, 1890a: cxi.

**Distribution. *Northern***: Chiang Mai (Mae Taeng, Doi Suthep–Pui NP). ***Western***: Tak (Umphang WS), Kanchanaburi (Thong Pha Phum NP). ***Northeastern***: Nakhon Ratchasima (Sakaerat, Khao Yai NP). ***Southern***: Songkhla (Khao Nam Khang).

**References.**Jaitrong and Nabhitabhata (2005), Sakchoowong et al. (2009).

***Carebara affinis* (Jerdon, 1851)**

*Oecodoma affinis* Jerdon, 1851: 110.

**Distribution. *Northern***: Chiang Rai (Doi Tung), Chiang Mai (Khun Chang Khian, Pa Miang Village, Doi Ang Khang, Doi Luang Chiang Dao, Chiang Dao WS, Mae Taeng, Doi Suthep–Pui NP, Chiang Mai University Campus, Doi Inthanon NP, Mae Chaem), Phayao (Mae Ka), Lamphun (Mae Li Forest Plantation), Lampang (Haui Tak, Tham Pha Thai NP), Phrae (Wang Chin Plantation), Nan (Doi Phu Kha NP, Nakhon Nan Forest Plantation) ***Western***: Tak (Umphang WS, Thung Yai Naresuan East WS, Lansang NP, Taksin Maharat NP), Kanchanaburi (Thong Pha Phum NP, Pha Tad Watershed Management station), Phetchaburi (Kaeng Krachan NP), Prachuap Khiri Khan (Kui Buri). ***Northeastern***: Kalasin (Phu SithanWS), Chaiyaphum (Phu Khiao WS), Loei (Phu Luang WS), Nakhon Ratchasima (Sakaerat, Khao Yai NP, Buer Yai), Ubon Ratchathani (Pha Taem NP). ***Central***: Uthai Thani (Huai Kha Khaeng WS), Saraburi (Phukae BG, Namtok Sam Lan NP), Bangkok (Bang Khen, Chatuchak) ***Eastern***: Chachoengsao (Khao Ang Reu Nai WS), Chon Buri (Si Racha, Khao Kheow), Chanthaburi (Khao Soi Dao WS, Pheao NP, Khao Ang Reu Nai WS). ***Southern***: Chumphon (Krom Luang Chumphon NP), Ranong (Khlong Na Kha WS), Surat Thani (Tai Rom Yen NP, Khlong Yan WS), Nakhon Si Thammarat (Tapi Watershed Research Station, Khao Nan NP, Khao Luang NP, Krung Ching Waterfall), Krabi (Ko Lanta), Trang (Khao Chong BG, Thung Khai BG, Palian), Phatthalung (Khao Pu–Khao Ya NP, Khao Bantad WS), Satun (Tarutao NP), Songkhla (Khao Kho Hong, Khao Nam Khang NP), Yala, Narathiwat (Hala–Bala WS).

**References.**Bingham (1906), Jaitrong and Nabhitabhata (2005, cited as *Pheidologeton affinis* (Jerdon, 1851)), Jaitrong and Jeenthong (2014a, cited as *Pheidologeton affinis* (Jerdon, 1851)), Sakchoowong et al. (2008, cited as *Pheidologeton affinis* (Jerdon, 1851)), Sakchoowong et al. (2009, cited as *Pheidologeton affinis* (Jerdon, 1851)), Onishi et al. (2016).

***Carebara castanea* Smith, 1858**

*Carebara castanea* Smith, 1858: 178.

**Distribution. *Northern***: Chiang Rai (Doi Tung), Chiang Mai (Mae Chaem, Mae Taeng, Fang, Chiang Dao WS, Doi Suthep–Pui NP, Prao), Phayao (Mae Ka), Lampang (Tham Pha Thai NP). ***Western***: Tak (Thung Yai Naresuan East WS), Kanchanaburi (Thong Pha Phum NP). ***Northeastern***: Nakhon Ratchasima (Sakaerat, Khao Yai NP). ***Southern***: Chumphon (Krom Luang Chumphon WS).

**References.**Jaitrong and Nabhitabhata (2005), Sakchoowong et al. (2009), Jaitrong (2011).

***Carebara diversa* (Jerdon, 1851)**

*Oecodoma diversa* Jerdon, 1851: 109.

**Distribution. *Northern***: Chiang Rai (Doi Tung), Chiang Mai (Khun Chang Khian, Pa Miang Village, Doi Ang Khang, Doi Luang Chiang Dao, Chiang Dao WS, Mae Taeng, Doi Suthep–Pui NP, Chiang Mai University Campus, Doi Inthanon NP, Mae Chaem, Omkoi), Phayao (Mae Ka), Lamphun (Mae Li Forest Plantation), Lampang (Haui Tak, Tham Pha Thai NP), Phrae (Wang Chin Forest Plantation), Nan (Doi Phu Kha NP, Nakhon Nan Forest Plantation) ***Western***: Tak (Umphang WS, Thung Yai Naresuan East WS, Lansang NP, Taksin Maharat NP), Kanchanaburi (Thong Pha Phum NP, Pha Tad Watershed Management station), Phetchaburi (Kaeng Krachan NP), Prachuap Khiri Khan (Kui Buri). ***Northeastern***: Kalasin (Phu SithanWS), Chaiyaphum (Phu Khiao WS), Loei (Phu Luang WS), Nakhon Ratchasima (Sakaerat, Khao Yai NP, Buer Yai), Ubon Ratchathani (Pha Taem NP). ***Central***: Uthai Thani (Huai Kha Khaeng WS), Saraburi (Phukae BG), Nakhon Nayok (Sarika Waterfall), Bangkok (Bang Khen, Chatuchak), Pathum Thani (Khlong Luang), Samut Prakan (Bang Krachao), Samut Songkhram (Mueang Samut Songkhram). ***Eastern***: Chachoengsao (Khao Ang Reu Nai WS), Chon Buri (Si Racha, Khao Kheow), Chanthaburi (Khao Soi Dao WS, Pheao NP, Khao Ang Reu Nai WS). ***Southern***: Chumphon (Krom Luang Chumphon NP), Ranong (Khlong Na Kha WS), Surat Thani (Tai Rom Yen NP, Khlong Yan WS), Nakhon Si Thammarat (Khao Nan NP, Khao Luang NP, Krung Ching Waterfall), Krabi (Ko Lanta), Trang (Khao Chong BG, Thung Khai BG, Palian), Phatthalung (Khao Pu–Khao Ya NP, Khao Bantad WS), Satun (Tarutao NP), Songkhla (Khao Kho Hong, Khao Nam Khang NP), Narathiwat (Hala–Bala WS).

**References.**Jaitrong and Nabhitabhata (2005, cited as *Pheidologeton diversus* (Jerdon, 1851)), Sakchoowong et al. (2008, cited as *Pheidologeton diversus* (Jerdon, 1851)), Sakchoowong et al. (2009, cited as *Pheidologeton diversus* (Jerdon, 1851)), Jaitrong and Jeenthong (2014a), Suwannaphak et al. (2016), Onishi et al. (2016).

***Carebara lignata* Westwood, 1840**

*Carebara lignata* Westwood, 1840: 86, pl. 2, fig. 6.

**Distribution. *Northern***: Nan (Wiang Sa).

**References.**Collingwood (1962); Sitthicharoenchai and Chantarasawat (2006).

***Carebara pygmaea* (Emery, 1887)**

*Pheidologeton pygmaeus* Emery, 1887f: 465.

**Distribution. *Northern***: Chiang Rai (Doi Tung). ***Western***: Tak (Umphang WS, Thung Yai Naresuan East WS), Kanchanaburi (Thong Pha Phum NP, Maekhlong Watershed). ***Northeastern***: Chaiyaphum (Phu Khiao WS), Nakhon Ratchasima (Sakaerat, Khao Yai NP). ***Central***: Uthai Thani (Huai Kha Khaeng WS). ***Eastern***: Chachoengsao (Khao Ang ReuNai WS), Rayong (Khao Ang Reu Nai WS), Chanthaburi (Khao Soi Dao WS, Pheao NP). ***Southern***: Surat Thani (Tai Rom Yen NP, Khlong Yan WS), Satun (Tarutao NP), Nakhon Si Thammarat (Khao Nan NP).

**References.**Jaitrong and Nabhitabhata (2005, cited as *Pheidologeton pygmaeus* Emery, 1887), Sakchoowong et al. (2009, cited as *Pheidologeton pygmaeus* Emery, 1887).

***Carebara silenus* (Smith, 1858)**

*Pheidole silenus* Smith, 1858: 176.

**Distribution. *Southern***: Ranong (Khlong Na Kha WS), Surat Thani (Tai Rom Yen NP, Khlong Yan WS), Nakhon Si Thammarat (Tapi Watershed Research Station, Khao Nan NP), Phatthalung (Khao Pappha), Trang (Khao Chong BG), Songkhla (Khao Nam Khang NP, Ton Nga Chang), Narathiwat (Hala–Bala WS).

**References.**Jaitrong and Nabhitabhata (2005, cited as *Pheidologeton silenus* (Smith, 1858).

***Carebara trechideros* (Zhou & Zheng, 1997)**

*Pheidologeton trechideros* Zhou & Zheng, 1997: 167, figs 7–9.

**Distribution. *Northern***: Chiang Mai (Doi Ang Khang, Doi Inthanon NP, Doi Pha Hom Pok NP). ***Western***: Tak (Thung Yai Naresuan East WS).

**References.**Jaitrong and Nabhitabhata (2005, cited as *Pheidologeton trechideros* Zhou & Zheng, 1997).

***Cataulacus granulatus* (Latreille, 1802)**

*Formica granulata* Latreille, 1802: 275, pl. 12, fig. 75.

**Distribution. *Northern***: Chiang Rai (Doi Tung), Chiang Mai (Pa Miang Village, Doi Ang Khang, Doi Luang Chiang Dao, Chiang Dao WS, Mae Taeng, Doi Suthep–Pui NP, Chiang Mai University Campus, Doi Inthanon NP, Mae Chaem), Phayao (Mae Ka), Lamphun (Mae Li Forest Plantation), Lampang (Haui Tak, Tham Pha Thai NP), Phrae (Wang Chin Forest Plantation), Nan (Doi Phu Kha NP, Nakhon Nan Forest Plantation). ***Western***: Tak (Umphang WS, Thung Yai Naresuan East WS, Lansang NP, Taksin Maharat NP), Kanchanaburi (Thong Pha Phum NP, Pha Tad Watershed Management station), Phetchaburi (Kaeng Krachan NP), Prachuap Khiri Khan (Kui Buri). ***Northeastern***: Kalasin (Phu Sithan WS), Chaiyaphum (Phu Khiao WS), Loei (Phu Luang WS), Nakhon Ratchasima (Sakaerat, Khao Yai NP, Buer Yai), Ubon Ratchathani (Pha Taem NP). ***Central***: Saraburi (Phukae BG), Bangkok (Bang Khen, Chatuchak), Pathum Thani (Khlong Luang), Samut Prakan (Bang Krachao), Samut Songkhram (Mueang Samut Songkhram). ***Eastern***: Sa Kaeo (Khao Ang Reu Nai WS), Chachoengsao (Khao Ang Reu Nai WS), Chon Buri (Si Racha, Khao Kheow), Chanthaburi (Khao Soi Dao WS, Pheao NP, Khao Ang Reu Nai WS), Rayong (Ko Man Nai, Khao Ang Reu Nai WS). ***Southern***: Chumphon (Krom Luang Chumphon NP), Ranong (Suk Samran, Khlong Na Kha WS), Surat Thani (Tai Rom Yen NP, Khlong Yan WS, Mu Ko Ang Thong NP), Nakhon Si Thammarat (Khao Nan NP, Khao Luang NP, Krung Ching Waterfall), Krabi (Ko Lanta), Trang (Khao Chong BG, Thung Khai BG, Palian), Phatthalung (Khao Pu–Khao Ya NP, Khao Bantad WS), Satun (Tarutao NP), Songkhla (Khao Kho Hong, Khao Nam Khang NP), Yala, Pattani (Yaring), Narathiwat (Hala–Bala WS).

**References.**Bingham (1906), Jaitrong and Nabhitabhata (2005), Sakchoowong et al. (2009), Onishi et al. (2016).

***Cataulacus horridus* Smith, 1857**

*Cataulacus horridus* Smith, 1857: 81, pl. 2, fig. 3.

**Distribution. *Southern***: Narathiwat (Hala–Bala WS).

**References.**Jaitrong and Nabhitabhata (2005).

***Cataulacus latus* Forel, 1891**

*Cataulacus latus* Forel, 1891: 144.

**Distribution. *Southern***: Yala.

**References.**Bingham (1906).

***Cataulacus muticus* Emery, 1889**

Figs 56, 57

*Cataulacus muticus* Emery, 1889a: 507, pl. 10, fig. 17

**Distribution. *Western***: Kanchanaburi (Khuean Srinagarindra NP).

**Remarks.** New record.

**Material examined.** W Thailand, Kanchanaburi Prov, Si Sawat Dist, Khuean Sri Nakarin NP, 20–21.III.2014, W. Jaitrong leg., WJT200314–HC2 (Phak) (THNHM).

***Cataulacus praetextus* Smith, 1867**

Figs 58, 59

*Cataulacus praetextus* Smith, 1867: 528.

**Distribution. *Western***: Phetchaburi (Kaeng Krachan).

**Remarks.** New record.

**Material examined.** W. Thaialnd, Phetchaburi Prov, Kaeng Krachan, 27.VI.2014, Sk. Yamane & M. Maruyama leg., TH14–SKY–66 (SKYC).

***Crematogaster aberrans* Forel, 1892**

*Crematogaster aberrans* Forel, 1892c: 532.

**Distribution. *Northern***: Chiang Mai (Chiang Dao WS), Lampang (Ngao). ***Central***: Bangkok.

**References.**Hosoishi (2015).

***Crematogaster artifex* Mayr, 1879**

*Crematogaster artifex* Mayr, 1879: 684.

**Distribution. *Central***: Bangkok*.

**References.**Mayr (1879), Bolton (1995), Hosoishi and Ogata (2009).

***Crematogaster aurita* Karavaiev, 1935**

*Crematogaster aurita* Karavaiev, 1935: 92, fig. 18.

**Distribution. *Northern***: Chiang Mai (Doi Chiang Dao, Omkoi), Lampang (Tham Pha Thai NP, Huai Tak), Phayao (Mae Ka, Mae Yod), Phrae (Wang Chin), Lamphun (Mae Li Forest Plantation). ***Western***: Tak (Lansang NP), Kanchanaburi (Mae Khlong, Khuean Srinagarindra NP). ***Northeastern***: Kalasin (Phu Sithan WS), Mukdahan (Phu Sithan WS), Ubon Ratchathani (Pha Tam NP, Ubon Ratchathani Zoo), Loei (Phu Luang WS). ***Central***: Uthai Thani (Huai Kha Khaeng WS), Saraburi (Phu Kae BG).

**References.**Hosoishi (2009), Hosoishi and Ogata (2009).

***Crematogaster baduvi* Forel, 1912**

Figs 60, 61

*Crematogaster baduvi* Forel, 1912a: 106.

**Distribution. *Southern***: Ranong (Suk Samran), Surat Thani (Tai Rom Yen NP).

**Remarks.** New record.

**Material examined.** S Thailand, Ranong Prov, Suk Samran Dist., Ban Nuar village, 24.iv.2018, Sk. Yamane, TH18–SKY–031 (SKYC); S Thailand, Surat Thani Prov, Tai Rom Yen NP, Datfa Waterfall, 12.x.2011, Sk. Yamane, TH11–SKY–042 (SKYC).

***Crematogaster bandarensis* Forel, 1913**

Crematogaster biroi var. bandarensis Forel, 1913: 76.

**Distribution. *Western***: Tak (Thung Yai Naresuan East WS). ***Southern***: Surat Thani (Khlong SaengWS), Nakhon Si Thammarat (Tai Rom Yen NP), Songkhla (Ton Nga Chang).

**References.**Hosoishi and Ogata (2016).

***Crematogaster binghamii* Forel, 1904**

*Crematogaster binghamii* Forel, 1904: 24.

**Distribution. *Eastern***: Chachoengsao (Khao Ang Reu Nai WS).

**References.**Hosoishi and Ogata (2016).

***Crematogaster bouvardi* Santschi, 1920**

Crematogaster walshi st. bourvardi Santschi, 1920.

**Distribution. *Northern***: Chiang Mai (Doi Suthep).

**References.**Hosoishi and Ogata (2012).

***Crematogaster coriaria* Mayr, 1872**

*Crematogaster coriaria* Mayr, 1872: 154.

**Distribution. *Northern***: Chaiang Mai (Doi Suthep–Pui NP). ***Western***: Kanchanaburi (Thong Pha Phum NP), Tak (Umphang). ***Northeastern***: Nakhon Ratchasima (Khao Yai NP, Sakaerat). ***Eastern***: Chachoengsao (Khao Ang Reu Nai WS). ***Southern***: Trang (Khao Chong BG).

**References.**Jaitrong and Nabhitabhata (2005), Hosoishi and Ogata (2009), Sakchoowong et al. (2009).

***Crematogaster difformis* Smith, 1857**

*Crematogaster difformis* Smith, 1857: 76.

**Distribution. *Eastern***: Chanthaburi (Khao Soi Dao WS). ***Southern***: Yala.

**References.**Bingham (1906), Jaitrong and Nabhitabhata (2005), Hosoishi and Ogata (2009).

***Crematogaster dohrni* Mayr, 1879**

*Crematogaster dohrni* Mayr, 1879: 682.

**Distribution. *Northern***: Chiang Mai (unknown locality).

**References.**Antweb (2019).

***Crematogaster dohrni fabricans* Forel, 1911**

Crematogaster rogenhoferi var. fabricans Forel, 1911e: 201.

**Distribution.** Unknown locality.

**References.**Forel (1911e).

***Crematogaster dohrni kerri* Forel, 1911**

Crematogaster rogenhoferi subsp. kerri Forel, 1911d: 284.

**Distribution. *Northern***: Chiang Mai*.

**References.**Forel (1911d), Bolton (1995), Hosoishi and Ogata (2009).

***Crematogaster dubia* Karavaiev, 1935**

Figs 62, 63

Crematogaster (Paracrema) dubia Karavaiev, 1935: 93, fig. 19.

**Distribution. *Northern***: Chiang Mai (Doi Suthep–Pui NP). ***Northeastern***: Nakhon Ratchasima (Khai Yai NP, Sakaerat). ***Western***: Tak (Umphang WS, Thung Yai Naresuan East WS).

**Remarks.** New record.

**Material examined.** N Thailand, Chiang Mai Prov, Doi Pui, 22.xii.1997, F. Yamane; same loc., 18.VIII.1998, Sk. Yamane; N Thailand, Chiang Mai Prov, Doi Suthep–Pui, 7.vi.2001, K. Eguchi, Eg01–TH–076. NE Thailand, Nakhon Ratchasima Prov, Sakaerat, 10.VII.1999, Sk. Yamane leg (SKYC); NE Thailand, Nakhon Ratchasima Prov, Khao Yai NP, 30.V.2000, Sk. Yamane; same locality, 21.II.1998, W. Jaitrong. W Thailand, Tak Prov, Umphang Dist, Umphang WS, Mae Khlong Ki, 25.I.2015, W. Jaitrong leg., TH15–WJT–043 (THNHM); same loc., date and collector, TH15–WJT–021 (THNHM); same locality, date and collector, TH15–WJT–22 (THNHM); W Thailand, Tak Prov, Umphang Dist, Umphang WS, Mae Khlong Yai, 26.V.2015, W. Jaitrong leg., TH15–WJT–649 (THNHM); same locality, date and collector, TH15–WJT–646 (THNHM); W Thailand, Tak Prov, Umphang Dist, Umphang WS, Pha Luard, 30.V.2015, W. Jaitrong leg., TH15–WJT–759 (THNHM); W Thailand, Tak Prov, Umphang Dist, Umphang WS, Doi Huar Mod, 31.V.2015, W. Jaitrong leg., TH15–WJT–792 (THNHM); W Thailand, Tak Prov, Umphang Dist, Umphang WS, km 15 from Mae Sod, 26.V.2015, W. Jaitrong leg., TH15–WJT–283 (THNHM); W Thailand, Tak Prov, Unphang WS, Doi Cha Rod Pha, 26.V.2015, Sk. Yamane, TH15–SKY–167 (SKYC).

**Figures 62–71.**
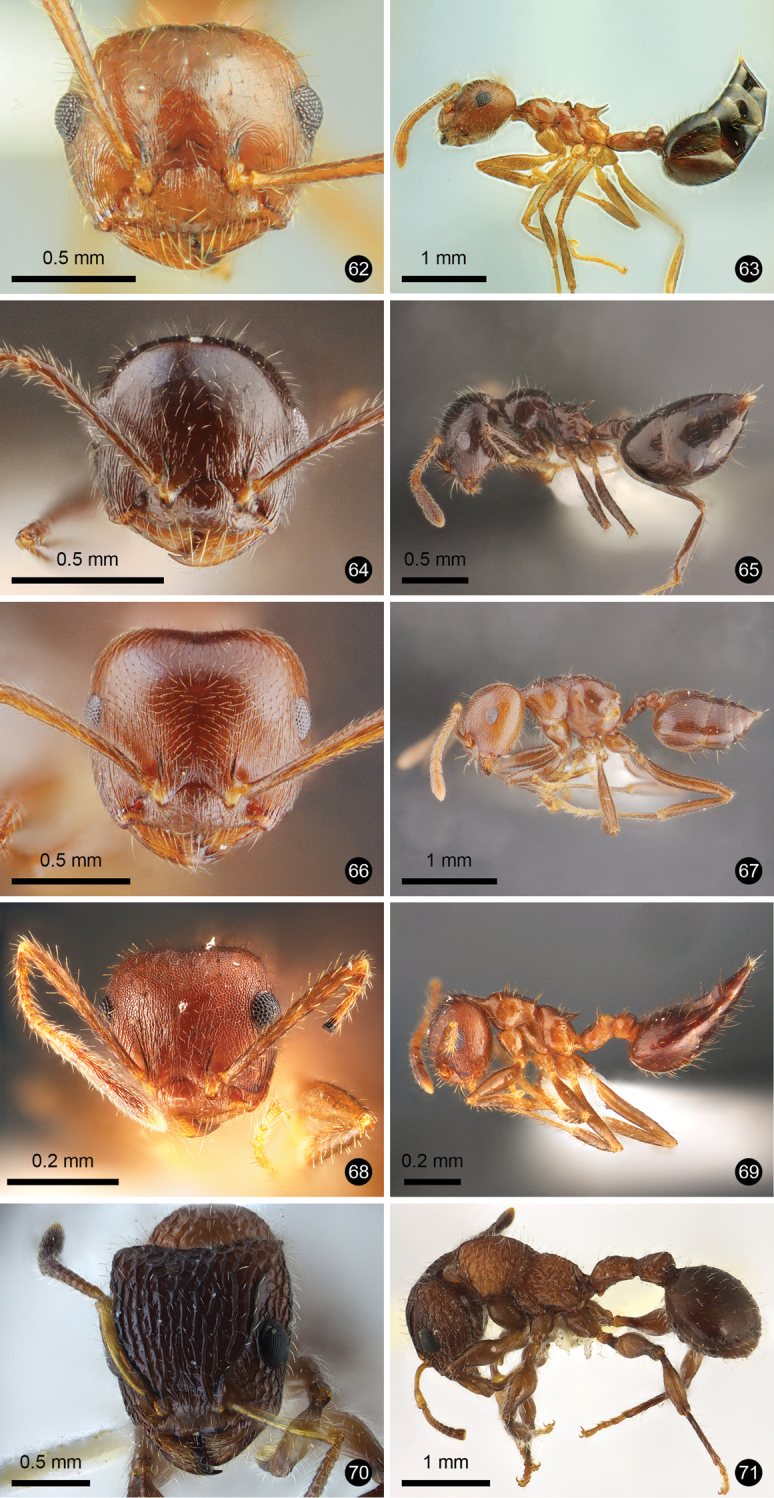
Ant species new to Thailand. **62, 63***Crematogaster dubia***64, 65***Crematogaster ferrarii***66, 67***Crematogaster onusta***68, 69***Crematogaster rothneyi***70, 71***Dilobocondyla fouqueti*.

***Crematogaster ferrarii* Emery, 1888**

Figs 64, 65

*Crematogaster ferrarii* Emery, 1888: 533.

**Distribution. *Northern***: Chiang Mai (Doi Chiang Dao). ***Western***: Tak (Umphang WS, Thung Yai Naresuan East WS, Mae Sot), Kanchanaburi (Maeklong Watershed, Pha Tad Watershed), Phetchaburi (Kaeng Krachan NP). ***Northeastern***: Nakhon Ratchasima (Khao Yai NP) ***Southern***: Nakhon Si Thammarat (Tapi Watershed), Songkhla (Hat Yai).

**Remarks.** New record.

**Material examined.** N Thailand, Chiang Mai Prov, Doi Chiang Dao, 3.IV.2005, Sk. Yamane leg., TH05–SKY–60 (SKYC). W Thailand, Tak Prov, Umphang Dist, Umphang WS, Doi Huar Mod, 27.I.2015, W. Jaitrong leg., TH15–WJT–86 (THNHM); W Thailand, Tak Prov, Umphang Dist, Umphang WS, Mae Khlong Ki, 25.V.2015, W. Jaitrong leg., WJT250515–2 (THNHM); W Thailand, Tak Prov, Umphang Dist, Umphang WS, Doi Cha Rod Fa, 26.I.2015, Sk. Yamane leg., TH15–SKY–195 (SKYC, THNHM); W Thailand, Tak Prov, Umphang Dist, Umphang WS, Pha Luard, 30.I.2015, Sk. Yamane leg., TH15–SKY–234 (SKYC, THNHM); W Thailand, Tak Prov, Mae Sot dist, 31.i.2015, Sk. Yamane, TH15–SKY–195; W Thailand, Kanchanaburi Prov, Maeklong Watershed Res. Stn., 29.xi.2003, Sk. Yamane (SKYC); W Thaialnd, Kanchanaburi Prov, Pha Tad Watershed, 30.xi.2003, Sk. Yamane (SKYC); W Thailand, Phetchaburi Prov, Kaeng Krachan NP, 25.vi.2014, Sk. Yamane & M. Maruyama leg., TH14–SKY–46. NE Thailand, Nakhon Ratchasima Prov, Khao Yai NP, 30.V.2000, Sk. Yamane; NE Thailand, Nakhon Ratchasima Prov, Sakaerat, 9.vii.1999, lowland DDF, Sk. Yamane (SKYC). S Thailand, Nakhon Si Thamarat Prov, Tapi Watershed, 13.x.2011, Sk. Yamane leg., TH11–SKY–70 (SKYC); S Thailand, Songkhla Prov, Hat Yai, 26.VII.1997, H. Okido leg. (SKYC).

***Crematogaster fraxatrix* Forel, 1911**

*Crematogaster fraxatrix* Forel, 1911a: 28.

**Distribution. *Southern***: Nakhon Si Thammarat (Tapi Watershed Research Station, Khao Nan NP), Surat Thani (Tai Rom Yen NP).

**References.**Hosoishi and Ogata (2014).

***Crematogaster fumikoae* Hosoishi & Ogata, 2015**

*Crematogaster fumikoae* Hosoishi & Ogata, 2015: 14.

**Distribution. *Northern***: Chiang Mai (Doi Suthep–Pui NP*, Doi Ang Khang). ***Western***: Tak (Umphang WS).

**References.**Hosoishi and Ogata (2015).

***Crematogaster hashimi* Hosoishi, 2015**

*Crematogaster hashimi* Hosoishi, 2015: 80, fig. 31.

**Distribution. *Western***: Kanchanaburi (Mae Klong).

**References.**Hosoishi (2015).

***Crematogaster inflata* Smith, 1857**

*Crematogaster inflatus* Smith, 1857: 76, pl.2, fig. 2.

**Distribution. *Southern***: Nakhon Si Thammarat (Khao Luang NP, Khao Nan NP), Narathiwat (Hala–Bala WS).

**References.**Jaitrong and Nabhitabhata (2005); Hosoishi (2009), Hosoishi and Ogata (2009).

***Crematogaster longipilosa* Forel, 1907**

*Crematogaster longipilosa* Forel, 1907: 24.

**Distribution. *Western***: Tak (Thung Yai Naresuan East WS), Phetchaburi (Kaeng Krachan). ***Southern***: Nakhon Si Thammarat (Khao Nan NP), Songkhla (Khao Nam Khang).

**References.**Hosoishi (2009), Hosoishi and Ogata (2016).

***Crematogaster modiglianii* Emery, 1900**

*Crematogaster modiglianii* Emery, 1900a: 688.

**Distribution. *Western***: Tak (Thung Yai Naresuan East WS), Phetchaburi (Kaeng Krachan NP). ***Southern***: Chumphon (Krom Luang Chumphon WS), Surat Thani (Tai Rom yen NP), Phatthalung (Khao Pappha), Trang (Khao Chong BG), Songkhla (Hat Yai, Khao Nam Khang NP, Ton Nga Chang WS), Pattani (Nong Chik), Narathiwat (Toh Daeng), Nakhon Si Thammarat (Khao Nan NP).

**References.**Bingham (1906), Jaitrong and Nabhitabhata (2005), Hosoishi et al. (2011).

***Crematogaster onusta* Stitz, 1925**

Figs 66, 67

Crematogaster (Physocrema) onusta Stitz, 1925: 118.

**Distribution. *Southern***: Ranong (Khlong Na Kha WS).

**Remarks.** New record.

**Material examined.** S Thailand, Ranong Prov, Khlong Na Kha WS, Evergreen Forest, 12.VII.2009, W. Jaitrong leg., WJT09–TH2071 (THNHM, SKYC); same locality, date and collector, WJT09–TH2080 (THNHM).

***Crematogaster physothorax* Emery, 1889**

Crematogaster deformis r. physothorax Emery, 1889a: 509.

**Distribution. *Southern***: Trang (Ton Tae Waterfall), Narathiwat (Hala–Bala WS).

**References.**Hosoishi (2009), Hosoishi and Ogata (2009).

***Crematogaster pia* Forel, 1911**

Crematogaster tumidula subsp. pia Forel, 1911b: 384.

**Distribution. *Northern***: Phayao (Mae Ka). ***Western***: Phetchaburi (Kaeng Krachan NP). ***Central***: Sukhothai (Thachai). ***Southern***: Ranong (Khlong Na Kha WS).

**References.**Hosoishi (2015).

***Crematogaster quadriruga* Forel, 1911**

Crematogaster biroi var. quadriruga Forel, 1911f: 455.

**Distribution. *Northern***: Chaing Mai (Chiang Mai, Doi Chiang Dao, Doi Suthep). Northeastern: Nakhon Ratchasima (Sakaerat). ***Western***: Kanchanaburi (Pha Tad Watershed) , Phetchaburi (Kaeng Krachan). ***Eastern***: Chacheongsao (Khao Ang Reu Nai WS), Chon Buri (Si Racha). ***Central***: Bangkok. ***Southern***: Ranong (Khlong Na Kha WS, Suk Samran), Nakhon Si Thammarat (Khao Nan NP, Tai Rom Yen NP), Pattani (Namtok Sai Khao NP), Songkhla (Hat Yai).

**References.**Hosoishi and Ogata (2016).

***Crematogaster reticulata* Hosoishi, 2009**

*Crematogaster reticulata* Hosoishi, 2009: 259, figs 1–3.

**Distribution. *Southern***: Ranong (Khlong Na Kha WS), Nakhon Si Thammarat (Khao Nan NP, Tai Rom Yen NP), Surat Thani (Tai Rom Yen NP).

**References.**Hosoishi and Ogata (2016).

***Crematogaster rogenhoferi* Mayr, 1879**

*Crematogaster rogenhoferi* Mayr, 1879: 683.

**Distribution. *Northern***: Chiang Rai (Doi Tung), Chiang Mai (Khun Chang Khian, Pa Miang Village, Doi Ang Khang, Doi Luang Chiang Dao, Chiang Dao WS, Mae Taeng, Doi Suthep–Pui NP, Chiang Mai University Campus, Doi Inthanon NP, Mae Chaem, Omkoi), Phayao (Mae Ka), Lamphun (Mae Li Forest Plantation), Lampang (Haui Tak, Tham Pha Thai NP), Phrae (Wang Chin Forest Plantation), Nan (Doi Phu Kha NP, Nakhon Nan Forest Plantation). ***Western***: Tak (Umphang WS, Thung Yai Naresuan East WS, Lansang NP, Taksin Maharat NP), Kanchanaburi (Thong Pha Phum NP, Pha Tad Watershed Management station), Phetchaburi (Kaeng Krachan NP), Prachuap Khiri Khan (Kui Buri). ***Northeastern***: Kalasin (Phu Sithan WS), Chaiyaphum (Phu Khiao WS), Loei (Phu Luang WS), Nakhon Ratchasima (Sakaerat, Khao Yai NP, Buer Yai), Ubon Ratchathani (Pha Taem NP). ***Central***: Uthai Thani (Huai Kha Khaeng WS), Saraburi (Phukae BG), Bangkok (Bang Khen, Chatuchak), Pathum Thani (Khlong Luang), Samut Songkhram (Mueang Samut Songkhram). ***Eastern***: Sa Kaeo (Khao Ang Reu Nai WS), Chachoengsao (Khao Ang Reu Nai WS), Chon Buri (Si Racha, Khao Kheow), Chanthaburi (Khao Soi Dao WS, Pheao NP, Khao Ang Reu Nai WS), Rayong (Ko Man Nai, Khao Ang Reu Nai WS). ***Southern***: Chumphon (Krom Luang Chumphon NP), Ranong (Suk Samran, Khlong Na Kha WS), Surat Thani (Tai Rom Yen NP, Khlong Yan WS, Mu Ko Ang Thong NP), Nakhon Si Thammarat (Khao Nan NP, Khao Luang NP, Krung Ching Waterfall), Krabi (Ko Lanta), Trang (Khao Chong BG, Thung Khai BG, Palian), Phatthalung (Khao Pu–Khao Ya NP, Khao Bantad WS), Satun (Tarutao NP), Songkhla (Khao Kho Hong, Khao Nam Khang NP), Pattani (Yaring), Narathiwat (Hala–Bala WS).

**References.**Jaitrong and Nabhitabhata (2005), Sakchoowong et al. (2009), Suwannaphak et al. (2016), Onishi et al. (2016).

***Crematogaster rothneyi* Mayr, 1879**

Figs 68, 69

*Crematogaster rothneyi* Mayr, 1879: 685.

**Distribution. *Northern***: ChiangMai (Doi Suthep)

**Remarks.** New record.

**Material examined.** N Thailand, Chiang Mai Prov, Doi Suthep, 22.XII.1997, Sk. Yamane (SKYC).

***Crematogaster sewardi* Forel, 1901**

Crematogaster deformis r. sewardi Forel, 1901b: 64.

**Distribution. *Northeastern***: Nakhon Ratchasima (Sakaerat). ***Central***: Saraburi (Kok E–dok Waterfall), Nakhon Nayok (Nang Rong waterfall).

**Northestern**: Chacheongsao (Khao Ang Reu Nai). ***Eastern***: Chanthaburi (Khao Soi Dao WS, Pheao NP, Namtok Phlio NP). ***Southern***: Trang (Khao Chong BG), Pattani (Namtok Sai Khao NP), Ranong (Suk Samran), Songkhla (Ton Nga Chang), Surat Thani (Khlong Saeng WS).

**References.**Hosoishi (2009), Hosoishi and Ogata (2009).

***Crematogaster treubi* Emery, 1896: 246**

*Crematogaster treubi* Emery, 1896: 246.

**Distribution. *Northern***: Phayao (Mae Ka), Lampang (Ngao). ***Western***: Tak (Umphang WS), Kanchanaburi (Mae Klong), Phetchaburi (Kaeng Krachan). ***Northeastern***: Nakhon Ratchasima (Sakaerat). ***Eastern***: Chanthaburi (Khao Soi Dao WS). ***Southern***: Songkhla (Hat Yai), Trang (Khao Chong).

**References.**Hosoishi and Ogata (2012), Hosoishi (2015).

***Dacetinops concinnus* Taylor, 1965**

*Dacetinops concinna* Taylor, 1965: 1, figs 1, 2.

**Distribution. *Southern***: Songkhla (Khao Nam Khang NP).

**References.**Jaitrong and Nabhitabhata (2005), Jaitrong (2011).

***Dilobocondyla fouqueti* Santschi, 1910**

Figs 70, 71

*Dilobocondyla fouqueti* Santschi, 1910: 283.

**Distribution. *Northern***: Chiang Mai (Chiang Mai University Campus), Phrae (Wang Chin Forest Plantation). ***Western***: Kanchanaburi (Thong Pha Phum). ***Eastern***: Chanthaburi (Pheao NP).

**Remarks.** New record.

**Material examined.** N Thailand, Chiang Mai Prov, Chiang Mai Univesity Campus (CMU), 29.VIII.2014, W. Sangtow leg. (THNHM); N Thailand, Phrae Prov, Wang Chin Forest Plantation, 25.III.2002, N. Kongjam leg. (THNHM); W Thailand, Kanchanaburi Prov, Thong Pha Phum Dist, Disturbed forest, 15.X.2004, C. Bourmas leg. (THNHM); E Thailand, Chanthaburi Prov, 26.I.2014, W. Jaitrong leg., WJT260114–GC (THNHM).

***Epelysidris brocha* Bolton, 1987**

Figs 72, 73

*Epelysidris brocha* Bolton, 1987: 280, figs 16, 17.

**Distribution. *Southern***: Narathiwat (Hala–Bala WS).

**Remarks.** New record.

**Material examined.** S Thailand, Narathiwat Prov, Wang Dist, Hala–Bala WS, 24.II.2002, S. Hasin Leg. (AMK, THNHM).

**Figures 72–81.**
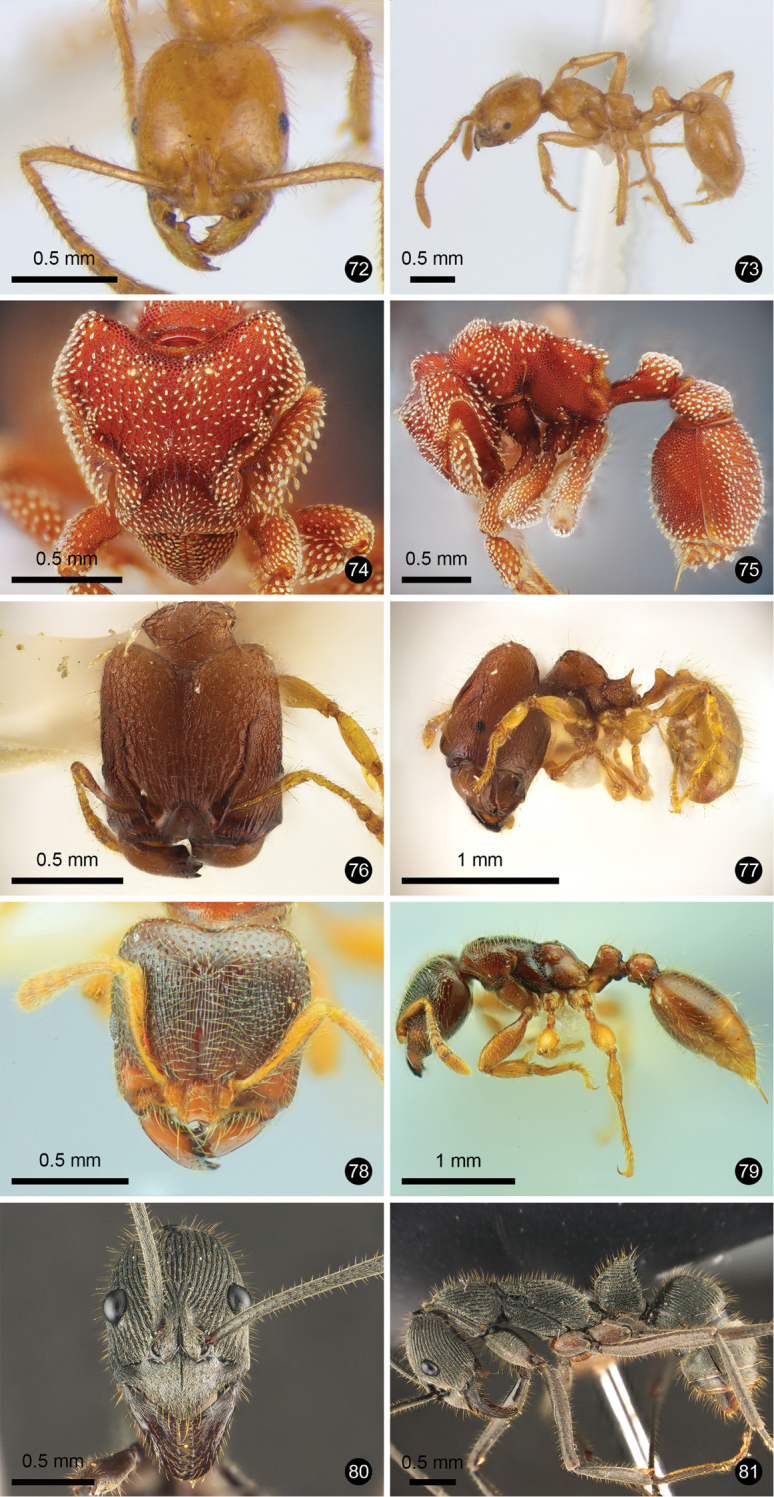
Ant species new to Thailand. **72, 73***Epelysidris brocha***74, 75***Eurhopalothrix heliscata***76, 77***Pheidole nodgii***78, 79***Vollenhovia fridae***80, 81***Diacamma longitudinale*.

***Erromyrma latinodis* (Mayr, 1872)**

*Monomorium latinode* Mayr, 1872: 152.

**Distribution. *Northern***: Lampang (Tham Pha Thai NP). ***Eastern***: Chanthaburi (Khao Soi Dao WS). ***Southern***: Yala.

**References.**Bingham (1906), Jaitrong and Nabhitabhata (2005, cited as *Monomorium latinode* Mayr, 1872).

***Eurhopalothrix heliscata* Wilson & Brown, 1985**

Figs 74, 75

*Eurhopalothrix heliscata* Wilson & Brown, 1985: 410, figs 1–3.

**Distribution. *Southern***: Trang (Khao Chong BG, Ton Tae Waterfall), Satun (Tarutao NP).

**Remarks.** New record.

**Material examined.** S Thailand, Trang Prov, Ton Tae Waterfall, 200–300m, Tropical rainforest, 28.III.2005, W. Jaitrong leg., WJT05–S150 (THNHM); S Thailand, Trang Prov, Khao Chong BG, 16.IV.2002, W. Jaitrong leg., WJT02–S002 (THNHM); S Thailand, Satun Prov, Tarutao NP, 7.III.2007, W. Jaitrong leg., WJT07–TH327 (THNHM).

***Gauromyrmex acanthinus* (Karavaiev, 1935)**

*Solenomyrma acanthina* Karavaiev, 1935: 103, fig. 23.

**Distribution. *Northern***: Chiang Mai (Doi Suthep–Pui NP). ***Western***: Tak (Thung Yai Naresuan East WS). ***Southern***: Krabi (Ko Lanta), Trang (Khao Chong BG).

**References.**Jaitrong and Jeenthong (2014b).

***Kartidris matertera* Bolton, 1991**

*Kartidris matertera* Bolton, 1991: 13, fig. 21.

**Distribution. *Northern***: Chiang Mai (Nong Hoi*). ***Northeastern***: Chaiyaphum (Phu Khiao WS). ***Central***: Phitsanulok (Phu Soi Dao NP).

**References.**Bolton (1991, 1995).

***Lasiomyrma wiwatwitayai* Jaitrong, 2010**

*Lasiomyrma wiwatwitayai* Jaitrong, 2010: 428, figs 1–3.

**Distribution. *Western***: Tak (Umphang WS), Phetchaburi (Kaeng Krachan NP). ***Northeastern***: Nakhon Ratchasima (Khao Yai NP*). ***Eastern***: Chanthaburi (Khao Soi Dao WS*).

**References.**Jaitrong (2010), Jaitrong (2011).

***Liomyrmex gestroi* (Emery, 1887)**

*Laparomyrmex gestroi* Emery, 1887f: 461, pl. 2, fig. 16.

**Distribution. *Western***: Tak (Umphang WS, Thung Yai Naresuan East WS). ***Northeastern***: Nakhon Ratchasima (Pak Chong). ***Eastern***: Chachoengsao (Khao Ang Reu Nai WS).

**References.**Jaitrong and Nabhitabhata (2005), Jaitrong (2011).

***Lophomyrmex bedoti* Emery, 1893**

*Lophomyrmex bedoti* Emery, 1893b: 192, pl. 8, fig. 17.

**Distribution. *Northern***: Chiang Mai (Mae Taeng). ***Western***: Kanchanaburi (Sai Yok NP). ***Eastern***: Chachoengsao (Khao Ang Reu nai WS), Chanthaburi (Pheao NP). ***Southern***: Chumphon (Krom Luang Chumphon WS), Ranong (Khlong Na Kha WS), Surat Thani (Tai Rom Yen NP, Khlong Yan WS), Nakhon Si Thammarat (Khao Nan, Khao Luang NP), Krabi (Ko Lanta), Phatthalung (Khao Pappha), Trang (Khao Chong BG), Satun (Tarutao NP), Narathiwat (Hala–Bala WS), Songkhla (Hat Yai, Ton Nga Chang).

**References.**Jaitrong and Nabhitabhata (2005).

***Lophomyrmex birmanus* Emery, 1893**

*Lophomyrmex birmanus* Emery, 1893b: 192

**Distribution. *Northern***: Chiang Mai (Khun Chang Khian, Pa Miang Village, Doi Suthep–Pui NP, Mae Chaem, Mae Taeng, Doi Chiang Dao, Doi Luang). ***Western***: Tak (Thung Yai Naresuan East WS, Umphang WS), Kanchanaburi (Thong Pha Phum NP), Phetchaburi (Kaeng Krachan). ***Northeastern***: Chaiyaphum (Phu Khiao WS), Nakhon Ratchasima (Khao Yai NP, Sakaerat). ***Central***: Uthai Thani (Huai Kha Khaeng WS).

**References.**Jaitrong and Nabhitabhata (2005), Sakchoowong et al. (2008), Sakchoowong et al. (2009), Onishi et al. (2016).

***Lophomyrmex lucidus* Menozzi, 1930**

Lophomyrmex bedoti var. lucida Menozzi, 1930: 328.

**Distribution. *Northern***: Chiang Mai (Mae Chaem). ***Western***: Kanchanaburi (Sai Yok NP). ***Northeastern***: Chaiyaphum (Phu Khiao WS), Loei (Phu Luang WS) ***Southern***: Trang (Khao Chong BG), Songkhla (Hat Yai, Khao Nam Khang NP, Sadao), Surat Thani (Tai Rom Yen NP), Ranong (Suk Samran), Naratiwat (Hala–Bala WS).

**References.**Jaitrong and Nabhitabhata (2005).

***Lophomyrmex striatulus* Rigato, 1994**

*Lophomyrmex striatulus* Rigato, 1994: 56, figs 16, 17.

**Distribution. *Eastern***: Chanthaburi (Pheao NP*, Khao Soi Dao WS, Namtok Phlio NP), Chacheongsao (Khao Ang Reu Nai WS).

**References.**Rigato (1994), Jaitrong and Nabhitabhata (2005).

***Mayriella transfuga* Baroni Urbani, 1977**

*Mayriella transfuga* Baroni Urbani, 1977b: 411, figs 1, 2.

**Distribution. *Northeastern***: Nakhon Ratchasima (Khao Yai NP). ***Eastern***: Chanthaburi (Pheao NP [Khao Sabap]).

**References.** Shattuck and Barne**t**t (2007).

***Meranoplus bicolor* (Guérin–Méneville, 1844)**

*Cryptocerus bicolor* Guérin–Méneville, 1844: 425.

**Distribution. *Northern***: Chiang Rai (Doi Tung), Chiang Mai (Doi Ang Khang, Doi Luang Chiang Dao, Chiang Dao WS, Mae Taeng, Doi Suthep–Pui NP, Chiang Mai University Campus, Doi Inthanon NP, Mae Chaem), Phayao (Mae Ka), Lamphun (Mae Li Forest Plantation), Lampang (Haui Tak, Tham Pha Thai NP), Phrae (Wang Chin Forest Plantation), Nan (Doi Phu Kha NP, Nakhon Nan Forest Plantation). ***Western***: Tak (Umphang WS, Thung Yai Naresuan East WS, Lansang NP, Taksin Maharat NP), Kanchanaburi (Thong Pha Phum NP, Pha Tad Watershed Management station), Phetchaburi (Kaeng Krachan NP), Prachuap Khiri Khan (Kui Buri). ***Northeastern***: Kalasin (Phu Sithan WS), Chaiyaphum (Phu Khiao WS), Loei (Phu Luang WS), Nakhon Ratchasima (Sakaerat, Khao Yai NP, Buer Yai, Pak Chong), Ubon Ratchathani (Pha Taem NP). ***Central***: Uthai Thani (Huai Kha Khaeng WS), Saraburi (Phukae BG), Bangkok (Bang Khen, Chatuchak), Pathum Thani (Khlong Luang), Samut Prakan (Bang Krachao), Samut Songkhram (Mueang Samut Songkhram). ***Eastern***: Sa Kaeo (Khao Ang Reu Nai WS), Chachoengsao (Khao Ang Reu Nai WS), Chon Buri (Si Racha, Khao Kheow, Ko Samaesarn), Chanthaburi (Khao Soi Dao WS, Pheao NP, Khao Ang Reu Nai WS), Rayong (Ko Man Nai, Khao Ang Reu Nai WS). ***Southern***: Chumphon (Krom Luang Chumphon NP), Ranong (Suk Samran, Khlong Na Kha WS), Surat Thani (Tai Rom Yen NP, Khlong Yan WS, Mu Ko Ang Thong NP), Nakhon Si Thammarat (Khao Nan NP, Khao Luang NP, Krung Ching Waterfall), Krabi (Ko Lanta, Had Nopparat Thara, Ao Nang), Phang–nga (Khao Lak), Trang (Khao Chong BG, Thung Khai BG, Palian), Phatthalung (Khao Pu–Khao Ya NP, Khao Bantad WS), Satun (Tarutao NP), Songkhla (Khao Kho Hong, Khao Nam Khang NP), Yala, Pattani (Yaring), Narathiwat (Hala–Bala WS).

**References.**Bingham (1906), Schödl (1998), Jaitrong and Nabhitabhata (2005), Jaitrong and Jeenthong (2014a), Suwannaphak et al. (2016).

***Meranoplus castaneus* Smith, 1857**

*Meranoplus castaneus* Smith, 1857: 81, pl. 2, fig. 7.

**Distribution. *Southern***: Nakhon Si Thammarat (Peom Lok Waterfall), Trang (Khao Chong BG, Thung Khai BG), Songkhla (Khao Kho Hong, Ton Nga Chang WS), Narathiwat (Hala–Bala WS, Toh Daeng).

**References.**Schödl (1998), Tongjerm et al. (2003), Jaitrong and Nabhitabhata (2005).

***Meranoplus laeviventris* Emery, 1889**

*Meranoplus laeviventris* Emery, 1889a: 506, pl. 10, fig. 16.

**Distribution. *Northern***: Chiang Mai (Khun Chang Khian, Doi Suthep–Pui NP, Doi Ang Khang, Omkoi, Doi Pha Hom Pok NP, Mae Chaem), Nan (Doi Phu Kha NP). ***Western***: Tak (Thung Yai Naresuan East WS). ***Central***: Uthai Thani (Huai Kha Khaeng WS).

**References.**Schödl (1998), Jaitrong and Nabhitabhata (2005), Onishi et al. (2016).

***Meranoplus mucronatus* Smith, 1857**

*Meranoplus mucronatus* Smith, 1857: 82, pl. 2, fig. 6.

**Distribution. *Southern***: Narathiwat (Hala–Bala WS).

**References.**Schödl (1998), Jaitrong and Nabhitabhata (2005).

***Monomorium chinense* Santschi, 1925**

Monomorium minutum var. chinensis Santschi, 1925: 86.

**Distribution. *Northern***: Chiang Rai (Doi Tung), Chiang Mai (Doi Ang Khang, Doi Luang Chiang Dao, Chiang Dao WS, Mae Taeng, Doi Suthep–Pui NP, Chiang Mai University Campus, Doi Inthanon NP, Mae Chaem), Phayao (Mae Ka), Lamphun (Mae Li Forest Plantation), Lampang (Haui Tak, Tham Pha Thai NP), Phrae (Wang Chin Forest Plantation), Nan (Doi Phu Kha NP, Nakhon Nan Forest Plantation). ***Western***: Tak (Umphang WS, Thung Yai Naresuan East WS, Lansang NP, Taksin Maharat NP), Kanchanaburi (Thong Pha Phum NP, Pha Tad Watershed Management station), Phetchaburi (Kaeng Krachan NP). ***Northeastern***: Kalasin (Phu Sithan WS), Chaiyaphum (Phu Khiao WS), Loei (Phu Luang WS), Nakhon Ratchasima (Sakaerat, Khao Yai NP, Buer Yai), Ubon Ratchathani (Pha Taem NP). ***Central***: Uthai Thani (Huai Kha Khaeng WS), Saraburi (Phukae BG), Bangkok (Bang Khen, Chatuchak), Pathum Thani (Khlong Luang), Samut Prakan (Bang Krachao). ***Eastern***: Sa Kaeo (Khao Ang Reu Nai WS), Chachoengsao (Khao Ang Reu Nai WS), Chon Buri (Si Racha, Khao Kheow), Chanthaburi (Khao Soi Dao WS, Pheao NP, Khao Ang Reu Nai WS), Rayong (Ko Man Nai, Khao Ang Reu Nai WS), Trat (Ko Kut). ***Southern***: Chumphon (Krom Luang Chumphon NP), Ranong (Suk Samran, Khlong Na Kha WS), Surat Thani (Khlong Yan WS, Mu Ko Ang Thong NP), Nakhon Si Thammarat (Khao Nan NP, Khao Luang NP, Krung Ching Waterfall), Krabi (Ko Lanta), Trang (Khao Chong BG, Thung Khai BG, Palian), Phatthalung (Khao Pu–Khao Ya NP, Khao Bantad WS), Satun (Tarutao NP), Songkhla (Khao Kho Hong, Khao Nam Khang NP), Pattani (Yaring), Narathiwat (Hala–Bala WS).

**References.**Jaitrong and Nabhitabhata (2005), Sakchoowong et al. (2008), Suwannaphak et al. (2016).

***Monomorium floricola* (Jerdon, 1851)**

*Atta floricola* Jerdon, 1851: 107.

**Distribution. *Northern***: Chiang Rai (Doi Tung), Chiang Mai (Doi Ang Khang, Doi Luang Chiang Dao, Chiang Dao WS, Mae Taeng, Doi Suthep–Pui NP, Chiang Mai University Campus, Doi Inthanon NP, Mae Chaem), Phayao (Mae Ka), Lamphun (Mae Li Forest Plantation), Lampang (Haui Tak, Tham Pha Thai NP), Phrae (Wang Chin Forest Plantation), Nan (Doi Phu Kha NP, Nakhon Nan Forest Plantation). ***Western***: Tak (Umphang WS, Thung Yai Naresuan East WS, Lansang NP, Taksin Maharat NP), Kanchanaburi (Thong Pha Phum NP, Pha Tad Watershed Management station), Phetchaburi (Kaeng Krachan NP), Prachuap Khiri Khan (Kui Buri). ***Northeastern***: Kalasin (Phu Sithan WS), Chaiyaphum (Phu Khiao WS), Loei (Phu Luang WS), Nakhon Ratchasima (Sakaerat, Khao Yai NP, Buer Yai), Ubon Ratchathani (Pha Taem NP). ***Central***: Uthai Thani (Huai Kha Khaeng WS), Saraburi (Phukae BG), Bangkok (Bang Khen, Chatuchak), Pathum Thani (Khlong Luang), Samut Prakan (Bang Krachao), Samut Songkhram (Mueang Samut Songkhram). ***Eastern***: Sa Kaeo (Khao Ang Reu Nai WS), Chachoengsao (Khao Ang Reu Nai WS), Chon Buri (Si Racha, Khao Kheow, Ko Samaesarn), Chanthaburi (Khao Soi Dao WS, Pheao NP, Khao Ang Reu Nai WS), Rayong (Ko Man Nai, Khao Ang Reu Nai WS), Trat (Ko Kut). ***Southern***: Chumphon (Krom Luang Chumphon NP), Ranong (Suk Samran, Khlong Na Kha WS), Surat Thani (Tai Rom Yen NP, Khlong Yan WS, Mu Ko Ang Thong NP), Nakhon Si Thammarat (Khao Nan NP, Khao Luang NP, Krung Ching Waterfall), Krabi (Ko Lanta), Trang (Khao Chong BG, Thung Khai BG, Palian), Phatthalung (Khao Pu–Khao Ya NP, Khao Bantad WS), Satun (Tarutao NP), Songkhla (Khao Kho Hong, Khao Nam Khang NP), Pattani (Yaring), Narathiwat (Hala–Bala WS).

**References.**Jaitrong and Nabhitabhata (2005), Sakchoowong et al. (2009), Jaitrong and Jeenthong (2014a), Suwannaphak et al. (2016).

***Monomorium pharaonis* (Linnaeus, 1758)**

*Formica pharaonis* Linnaeus, 1758: 580.

**Distribution. *Northern***: Chiang Rai (Doi Tung), Chiang Mai (Doi Ang Khang, Doi Luang Chiang Dao, Chiang Dao WS, Mae Taeng, Doi Suthep–Pui NP, Chiang Mai University Campus, Doi Inthanon NP, Mae Chaem), Phayao (Mae Ka), Lamphun (Mae Li Forest Plantation), Lampang (Haui Tak, Tham Pha Thai NP), Phrae (Wang Chin Forest Plantation), Nan (Doi Phu Kha NP, Nakhon Nan Forest Plantation). ***Western***: Tak (Umphang WS, Thung Yai Naresuan East WS, Lansang NP, Taksin Maharat NP), Kanchanaburi (Thong Pha Phum NP, Pha Tad Watershed Management station), Phetchaburi (Kaeng Krachan NP), Prachuap Khiri Khan (Kui Buri). ***Northeastern***: Kalasin (Phu Sithan WS), Chaiyaphum (Phu Khiao WS), Loei (Phu Luang WS), Nakhon Ratchasima (Sakaerat, Khao Yai NP, Buer Yai), Ubon Ratchathani (Pha Taem NP), Samut Prakan (Bang Krachao). ***Central***: Uthai Thani (Huai Kha Khaeng WS), Saraburi (Phukae BG), Bangkok (Bang Khen, Chatuchak), Pathum Thani (Khlong Luang), Samut Songkhram (Mueang Samut Songkhram). ***Eastern***: Sa Kaeo (Khao Ang Reu Nai WS), Chachoengsao (Khao Ang Reu Nai WS), Chon Buri (Si Racha, Khao Kheow, Ko Samaesarn), Chanthaburi (Khao Soi Dao WS, Pheao NP, Khao Ang Reu Nai WS, Kung Krabaen), Rayong (Ko Man Nai, Khao Ang Reu Nai WS), Trat (Ko Kut). ***Southern***: Chumphon (Krom Luang Chumphon NP), Ranong (Suk Samran, Khlong Na Kha WS), Surat Thani (Tai Rom Yen NP, Khlong Yan WS, Mu Ko Ang Thong NP), Nakhon Si Thammarat (Khao Nan NP, Khao Luang NP, Krung Ching Waterfall), Krabi (Ko Lanta), Trang (Khao Chong BG, Thung Khai BG, Palian), Phatthalung (Khao Pu–Khao Ya NP, Khao Bantad WS), Satun (Tarutao NP), Songkhla (Khao Kho Hong, Khao Nam Khang NP), Pattani (Yaring), Narathiwat (Hala–Bala WS).

**References.**Jaitrong and Nabhitabhata (2005), Sakchoowong et al. (2009), Jaitrong and Jeenthong (2014a), Suwannaphak et al. (2016).

***Myrmecina asiatica* Okido, Ogata & Hosoishi, 2020**

*Myrmecina asiatica* Okido, Ogata & Hosoishi, 2020: 17, fig. 4.

**Distribution. *Northern***: Chiang Mai. ***Eastern***: Chanthanburi. ***Westhern***: Phetchaburi (Kaeng Krachan NP). ***Northeastern***: Nakhon Ratchasima. ***Sotuhern***: Pattani.

**References.**Okido et al. (2020).

***Myrmecina dechai* Okido, Ogata & Hosoishi, 2020**

*Myrmecina dechai* Okido, Ogata & Hosoishi, 2020: 34, fig. 13.

**Distribution. Westhern**: Phetchaburi (Kaeng Krachan NP).

**References.**Okido et al. (2020).

***Myrmecina inflata* Okido, Ogata & Hosoishi, 2020**

*Myrmecina inflata* Okido, Ogata & Hosoishi, 2020: 51, fig. 22.

**Distribution. Sotuhern**: Phang Nga

**References.**Okido et al. (2020).

***Myrmecina inthanonensis* Okido, Ogata & Hosoishi, 2020**

*Myrmecina inthanonensis* Okido, Ogata & Hosoishi, 2020: 55, fig. 24.

**Distribution. *Northern***: Chiang Mai

**References.**Okido et al. (2020).

***Myrmecina maryatiae* Okido, Ogata & Hosoishi, 2020**

*Myrmecina maryatiae* Okido, Ogata & Hosoishi, 2020: 70, fig. 32.

**Distribution. *Sotuhern***: Phang Nga.

**References.**Okido et al. (2020).

***Myrmecina raviwonghei* Jaitrong, Samung, Waengsothorn & Okido, 2019**

*Myrmecina raviwonghei*Jaitrong et al., 2019a: 3, figs 1–5.

**Distribution. *Western***: Tak (Thung Yai Naresuan East WS), Kanchanaburi (Thong Pha Phum). ***Northeastern***: Nakhon Ratchasima (Sakaerat*).

**References.**Jaitrong et al. (2019a).

***Myrmica ritae* Emery, 1889**

*Myrmica ritae* Emery, 1889a: 501, pl. 11, fig. 27.

**Distribution. *Northern***: Chiang Mai (Doi Inthanon NP). ***Western***: Tak (Umphang WS).

**References.**Radchenko and Elmes (1998, 1999), Jaitrong and Nabhitabhata (2005), Jaitrong (2011).

***Myrmicaria arachnoides lutea* Emery, 1900**

Myrmicaria arachnoides var. lutea Emery, 1900b: 692.

**Distribution. *Eastern***: Chachoengsao (Khao Ang Reu Nai WS). ***Southern***: Krabi (Ko Lanta).

**References.**Bakhtiar et al. (2009), Jaitrong and Jeenthong (2014, cited as *Myrmicaria luteiventris* Emery, 1900).

***Myrmicaria birmana* Forel, 1902**

Myrmicaria arachnoides r. birmana Forel, 1902b: 243.

**Distribution. *Northeastern***: Nakhon Ratchasima (Khao Yai NP). ***Eastern***: Chachoengsao (Khao Ang Reu Nai WS).

**References.**Jaitrong and Nabhitabhata (2005).

***Myrmicaria brunnea* Saunders, 1842**

*Myrmicaria brunnea* Saunders, 1842: 57, pl. 5, fig. 2.

**Distribution. *Northern***: Chiang Mai (Doi Ang Khang, Ob Luang Chiang Dao WS, Mae Taeng, Doi Suthep–Pui NP, Mae Chaem), Phrae (Wang Chin). ***Western***: Tak (Thung Yai Naresuan East WS, Umphang WS). ***Northeastern***: Chaiyaphum (Phu Khiao WS), Nakhon Ratchasima (Sakaerat, Khao Yai NP). ***Central***: Phitsanulok (Thung Salaeng Luang NP). ***Eastern***: Chachoengsao (Khao Ang Reu Nai WS), Chanthaburi (Khao Soi Dao WS). ***Southern***: Ranong (Suk Samran, Khlong Na Kha WS), Surat Thani (Tai Rom Yen NP, Khlong Yan WS), Nakhon Si Thammarat (Tapi Watershed Research Station, Khao Luang NP), Phatthalung (Khao Pappha), Trang (Khao Chong BG), Yala, Narathiwat (Hala–Bala WS).

**References.**Bingham (1906), Jaitrong and Nabhitabhata (2005).

***Myrmicaria vidua* Smith, 1858**

*Myrmicaria vidua* Smith, 1858: 141.

**Distribution. *Northern***: Chiang Mai (Doi Inthanon NP).

**References.**Bakhtiar et al. (2009).

***Paratopula macta* Bolton, 1988**

*Paratopula macta* Bolton, 1988: 140, fig. 2.

**Distribution. *Northeastern***: Nakhon Ratchasima (Khao Yai NP, Sakaerat). ***Central***: Bangkok (Kasetsart University). ***Eastern***: Chachoengsao (Khao Ang Reu Nai WS), Chanthaburi (Khao Ang Reu Nai WS, Khao Soi Dao WS), Trat (Agoforestry Research Station).

**References.**Luo and Guénard (2016), Jaitrong and Nabhitabhata (2005), Jaitrong (2011).

***Pheidole aglae* Forel, 1913**

*Pheidole aglae* Forel, 1913: 32.

**Distribution. *Southern***: Narathiwat (Hala–Bala WS).

**References.**Jaitrong and Nabhitabhata (2005).

***Pheidole annexa* Eguchi, 2001**

*Pheidole annexus* Eguchi, 2001: 32, fig. 6.

**Distribution. *Southern***: Narathiwat (Hala–Bala WS).

**References.**Jaitrong and Nabhitabhata (2005).

***Pheidole aristotelis* Forel, 1911**

*Pheidole aristotelis* Forel, 1911a: 43.

**Distribution. *Western***: Tak (Thung Yai Naresuan East WS), Prachuap Khiri Khan (Kaeng Krachan NP). ***Southern***: Ranong (Khlong Na Kha WS), Nakhon Si Thammarat (Tapi Watershed Research Station, Khao Nan NP), Trang (Khao Chong BG), Narathiwat (Hala–Bala WS).

**References.**Jaitrong and Nabhitabhata (2005).

***Pheidole binghamii* Forel, 1902**

*Pheidole binghamii* Forel, 1902b: 184.

**Distribution. *Northeastern***: Nakhon Ratchasima (Sakaerat Environmental Research Station).

**References.**Antweb (2019).

***Pheidole bluntschlii* Forel, 1911**

Pheidole (Ceratopheidole) bluntschlii Forel, 1911b: 373.

**Distribution. *Southern***: Narathiwat (Hala–Bala WS).

**References.**Jaitrong and Nabhitabhata (2005).

***Pheidole butteli* Forel, 1913**

*Pheidole butteli* Forel, 1913: 36.

**Distribution. *Southern***: Trang (Khao Chong BG).

**References.**Jaitrong and Nabhitabhata (2005).

***Pheidole capellinii* Emery, 1887**

*Pheidole capellinii* Emery, 1887e: 463.

**Distribution. *Northern***: Chiang Mai (Mae Taeng, Mae Chaem, Doi Suthep–Pui NP, Omkoi) ***Western***: Tak (Thung Yai Naresuan East WS, Umphang WS), Kanchanaburi (Thong Pha Phum NP). ***Northeastern***: Chaiyaphum (Phu Khiao WS), Nakhon Ratchasima (Khao Yai NP). ***Southern***: Trang (Palian), Songkhla (Ton Nga Chang WS).

**References.**Jaitrong and Nabhitabhata (2005), Eguchi (2008), Sakchoowong et al. (2008), Sakchoowong et al. (2009).

***Pheidole cariniceps* Eguchi, 2001**

*Pheidole cariniceps* Eguchi, 2001: 41, fig. 10.

**Distribution. *Southern***: Narathiwat (Hala–Bala WS).

**References.**Jaitrong and Nabhitabhata (2005).

***Pheidole clypeocornis* Eguchi, 2001**

*Pheidole clypeocornis* Eguchi, 2001: 44, fig. 11.

**Distribution. *Southern***: Ranong (Khlong Na Kha WS), Nakhon Si Thammarat (Khao Nan NP), Trang (Khao Chong BG), Narathiwat (Hala–Bala WS).

**References.**Jaitrong and Nabhitabhata (2005).

***Pheidole comata* Smith, 1858**

*Pheidole comata* Smith, 1858: 176.

**Distribution. *Northern***: Chiang Mai (Chiang Dao WS). ***Western***: Tak (Thung Yai Naresuan East WS). ***Northeastern***: Nakhon Ratchasima (Khao Yai NP). ***Eastern***: Chanthaburi (Khao Soi Dao WS).

**References.**Jaitrong and Nabhitabhata (2005).

***Pheidole dugasi* Forel, 1911**

*Pheidole dugasi* Forel, 1911g: 222.

**Distribution. *Northern***: Chiang Mai (Chiang Mai University, Chiang Dao WS). ***Western***: Tak (Thung Yai Naresuan East WS, Umphang WS), Kanchanaburi (Thong Pha Phum NP). ***Northeastern***: Kalasin (Phu Sithan WS), Nakhon Ratchasima (Khao Yai NP). ***Central***: Phechabun (Bueng Sam Pan). ***Eastern***: Chachoengsao (Khao Ang Reu Nai WS), Chanthaburi (Pheao NP, Khao Soi Dao WS).

**References.**Jaitrong and Nabhitabhata (2005), Eguchi (2008), Sakchoowong et al. (2009).

***Pheidole elongicephala* Eguchi, 2008**

*Pheidole elongicephala* Eguchi, 2008: 20, fig. 4a–h.

**Distribution. *Western***: Tak (Thung Yai Naresuan East WS, Umphang WS).

**Remarks.** New record.

**Material examined.** W Thailand, Tak Prov, Umphang Dist, Umphang WS, Mae Khlong Ki Forest Ranger Station, Hill Evergreen Forest (HEF), 25.I.2015, W. Jaitrong leg., TH15–WJT–228 (THNHM); W Thailand, Tak Prov, Umphang Dist, Umphang WS, Pha Luard Forest Ranger Station, Mixed Deciduous Forest (MEF), 28.I.2015, W. Jaitrong leg., TH15–WJT–239 (THNHM); W Thailand, Tak Prov, Umphang Dist, Umphang WS, Doi Huar Mod Forest Ranger Station, Dry Dipterocarp Forest (DDF), 27.I.2015, W. Jaitrong leg., TH15–WJT–253 (THNHM).

***Pheidole elisae* Emery, 1900**

*Pheidole elisae* Emery, 1900a: 686.

**Distribution. *Southern***: Nakhon Si Thammarat (Khao Nan NP, Khao Luang NP), Trang (Khao Chong BG), Phatthalung (Khao Pu–Khao Ya NP).

**References.**Jaitrong and Nabhitabhata (2005).

***Pheidole fervens* Smith, 1858**

*Pheidole fervens* Smith, 1858: 176.

**Distribution. *Western***: Prachuap Khiri Khan (Pala–U Waterfall). ***Central***: Bangkok (Kasetsart University). ***Eastern***: Chachoengsao (Khao Ang Reu Nai WS), Chanthaburi (Khao Soi Dao WS), Rayong (Na Yai Arm). ***Southern***: Krabi (Ko Lanta), Trang (Khao Chong BG, Palian).

**References.**Jaitrong and Nabhitabhata (2005), Eguchi (2008), Jaitrong and Jeenthong (2014a).

***Pheidole fortis* Eguchi, 2006**

*Pheidole fortis* Eguchi, 2006: 118, fig. 2A–I.

**Distribution. *Northern***: Chiang Mai (Doi Suthep–Pui NP), Nan (Doi Phu Kha NP).

**References.**Eguchi (2006), Eguchi (2008).

***Pheidole gatesi* (Wheeler, 1927)**

Aphaenogaster (Attomyrma) gatesi Wheeler, 1927b: 44.

**Distribution. *Northern***: Chiang Rai (Mae Fa Luang), Chiang Mai (Doi Suthep–Pui NP, Doi Ang Khang, Ob Luang NP, Doi Inthanon NP, Omkoi), Nan (Doi Phu Kha NP). ***Western***: Tak (Thung Yai Naresuan East WS, Umphang WS).

**References.**Jaitrong and Nabhitabhata (2005), Eguchi (2008), Eguchi et al. (2020).

***Pheidole hongkongensis* Wheeler, 1928**

Pheidole rinae subsp. hongkongensis Wheeler, 1928c: 11.

**Distribution. *Northern***: Chiang Mai (Mae Chaem). ***Northeastern***: Kalasin (Phu Sithan WS), Nakhon Ratchasima (Sakaerat). ***Eastern***: Chachoengsao (Khao Ang Reu Nai WS), Rayong (Mu Ko Man).

**References.**Jaitrong and Nabhitabhata (2005), Eguchi (2008).

***Pheidole hortensis* Forel, 1913**

*Pheidole hortensis* Forel, 1913: 38, fig. J.

**Distribution. *Northeastern***: Nakhon Ratchasima (Khao Yai NP, Sakaerat). ***Eastern***: Chachoengsao (Khao Ang Reu Nai WS), Chanthaburi (Khao Soi Dao WS, Khao Khitchakut NP). ***Southern***: Ranong (Khlong Na Kha WS), Nakhon Si Thammarat (Khao Nan NP), Trang (Ton Tae Waterfall, Khao Chong BG).

**References.**Jaitrong and Nabhitabhata (2005).

***Pheidole huberi* Forel, 1911**

*Pheidole huberi* Forel, 1911b: 374.

**Distribution. *Western***: Tak (Thung Yai Naresuan East WS), Phetchaburi (Kaeng Krachan NP). ***Southern***: Chumphon (Krom Luang Chumphon WS), Phatthalung (Khao Pu–Khao Ya NP), Songkhla (Ton Nga Chang WS, Prince of Songkhla University).

**References.**Watanasit and Noon–anant (2005), Watanasit et al. (2007).

***Pheidole inornata* Eguchi, 2001**

*Pheidole inornata* Eguchi, 2001: 66, fig. 22.

**Distribution. *Western***: Tak (Thung Yai Naresuan East WS), Kanchanaburi (Thong Pha Phum NP). ***Northeastern***: Mukdahan (Phu Sithan WS), Nakhon Ratchasima (Khao Yai NP, Sakaerat). ***Central***: Uthai Thani (Ban Rai). ***Eastern***: Sa Kaeo (Pang Sida NP), Chon Buri (Si Racha), Chachoengsao (Khao Ang Reu Nai WS), Chanthaburi (Pheao NP, Khao Soi Dao WS), Rayong (Mu Ko Man). ***Southern***: Ranong (Khlong Na Kha WS), Surat Thani (Mu Ko Ang Thong), Nakhon Si Thammarat (Khao Luang NP), Krabi (Ko Lanta), Trang (Ton Tae Waterfall, Khao Chong BG), Satun (Kok Adunk), Narathiwat (Hala–Bala WS).

**References.**Jaitrong and Nabhitabhata (2005), Jaitrong and Jeenthong (2014a).

***Pheidole inscrobiculata* Viehmeyer, 1916**

*Pheidole inscrobiculatus* Viehmeyer, 1916: 120.

**Distribution. *Southern***: Narathiwat (Hala–Bala WS).

**References.**Jaitrong and Nabhitabhata (2005).

***Pheidole jacobsoni* Forel, 1911**

Pheidole javana subsp. jacobsoni Forel, 1911e: 203.

**Distribution. *Eastern***: Chanthaburi (Pheao NP)

**References.**Jaitrong and Nabhitabhata (2005).

***Pheidole rugifera* Eguchi, 2001**

*Pheidole rugifera* Eguchi, 2001: 106, fig. 43.

**Distribution. *Southern***: Satun (Tarutao NP); Songkhla (Ton Nga Chang WS).

**References.**Watanasit et al. (2003), Watanasit et al. (2005).

***Pheidole magrettii* Emery, 1887**

*Pheidole magrettii* Emery, 1887e: 462.

**Distribution. *Southern***: Ranong (Khlong Na Kha WS).

**References.**Jaitrong and Nabhitabhata (2005).

***Pheidole megacephala* (Fabricius, 1793)**

*Formica megacephala* Fabricius, 1793: 36.

**Distribution. *Central***: Samut Prakan (Bang Krachao). ***Southern***: Krabi (Ko Lanta), Trang (Khao Chong BG).

**References.**Jaitrong and Nabhitabhata (2005), Eguchi (2008), Jaitrong and Jeenthong (2014a).

***Pheidole nodgii* Forel, 1905**

Figs 76, 77

*Pheidole nodgii* Forel, 1905: 16.

**Distribution. *Eastern***: Chachoengsao (Khao Ang Reu Nai WS), Chanthaburi (Khao Khitchakut NP). ***Southern***: Phatthalung (Khao Pu–Khao Ya NP).

**Remarks.** New record.

**Material examined.** E Thailand, Chachoengsao Prov Khao Ang Reu Nai WS, Bo Thong Station, 30.XII.2002, W. Jaitrong leg. (THNHM); E Thailand, Chachoengsao Prov Khao Ang Reu Nai WS, Lumchangwat Station, 22.VIII.2003, W. Jaitrong leg., WJT03–TH–258 (THNHM); E Thailand, Chanthaburi Prov, Khao Khitchakut NP, 19.I.2006, Watana leg., WJT06–E1005 (THNHM); same loc. and collector, 26.XI.2006 (THNHM); S Thailand, Phatthalung Prov, Khao Pu–Khao Ya NP, 28.IX.2007, WJT07–TH2030 (THNHM).

***Pheidole nodifera* (Smith, 1858)**

*Atta nodifera* Smith, 1858: 165.

**Distribution.** Northern: Chiang Mai (Mae Taeng, Doi Luang). Eastern: Chachoengsao (Khao Ang Reu Nai WS). Southern: Narathiwat (Hala–Bala WS).

**References.**Jaitrong and Nabhitabhata (2005).

***Pheidole nodus* Smith, 1874**

*Pheidole nodus* Smith, 1874: 407.

**Distribution. *Northern***: Chiang Mai (Mae Taeng).

**References.**Jaitrong and Nabhitabhata (2005).

***Pheidole parva* Mayr, 1865**

*Pheidole parva* Mayr, 1865: 98.

**Distribution. *Western***: Kanchanaburi (Thong Pha Phum). ***Central***: Bangkok (Bang Khen), Pathum Thani (Khlong Luang). ***Southern***: Narathiwat (Hala–Bala WS).

**References.**Jaitrong and Nabhitabhata (2005, cited as *Pheidole bugi* Wheeler, 1919, junior synonym of *P. parva*), Eguchi (2008).

***Pheidole pieli* Santschi, 1925**

*Pheidole pieli* Santschi, 1925: 83.

**Distribution. *Northern***: Chiang Mai (Chiang Mai University, Doi Suthep–Pui NP). ***Western***: Tak (Umphang WS, Thung Yai Naresuan East WS), Kanchanaburi (Khuean Srinagarindra NP, Thong Pha Phum NP). ***Northeastern***: Nakhon Ratchasima (Khao Yai NP, Sakaerat). ***Eastern***: Chon Buri (Khao Ang Reu Nai WS), Chachoengsao (Khao Ang Reu Nai WS), Rayong (Mu Ko Man), Chanthaburi (Khao Soi Dao WS). ***Southern***: Surat Thani (Mu Ko Ang Thong), Nakhon Si Thammarat (Tapi Watershed Research Station, Khao Nan NP), Krabi (Ko Lanta), Trang (Thung Khai BG), Songkhla (Khao Kho Hong).

**References.**Jaitrong and Nabhitabhata (2005, cited as *Pheidole incense* Wheeler, 1928, junior synonym of *P. pieli*), Eguchi (2008), Sakchoowong et al. (2009), Jaitrong and Jeenthong (2014a).

***Pheidole plagiaria* Smith, 1860**

*Pheidole plagiaria* Smith, 1860a: 112.

**Distribution. *Northern***: Chiang Rai (Doi Tung), Chiang Mai (Doi Ang Khang, Doi Luang Chiang Dao, Chiang Dao WS, Mae Taeng, Doi Suthep–Pui NP, Chiang Mai University Campus, Doi Inthanon NP, Mae Chaem, Omkoi, Doi Pha Hom Pok NP), Phayao (Mae Ka), Lamphun (Mae Li Forest Plantation), Lampang (Haui Tak, Tham Pha Thai NP), Phrae (Wang Chin Forest Plantation), Nan (Doi Phu Kha NP, Nakhon Nan Forest Plantation). ***Western***: Tak (Umphang WS, Thung Yai Naresuan East WS, Lansang NP, Taksin Maharat NP), Kanchanaburi (Thong Pha Phum NP, Pha Tad Watershed Management station), Phetchaburi (Kaeng Krachan NP). ***Northeastern***: Kalasin (Phu Sithan WS), Chaiyaphum (Phu Khiao WS), Loei (Phu Luang WS), Nakhon Ratchasima (Sakaerat, Khao Yai NP, Buer Yai), Ubon Ratchathani (Pha Taem NP). ***Central***: Uthai Thani (Huai Kha Khaeng WS), Saraburi (Phukae BG), Bangkok (Bang Khen, Chatuchak) ***Eastern***: Sa Kaeo (Khao Ang Reu Nai WS), Chachoengsao (Khao Ang Reu Nai WS), Chon Buri (Si Racha, Khao Kheow), Chanthaburi (Khao Soi Dao WS, Pheao NP, Khao Ang Reu Nai WS), Rayong (Ko Man Nai, Khao Ang Reu Nai WS), Trat (Trat Agoforestry Research Station, Ko Kut). ***Southern***: Chumphon (Krom Luang Chumphon NP), Ranong (Suk Samran, Khlong Na Kha WS), Surat Thani (Tai Rom Yen NP, Khlong Yan WS, Mu Ko Ang Thong NP, Khlong Saeng WS), Nakhon Si Thammarat (Khao Nan NP, Khao Luang NP, Krung Ching Waterfall), Phuket (Thalang), Krabi (Ko Lanta), Trang (Khao Chong BG, Thung Khai BG, Palian, Ton Tae Waterfall), Phatthalung (Khao Pu–Khao Ya NP, Khao Bantad WS), Satun (Tarutao NP), Songkhla (Khao Kho Hong, Khao Nam Khang NP), Pattani (Namtok Sai Khao NP), Narathiwat (Hala–Bala WS).

**References.**Jaitrong and Nabhitabhata (2005), Eguchi (2008), Sakchoowong et al. (2008), Jaitrong and Jeenthong (2014a), Eguchi et al. (2020).

***Pheidole planifrons* Santschi, 1920**

*Pheidole planifrons* Santschi, 1920: 166, fig. 1.

**Distribution. *Northern***: Chiang Mai (Chiang Dao WS, Mae Taeng, Mae Chaem, Doi Suthep–Pui NP), Lampang (Tham Pha Thai NP). ***Western***: Tak (Umphang WS, Thung Yai Naresuan East WS). ***Northeastern***: Kalasin (Phu Sithan WS), Loei (Phu Luang WS), Mukdahan (Phu Sithan WS), Nakhon Ratchasima (Sakaerat, Khao Yai NP), Chaiyaphum (Phu Khiao WS). ***Central***: Phitsanulok (Thung Salaeng Luang NP). ***Eastern***: Chachoengsao (Khao Ang Reu Nai WS), Chanthaburi (Khao Soi Dao WS, Pheao NP), Trat (Ko Kut). ***Southern***: Surat Thani (Tai Rom Yen NP), Phatthalung (Khao Pu–Khao Ya NP), Krabi (Ko Lanta), Krabi (Ko Lanta), Trang (Khao Chong BG), Songkhla (Ton Nga Chang WS), Narathiwat (Hala–Bala WS).

**References.**Jaitrong and Nabhitabhata (2005), Eguchi (2008), Jaitrong and Jeenthong (2014a).

***Pheidole plinii* Forel, 1911**

*Pheidole plinii* Forel, 1911a: 40.

**Distribution. *Western***: Tak (Thung Yai Naresuan East WS). ***Eastern***: Chanthaburi (Pheao NP). ***Southern***: Songkhla (Ton Nga Chang WS, Prince of Songkhla University).

**References.**Watanasit and Noon–anant (2005), Watanasit et al. (2007).

***Pheidole protea* Forel, 1912**

Pheidole javana subsp. proteus Forel, 1912c: 55.

**Distribution. *Northern***: Chiang Mai (Doi Suthep–Pui NP, Omkoi). ***Western***: Tak (Umphang WS, Thung Yai Naresuan East WS), Prachuap Khiri Khan (Pala–U Waterfall). ***Northeastern***: Loei (Phu Luang WS). ***Central***: Samut Prakan (Bang Krachao). ***Eastern***: Chanthaburi (Khao Soi Dao WS), Trat (Ko Kut).

**References.**Jaitrong and Nabhitabhata (2005), Eguchi et al. (2020).

***Pheidole quadricuspis* Emery, 1900**

*Pheidole quadricuspis* Emery, 1900a: 683.

**Distribution. *Western***: Tak (Thung Yai Naresuan East WS). ***Eastern***: Chanthaburi (Khao Ang Reu Nai WS). ***Southern***: Ranong (Khlong Na Kha WS), Surat Thani (Khlong Yan WS), Nakhon Si Thammarat (Khao Nan NP, Khiri Wong), Phatthalung (Khao Pu–Khao Ya NP), Songkhla (Ton Nga Chang WS), Narathiwat (Hala–Bala WS).

**References.**Jaitrong and Nabhitabhata (2005).

***Pheidole rabo* Forel, 1913**

*Pheidole rabo* Forel, 1913: 28.

**Distribution. *Western***: Tak (Thung Yai Naresuan East WS). ***Northeastern***: Mukdahan (Phu Sithan WS), Nakhon Ratchasima (Sakaerat) ***Eastern***: Chachoengsao (Khao Ang Reu Nai WS), Sa Kaeo (Khao Ang Reunai WS), Rayong (Mu Ko Man). ***Southern***: Surat Thani (Khao Sok NP), Nakhon Si Thammarat (Khao Luang NP, Khao Nan NP), Krabi (Ko Lanta), Trang (Khao Chong BG), Songkhla (Ton Nga Chang WS, Khao Kho Hong), Narathiwat (Hala–Bala WS).

**References.**Jaitrong and Nabhitabhata (2005, cited as *Pheidole tsailuni* Wheeler, 1929, junior synonym of *P. rabo*), Eguchi (2008), Jaitrong and Jeenthong (2014a).

***Pheidole rinae* Emery, 1900**

*Pheidole rinae* Emery, 1900a: 687.

**Distribution. *Western***: Tak (Thung Yai Naresuan East WS). ***Southern***: Trang (Khao Chong BG).

**References.**Jaitrong and Nabhitabhata (2005).

***Pheidole rugithorax* Eguchi, 2008**

*Pheidole rugithorax* Eguchi, 2008: 84, fig. 23a–g.

**Distribution. *Northern***: Chiang Mai (Doi Suthep–Pui NP). ***Western***: Kanchanaburi (Khuean Srinagarindra NP) ***Northeastern***: Mukdahan (Phu Sithan WS). ***Eastern***: Chanthaburi (Khao Soi Dao WS).

**References.**Eguchi (2008).

***Pheidole sarawakana* Forel, 1911**

Pheidole sauberi subsp. sarawakana Forel, 1911a: 45.

**Distribution. *Southern***: Narathiwat (Hala–Bala WS).

**References.**Jaitrong and Nabhitabhata (2005).

***Pheidole sauberi* Forel, 1905**

*Pheidole sauberi* Forel, 1905: 18.

**Distribution. *Southern***: Surat Thani (Khlong Yan WS, Khlong Saeng WS), Trang (Khao Chong BG), Narathiwat (Hala–Bala WS).

**References.**Jaitrong and Nabhitabhata (2005).

***Pheidole singaporensis* Özdikmen, 2010**

*Myrmica longipes* Smith, 1857: 233, pl. 11, fig. 68.

**Distribution. *Western***: Tak (Thung Yai Naresuan East WS, Umphang WS), Kanchanaburi (Maeklong Watershed Research Station, Thong Pha Phum NP), Phetchaburi (Kaeng Krachan NP), Prachuap Khiri Khan (Pala–U Waterfall). ***Northeastern***: Nakhon Ratchasima (Khao Yai NP, Sakaerat). ***Central***: Uthai Thani (Huai Kha Khaeng WS). ***Eastern***: Chachoengsao (Khao Ang Reu Nai WS), Chanthaburi (Khao Soi Dao WS, Pheao NP). ***Southern***: Chumphon (Krom Luang Chumphon WS), Ranong (Khlong Na Kha WS), Surat Thani (Tai Rom Yen NP, Khlong Yan WS, Khlong Saeng WS), Nakhon Si Thammarat (Khao Luang NP, Khao Nan NP), Phatthalung (Khao Pu–Khao Ya NP), Trang (Ton Tae Waterfall, Khao Chong BG, Thung Khai BG), Satun (Tarutao NP), Songkhla (Ton Nga Chang WS), Narathiwat (Hala–Bala WS).

**References.**Eguchi (2001), Jaitrong and Nabhitabhata (2005).

***Pheidole smythiesii* Forel, 1902**

Pheidole (Ceratopheidole) smythiesii Forel, 1902b: 165.

**Distribution. *Northern***: Chiang Mai (Khun Chang Khian, Doi Suthep–Pui NP), Nan (Doi Phu Kha NP). ***Western***: Tak (Umphang WS, Thung Yai Naresuan East WS). ***Northeastern***: Loei (Phu Luang WS). ***Central***: Uthai Thani (Huai Kha Khaeng WS).

**References.**Jaitrong and Nabhitabhata (2005), Eguchi (2008), Sakchoowong et al. (2008), Onishi et al. (2016), Eguchi et al. (2020).

***Pheidole spathifera* Forel, 1902**

*Pheidole spathifera* Forel, 1902b: 168.

**Distribution. *Northern***: Chiang Mai (Mae Taeng, Doi Ang Khang). ***Western***: Tak (Thung Yai Naresuan East WS). ***Northeastern***: Nakhon Ratchasima (Sakaerat).

**References.**Jaitrong and Nabhitabhata (2005).

***Pheidole taipoana* Wheeler, 1928**

Pheidole rinae subsp. taipoana Wheeler, 1928b: 12.

**Distribution. *Western***: Kanchanaburi (Thong Pha Phum).

**References.**Torchote et al. (2010).

***Pheidole taivanensis* Forel, 1912**

*Pheidole taivanensis* Forel, 1912d: 59.

**Distribution. *Northern***: Chiang Mai (Doi Chiang Dao).

**References.**Jaitrong and Nabhitabhata (2005).

***Pheidole tandjongensis* Forel, 1913**

*Pheidole tandjongensis* Forel, 1913: 42.

**Distribution. *Northern***: Chiang Mai (Khun Chang Khian). ***Western***: Tak (Thung Yai Naresuan East WS, Umphang WS), Kanchanaburi (Thong Pha Phum NP). ***Northeastern***: Mukdahan (Phu Sithan WS), Nakhon Ratchasima (Khao Yai NP, Sakaerat). ***Central***: Uthai Thani (Ban Rai), Phetchaburi (Kaeng Krachan NP). ***Eastern***: Sa Kaeo (Khao Ang Reu Nai WS, Pang Sida NP), Chon Buri (Si Racha), Chachoengsao (Khao Ang Reu Nai WS), Chanthaburi (Pheao NP, Khao Soi Dao WS), Rayong (Mu Ko Man). ***Southern***: Chumphon (Krom Luang Chumphon WS), Ranong (Khlong Na Kha WS), Surat Thani (Mu Ko Ang ThongNP), Nakhon Si Thammarat (Khao Luang NP), Phatthalung (Khao Pu–Khao Ya NP), Krabi (Ko Lanta), Trang (Ton Tae Waterfall, Khao Chong BG), Satun (Kok Adunk), Narathiwat (Hala–Bala WS).

**References.**Jaitrong and Nabhitabhata (2005), Jaitrong and Jeenthong (2014a).

***Pheidole tjibodana* Forel, 1905**

Pheidole nodgii var. tjibodana Forel, 1905: 16.

**Distribution. *Western***: Tak (Umphang WS, Thung Yai Naresuan East WS). ***Northeastern***: Nakhon Ratchasima (Khao Yai NP, Sakaerat). ***Eastern***: Sa Kaeo (Pang Sida NP), Chachoengsao (Khao Ang Reu Nai WS). ***Southern***: Surat Thani (Khlong Saeng WS), Trang (Khao Chong BG).

**References.**Jaitrong and Nabhitabhata (2005), Eguchi (2008).

***Pheidole tumida* Eguchi, 2008**

*Pheidole tumida* Eguchi, 2008: 97, fig. 27a–g.

**Distribution. *Northern***: Chiang Rai (Doi Tung), Chiang Mai (Omkoi). ***Western***: Tak (Umphang WS, Thung Yai Naresuan East WS), Phetchaburi (Kaeng Krachan NP). ***Northeastern***: Mukdahan (Phu Sithan WS), Nakhon Ratchasima (Khao Yai NP, Sakaerat). ***Central***: Uthai Thani (Huai Kha Khaeng WS). ***Eastern***: Chachoengsao (Khao Ang Reu Nai WS), Chanthaburi (Khao Soi Dao WS, Pheao NP). ***Southern***: Ranong (Khlong Na Kha WS), Surat Thani (Tai Rom Yen NP), Trang (Khao Chong BG), Narathiwat (Hala–Bala WS).

**References.**Eguchi (2008).

**Remarks.** All Thai specimens identified as *Pheidole nodifera* (Smith, 1858) in Jaitrong and Nabhitabhata (2005) should be reidentified as *P. tumida* in the present paper (sensu Eguchi, 2008).

***Pheidole vulgaris* Eguchi, 2006**

*Pheidole vulgaris* Eguchi, 2006: 127, fig. 6A–I.

**Distribution. *Northern***: Chiang Mai (Doi Suthep–Pui NP). ***Western***: Tak (Umphang WS). ***Northeastern***: Nakhon Ratchasima (Khao Yai NP).

**References.**Eguchi (2006, 2008).

***Pheidole yeensis* Forel, 1902**

Pheidole sulcaticeps r. yeensis Forel, 1902b: 179.

**Distribution. *Northern***: Chiang Mai (Omkoi, Chiang Dao WS), Lampang (Tham Pha Thai NP). ***Western***: Tak (Thung Yai Naresuan East WS, Umphang WS). ***Northeastern***: Loei (Phu Luang WS), Kalasin (Phuthan WS), Nakhon Rachasima (Sakaerat). ***Central***: Uthai Thani (Huai Kha Khaeng WS). ***Eastern***: Chachoengsao (Khao Ang Reu Nai WS), Chanthaburi (Khao Soi Dao WS, Pheao NP), Rayong (Khao Ang Reu Nai WS). ***Southern***: Chumphom (Krom Luang Chumphon WS), Surat Thani (Khlong Saeng WS).

**References.**Jaitrong and Nabhitabhata (2005), Eguchi (2008).

***Pheidole zoceana* Santschi, 1925**

Pheidole nodgii var. zoceana Santschi, 1925: 83.

**Distribution. *Northern***: Chiang Mai (Doi Suthep–Pui NP). ***Western***: Prachuap Khiri Khan (Pala–U Waterfall).

**References.**Jaitrong and Nabhitabhata (2005), Eguchi (2008).

***Pristomyrmex bicolor* Emery, 1900**

Pristomyrmex trachylissa var. bicolor Emery, 1900a: 678.

**Distribution. *Southern***: Surat Thani (Ban Na San).

**References.**Laciny et al. (2016).

***Pristomyrmex brevispinosus* Emery, 1887**

*Pristomyrmex brevispinosus* Emery, 1887e: 451.

**Distribution. *Northern***: Chiang Mai (Doi Suthep–Pui NP). ***Western***: Tak (Thung Yai Naresuan East WS, Umphang WS). ***Northeastern***: Nakhon Rachasima (Khao Yai NP). ***Eastern***: Chachoengsao (Khao Ang Reu Nai WS), Chanthaburi (Khao Soi Dao WS). ***Southern***: Trang (Khao Chong BG), Pattani (Nong Chik).

**References.**Bingham (1906), Jaitrong and Nabhitabhata (2005), Zettel (2006).

***Pristomyrmex leleji* Yamane & Dias, 2016**

*Pristomyrmex leleji* Yamane & Dias, 2016: 189, figs 2, 4–9.

**Distribution. *Western***: Tak (Thung Yai Naresuan East WS). ***Northeastern***: Nakhon Rachasima (Sakaerat). ***Central***: Uthai Thani (Ban Rai). ***Eastern***: Chanthaburi (Khao Khitchakut NP*).

**References.**Yamane and Dias (2016).

***Pristomyrmex punctatus* (Smith, 1860)**

*Myrmica punctata* Smith, 1860a: 108.

**Distribution. *Northern***: Chiang Rai (Doi Tung), Chiang Mai (Doi Suthep–Pui NP, Fang, Mae Wang), Nan (Doi Phu Kha NP). ***Western***: Tak (Thung Yai Naresuan East WS, Umphang WS). ***Northeastern***: Loei (Phu Luang WS), Chaiyaphum (Phu Khiao WS), Nakhon Rachasima (Khao Yai NP). ***Eastern***: Chanthaburi (Khao Soi Dao WS). ***Southern***: Trang (Khao Chong BG).

**References.**Jaitrong and Nabhitabhata (2005, cited as *Pristomyrmex pungens* Mayr, 1866, junior synonym of *P. punctatus*), Zettel (2006).

***Pristomyrmex rigidus* Wang, 2003**

*Pristomyrmex rigidus* Wang, 2003: 415, figs 94–97.

**Distribution. *Western***: Tak (Thung Yai Naresuan East WS, Umphang WS), Phetchaburi (Kaeng Krachan NP). ***Eastern***: Chanthaburi (Khao Sabab*). ***Southern***: Nakhon Si Thammarat (Tapi Watershed Research Station, Khao Nan NP), Phuket (Thalang), Krabi (Ko Lanta).

**References.**Wang (2003), Jaitrong and Jeenthong (2014a).

***Pristomyrmex sulcatus* Emery, 1895**

Pristomyrmex brevispinosus subsp. sulcatus Emery, 1895: 464.

**Distribution. *Northern***: Chiang Mai (Doi Suthep–Pui NP, Khao Soi Dao WS, 53 km from Chiang Mai), Nan (Doi Phuka NP). ***Western***: Tak (Umphang WS, Thung Yai Naresuan East WS), Kanchanaburi (Thong Pha Phum NP). ***Northeastern***: Loei (Phu Luang WS), Nakhon Rachasima (Khao Yai NP). ***Central***: Uthai Thani (Ban Rai). ***Eastern***: Chachoengsao (Khao Ang Reu Nai WS), Chanthaburi (Khao Soi Dao WS, Pheao NP, Khao Ang Reu Nai WS, Khlong Thab Mak Waterfall), Trat (Ko Kut).

**References.**Jaitrong and Nabhitabhata (2005), Wang (2003), Sakchoowong et al. (2009).

***Pristomyrmex trachylissus* (Smith, 1858)**

*Myrmica trachylissa* Smith, 1858: 126.

**Distribution. *Southern***: Trang (Khao Chong BG).

**References.**Jaitrong and Nabhitabhata (2005).

***Proatta butteli* Forel, 1912**

*Proatta butteli* Forel, 1912b: 769.

**Distribution. *Western***: Tak (Thung Yai Naresuan East WS, Umphang WS). ***Northeastern***: Chaiyaphum (Phu Khiao WS), Nakhon Rachasima (Sakaerat, Khao Yai NP). ***Central***: Uthai Thani (Huai Kha Khaeng WS), Saraburi (Phu Kae BG), Nakhon Nayok (Khao Cha Ngok, Sarika Waterfall). ***Eastern***: Sa Kaeo (Khao Ang Reu Nai WS), Chachoengsao (Khao Ang Reu Nai WS), Chanthaburi (Khao Soi Dao WS, Pheao NP). ***Southern***: Chumphon (Krom Luang Chumphon WS), Surat Thani (Tai Rom Yen NP), Krabi (Ko Lanta), Trang (Khao Chong BG).

**References.**Jaitrong and Nabhitabhata (2005), Jaitrong (2011), Jaitrong and Jeenthong (2014a).

***Recurvidris browni* Bolton, 1992**

*Recurvidris browni* Bolton, 1992: 43, figs 1, 3.

**Distribution. *Southern***: Nakhon Si Thammarat (Khao Nan NP), Narathiwat (Hala–Bala WS).

**References.**Jaitrong and Wiwatwitaya (2015).

***Recurvidris chanapaithooni* Jaitrong & Wiwatwitaya, 2015**

*Recurvidris chanapaithooni* Jaitrong & Wiwatwitaya, 2015: 106, fig. 2.

**Distribution. *Eastern***: Chon Buri (Khao Kheow Open Zoo), Chachoengsao (Khao Ang Reu Nai WS), Chanthaburi (Khao Soi Dao WS*). ***Southern***: Trang (Khao Chong BG).

**References.**Jaitrong and Wiwatwitaya (2015), Jaitrong et al. (2019b).

***Recurvidris lekakuli* Jaitrong, Tokeeree & Pitaktunsakul, 2019**

*Recurvidris lekakuli* Jaitrong, Tokeeree & Pitaktunsakul, 2019b: 55, figs 1–5.

**Distribution. *Western***: Kanchanaburi (Thong Pha Phum*).

**References.**Jaitrong et al. (2019b).

***Recurvidris recurvispinosa* (Forel, 1890)**

*Trigonogaster recurvispinosus* Forel, 1890b: cix.

**Distribution. *Northern***: Chiang Mai (Khun Chang Khian, Chiang Mai University, Doi Chang Khian). ***Western***: Tak (Umphang WS), Kanchanaburi (Thong Pha Phum NP). ***Northeastern***: Nakhon Rachasima (Sakaerat, Khao Yai NP, Pakchong). ***Eastern***: Chon Buri (Khao Kheow Open Zoo), Chachoengsao (Khao Ang Reu Nai WS), Chanthaburi (Khao Soi Dao WS), Trat (Ko Kut).

**References.**Jaitrong and Nabhitabhata (2005), Jaitrong and Wiwatwitaya (2015), Jaitrong (2011), Onishi et al. (2016), Jaitrong et al. (2019b).

***Rhopalomastix javana* Wheeler, 1929**

Rhopalomastix rothneyi subsp. javana Wheeler, 1929: 96.

**Distribution. *Western***: Kanchanaburi (Sai Yok). ***Central***: Saraburi (Phu Kae BG).

**References.** Wang et al. (2018)

***Rhopalomastix johorensis* Wheeler, 1929**

Rhopalomastix rothneyi subsp. johorensis Wheeler, 1929: 96.

*Rhopalomastix janeti* Donisthorpe, 1936: 55.

**Distribution. *Central***: Ang Thong (Chaiyo), Nakhon Nayok (Banna), Pathumthani (Khlong Luang, Khlong 5 and Khlong 3), Saraburi (Phu Kae BG, Kaeng Koi). ***Eastern***: Chanthaburi (Tha Mai). ***Southern***: Trang (Nayong).

**References.** Wang et al. (2018)

***Solenopsis geminata* (Fabricius, 1804)**

*Atta geminata* Fabricius, 1804: 423.

**Distribution. *Northern***: Chiang Rai (Doi Tung), Chiang Mai (Doi Ang Khang, Doi Luang Chiang Dao, Chiang Dao WS, Mae Taeng, Doi Suthep–Pui NP, Chiang Mai University Campus, Doi Inthanon NP, Mae Chaem, Omkoi, Doi Pha Hom Pok NP), Phayao (Mae Ka), Lamphun (Mae Li Forest Plantation), Lampang (Haui Tak, Tham Pha Thai NP), Phrae (Wang Chin Forest Plantation), Nan (Doi Phu Kha NP, Nakhon Nan Forest Plantation). ***Western***: Tak (Umphang WS, Thung Yai Naresuan East WS, Lansang NP, Taksin Maharat NP), Kanchanaburi (Thong Pha Phum NP, Pha Tad Watershed Management station), Phetchaburi (Kaeng Krachan NP). ***Northeastern***: Kalasin (Phu Sithan WS), Chaiyaphum (Phu Khiao WS), Loei (Phu Luang WS), Nakhon Ratchasima (Sakaerat, Khao Yai NP, Buer Yai), Ubon Ratchathani (Pha Taem NP). ***Central***: Saraburi (Phukae BG), Bangkok (Bang Khen, Chatuchak), Pathum Thani (Khlong Luang), Samut Songkhram (Mueang Samut Songkhram). ***Eastern***: Sa Kaeo (Khao Ang Reu Nai WS), Chachoengsao (Khao Ang Reu Nai WS), Chon Buri (Si Racha, Khao Kheow, Ko Samaesarn), Chanthaburi (Khao Soi Dao WS, Pheao NP, Khao Ang Reu Nai WS), Rayong (Ko Man Nai, Khao Ang Reu Nai WS), Trat (Khlong Yai). ***Southern***: Chumphon (Krom Luang Chumphon NP), Ranong (Suk Samran, Khlong Na Kha WS), Surat Thani (Tai Rom Yen NP, Khlong Yan WS, Mu Ko Ang Thong NP, Khlong Saeng WS), Nakhon Si Thammarat (Khao Nan NP, Khao Luang NP, Krung Ching Waterfall), Krabi (Ko Lanta), Trang (Khao Chong BG, Thung Khai BG, Palian, Ton Tae Waterfall), Phatthalung (Khao Pu–Khao Ya NP, Khao Bantad WS), Satun (Tarutao NP), Songkhla (Khao Kho Hong, Khao Nam Khang NP), Pattani (Namtok Sai Khao NP), Narathiwat (Hala–Bala WS).

**References.**Jaitrong and Nabhitabhata (2005), Jaitrong (2011), Jaitrong and Jeenthong (2014a), Suwannaphak et al. (2016).

***Strumigenys adiastola* Bolton, 2000**

*Strumigenys adiastola* Bolton, 2000: 836.

**Distribution. *Northern***: Chiang Mai (Pa Miang Village). ***Western***: Tak (Thung Yai Naresuan East WS), Kanchanaburi (Erawan Waterfall*), Phetchaburi (Kaeng Krachan NP). ***Northeastern***: Nakhon Ratchasima (Khao Yai NP). ***Eastern***: Chanthaburi (Khao Khitchakut NP). ***Southern***: Nakhon Si Thammarat (Khao Nan NP).

**References.**Bolton (2000), Onishi et al. (2016).

***Strumigenys amnesia* Bolton, 2000**

*Strumigenys amnesia* Bolton, 2000: 838.

**Distribution. *Southern***: Trang (Khao Chong BG).

**References.**Bolton (2000).

***Strumigenys arges* (Bolton, 2000)**

*Pyramica arges* Bolton, 2000: 462.

**Distribution. *Northern***: Chiang Mai (Doi Suthep–Pui NP*).

**References.** Bolton (2000)

***Strumigenys atropos* (Bolton, 2000)**

*Pyramica atropos* Bolton, 2000: 457.

**Distribution. *Western***: Phetchaburi (Kaeng Krachan NP*).

**References.** Bolton (2000)

***Strumigenys benulia* Bolton, 2000**

*Strumigenys benulia* Bolton, 2000: 754

**Distribution. *Western***: Phetchaburi (Kaeng Krachan NP*).

**References.**Bolton (2000).

***Strumigenys brontes* (Bolton, 2000)**

*Pyramica brontes* Bolton, 2000: 463, figs 268, 294.

**Distribution. *Western***: Phetchaburi (Kaeng Krachan NP*). ***Eastern***: Chanthaburi (Khao Khitchakut NP).

**References.**Bolton (2000).

***Strumigenys caniophanes* Bolton, 2000**

*Strumigenys caniophanes* Bolton, 2000: 755, figs 423, 490.

**Distribution. *Northern***: Chiang Mai (Doi Suthep–Pui NP*).

**References.**Bolton (2000).

***Strumigenys confusatrix* Bolton, 2000**

*Strumigenys confusatrix* Bolton, 2000: 789, figs 433, 499.

**Distribution. *Southern***: Phatthalung (Phatthalung Wildlife Breeding Center).

**References.**Bolton (2000).

***Strumigenys dipsas* Bolton, 2000**

*Strumigenys dipsas* Bolton, 2000: 757.

**Distribution. *Northern***: Chiang Mai (Doi Inthanon NP*).

**References.**Bolton (2000).

***Strumigenys dohertyi* Emery, 1897**

*Strumigenys dohertyi* Emery, 1897: 576.

**Distribution. *Northern***: Chiang Mai (Doi Suthep–Pui NP). ***Western***: Tak (Umphang WS, Thung Yai WS). ***Western***: Phetchaburi (Kaeng Krachan NP). ***Northeastern***: Nakhon Ratchasima (Khao Yai NP). ***Southern***: Surat Thani (Ban Nasan), Nakhon Si Thammarat (Khao Nan NP).

**References.** Bolton (2000)

***Strumigenys doriae* Emery, 1887**

*Strumigenys doriae* Emery, 1887f: 469, pl. 2, fig. 22.

**Distribution. *Northern***: Chiang Mai (unknown locality). ***Western***: Tak (Umphang WS, Thung Yai Naresuan East WS).

**References.**Bolton (2000).

***Strumigenys elegantula* (Terayama & Kubota, 1989)**

*Smisthistruma elegantula* Terayama & Kubota, 1989: 788, figs 23–27.

**Distribution. *Northern***: Chiang Mai (Doi Inthanon NP). ***Northeastern***: Mukdahan (Phu Sithan WS).

**References.** Bolton (2000)

***Strumigenys exilirhina* Bolton, 2000**

*Strumigenys exilirhina* Bolton, 2000: 881.

**Distribution. *Northern***: Chiang Mai (Doi Suthep–Pui NP). ***Western***: Phetchaburi (Kaeng Krachan NP). ***Northeastern***: Chaiyaphum (Thung Ka Mang [Phu Khiao WS]), Nakhon Ratchasima (Khao Yai NP). ***Eastern***: Chanthaburi (Khao Sabab).

**References.**Bolton (2000).

***Strumigenys feae* Emery, 1895**

*Strumigenys feae* Emery, 1895: 473.

**Distribution. *Northern***: Mae Hong Son (Sop Pong). ***Western***: Phetchaburi (Kaeng Krachan NP). ***Eastern***: Chanthaburi (Khao Chamao NP).

**References.**Bolton (2000).

***Strumigenys gnathosphax* Bolton, 2000**

*Strumigenys gnathosphax* Bolton, 2000: 911, fig. 481.

**Distribution. *Northern***: Chiang Mai (Doi Suthep–Pui NP). ***Northeastern***: Nakhon Ratchasima (Khao Yai NP). ***Southern***: Nakhon Si Thammarat (Khao Luang NP), Satun (Thale Ban NP).

**References.**Bolton (2000).

***Strumigenys kichijo* (Terayama, Lin et Wu, 1996)**

*Smithistruma kichijo* Terayama, Lin & Wu, 1996: 335, figs 23–25, 28, 29.

**Distribution. *Northern***: Chiang Mai (Doi Inthanon NP), Mae Hong Son (Pai). ***Northeastern***: Nakhon Ratchasima (Khao Yai NP).

**References.** Bolton (2000)

***Strumigenys kraepelini* Forel, 1905**

*Strumigenys kraepelini* Forel, 1905: 8.

**Distribution. *Western***: Tak (Umphang WS, Thung Yai Naresuan East WS), Prachuap Khiri Khan (Pala–U Waterfall). ***Southern***: Surat Thani (Khlong Saeng WS), Krabi (Ko Lanta), Nakhon Si Thammarat (Khao Nan NP, Khao Luang NP), Trang (Khao Chong BG, Ton Tae Waterfall), Satun (Thale Ban NP), Narathiwat (Hala–Bala WS).

**References.**Bolton (2000).

***Strumigenys mitis* (Brown, 2000)**

*Pyramica mitis* Brown, in Bolton, 2000: 442, figs 267, 290.

**Distribution. *Northern***: Chiang Mai (Web Pang An, Doi Inthanon NP, Doi Saket). ***Eastern***: Chanthaburi (Khao Khitchakut NP). ***Southern***: Nakhon Si Thammarat (Nam Tok Prom Lok, Khao Nan NP).

**References.**Bolton (2000).

***Strumigenys nanzanensis* Lin & Wu, 1996**

*Strumigenys nanzanensis* Lin & Wu, 1996: 148, figs 13, 30–34.

**Distribution. *Western***: Phetchaburi (Kaeng Krachan NP). ***Northeastern***: Nakhon Ratchasima (Khao Yai NP). ***Southern***: Phang–nga (Khao Lak), Trang (Khao Chong BG), Satun (Thale Ban NP).

**References.**Bolton (2000).

***Strumigenys nepalensis* De Andrade, 1994**

*Strumigenys nepalensis* De Andrade, in Baroni Urbani & De Andrade, 1994: 57, figs 33, 34.

**Distribution. *Northern***: Chiang Mai (Chiang Dao WS, Doi Suthep–Pui NP, Doi Inthanon NP), Mae Hong Son (Tham Lot). ***Western***: Kanchanaburi (Erawan NP), Phetchaburi (Kaeng Krachan NP). ***Eastern***: Chanthaburi (Khao Chamao NP). ***Southern***: Chumphon (Krom Luang Chumphon WS).

**References.** Bolton (2000)

***Strumigenys nothomopyx* Bolton, 2000**

*Strumigenys nothomopyx* Bolton, 2000: 763.

**Distribution. *Northern***: Chiang Mai (Doi Suthep–Pui NP [Monthathan Waterfall*]).

**References.**Bolton (2000).

***Strumigenys nytaxis* Bolton, 2000**

*Strumigenys nytaxis* Bolton, 2000: 797.

**Distribution. *Western***: Phetchaburi (Kaeng Krachan NP*). ***Eastern***: Chanthaburi (Khao Sabab [Pheao NP]).

**References.**Bolton (2000).

***Strumigenys paraposta* Bolton, 2000**

*Strumigenys paraposta* Bolton, 2000: 763

**Distribution. *Northern***: Chiang Mai (Doi Inthanon NP*).

**References.**Bolton (2000).

***Strumigenys rotogenys* Bolton, 2000**

*Strumigenys rotogenys* Bolton, 2000: 769, figs 426, 491.

**Distribution. *Southern***: Nakhon Si Thammarat (Khao Nan NP), Phang–nga (Ton Chang Fa Waterfall).

**References.**Bolton (2000).

***Strumigenys sauteri* (Forel, 1912)**

*Pentastruma sauteri* Forel, 1912d: 51.

**Distribution. *Northeastern***: Nakhon Ratchasima (Khao Yai NP).

**References.** Bolton (2000)

***Strumigenys scolopax* (Bolton, 2000)**

*Pyramica scolopax* Bolton, 2000: 439.

**Distribution. *Southern***: Phang–nga (Si Phang–nga NP*).

**References.** Bolton (2000)

***Strumigenys signeae* Forel, 1905**

*Strumigenys signeae* Forel, 1905: 10.

**Distribution. *Southern***: Phang–nga (Ton Chang Fa Waterfall).

**References.**Bolton (2000).

***Strumigenys strygax* Bolton, 2000**

*Strumigenys strygax* Bolton, 2000: 853.

**Distribution. *Northern***: Chiang Mai (Chiang Dao WS, Doi Inthanon NP, Khun Chang Khian*). ***Western***: Phetchaburi (Kaeng Krachan NP). ***Northeastern***: Nakhon Ratchasima (Khao Yai NP).

**References.**Bolton (2000).

***Strumigenys sublaminata* Brown, 1959**

*Strumigenys sublaminata* Brown, 1959: 84.

**Distribution. *Southern***: Nakhon Si Thammarat (Prom Lok Waterfall), Phang–nga (Ton Chang Fa Waterfall), Satun (Thale Ban NP).

**References.**Bolton (2000).

***Strumigenys sydorata* Bolton, 2000**

*Strumigenys sydorata* Bolton, 2000: 876

**Distribution. *Northern***: Chiang Mai (Doi Suthep–Pui NP). ***Western***: Tak (Umphang WS). ***Western***: Phetchaburi (Kaeng Krachan NP). ***Eastern***: Chanthaburi (Khao Soi Dao WS, Pheao NP). ***Southern***: Nakhon Si Thammarat (Khao Nan NP, Khao Luang NP).

**References.**Bolton (2000).

***Strumigenys taphra* Bolton, 2000**

*Pyramica taphra* Bolton, 2000: 448. Type locality: THAILAND.

**Distribution. *Northern***: Chiang Mai (Doi Inthanon NP).

**References.**Bolton (2000).

**Remarks.** Unknown type locality.

***Strumigenys tritomea* Bolton, 2000**

*Strumigenys tritomea* Bolton, 2000: 770

**Distribution. *Northern***: Chiang Mai (Web Pang An*).

**References.**Bolton (2000).

***Syllophopsis australica* (Forel, 1907)**

Monomorium subcoecum subsp. australicum Forel, 1907: 20.

**Distribution. *Western***: Tak (Thung Yai Naresuan East WS). ***Northeastern***: Nakhon Ratchasima (Khao Yai NP). ***Eastern***: Chachoengsao (Khao Ang Reu Nai WS).

**References.**Jaitrong and Nabhitabhata (2005, cited as *Monomorium talpa* Emery, 1911, junior synonym of *S. australica*), Jaitrong and Jeenthong (2014, cited as *Monomorium talpa* Emery, 1911).

***Syllophopsis sechellensis* (Emery, 1894)**

Monomorium fossulatum subsp. sechellense Emery, 1894: 69.

**Distribution. *Western***: Tak (Thung Yai Naresuan East WS, Umphang WS). ***Northeastern***: Nakhon Ratchasima (Khao Yai NP).

**References.**Jaitrong and Nabhitabhata (2005, cited as *Monomorium sechellense* Emery, 1894).

***Tetheamyrma subspongia* Bolton, 1991**

*Tetheamyrma subspongia* Bolton, 1991: 10, figs 16, 17.

**Distribution. *Southern***: Nakhon Si Thammarat (Khao Nan NP).

**References.**Jaitrong (2011).

***Tetramorium adelphon* Bolton, 1979**

*Tetramorium adelphon* Bolton, 1979: 177.

**Distribution.** Northern: Chiang Mai (Nong Hoi).

**References.**Bolton (1979).

***Tetramorium aptum* Bolton, 1977**

*Tetramorium aptum* Bolton, 1977: 115

**Distribution. *Northern***: Chiang Mai (Nong Hoi*).

**References.**Bolton (1977, 1995).

***Tetramorium bicarinatum* (Nylander, 1846)**

*Myrmica bicarinata* Nylander, 1846: 1061.

**Distribution. *Northern***: Chiang Mai (Doi Ang Khang), Phrae (Wang Ching). ***Central***: Bangkok (Kasetsart University).

**References.**Jaitrong and Nabhitabhata (2005).

***Tetramorium ciliatum* Bolton, 1977**

*Tetramorium ciliatum* Bolton, 1977: 121, fig. 49.

**Distribution. *Northern***: Chiang Mai (Nong Hoi*).

**References.**Bolton (1977, 1995), Jaitrong and Nabhitabhata (2005).

***Tetramorium cuneinode* Bolton, 1977**

*Tetramorium cuneinode* Bolton, 1977: 126, fig. 56.

**Distribution. *Northern***: Chiang Mai (Nong Hoi*).

**References.**Bolton (1977, 1995).

***Tetramorium eleates* Forel, 1913**

Tetramorium (Xiphomyrmex) tortuosum var. eleates Forel, 1913: 82.

**Distribution. *Northern***: Chiang Mai (Chiang Dao WS). ***Western***: Kanchanaburi (Thong Pha Phum NP). ***Northeastern***: Nakhon Ratchasima (Khao Yai NP, Sakaerat). ***Southern***: Trang (Khao Chong BG), Songkhla (Khao Nam Khang).

**References.**Jaitrong and Nabhitabhata (2005), Sakchoowong et al. (2009).

***Tetramorium flavipes* Emery, 1893**

Tetramorium (Xiphomyrmex) flavipes Emery, 1893c: 247.

**Distribution. *Northern***: Lampang (Mae Had), Chiang Mai (Nong Hoi*). ***Western***: Tak (Thung Yai Naresuan East WS). ***Northeastern***: Nakhon Ratchasima (Sakaerat). ***Eastern***: Chachoengsao (Khao Ang Reu Nai WS), Chanthaburi (Khao Soi Dao WS).

**References.**Emery (1893c), Jaitrong and Nabhitabhata (2005).

***Tetramorium hasinae* Yamane & Jaitrong, 2011**

*Tetramorium hasinae* Yamane & Jaitrong, 2011: 65, fig. 3A–F.

**Distribution. *Southern***: Nakhon Si Thammarat (Khao Nan NP*).

**References.**Yamane and Jaitrong (2011).

***Tetramorium indosinense* Wheeler, 1927**

*Tetramorium indosinense* Wheeler, 1927a: 97, fig. 6.

**Distribution. *Western***: Tak (Thung Yai Naresuan East WS).

**References.**Antweb (2019).

***Tetramorium insolens* (Smith, 1861)**

*Myrmica insolens* Smith, 1861: 47.

**Distribution. *Northeastern***: Nakhon Ratchasima (Khao Yai NP). ***Eastern***: Chanthaburi (Khao Soi Dao WS).

**References.**Jaitrong and Nabhitabhata (2005).

***Tetramorium kheperra* (Bolton, 1976)**

*Triglyphothrix kheperra* Bolton, 1976: 349, fig. 71.

**Distribution. *Northern***: Chiang Mai (Khun Chang Khian, Doi Suthep–Pui NP, Mae Taeng). ***Western***: Tak (Thung Yai Naresuan East WS, Umphang WS), Kanchanaburi (Khuean Srinagarindra NP, Pha Tad Watershed). ***Northeastern***: Chaiyaphum (Phu Khiao WS), Nakhon Ratchasima (Sakaerat, Khao Yai NP). ***Central***: Pathum Thani (Khlong). ***Eastern***: Chachoengsao (Khao Ang Reu Nai WS), Chanthaburi (Khao Soi Dao WS, Pheao NP, Khao Ang Reu Nai WS). ***Southern***: Trang (Khao Chong BG), Songkhla (Nathawee, Khao Nam Khang), Pattani (Namtok Sai Khao NP), Narathiwat (Hala–Bala WS).

**References.**Jaitrong and Nabhitabhata (2005), Yamane and Jaitrong (2011), Suwannaphak et al. (2016), Onishi et al. (2016).

***Tetramorium lanuginosum* Mayr, 1870**

*Tetramorium lanuginosum* Mayr, 1870: 976.

**Distribution. *Northern***: Chiang Mai (Khun Chang Khian) ***Western***: Tak (Thung Yai Naresuan East WS). ***Northeastern***: Nakhon Ratchasima (Khao Yai NP). ***Central***: Bangkok (Kasetsart University), Samut Prakan (Bang Krachao). ***Eastern***: Chanthaburi (Panatnicom). ***Southern***: Narathiwat (Toh Daeng).

**References.**Jaitrong and Nabhitabhata (2005), Wetterer (2010c), Onishi et al. (2016).

***Tetramorium nacta* (Bolton, 1976)**

*Triglyphothrix nacta* Bolton, 1976: 353. Type locality: THAILAND.

**Distribution. *Northern***: Chiang Mai (Nong Hoi*).

**References.**Bolton (1976, 1995).

***Tetramorium nipponense* Wheeler, 1928**

Tetramorium guineense subsp. nipponense Wheeler, 1928c: 115.

**Distribution. *Northern***: Chiang Mai (Mae Taeng). ***Western***: Tak (Thung Yai Naresuan East WS). ***Northeastern***: Nakhon Ratchasima (Sakaerat, Khao Yai NP).

**References.**Jaitrong and Nabhitabhata (2005).

***Tetramorium obtusidens* Viehmeyer, 1916**

*Tetramorium obtusidens* Viehmeyer, 1916: 138, fig. 6.

**Distribution. *Northeastern***: Chaiyaphum (Pha Hin Ngam NP).

**References.**Antweb (2019).

***Tetramorium pacificum* Mayr, 1870**

*Tetramorium pacificum* Mayr, 1870: 976.

**Distribution. *Western***: Tak (Umphang WS, Thung Yai Naresuan East WS). ***Southern***: Surat Thani (Khao Sok NP), Krabi (Ko Lanta).

**References.**Schlick–Steiner et al. (2006), Jaitrong and Jeenthong (2014a).

***Tetramorium palaense* Bolton, 1979**

*Tetramorium palaense* Bolton, 1979: 175, fig. 56

**Distribution. *Northern***: Chiang Mai (Mae Taeng, Chiang Dao WS). ***Western***: Tak (Thung Yai Naresuan East WS). ***Northeastern***: Chaiyaphum (Phu Khiao WS), Nakhon Ratchasima (Khao Yai NP).

**References.**Bolton (1979).

***Tetramorium parvispinum* (Emery, 1893)**

*Triglyphothrix parvispina* Emery, 1893b: 214.

**Distribution. *Northern***: Chiang Mai (Chiang Dao WS).

**References.**Jaitrong and Nabhitabhata (2005).

***Tetramorium parvum* Bolton, 1977**

*Tetramorium parvum* Bolton, 1977: 117.

**Distribution. *Western***: Tak (Thung Yai Naresuan East WS). ***Northeastern***: Nakhon Ratchasima (Khao Yai NP).

**References.**Jaitrong and Nabhitabhata (2005).

***Tetramorium polymorphum* Yamane & Jaitrong, 2011**

*Tetramorium polymorphum* Yamane & Jaitrong, 2011: 68, figs 2D–F, 4A–F.

**Distribution. *Northern***: Chiang Mai (Omkoi). ***Western***: Tak (Umphang WS). ***Northeastern***: Nakhon Ratchasima (Sakaerat) ***Eastern***: Chachoengsao (Khao Ang Reu Nai WS).

**References.**Yamane and Jaitrong (2011).

***Tetramorium securis* Roncin, 2002**

*Tetramorium securis* Roncin, 2002: 283, figs 1, 3, 5.

**Distribution. *Northeastern***: Chaiyaphum (Phu Khiao WS).

**References.**Antweb (2019).

***Tetramorium seneb* Bolton, 1977**

*Tetramorium seneb* Bolton, 1977: 128.

**Distribution. *Northern***: Chiang Mai (Doi Suthep–Pui NP)

**References.**Roncin (2002).

***Tetramorium simillimum* (Smith, 1851)**

*Myrmica simillima* Smith, 1851: 118.

**Distribution. *Northern***: Lampang (Tham Pha Thai NP). ***Northeastern***: Nakhon Ratchasima (Sakaerat, Khao Yai NP). ***Central***: Bangkok (Kasetsart University). ***Southern***: Narathiwat (Toh Daeng).

**References.**Jaitrong and Nabhitabhata (2005).

***Tetramorium smithi* Mayr, 1879**

*Tetramorium smithi* Mayr, 1879: 673.

**Distribution. *Northern***: Chiang Mai (Mae Taeng, Chiang Dao WS). ***Western***: Tak (Umphang WS, Thung Yai Naresuan East WS). ***Northeastern***: Nakhon Ratchasima (Sakaerat, Khao Yai NP). ***Central***: Bangkok (Chatuchak), Pathum Thani (Khlong Luang), Samut Prakan (Bang Krachao). ***Southern***: Narathiwat (Hala–Bala WS).

**References.**Bolton (1977), Jaitrong and Nabhitabhata (2005), Suwannaphak et al. (2016).

***Tetramorium walshi* (Forel, 1890)**

*Triglyphothrix walshi* Forel, 1890a: cvii.

**Distribution. *Northern***: Chiang Mai (Ob Luang NP), Phrae (Wang Chin). ***Northeastern***: Chaiyaphum (Phu Khiao WS), Nakhon Ratchasima (Sakaerat, Khao Yai NP).

**References.**Jaitrong and Nabhitabhata (2005).

***Tetramorium wroughtonii* (Forel, 1902)**

*Rhoptromyrmex wroughtonii* Forel, 1902b: 231.

**Distribution. *Northern***: Chiang Mai (Omkoi). ***Western***: Tak (Umphang WS). ***Northeastern***: Nakhon Ratchasima (Sakaerat, Khao Yai NP). ***Central***: Uthai Thani (Huai Kha Khaeng WS). ***Eastern***: Chachoengsao (Khao Ang Reu Nai WS), Chanthaburi (Khao Soi Dao WS).

**References.**Jaitrong and Nabhitabhata (2005, cited as *Rhoptromyrmex wroughtonii* Forel, 1902b), Jaitrong (2011, cited as *Rhoptromyrmex wroughtonii* Forel, 1902b).

***Trichomyrmex destructor* (Jerdon, 1851)**

*Atta destructor* Jerdon, 1851: 105.

**Distribution. *Northern***: Chiang Rai (Doi Tung), Chiang Mai (Khun Chang Khian, Doi Ang Khang, Doi Luang Chiang Dao, Chiang Dao WS, Mae Taeng, Doi Suthep–Pui NP, Chiang Mai University Campus, Doi Inthanon NP, Mae Chaem), Phayao (Mae Ka), Lamphun (Mae Li Forest Plantation), Lampang (Haui Tak, Tham Pha Thai NP), Phrae (Wang Chin Forest Plantation), Nan (Doi Phu Kha NP, Nakhon Nan Forest Plantation). ***Western***: Tak (Umphang WS, Thung Yai Naresuan East WS, Lansang NP, Taksin Maharat NP), Kanchanaburi (Thong Pha Phum NP, Pha Tad Watershed Management station), Phetchaburi (Kaeng Krachan NP). ***Northeastern***: Kalasin (Phu Sithan WS), Chaiyaphum (Phu Khiao WS), Loei (Phu Luang WS), Nakhon Ratchasima (Sakaerat, Khao Yai NP, Buer Yai), Ubon Ratchathani (Pha Taem NP). ***Central***: Saraburi (Phukae BG), Bangkok (Bang Khen, Chatuchak), Pathum Thani (Kholng Luang). ***Eastern***: Sa Kaeo (Khao Ang Reu Nai WS), Chachoengsao (Khao Ang Reu Nai WS), Chon Buri (Si Racha, Khao Kheow, Ko Samaesarn), Chanthaburi (Khao Soi Dao WS, Pheao NP, Khao Ang Reu Nai WS), Rayong (Ko Man Nai, Khao Ang Reu Nai WS). ***Southern***: Chumphon (Krom Luang Chumphon NP), Ranong (Suk Samran, Khlong Na Kha WS), Surat Thani (Khlong Yan WS, Mu Ko Ang Thong NP), Nakhon Si Thammarat (Khao Nan NP, Khao Luang NP, Krung Ching Waterfall), Krabi (Ko Lanta), Trang (Khao Chong BG, Thung Khai BG, Palian), Phatthalung (Khao Pu–Khao Ya NP, Khao Bantad WS), Satun (Tarutao NP), Songkhla (Khao Kho Hong, Khao Nam Khang NP), Pattani (Yaring), Narathiwat (Hala–Bala WS).

**References.**Bolton (1987), Jaitrong and Nabhitabhata (2005, cited as *Monomorium destructor* (Jerdon, 1851)), Sakchoowong et al. (2009, cited as *Monomorium destructor* (Jerdon, 1851)), Suwannaphak et al. (2016), Onishi et al. (2016).

***Vollenhovia emeryi* Wheeler, 1906**

*Vollenhovia emeryi* Wheeler, 1906: 312, pl. 41, figs 10, 11.

**Distribution.** Thailand (unknown province).

**References.**Brown (1988), Wetterer et al. (2015).

***Vollenhovia fridae* Forel, 1913**

Figs 78, 79

*Vollenhovia fridae* Forel, 1913: 65.

**Distribution. *Western***: Tak (Thung Yai Naresuan East WS, Umphang WS), Kanchanaburi (Thong Pha Phum NP). ***Northeastern***: Loei (Phu Luang WS). ***Southern***: Ranong (Suk Samran), Nakhon Si Thammarat (Tapi Watershed Research Station, Khao Luang NP, Khao Nan NP), Phuket (Thalang), Trang (Khao Chong BG, Ton Tae Waterfall), Phatthalung (Khao Pu–Khao Ya NP), Narathiwat (Hala–Bala WS).

**Remarks.** New record.

**Material examined.** W Thailand, Tak Prov, Umphang Dist, Umphang WS, Kui Lur Tor Station, 25.II.2016, W. Jaitrong leg., TH16–WJT–0104 (THNHM); W Thailand, Tak Prov, Umphang Dist, Umphang WS, Pha Luard Station, 28.I.2015, W. Jaitrong leg., TH15–WJT–149 (THNHM); W Thailand, Tak Prov, Umphang Dist, Thung Yai Naresuan East WS, Uthaki Station, 22.IX.2016, W. Jaitrong leg., TH16–WJT–1040 (THNHM); W Thailand, Tak Prov, Umphang Dist, Thung Yai Naresuan East WS, Thi So Mae Station, 23.II.2016, W. Jaitrong leg., TH16–WJT–0150 (THNHM); W Thailand, Tak Prov, Umphang Dist, Thung Yai Naresuan East WS, Song Pae Station, 23.II.2016, W. Jaitrong leg., TH16–WJT–00049 (THNHM); W Thailand, Kanchanaburi Prov, Thong Pha Phum NP, Hill Evergreen Forest, 20.XII.2003, W. Jaitrong leg., (THNHM); NE Thailand, Loei Prov, Phu Luang WS, 15.V.2007, S. Hasin leg., SH07–TH95 (THNHM); S Thailand, Ranong Prov, Suk Samran Dist, 11.VIII.2009, W. Jaitrong leg. (THNHM); S Thailand, Nakhon Si Thammarat Prov, Noppitam Dist, Khao Luang NP, Krung Ching Waterfall, 20.V.2003, W. Jaitrong leg. (THNHM); S Thailand, Nakhon Si Thammarat Prov, Khao Nan NP, The Wey to Sen Yen, 200–500m, 16.IV.2007, W. Jaitrong leg., WJT07–TH316 (THNHM); S Thailand, Trang Prov, Nayong Dist, Khao Chong BG, 10.III.2007, W. Jaitrong leg., WJT07–TH124 (THNHM); S Thailand, Trang Prov, Palian Dist, Ton Tae Waterfall, 28.III.2005, W. Jaitrong leg., WJT05–S167 (THNHM); S Thailand, Phatthalung Prov, Sribanpot Dist, Khao Pu–Khao Ya NP, 28.IX.2007, W. Jaitrong leg., WJT07–TH2037 (THNHM); S Thailand, Narathiwat Prov, Hala–Bala WS, 7.XI.2002, W. Jaitrong leg., WJT02–TH318 (THNHM).

***Vollenhovia rufiventris* Forel, 1901**

*Vollenhovia rufiventris* Forel, 1901c: 374.

**Distribution. *Southern***: Songkhla (Ton Nga Chang WS, Klong U–Tapao Basin).

**References.**Watanasit et al. (2007).

#### Subfamily Ponerinae [19 genera, 56 species]

***Anochetus graeffei* Mayr, 1870**

*Anochetus graeffei* Mayr, 1870: 961.

**Distribution. *Northern***: Chiang Mai (Doi Ang Khang, Chiang Dao WS, Ob Luang NP, Doi Luang NP, Doi Suthep–Pui NP, Doi Inthanon NP, Mae Taeng, Mae Chaem), Lampang (Huai Tak, Tham Pha Thai NP), Phrae (Wang Chin, Rong Kwang), Nan (Nakhon Nan Forest Plantation). ***Western***: Tak (Umphang WS, Thung Yai Naresuan East WS, Lan Sang NP), Kanchanaburi (Thong Pha Phum NP, Huai Kayeang), Phetchaburi (Kaeng Krachan NP). ***Northeastern***: Kalasin (Phu Sithan WS), Chaiyaphum (Phu Khiao WS, Pa Hin Ngam NP), Loei (Phu Luang WS), Nakhon Ratchasima (Sakaerat, Khao Yai NP), Ubon Ratchathani (Pha Taem NP). ***Central***: Uthai Thani (Huai Kha kang WS), Saraburi (Phu Kae BG), Bangkok (Chatuchak, Bang Khen), Pathum Thani (Khlong Luang), Samut Prakan (Bang Krachao), Samut Songkhram (Mueang Samut Songkhram). ***Eastern***: Chachoengsao (Khao Ang Reu Nai WS), Chon Buri (Khao Kheow Zoo, Si Racha, Ko Samaesarn), Chanthaburi (Khao Soi Dao WS, Pheao NP), Trat (Ko Chang). ***Southern***: Chumphon (Krom Luang Chumphon WS), Ranong (Khlong Na Kha WS, Khlong Yan WS), Nakhon Si Thammarat (Khao Luang NP, Khao Nan NP), Krabi (Ko Lanta), Trang (Palian, Khao Chong BG, Thung Khai BG), Phatthalung (Khao Pu–Khao Ya NP, Tuag Khao Bantad WS), Songkhla (Ton Nga Chang WS, Khao Nam Khang NP, Khao Kho Hong), Narathiwat (Hala–Bala WS).

**References.**Jaitrong and Nabhitabhata (2005), Sakchoowong et al. (2008), Sakchoowong et al. (2009), Jaitrong and Jeenthong (2014a), Suwannaphak et al. (2016).

***Anochetus modicus* Brown, 1978**

*Anochetus modicus* Brown, 1978: 582.

**Distribution. *Western***: Kanchanaburi (Thong Pha Phum NP). ***Eastern***: Chachoengsao (Khao Ang Reu Nai WS), Chanthaburi (Pong Nam Ron).

**References.**Jaitrong and Nabhitabhata (2005), Sakchoowong et al. (2009).

***Anochetus myops* Emery, 1893**

*Anochetus myops* Emery, 1893b: 201, pl. 8, figs 11, 12.

**Distribution. *Northern***: Chiang Mai (Pa Miang Village, Doi Suthep–Pui NP). ***Central***: Uthai Thani (Huai Kha Khaeng WS). ***Eastern***: Chachoengsao (Khao Ang Reu Nai WS). ***Southern***: Surat Thani (Khlong Yan WS), Songkhla (Ton Nga Chang WS).

**References.**Jaitrong and Nabhitabhata (2005), Onishi et al. (2016).

***Anochetus princeps* Emery, 1884**

*Anochetus princeps* Emery, 1884: 379.

**Distribution. *Western***: Tak (Thung Yai Naresuan East WS), Phetchaburi (Kaeng Krachan NP). Northeastern: Loei (Phu Luang WS), Nakhon Ratchasima (Khao Yai NP). ***Central***: Uthai Thani (Ban Rai). ***Eastern***: Sa Kaeo (Khao Ang Reu Nai WS), Chachoengsao (Khao Ang Reu Nai WS), Trat (Ko Kut).

**References.**Jaitrong and Nabhitabhata (2005).

***Anochetus rugosus* (Smith, 1857)**

*Odontomachus rugosus* Smith, 1857: 65.

**Distribution. *Eastern***: Chachoengsao (Khao Ang Reu Nai WS).

**References.**Jaitrong and Nabhitabhata (2005).

***Brachyponera chinensis* (Emery, 1895) species complex**

Ponera nigrita subsp. chinensis Emery, 1895: 460.

**Distribution. *Northern***: Chiang Mai (Pa Miang Village, Doi Luang NP, Omkoi). ***Western***: Tak (Thung Yai Naresuan East WS, Umphang WS), Kanchanaburi (Thong Pha Phum). ***Northeastern***: Chaiyaphum (Phu Khiao WS), Nakhon Ratchasima (Khao Yai NP, Sakaerat). ***Central***: Uthai Thani (Huai Kha Khaeng WS). ***Eastern***: Chachoengsao (Khao Ang Reu Nai WS), Chanthaburi (Khao Soi Dao WS). ***Southern***: Ranong (Khlong Na Kha WS), Surat Thani (Khlong Yan WS), Nakhon Si Thammarat (Khao Luang NP, Khao Nan NP), Trang (Palian, Khao Chong BG, Thung Khai BG), Phatthalung (Khao Pu–Khao Ya NP, Tuag Khao Bantad WS), Narathiwat (Hala–Bala WS).

**References.**Jaitrong and Nabhitabhata (2005, cited as *Pachycondyla chinensis* (Emery, 1895)), Sakchoowong et al. (2008, cited as *Pachycondyla chinensis* (Emery, 1895)), Onishi et al. (2016).

***Brachyponera luteipes* (Mayr, 1862)**

*Ponera luteipes* Mayr, 1862: 722.

**Distribution. *Northern***: Chiang Mai (Khun Chang Khian, Pa Miang Village, Doi Ang Khang, Doi Luang Chiang Dao, Chiang Dao WS, Mae Taeng, Doi Suthep–Pui NP, Chiang Mai University Campus, Doi Inthanon NP, Mae Chaem), Phayao (Mae Ka), Lamphun (Mae Li Forest Plantation), Lampang (Haui Tak, Tham Pha Thai NP), Phrae (Wang Chin Forest Plantation), Nan (Doi Phu Kha NP, Nakhon Nan Forest Plantation). ***Western***: Tak (Umphang WS, Thung Yai Naresuan East WS, Lansang NP, Taksin Maharat NP), Kanchanaburi (Thong Pha Phum NP, Pha Tad Watershed Management station), Phetchaburi (Kaeng Krachan NP). ***Northeastern***: Kalasin (Phu Sithan WS), Chaiyaphum (Phu Khiao WS), Loei (Phu Luang WS), Nakhon Ratchasima (Sakaerat, Khao Yai NP, Buer Yai), Ubon Ratchathani (Pha Taem NP). ***Central***: Uthai Thani (Huai Kha Khaeng WS), Saraburi (Phukae BG), Bangkok (Bang Khen, Chatuchak) ***Eastern***: Sa Kaeo (Khao Ang Reu Nai WS), Chachoengsao (Khao Ang Reu Nai WS), Chon Buri (Si Racha, Khao Kheow), Chanthaburi (Khao Soi Dao WS, Pheao NP, Khao Ang Reu Nai WS), Rayong (Ko Man Nai, Khao Ang Reu Nai WS). ***Southern***: Chumphon (Krom Luang Chumphon NP), Ranong (Suk Samran, Khlong Na Kha WS), Surat Thani (Khlong Yan WS, Mu Ko Ang Thong NP), Nakhon Si Thammarat (Khao Nan NP, Khao Luang NP, Krung Ching Waterfall), Krabi (Ko Lanta), Trang (Khao Chong BG, Thung Khai BG, Palian), Phatthalung (Khao Pu–Khao Ya NP, Khao Bantad WS), Satun (Tarutao NP), Songkhla (Khao Kho Hong, Khao Nam Khang NP), Pattani (Yaring), Narathiwat (Hala–Bala WS).

**References.**Jaitrong and Nabhitabhata (2005, cited as *Pachycondyla luteipes* (Mayr, 1862)), Sakchoowong et al. (2008), Onishi et al. (2016).

***Brachyponera nigrita* (Emery, 1895)**

*Ponera nigrita* Emery, 1895: 459.

**Distribution. *Northern***: Chiang Mai (Khun Chang Khian, Pa Miang Village, Doi Inthanon NP, Doi Ang Khang, Doi Suthep–Pui NP, Omkoi). ***Western***: Tak (Umphang WS, Thung Yai Naresuan East WS). ***Northeastern***: Nakhon Ratchasima (Sakaerat, Khao Yai NP, Buer Yai). ***Central***: Uthai Thani (Huai Kha Khaeng WS). ***Southern***: Trang (Khao Chong BG, Thung Khai BG), Narathiwat (Hala–Bala WS).

**References.**Jaitrong and Nabhitabhata (2005, cited as *Pachycondyla nigrita* (Emery, 1895)), Yamane (2007), Onishi et al. (2016).

***Buniapone amblyops* (Emery, 1887)**

*Ponera amblyops* Emery, 1887d: 434.

**Distribution. *Northern***: Chiang Mai (Mae Chaem, Omkoi). ***Western***: Tak (Thung Yai Naresuan East WS). ***Eastern***: Chachoengsao (Khao Ang Reu Nai WS). ***Southern***: Narathiwat (Hala–Bala WS).

**References.**Jaitrong and Nabhitabhata (2005, cited as *Pachycondyla amblyops* (Emery, 1887)).

***Centromyrmex feae* (Emery, 1889)**

*Spalacomyrmex feae* Emery, 1889a: 491, pl. 10, figs 11–15.

**Distribution. *Northern***: Chiang Mai (Mae Chaem, Mae Taeng, Chiang Dao WS, Omkoi, Pha Hom Pok NP), Phayao (Mae Ka, Mae Yluard), Lampang (Tham Pha Thai NP). ***Western***: Tak (Thung Yai Naresuan East WS, Umphang WS). ***Northeastern***: Chaiyaphum (Phu Khiao WS), Mukdahan (Phu Sithan WS), Nakhon Ratchasima (Khao Yai NP, Sakaerat). ***Central***: Uthai Thani (Huai Kha Khaeng WS), Saraburi (Phu Kae BG). ***Eastern***: Chachoengsao (Khao Ang Reu Nai WS), Chanthaburi (Khao Soi Dao WS, Pheao NP), Trat (Ko Chang, Ko Kut). ***Southern***: Phang–nga (Khura Buri), Nakhon Si Thamarat (Khao Luang NP, Tapi Watershed Research Station), Phuket (Thalang), Krabi (Ko Lanta), Trang (Khao Chong BG, Thung Khai BG, Palian), Narathiwat (Hala–Bala WS).

**References.**Jaitrong and Nabhitabhata (2005), Sakchoowong et al. (2008), Jaitrong (2011), Jaitrong and Jeenthong (2014a).

***Cryptopone testacea* Emery, 1893**

*Cryptopone testacea* Emery, 1893a: cclxxv.

**Distribution. *Southern***: Yala.

**References.**Bingham (1906).

***Diacamma intricatum* (Smith, 1857)**

*Ponera intricata* Smith, 1857: 67.

**Distribution. *Eastern***: Chachoengsao (Khao Ang Reu Nai WS), Chanthaburi (Pheao NP).

**References.**Jaitrong and Nabhitabhata (2005).

***Diacamma jaitrongi* Zettel, Pal & Laciny, 2016**

*Diacamma jaitrongi* Zettel, Pal & Laciny, 2016: 141, figs 14–17.

**Distribution. *Northern***: Chiang Mai (Pha Hom Pok NP*).

**References.**Zettel et al. (2016).

***Diacamma longitudinale* Emery, 1889**

Figs 80, 81

*Diacamma longitudinale* Emery, 1889a: 496.

**Distribution. *Western***: Kanchanaburi (Thong Pha Phum). ***Central***: Saraburi (Phu Kae BG). ***Eastern***: Sa Kaeo (Pang Sida NP), Chachoengsao (Khao Ang Reu Nai WS).

**Remarks.** New record.

**Material examined.** W Thailand, Kanchanaburi Prov, Thong Pha Phum Dist, Natural Forest, 8.III.2005, Watana leg., PF1–14 (THNHM); C Thailand, Saraburi Prov, Phu Kae BG, 19.IX.2016, W. Jaitrong leg., WJT190916–1 (THNHM); Thailand, Chachoengsao Prov, Khao Ang Reu Nai WS, Lumchangwat Station, 25.IV.2003, W. Jaitrong leg. (THNHM); E Thailand, Sa Kaeo Prov, Huai Nam Yen, Pang Sida NP, 27.V.2006, WJT06–E367 (THNHM).

***Diacamma orbiculatum* Santschi, 1932**

*Diacamma ceylonensis* [sic!] var. orbiculatum Santschi, 1932: 14.

**Distribution. *Northern***: Chiang Mai (Omkoi), Lampang (Ngao). ***Northeastern***: Kalasin (Phu Sithan WS), Mukdahan (Phu Sithan WS), Ubon Ratchathani (Pha Taem NP), Loei (Phu Kradueng NP), Chaiyaphum (Phu Khiao WS, Nam Tok Tad Ton, Pa Hin Ngam NP), Sakon Nakhon (Phu Phan NP), Nakhon Ratchasima (Sakaerat, Khao Yai NP). ***Central***: Phetchabun (Khao Khor NP, Nam Nao NP), Uthai Thani (Huai Kha Khaeng WS), Saraburi (Namtok Sam Lan NP), Nakhon Nayok (Khao Yai NP), Pathum Thani (Khlong Luang), Bangkok (Bang Khen), Samut Songkhram (Mueang Samut Songkhram). ***Eastern***: Sa Kaeo (Khao Ang Reu Nai WS), Chachoengsao (Khao Ang Reu Nai WS), Chanthaburi (Khao Soi Dao WS, Pheao NP), Chon Buri (Si Racha), Rayong (Khao Chamao–Khao Wong NP, Khao Ang Reu Nai WS), Trat (Ko Chang, Ko Kut, Agroforestry Research Station). ***Southern***: Trang (Khao Chong BG), Narathiwat (Hala–Bala WS).

**References.**Jaitrong and Nabhitabhata (2005), Zettel et al. (2016), Suwannaphak et al. (2016).

**Remarks.** All *Diacamma* specimens were identified as *Diacamma rugosum* (Le Guillou, 1842) in Jaitrong and Nabhitabhata (2005) being reidentified as *D. orbiculatum* in the present paper.

***Diacamma violaceum* Forel, 1900**

Diacamma scalpratum var. violaceum Forel, 1900c: 317. Status as species: Laciny, Pal & Zettel, 2015: 93.

**Distribution. *Northern***: Chiang Mai (Mae Rim, Mae Sa Waterfall, Doi Suthep–Pui NP, Doi Inthanon NP, Omkoi), Mae Hong Son (Tham Lot Forest Park, Mae Sariang).

**References.**Laciny et al. (2015), Zettel et al. (2016).

**Remarks.** All *Diacamma* specimens were identified as *Diacamma sculpturata* (Smith, 1859) in Jaitrong and Nabhitabhata (2005) should be reidentified as *D. violaceum* in the present paper.

***Ectomomyrmex annamitus* (André, 1892)**

*Ponera annamita* André, 1892: 48.

**Distribution.** Thailand (unknown locality).

**References.**André (1892), Emery (1911b), Chapman and Capco (1951), Tiwari (1999).

***Ectomomyrmex astutus* (Smith, 1858)**

*Pachycondyla astuta* Smith, 1858: 107.

**Distribution. *Northern***: Chiang Mai (Doi Suthep–Pui NP, Doi Ang Khang, Omkoi), Nan (Doi Phu Kha NP). ***Western***: Tak (Thung Yai Naresuan East WS, Umphang WS), Kanchanaburi (Thong Pha Phum NP). ***Northeastern***: Nakhon Ratchasima (Forestry Camp, Sakaerat, Khao Yai NP). ***Central***: Pathum Thani (Khlong Luang). ***Eastern***: Chachoengsao (Khao Ang Reu Nai WS), Chanthaburi (Khao Khitchakut NP, Khao Soi Dao WS, Khao Chamao–Khao Wong NP, Pheao NP), Trat (Ko Kut). ***Southern***: Chumphon (Krom Luang Chumphon WS), Surat Thani (Khlong Yan WS), Nakhon Si Thammarat (Khao Luang NP, Khao Nan NP), Krabi (Ko Lanta), Trang (Khao Chong BG), Songkhla (Ton Nga Chang WS), Yala.

**References.**Bingham (1906), Jaitrong and Nabhitabhata (2005), Sakchoowong et al. (2009, cited as *Pachycondyla astuta* Smith, 1858), Jaitrong and Jeenthong (2014a), Suwannaphak et al. (2016).

***Ectomomyrmex leeuwenhoeki* (Forel, 1886)**

*Ponera leeuwenhoeki* Forel, 1886: 244.

**Distribution. *Northern***: Chiang Mai (Pa Miang Village, Chiang Dao WS, Mae Chaem, Mae Taeng). ***Western***: Tak (Thung Yai Naresuan East WS, Umphang WS), Kanchanaburi (Thong Pha Phum NP). ***Northeastern***: Kalsin (Phu Sithan WS), Mukdahan (Phu Sithan WS), Chaiyaphum (Phu Khiao WS), Nakhon Ratchasima (Sakaerat, Khao Yai NP). ***Central***: Uthai Thani (Huai Kha Khaeng WS). ***Eastern***: Chachoengsao (Khao Ang Reu Nai WS), Chanthaburi (Khlung, Khao Soi Dao WS, Pheao NP), Trat (Ko Kut). ***Southern***: Chumphon (Krom Luang Chumphon WS), Surat Thani (Khlong Saeng WS, Khlong Yan WS), Krabi (Ko Lanta), Trang (Thung Khai BG, Khao Chong BG), Phatthalung (Khao Pu–Khao Ya NP), Songkhla (Kuan Khao Wang Forest Park).

**References.**Jaitrong and Nabhitabhata (2005, cited as *Pachycondyla leeuwenhoeki* (Forel, 1886)), Sakchoowong et al. (2009, cited as *Pachycondyla leeuwenhoeki* (Forel, 1886)), Onishi et al. (2016).

***Emeryopone buttelreepeni* Forel, 1912**

*Emeryopone buttelreepeni* Forel, 1912b: 762.

**Distribution. *Northern***: Chiang Mai (Chiang Dao WS). ***Western***: Tak (Umphang WS, Thung Yai Naresuan East WS), Kanchanaburi (Thong Pha Phum). ***Northeastern***: Chaiyaphum (Phu Khiao WS), Nakhon Ratchasima (Sakaerat, Khao Yai NP). ***Central***: Uthai Thani (Ban Rai). ***Eastern***: Chachoengsao (Khao Ang Reu Nai WS), Chanthaburi (Khao Soi Dao WS, Pheao NP). ***Southern***: Chumphon (Krom Luang Chumphon WS), Ranong (Khlong Na Kha WS), Surat Thani (Khlong Yan WS), Nakhon Si Thamarat (Khao Luang NP), Trang (Khao Chong BG), Narathiwat (Hala–Bala WS).

**References.**Jaitrong and Nabhitabhata (2005), Jaitrong (2011).

***Harpegnathos venator* (Smith, 1858)**

*Drepanognathus venator* Smith, 1858: 82.

**Distribution. *Northern***: Chiang Mai (Chiang Mai University, Chiang Dao WS, Omkoi), Nan (Nakhon Nan Forest Plantation). ***Northeastern***: Mukdahan (Phu Sithan WS), Nakhon Ratchasima (Sakaerat). ***Central***: Phitsanulok (Thung Salaeng Luang NP), Saraburi (Phu Kae BG). ***Eastern***: Chanthaburi (Khlung).

**References.**Jaitrong and Nabhitabhata (2005), Jaitrong (2011).

***Leptogenys aspera* (André, 1889)**

*Lobopelta aspera* André, 1889: 222.

**Distribution. *Eastern***: Chachoengsao (Khao Ang Reu Nai).

**References.** Arimoto and Yamane (2018)

***Leptogenys birmana* Forel, 1900**

Leptogenys (Lobopelta) birmana Forel, 1900c: 310.

**Distribution. *Northern***: Chiang Rai (Doi Tung), Chiang Mai (Doi Ang Khang, Doi Luang Chiang Dao, Chiang Dao WS, Mae Taeng, Doi Suthep–Pui NP, Chiang Mai University Campus, Doi Inthanon NP, Mae Chaem, Omkoi, Doi Pha Hom Pok NP), Phayao (Mae Ka), Lamphun (Mae Li Forest Plantation), Lampang (Haui Tak, Tham Pha Thai NP), Phrae (Wang Chin Forest Plantation), Nan (Doi Phu Kha NP, Nakhon Nan Forest Plantation). ***Western***: Tak (Umphang WS, Thung Yai Naresuan East WS, Lansang NP, Taksin Maharat NP), Kanchanaburi (Thong Pha Phum NP, Pha Tad Watershed Management station), Phetchaburi (Kaeng Krachan NP), Prachuap Khiri Khan (Huai Yang NP). ***Northeastern***: Udon Thani (Nong Saeng), Surin (Huai Thab Than WS), Kalasin (Phu Sithan WS), Chaiyaphum (Phu Khiao WS), Loei (Phu Luang WS), Ubon Ratchathani (Khong Chiam), Nakhon Ratchasima (Sakaerat, Khao Yai NP, Buer Yai), Ubon Ratchathani (Pha Taem NP). ***Central***: Phetchabun (Khao Khor NP), Phitsanulok (Thung Salaeng Luang NP), Uthai Thai (Huai Kha Khaeng WS), Saraburi (Phukae BG), Bangkok (Bang Khen, Chatuchak) ***Eastern***: Sa Kaeo (Khao Ang Reu Nai WS), Chachoengsao (Khao Ang Reu Nai WS), Chon Buri (Si Racha, Khao Kheow, Khao Ang Reu Nai WS), Chanthaburi (Khao Soi Dao WS, Pheao NP, Khao Ang Reu Nai WS), Rayong (Ko Man Nai, Khao Ang Reu Nai WS), Trat (Khlong Yai, Ko Chang). ***Southern***: Chumphon (Krom Luang Chumphon NP), Ranong (Khlong Na Kha WS), Surat Thani (Khlong Yan WS, Mu Ko Ang Thong NP, Khlong Saeng WS), Nakhon Si Thammarat (Khao Nan NP, Khao Luang NP, Krung Ching Waterfall), Krabi (Ko Lanta), Trang (Khao Chong BG, Thung Khai BG, Palian, Ton Tae Waterfall), Phatthalung (Khao Pu–Khao Ya NP, Khao Bantad WS), Satun (Tarutao NP), Songkhla (Khao Kho Hong, Khao Nam Khang NP, Ton Nga Chang WS), Narathiwat (Hala–Bala WS).

**References.**Jaitrong and Nabhitabhata (2005), Jaitrong and Jeenthong (2014a).

***Leptogenys borneensis* Wheeler, 1919**

Leptogenys (Lobopelta) borneensis Wheeler, 1919: 59.

**Distribution. *Western***: Kanchanaburi (Thong Pha Phum NP). ***Central***: Uthai Thani (Huai Kha Khaeng WS). ***Eastern***: Chachoengsao (Khao Ang Reu Nai WS). ***Southern***: Chumphon (Krom Luang Chumphon WS), Nakhon Si Thammarat (Khao Luang NP, Khao Nan NP), Trang (Khao Chong BG, Thung Khai BG), Narathiwat (Hala–Bala WS).

**References.**Jaitrong and Nabhitabhata (2005), Sakchoowong et al. (2009).

***Leptogenys cyanicatena* Arimoto & Yamane, 2018**

*Lobopelta chalybaea* Emery, 1887c: 432.

**Distribution. *Northern***: Chiang Mai (Doi Suthep). ***Western***: Tak (Thung Yai Naresuan East WS), Kanchanaburi (Khuean Srinagarindra NP), Phetchaburi (Kaeng Krachan NP). ***Northeastern***: Nakhon Ratchasima (Khao Yai NP). ***Central***: Nakhon Nayok (Sarika Watwerfall). ***Eastern***: Chachoengsao (Khao Ang Reu Nai WS*), Chon Buri (Bang Phra), Chanthaburi (Khao Khitchakut NP).

**References.**Jaitrong and Nabhitabhata (2005, cited as *Leptogenys chalybaea* (Emery, 1887)), Arimoto and Yamane (2018).

**Remarks.** All *Leptogenys* specimens were identified as *L. chalybaea* in Jaitrong and Nabhitabhata (2005) being reidentified as *L. cyanicatena* in the present paper.

***Leptogenys diminuta* (Smith, 1857)**

*Ponera diminuta* Smith, 1857: 69.

**Distribution. *Northern***: Chiang Rai (Doi Tung), Chiang Mai (Doi Ang Khang, Doi Luang Chiang Dao, Chiang Dao WS, Mae Taeng, Doi Suthep–Pui NP, Chiang Mai University Campus, Doi Inthanon NP, Mae Chaem, Omkoi, Doi Pha Hom Pok NP), Phayao (Mae Ka), Lamphun (Mae Li Forest Plantation), Lampang (Haui Tak, Tham Pha Thai NP), Phrae (Wang Chin Forest Plantation), Nan (Doi Phu Kha NP, Nakhon Nan Forest Plantation). ***Western***: Tak (Umphang WS, Thung Yai Naresuan East WS, Lansang NP, Taksin Maharat NP), Kanchanaburi (Thong Pha Phum NP, Pha Tad Watershed Management station), Phetchaburi (Kaeng Krachan NP), Prachuap Khiri Khan (Huai Yang NP). ***Northeastern***: Udon Thani (Nong Saeng), Surin (Huai Thab Than WS), Kalasin (Phu Sithan WS), Chaiyaphum (Phu Khiao WS), Loei (Phu Luang WS), Nakhon Ratchasima (Sakaerat, Khao Yai NP, Buer Yai), Ubon Ratchathani (Khong Chiam, Pha Taem NP). ***Central***: Phetchabun (Khao Khor NP), Phitsanulok (Thung Salaeng Luang NP), Uthai Thai (Huai Kha Khaeng WS), Saraburi (Phukae BG), Bangkok (Bang Khen, Chatuchak), Pathum Thani (Khlong Luang). ***Eastern***: Sa Kaeo (Khao Ang Reu Nai WS), Chachoengsao (Khao Ang Reu Nai WS), Chon Buri (Si Racha, Khao Kheow, Khao Ang Reu Nai WS), Chanthaburi (Khao Soi Dao WS, Pheao NP, Khao Ang Reu Nai WS), Rayong (Ko Man Nai, Khao Ang Reu Nai WS), Trat (Trat Agroforestry Research Station, Ko Chang, Ko Kut). ***Southern***: Chumphon (Krom Luang Chumphon NP), Ranong (Khlong Na Kha WS), Surat Thani (Tai Rom Yen NP, Khlong Yan WS, Mu Ko Ang Thong NP, Khlong Saeng WS), Nakhon Si Thammarat (Khao Nan NP, Khao Luang NP, Krung Ching Waterfall), Krabi (Ko Lanta), Trang (Khao Chong BG, Thung Khai BG, Palian, Ton Tae Waterfall), Phatthalung (Khao Pu–Khao Ya NP, Khao Bantad WS, Khao Pappha), Satun (Tarutao NP), Songkhla (Khao Kho Hong, Khao Nam Khang NP, Ton Nga Chang WS), Narathiwat (Hala–Bala WS).

**References.**Jaitrong and Nabhitabhata (2005), Sakchoowong et al. (2009), Suwannaphak et al. (2016).

***Leptogenys hysterica* Forel, 1900**

Leptogenys (Lobopelta) hysterica Forel, 1900c: 311.

**Distribution. *Northern***: Chiang Mai (Omkoi). ***Western***: Tak (Umphang WS, Thung Yai Naresuan East WS). ***Northeastern***: Nakhon Ratchasima (Sakaerat, Khao Yai NP). ***Central***: Uthai Thani (Huai Kha Khaeng WS). ***Eastern***: Chachoengsao (Khao Ang Reu Nai WS), Chanthaburi (Khao Soi Dao WS). ***Southern***: Ranong (Khlong Na Kha WS), Trang (Khao Chong BG), Songkhla (Ton Nga Chang WS).

**References.**Jaitrong and Nabhitabhata (2005).

***Leptogenys iridescens* (Smith, 1857)**

*Ponera iridescens* Smith, 1857: 66.

**Distribution. *Western***: Kanchanaburi (Thong Pha Phum NP). ***Eastern***: Chachoengsao (Khao Ang Reu Nai WS).

**References.**Jaitrong and Nabhitabhata (2005), Sakchoowong et al. (2009).

***Leptogenys kitteli* (Mayr, 1870)**

*Lobopelta kitteli* Mayr, 1870: 966.

**Distribution. *Northern***: Chiang Rai (Doi Tung), Chiang Mai (Pa Miang Village, Doi Ang Khang, Doi Luang Chiang Dao, Chiang Dao WS, Mae Taeng, Doi Suthep–Pui NP, Chiang Mai University Campus, Doi Inthanon NP, Mae Chaem, Omkoi, Doi Pha Hom Pok NP), Phayao (Mae Ka), Lamphun (Mae Li Forest Plantation), Lampang (Haui Tak, Tham Pha Thai NP), Phrae (Wang Chin Forest Plantation), Nan (Doi Phu Kha NP, Nakhon Nan Forest Plantation). ***Western***: Tak (Umphang WS, Thung Yai Naresuan East WS, Lansang NP, Taksin Maharat NP), Kanchanaburi (Thong Pha Phum NP, Pha Tad Watershed Management station), Phetchaburi (Kaeng Krachan NP), Prachuap Khiri Khan (Huai Yang NP). ***Northeastern***: Udon Thani (Nong Saeng), Surin (Huai Thab Than WS), Kalasin (Phu Sithan WS), Chaiyaphum (Phu Khiao WS), Loei (Phu Luang WS), Ubon Ratchathani (Khong Chiam, Ubon Ratchathani Zoo, Pha Taem NP), Nakhon Ratchasima (Sakaerat, Khao Yai NP, Buer Yai). ***Central***: Phetchabun (Khao Khor NP), Phitsanulok (Thung Salaeng Luang NP), Uthai Thai (Huai Kha Khaeng WS), Saraburi (Phukae BG), Bangkok (Bang Khen, Chatuchak) ***Eastern***: Sa Kaeo (Khao Ang Reu Nai WS), Chachoengsao (Khao Ang Reu Nai WS), Chon Buri (Si Racha, Khao Kheow, Khao Ang Reu Nai WS), Chanthaburi (Khao Soi Dao WS, Pheao NP, Khao Ang Reu Nai WS), Rayong (Ko Man Nai, Khao Ang Reu Nai WS), Trat (Khlong Yai, Ko Chang), Trat (Ko Kut). ***Southern***: Chumphon (Krom Luang Chumphon NP), Ranong (Khlong Na Kha WS), Surat Thani (Tai Rom Yen NP, Khlong Yan WS, Mu Ko Ang Thong NP, Khlong Saeng WS), Nakhon Si Thammarat (Khao Nan NP, Khao Luang NP, Krung Ching Waterfall), Krabi (Ko Lanta), Trang (Khao Chong BG, Thung Khai BG, Palian, Ton Tae Waterfall), Phatthalung (Khao Pu–Khao Ya NP, Khao Bantad WS), Satun (Tarutao NP), Songkhla (Khao Kho Hong, Khao Nam Khang NP, Ton Nga Chang WS), Narathiwat (Hala–Bala WS).

**References.**Jaitrong and Nabhitabhata (2005), Onishi et al. (2016), Arimoto and Yamane (2018).

***Leptogenys kitteli altisquamis* Forel, 1900**

Leptogenys (Lobopelta) kitteli r. altisquamis Forel, 1900c: 306.

**Distribution.** Thailand (unknown locality).

**References.**Wheeler (1927a).

***Leptogenys kraepelini* Forel, 1905**

Leptogenys (Lobopelta) kraepelini Forel, 1905: 5.

**Distribution. *Northeastern***: Nakhon Ratchasima (Sakaerat, Khao Yai NP). ***Eastern***: Chachoengsao (Khao Ang Reu Nai WS), Trat (Ko Kut). ***Southern***: Trang (Khao Chong BG), Narathiwat (Hala–Bala WS).

**References.**Jaitrong and Nabhitabhata (2005).

***Leptogenys lucidula* Emery, 1895**

*Leptogenys lucidula* Emery, 1895: 462.

**Distribution. *Northeastern***: Nakhon Ratchasima (Khao Yai NP).

**References.**Antweb (2019).

***Leptogenys mutabilis* (Smith, 1861)**

*Ponera mutabilis* Smith, 1861: 45.

**Distribution. *Eastern***: Chachoengsao (Khao Ang Reu Nai WS). ***Southern***: Ranong (Khlong Na Kha WS), Phatthalung (Khao Phap Pha), Narathiwat (Hala–Bala WS).

**References.**Jaitrong and Nabhitabhata (2005).

***Leptogenys myops* (Emery, 1887)**

*Lobopelta myops* Emery, 1887c: 432.

**Distribution. *Northeastern***: Nakhon Ratchasima (Sakaerat, Khao Yai NP). ***Eastern***: Chachoengsao (Khao Ang Reu Nai WS), Trat (Ko Kut). ***Southern***: Ranong (Khlong Na Kha WS), Nakhon Si Thammarat (Khao Nan NP, Khao Luang NP), Krabi (Ko Lanta), Narathiwat (Hala–Bala WS).

**References.**Jaitrong and Nabhitabhata (2005).

***Leptogenys punctiventris* (Mayr, 1879)**

*Lobopelta punctiventris* Mayr, 1879: 666.

**Distribution. *Central***: Bangkok.

**References.**Antweb (2019).

***Mesoponera rubra* (Smith, 1857)**

*Ponera rubra* Smith, 1857: 66.

**Distribution. *Eastern***: Chachoengsao (Khao Ang Reu Nai WS). ***Southern***: Ranong (Khlong Yan WS), Nakhon Si Thammarat (Khao Nan NP).

**References.**Jaitrong and Nabhitabhata (2005, cited as *Pachycondyala rubra* (Smith 1857)).

***Myopias bidens* (Emery, 1900)**

*Trapeziopelta bidens* Emery, 1900a: 313.

**Distribution. *Western***: Tak (Umphang Dist, Um Phang WS, Thung Yai Naresuan East WS). ***Eastern***: Chanthaburi (Khlung Dist, near Trok Nong Waterfall). ***Southern***: Narathiwat (Su Ngai Ko Lok, Ban Toh Daeng).

**References.**Jaitrong et al. (2018a).

***Myopias crawleyi* (Donisthorpe, 1941)**

*Trapeziopelta nitida* Crawley, 1924: 384, fig. 2.

**Distribution. *Western***: Tak (Umphang, Thung Yai Naresuan East WS), Kanchanaburi (Thong Pha Phum), Phetchaburi (Kaeng Krachan NP), Prachuap Khiri Khan (Huai Yang NP). ***Southern***: Nakhon Si Thammarat (Tha Sala Dist).

**References.**Jaitrong et al. (2018a).

***Myopias maligna punctigera* (Emery, 1900)**

Trapeziopelta maligna var. punctigera Emery, 1900a: 663.

**Distribution. *Southern***: Narathiwat (Wang Dist).

**References.**Jaitrong et al. (2018a).

***Myopias mandibularis* (Crawley, 1924)**

*Trapeziopelta mandibularis* Crawley, 1924: 386, fig. 3.

**Distribution. *Southern***: Nakhon Si Thammarat (Tha Sala Dist), Surat Thani (Vibhavadi), Ranong (Khlong Naka).

**References.**Jaitrong et al. (2018a).

***Myopias minima* Jaitrong, Tasen & Guénard, 2018**

*Myopias minima* Jaitrong, Tasen & Guénard, 2018: 162–165, figs 28–30.

**Distribution. *Southern***: Phatthalung (Sribanpot, Riang Thong Waterfall*).

**References.**Jaitrong et al. (2018a).

***Myopias sakaeratensis* Jaitrong, Tasen & Guénard, 2018**

*Myopias sakaeratensis* Jaitrong, Tasen & Guénard, 2018: 166–168, figs 34–36.

**Distribution. *Northeastern***: Nakhon Ratchasima (Wang Nam Khiao, Sakaerat Environmental Research Station*), Chaiyaphum (Phu Khiao WS).

**References.**Jaitrong et al. (2018a).

***Myopias sonthichaiae* Jaitrong, Tasen & Guénard, 2018**

*Myopias sonthichaiae* Jaitrong, Tasen & Guénard, 2018: 168–172, figs 37–39.

**Distribution. *Northern***: Chiang Mai (Doi Ang Khang*). ***Western***: Tak (Thung Yai Naresuan East WS).

**References.**Jaitrong et al. (2018a).

***Odontomachus latidens* Mayr, 1867**

*Odontomachus latidens* Mayr, 1867: 80.

**Distribution. *Southern***: Narathiwat (Hala–Bala WS).

**References.**Jaitrong and Nabhitabhata (2005).

***Odontomachus monticola* Emery, 1892**

*Odontomachus monticola* Emery, 1892: 560.

**Distribution. *Northern***: Chiang Mai (Doi Ang Khang, Ob Luang NP). ***Western***: Tak (Thung Yai Naresuan East WS, Umphang WS). ***Central***: Uthai Thani (Huai Kha Khaeng WS).

**References.**Jaitrong and Nabhitabhata (2005).

***Odontomachus rixosus* Smith, 1857**

*Odontomachus rixosus* Smith, 1857: 64.

**Distribution. *Western***: Tak (Umphang WS, Thung Yai Naresuan East WS, Lansang NP, Taksin Maharat NP), Kanchanaburi (Thong Pha Phum NP, Pha Tad Watershed Management station), Phetchaburi (Kaeng Krachan NP), Prachuap Khiri Khan (Huai Yang NP). ***Northeastern***: Udon Thani (Nong Saeng), Surin (Huai Thab Than WS), Kalasin (Phu Sithan WS), Chaiyaphum (Phu Khiao WS), Loei (Phu Luang WS), Ubon Ratchathani (Khong Chiam, Pha Taem NP), Nakhon Ratchasima (Sakaerat, Khao Yai NP, Buer Yai). ***Central***: Phetchabun (Khao Khor NP), Phitsanulok (Thung Salaeng Luang NP), Uthai Thai (Huai Kha Khaeng WS), Saraburi (Phukae BG), Bangkok (Bang Khen, Chatuchak) ***Eastern***: Sa Kaeo (Khao Ang Reu Nai WS), Chachoengsao (Khao Ang Reu Nai WS), Chon Buri (Si Racha, Khao Kheow, Khao Ang Reu Nai WS), Chanthaburi (Khao Soi Dao WS, Pheao NP, Khao Ang Reu Nai WS), Rayong (Ko Man Nai, Khao Ang Reu Nai WS), Trat (Trat Agroforestry Research Station, Ko Chang, Ko Kut). ***Southern***: Chumphon (Krom Luang Chumphon WS), Ranong (Khlong Na Kha WS), Surat Thani (Tai Rom yen NP, Khlong Yan WS, Mu Ko Ang Thong NP, Khlong Saeng WS), Nakhon Si Thammarat (Khao Nan NP, Khao Luang NP, Krung Ching Waterfall), Krabi (Ko Lanta), Trang (Khao Chong BG, Thung Khai BG, Palian, Ton Tae Waterfall), Phatthalung (Khao Pu–Khao Ya NP, Khao Bantad WS), Satun (Tarutao NP), Songkhla (Khao Kho Hong, Khao Nam Khang NP, Ton Nga Chang WS), Yala, Pattani (Namtok Sai Khao NP, Nong Chik), Narathiwat (Hala–Bala WS).

**References.**Bingham (1906), Jaitrong and Nabhitabhata (2005), Sakchoowong et al. (2009), Jaitrong and Jeenthong (2014a), Satria et al. (2015).

***Odontomachus simillimus* Smith, 1858**

*Odontomachus simillimus* Smith, 1858: 80, pl. 5, figs 8, 9.

**Distribution. *Western***: Tak (Umphang WS, Thung Yai Naresuan East WS, Lansang NP, Taksin Maharat NP), Kanchanaburi (Thong Pha Phum NP, Pha Tad Watershed Management station), Phetchaburi (Kaeng Krachan NP), Prachuap Khiri Khan (Huai Yang NP). ***Northeastern***: Udon Thani (Nong Saeng), Surin (Huai Thab Than WS), Kalasin (Phu Sithan WS), Chaiyaphum (Phu Khiao WS), Loei (Phu Luang WS), Ubon Ratchathani (Khong Chiam), Nakhon Ratchasima (Sakaerat, Khao Yai NP, Buer Yai), Ubon Ratchathani (Pha Taem NP). ***Central***: Phetchabun (Khao Khor NP), Phitsanulok (Thung Salaeng Luang NP), Uthai Thai (Huai Kha Khaeng WS), Saraburi (Phukae BG), Bangkok (Bang Khen, Chatuchak), Pathum Thani (Khlong Luang), Samut Prakan (Bang Krachao), Samut Songkhram (Mueang Samut Songkhram). ***Eastern***: Sa Kaeo (Khao Ang Reu Nai WS), Chachoengsao (Khao Ang Reu Nai WS), Chon Buri (Si Racha, Khao Kheow, Khao Ang Reu Nai WS), Chanthaburi (Khao Soi Dao WS, Pheao NP, Khao Ang Reu Nai WS), Rayong (Ko Man Nai, Khao Ang Reu Nai WS), Trat (Khlong Yai, Ko Chang). ***Southern***: Chumphon (Krom Luang Chumphon WS), Ranong (Khlong Na Kha WS), Surat Thani (Khlong Yan WS, Mu Ko Ang Thong NP, Khlong Saeng WS), Nakhon Si Thammarat (Khao Nan NP, Khao Luang NP, Krung Ching Waterfall), Krabi (Ko Lanta), Trang (Khao Chong BG, Thung Khai BG, Palian, Ton Tae Waterfall), Phatthalung (Khao Pu–Khao Ya NP, Khao Bantad WS), Satun (Tarutao NP), Songkhla (Khao Kho Hong, Khao Nam Khang NP, Ton Nga Chang WS), Yala, Pattani (Namtok Sai Khao NP), Narathiwat (Hala–Bala WS).

**References.**Bingham (1906), Jaitrong and Nabhitabhata (2005), Jaitrong and Jeenthong (2014a), Satria et al. (2015).

***Odontoponera denticulata* (Smith, 1858)**

*Ponera denticulata* Smith, 1858: 90, pl. 6, figs 13, 14.

**Distribution. *Northern***: Chiang Rai (Doi Tung), Chiang Mai (Pa Miang Village, Doi Ang Khang, Doi Luang Chiang Dao, Chiang Dao WS, Mae Taeng, Doi Suthep–Pui NP, Chiang Mai University Campus, Doi Inthanon NP, Mae Chaem, Omkoi, Doi Pha Hom Pok NP), Phayao (Mae Ka), Lamphun (Mae Li Forest Plantation), Lampang (Haui Tak, Tham Pha Thai NP), Phrae (Wang Chin Forest Plantation), Nan (Doi Phu Kha NP, Nakhon Nan Forest Plantation). ***Western***: Tak (Umphang WS, Thung Yai Naresuan East WS, Lansang NP, Taksin Maharat NP), Kanchanaburi (Thong Pha Phum NP, Pha Tad Watershed Management station), Phetchaburi (Kaeng Krachan NP), Prachuap Khiri Khan (Huai Yang NP). ***Northeastern***: Udon Thani (Nong Saeng), Surin (Huai Thab Than WS), Kalasin (Phu Sithan WS), Chaiyaphum (Phu Khiao WS), Loei (Phu Luang WS), Ubon Ratchathani (Khong Chiam, Pha Taem NP), Nakhon Ratchasima (Sakaerat, Khao Yai NP, Buer Yai). ***Central***: Phetchabun (Khao Khor NP), Phitsanulok (Thung Salaeng Luang NP), Uthai Thai (Huai Kha Khaeng WS), Saraburi (Phukae BG), Bangkok (Bang Khen, Chatuchak), Pathum Thani (Khlong Luang), Samut Prakan (Bang Krachao), Samut Songkhram (Mueang Samut Songkhram). ***Eastern***: Sa Kaeo (Khao Ang Reu Nai WS), Chachoengsao (Khao Ang Reu Nai WS), Chon Buri (Si Racha, Khao Kheow, Ko Samaesarn, Khao Ang Reu Nai WS), Chanthaburi (Khao Soi Dao WS, Pheao NP, Khao Ang Reu Nai WS), Rayong (Ko Man Nai, Khao Ang Reu Nai WS), Trat (Trat Agroforestry Research Station, Ko Chang, Ko Kut). ***Southern***: Chumphon (Krom Luang Chumphon WS), Ranong (Khlong Na Kha WS), Surat Thani (Tai Rom Yen NP, Khlong Yan WS, Mu Ko Ang Thong NP, Khlong Saeng WS), Nakhon Si Thammarat (Khao Nan NP, Khao Luang NP, Krung Ching Waterfall), Krabi (Ko Lanta), Trang (Khao Chong BG, Thung Khai BG, Palian, Ton Tae Waterfall), Phatthalung (Khao Pu–Khao Ya NP, Khao Bantad WS), Satun (Tarutao NP), Songkhla (Khao Kho Hong, Khao Nam Khang NP, Ton Nga Chang WS), Yala, Pattani (Nong Chik), Narathiwat (Hala–Bala WS).

**References.**Jaitrong and Nabhitabhata (2005), Yamane (2009), Sakchoowong et al. (2009), Jaitrong (2011), Jaitrong and Jeenthong (2014a), Onishi et al. (2016).

***Odontoponera transversa* (Smith, 1857)**

*Ponera transversa* Smith, 1857: 68.

**Distribution. *Southern***: Nakhon Si Thammarat (Khao Nan NP, Khao Luang NP), Trang (Khao Chong BG), Narathiwat (Hala–Bala WS).

**References.**Jaitrong and Nabhitabhata (2005), Yamane (2009), Jaitrong (2011).

***Parvaponera darwinii* (Forel, 1893)**

*Belonopelta darwinii* Forel, 1893c: 460.

**Distribution.** Thailand (unknown locality).

**References.**Collingwood (1962).

***Platythyrea clypeata* Forel, 1911**

*Plathyrea clypeata* Forel, 1911b: 378.

**Distribution. *Eastern***: Chachoengsao (Khao Ang Reu Nai WS, Tha Takiab), Sa Kaeo (Khao Ang Reu Nai WS), Chanthaburi (Soi Dao)

**References.** Phengsi et al. (2018)

***Platythyrea janyai* Phengsi, Jaitrong, Ruangsittichai & Khachonpisitsak, 2018**

*Platythyrea janyai* Phengsi, Jaitrong, Ruangsittichai & Khachonpisitsak, 2018: 89–92, figs 1, 5.

**Distribution. *Southern***: Phatthalung (Riang Thong Waterfall, Khao Pu–Khao Ya NP, Si Banphot*), Trang (Khao Chong BG, Na Yong)

**References.** Phengsi et al. (2018)

***Platythyrea parallela* (Smith, 1859)**

*Ponera parallela* Smith, 1859: 143.

**Distribution. *Northern***: Chiang Mai (Omkoi, Chiang Mai University), Lamphun (Mae Li Forest Plantation). ***Western***: Tak (Umphang WS), Kanchanaburi (Thong Pha Phum NP). ***Northeastern***: Mukdahan (Phu Sithan WS), Loei (Phu Luang WS), Nakhon Ratchasima (Sakaerat). ***Central***: Phitsanulok (Phu Soi Dao NP), Pathum Thani (Khlong Luang). ***Eastern***: Chachoengsao (Khao Ang Reu Nai WS). ***Southern***: Surat Thani (Khlong Saeng WS), Nakhon Si Thammarat (Tai Rom Yen NP).

**References.**Jaitrong and Nabhitabhata (2005).

***Platythyrea quadridenta* Donisthorpe, 1941**

*Platythyrea quadridenta* Donisthorpe, 1941: 134.

**Distribution. *Southern***: Narathiwat (Hala–Bala WS).

**References.**Jaitrong and Nabhitabhata (2005).

***Platythyrea tricuspidata* Emery, 1900**

*Platythyrea tricuspidata* Emery, 1900a: 665.

**Distribution. *Southern***: Nakhon Si Thammarat (Khao Nan NP), Narathiwat (Hala–Bala WS).

**References.**Jaitrong and Nabhitabhata (2005).

***Pseudoneoponera rufipes* (Jerdon, 1851)**

*Ponera rufipes* Jerdon, 1851: 119.

**Distribution. *Northern***: Chiang Mai (Chiang Dao WS), Lampang (Ngao, Mae Chang Forest Plantation), Phrae (Wang Chin). ***Western***: Tak (Thung Yai Naresuan East WS, Umphang WS). ***Northeastern***: Mukdahan (Phu Sithan WS). ***Central***: Pathum Thani (Khlong Luang). ***Eastern***: Trat (Ko Chang). ***Southern***: Ranong (Khlong Na Kha WS), Krabi (Ko Lanta), Trang (Khao Chong BG), Narathiwat (Hala–Bala WS).

**References.**Jaitrong and Nabhitabhata (2005, cited as *Pachycondyla rufipes* (Jerdon 1851)).

#### Subfamily Proceratiinae [3 genera, 5 species]

***Probolomyrmex dammermani* Wheeler, 1928**

*Probolomyrmex dammermani* Wheeler, 1928a: 7, fig. 1.

**Distribution. *Western***: Tak (Thung Yai Naresuan East WS). ***Southern***: Trang (Khao Chong BG).

**References.**Jaitrong and Nabhitabhata (2005).

***Probolomyrmex longinodus* Terayama & Ogata, 1988**

*Probolomyrmex longinodus* Terayama & Ogata, 1988: 592, figs 6–8.

**Distribution. *Northern***: Chiang Mai (Doi Suthep–Pui NP).

**References.**Eguchi et al. (2006).

***Probolomyrmex vieti* Eguchi, Yoshimura et Yamane, 2006**

*Probolomyrmex vieti* Eguchi, Yoshimura & Yamane, 2006: 29, figs 7A–F, 9G, 14A–F, 15G, H, 16J–L.

**Distribution. *Northeastern***: Nakhon Ratchasima (Khao Yai NP).

**References.**Eguchi et al. (2006).

***Proceratium deelemani* Perrault, 1981**

*Procertium deelemani* Perrault, 1981: 189, figs 1–6.

**Distribution. *Western***: Tak (Thung Yai Naresuan East WS, Umphang WS), Phetchaburi (Kaeng Krachan NP). ***Northeastern***: Nakhon Ratchasima (Khao Yai NP). ***Central***: Uthai Thani (Huai Kha Khaeng WS).

**References.** Baroni Urbani & de Andrade (2003), Jaitrong and Nabhitabhata (2005), Jaitrong (2011).

***Proceratium siamense* de Andrade, 2003**

*Proceratium siamense* de Andrade, in Baroni Urbani & de Andrade, 2003: 342, fig. 136.

**Distribution. *Northern***: Chiang Mai (Doi Inthanon NP*).

**References.** Baroni Urbani & de Andrade (2003).

#### Subfamily Pseudomyrmecinae [1 genus, 16 species]

***Tetraponera aitkenii* (Forel, 1902)**

*Sima aitkenii* Forel, 1902b: 245.

**Distribution. *Northeastern***: Chaiyaphum (Pa Hin Ngam NP, Lan Hin Nau), Loei (Phu Kradueng NP, Bamboo forest at Lam Huai Taad)

**References.** Antweb (2018).

***Tetraponera allaborans* (Walker, 1859)**

*Pseudomyrma allaborans* Walker, 1859: 375.

**Distribution. *Northern***: Chiang Mai (Doi Suthep–Pui NP, Mae Rim), Mae Hong Son (Khun Yuam), Nan (Doi Phu Kha NP). ***Western***: Tak (Thung Yai Naresuan East WS, Umphang WS), Kanchanaburi (Thong Pha Phum NP). ***Northeastern***: Mukdahan (Phu Sithan WS), Chaiyaphum (Phu Khiao WS), Nakhon Ratchasima (Khao Yai NP). ***Central***: Bangkok (Bang Khen). ***Eastern***: Sa Kaeo (Khao Ang Reu Nai WS), Chachoengsao (Khao Ang Reu Nai WS), Chanthaburi (Khlung, Khao Soi Dao WS), Rayong (Ko Man), Trat (Ko Kut). ***Southern***: Nakhon Si Thammarat (Tai Rom Yen NP), Phang–nga (Khao Lak), Satun (Thale Ban NP), Trang (Khao Chong BG).

**References.**Ward (2001), Jaitrong and Nabhitabhata (2005), Sakchoowong et al. (2009).

***Tetraponera attenuata* Smith, 1877**

*Tetraponera attenuata* Smith, 1877: 71.

**Distribution. *Northern***: Chiang Rai (Doi Tung), Chiang Mai (Pa Miang Village, Doi Ang Khang, Doi Luang Chiang Dao, Chiang Dao WS, Mae Taeng, Doi Suthep–Pui NP, Chiang Mai University Campus, Doi Inthanon NP, Mae Chaem, Omkoi, Doi Pha Hom Pok NP), Phayao (Mae Ka), Lamphun (Mae Li Forest Plantation), Lampang (Haui Tak, Tham Pha Thai NP), Phrae (Wang Chin Forest Plantation), Nan (Doi Phu Kha NP, Nakhon Nan Forest Plantation). ***Western***: Tak (Umphang WS, Thung Yai Naresuan East WS, Lansang NP, Taksin Maharat NP), Kanchanaburi (Thong Pha Phum NP, Pha Tad Watershed Management station), Phetchaburi (Kaeng Krachan NP), Prachuap Khiri Khan (Huai Yang NP). ***Northeastern***: Udon Thani (Nong Saeng), Surin (Huai Thab Than WS), Kalasin (Phu Sithan WS), Chaiyaphum (Phu Khiao WS), Loei (Phu Luang WS), Ubon Ratchathani (Khong Chiam, Pha Taem NP), Nakhon Ratchasima (Sakaerat, Khao Yai NP, Buer Yai). ***Central***: Phetchabun (Khao Khor NP), Phitsanulok (Thung Salaeng Luang NP), Uthai Thai (Huai Kha Khaeng WS), Saraburi (Phukae BG), Bangkok (Bang Khen, Chatuchak), Pathum Thani (Khlong Luang), Samut Songkhram (Mueang Samut Songkhram). ***Eastern***: Sa Kaeo (Khao Ang Reu Nai WS), Chachoengsao (Khao Ang Reu Nai WS), Chon Buri (Si Racha, Khao Kheow, Khao Ang Reu Nai WS), Chanthaburi (Khao Soi Dao WS, Pheao NP, Khao Ang Reu Nai WS), Rayong (Ko Man, Ko Man Nai, Khao Ang Reu Nai WS), Trat (Khlong Yai, Ko Chang). ***Southern***: Chumphon (Krom Luang Chumphon NP), Ranong (Khlong Na Kha WS), Surat Thani (Khlong Yan WS, Mu Ko Ang Thong NP, Khlong Saeng WS), Nakhon Si Thammarat (Khao Nan NP, Khao Luang NP, Krung Ching Waterfall, Tapi Watershed Research Station), Krabi (Ko Lanta), Trang (Khao Chong BG, Thung Khai BG, Palian, Ton Tae Waterfall), Phatthalung (Khao Pu–Khao Ya NP, Khao Bantad WS), Satun (Tarutao NP), Songkhla (Khao Kho Hong, Khao Nam Khang NP, Ton Nga Chang WS), Narathiwat (Hala–Bala WS).

**References.**Ward (2001), Tongjerm et al. (2003), Jaitrong and Nabhitabhata (2005), Suwannaphak et al. (2016), Onishi et al. (2016).

***Tetraponera binghami* (Forel, 1902)**

*Sima binghami* Forel, 1902b: 243.

**Distribution. *Northern***: Chiang Mai (Doi Suthep–Pui NP, Mae Ya Waterfall, Omkoi), Mae Hong Son (Khun Yuam), Lamphun (Mae Li Forest Plantation). ***Western***: Tak (Thung Yai Naresuan East WS, Umphang WS).

**References.**Ward (2001), Jaitrong and Nabhitabhata (2005), Jaitrong and Jeenthong (2014a).

***Tetraponera concava* Xu & Chai, 2004**

*Tetraponera concava* Xu & Chai, 2004: 65, figs 6–10.

**Distribution. *Northeastern***: Chaiyaphum (Phu Khiao WS).

**References.**Antweb (2019).

***Tetraponera connectens* Ward, 2001**

*Tetraponera connectens* Ward, 2001: 611, figs 13, 24, 49.

**Distribution. *Southern***: Phang–nga (Khao Lak NP*).

**References.**Ward (2001).

***Tetraponera crassiuscula* (Emery, 1900)**

Sima allaborans subsp. crassiuscula Emery, 1900b: 677, fig. 6.

**Distribution. *Southern***: Surat Thani (Khao Sok NP).

**References.**Ward (2001), Jaitrong and Nabhitabhata (2005).

***Tetraponera difficilis* (Emery, 1900)**

*Sima difficilis* Emery, 1900a: 677.

**Distribution. *Western***: Kanchanaburi (Mae Klong Watershed Reseacch Station). ***Eastern***: Chachoengsao (Khao Ang Reu Nai WS). ***Southern***: Phang–nga (Khao Lak NP, Takua Pa), Nakhon Si Thammarat (Khao Luang NP, Phrom Lok Waterfall), Satun (Thale Ban NP), Songkhla (Ton Nga Chang WS), Yala (Betong).

**References.**Ward (2001), Jaitrong and Nabhitabhata (2005).

***Tetraponera extenuata* Ward, 2001**

*Tetraponera extenuata* Ward, 2001: 614, figs 16, 27, 33, 39, 50.

**Distribution. *Western***: Tak (Thung Yai Naresuan East WS). ***Northeastern***: Nakhon Ratchasima (Sakaerat). ***Eastern***: Chachoengsao (Khao Ang Reu Nai WS). ***Southern***: Nakhon Si Thammarat (Khao Nan NP), Trang (Khao Chong BG).

**References.**Ward (2001), Jaitrong and Nabhitabhata (2005).

***Tetraponera modesta* (Smith, 1860)**

*Pseudomyrma modesta* Smith, 1860a: 106.

**Distribution. *Western***: Tak (Thung Yai Naresuan East WS). ***Eastern***: Chachoengsao (Khao Ang Reu Nai WS). ***Southern***: Satun (Thale Ban NP).

**References.**Ward (2001), Jaitrong and Nabhitabhata (2005).

***Tetraponera nigra* (Jerdon, 1851)**

*Eciton nigrum* Jerdon, 1851: 112.

**Distribution. *Northern***: Chiang Mai (Ob Luang NP, Doi Suthep–Pui NP), Lampang (Huai Tak). ***Southern***: Nakhon Si Thammarat (Khao Nan NP), Satun (Thale Ban NP), Yala.

**References.**Bingham (1906), Ward (2001), Jaitrong and Nabhitabhata (2005).

***Tetraponera nitida* (Smith, 1860)**

*Pseudomyrma nitida* Smith, 1860a: 106.

**Distribution. *Western***: Kanchanaburi (Tham Than Lod). ***Northeastern***: Mukdahan (Phu Sithan WS), Si Sa Ket (Kanthararom). ***Central***: Bangkok (Bang Khen). ***Eastern***: Chon Buri (Si Racha), Chachoengsao (Khao Ang Reu Nai WS), Chanthaburi (Pong Nam Ron). ***Southern***: Phang–nga (Khao Lak NP), Nakhon Si Thammarat (Khao Nan NP, Khao Luang NP), Trang (Palian, Songkhla (Hat Yai).

**References.**Ward (2001), Jaitrong and Nabhitabhata (2005).

***Tetraponera nodosa* Ward, 2001**

*Tetraponera nodosa* Ward, 2001: 639, figs 75, 83.

**Distribution. *Southern***: Songkhla (Hat Yai).

**References.**Ward (2001), Jaitrong and Nabhitabhata (2005).

***Tetraponera notabilis* Ward, 2001**

*Tetraponera notabilis* Ward, 2001: 640, figs 76, 84.

**Distribution. *Northeastern***: Nakhon Ratchasima (Sakaerat*).

**References.**Ward (2001), Jaitrong and Nabhitabhata (2005).

***Tetraponera pilosa* (Smith, 1858)**

*Pseudomyrma pilosa* Smith, 1858: 160.

**Distribution. *Northern***: Chiang Rai (Doi Tung). ***Western***: Tak (Umphang WS), Kanchanaburi (Thong Pha Phum NP). ***Central***: Uthai Thani (Huai Kha Khaeng WS) ***Eastern***: Chanthaburi (Khao Soi Dao WS). ***Southern***: Phang–nga (Takua Pa), Krabi (Ko Lanta), Trang (Khao Chong BG), Songkhla (Ton Nga Chang WS).

**References.**Ward (2001), Jaitrong and Nabhitabhata (2005), Jaitrong and Jeenthong (2014a).

***Tetraponera rufonigra* (Jerdon, 1851)**

*Eciton rufonigrum* Jerdon, 1851: 111.

**Distribution. *Northern***: Chiang Rai (Doi Tung), Chiang Mai (Doi Ang Khang, Doi Luang Chiang Dao, Chiang Dao WS, Mae Taeng, Doi Suthep–Pui NP, Chiang Mai University Campus, Doi Inthanon NP, Mae Chaem, Omkoi, Doi Pha Hom Pok NP), Phayao (Mae Ka), Lamphun (Mae Li Forest Plantation), Lampang (Haui Tak, Tham Pha Thai NP), Phrae (Wang Chin Forest Plantation), Nan (Doi Phu Kha NP, Nakhon Nan Forest Plantation). ***Western***: Tak (Umphang WS, Thung Yai Naresuan East WS, Lansang NP, Taksin Maharat NP), Kanchanaburi (Thong Pha Phum NP, Pha Tad Watershed Management station), Phetchaburi (Kaeng Krachan NP), Prachuap Khiri Khan (Namtok Huai Yang NP). ***Northeastern***: Udon Thani (Nong Saeng), Surin (Huai Thab Than WS), Kalasin (Phu Sithan WS), Chaiyaphum (Phu Khiao WS), Loei (Phu Luang WS), Ubon Ratchathani (Khong Chiam, Pha Taem NP), Nakhon Ratchasima (Sakaerat, Khao Yai NP, Buer Yai). ***Central***: Phetchabun (Khao Khor NP), Phitsanulok (Thung Salaeng Luang NP), Uthai Thai (Huai Kha Khaeng WS), Saraburi (Phukae BG), Bangkok (Bang Khen, Chatuchak), Pathum Thani (Khlong Luang), Samut Songkhram (Mueang Samut Songkhram). ***Eastern***: Sa Kaeo (Khao Ang Reu Nai WS), Chachoengsao (Khao Ang Reu Nai WS), Chon Buri (Si Racha, Khao Kheow, Khao Ang Reu Nai WS), Chanthaburi (Khao Soi Dao WS, Pheao NP, Khao Ang Reu Nai WS), Rayong (Ko Man Nai, Khao Ang Reu Nai WS), Trat (Khlong Yai, Ko Chang). ***Southern***: Chumphon (Krom Luang Chumphon WS), Ranong (Khlong Na Kha WS), Surat Thani (Khlong Yan WS, Mu Ko Ang Thong NP, Khlong Saeng WS), Nakhon Si Thammarat (Khao Nan NP, Khao Luang NP, Krung Ching Waterfall), Krabi (Ko Lanta), Trang (Khao Chong BG, Thung Khai BG, Palian, Ton Tae Waterfall), Phatthalung (Khao Pu–Khao Ya NP, Khao Bantad WS), Satun (Tarutao NP), Songkhla (Khao Kho Hong, Khao Nam Khang NP, Ton Nga Chang WS), Yala, Pattani (Nong Chik), Narathiwat (Hala–Bala WS).

**References.**Bingham (1906), Ward (2001), Jaitrong and Nabhitabhata (2005), Jaitrong and Jeenthong (2014a), Suwannaphak et al. (2016).

**Figure 82.**
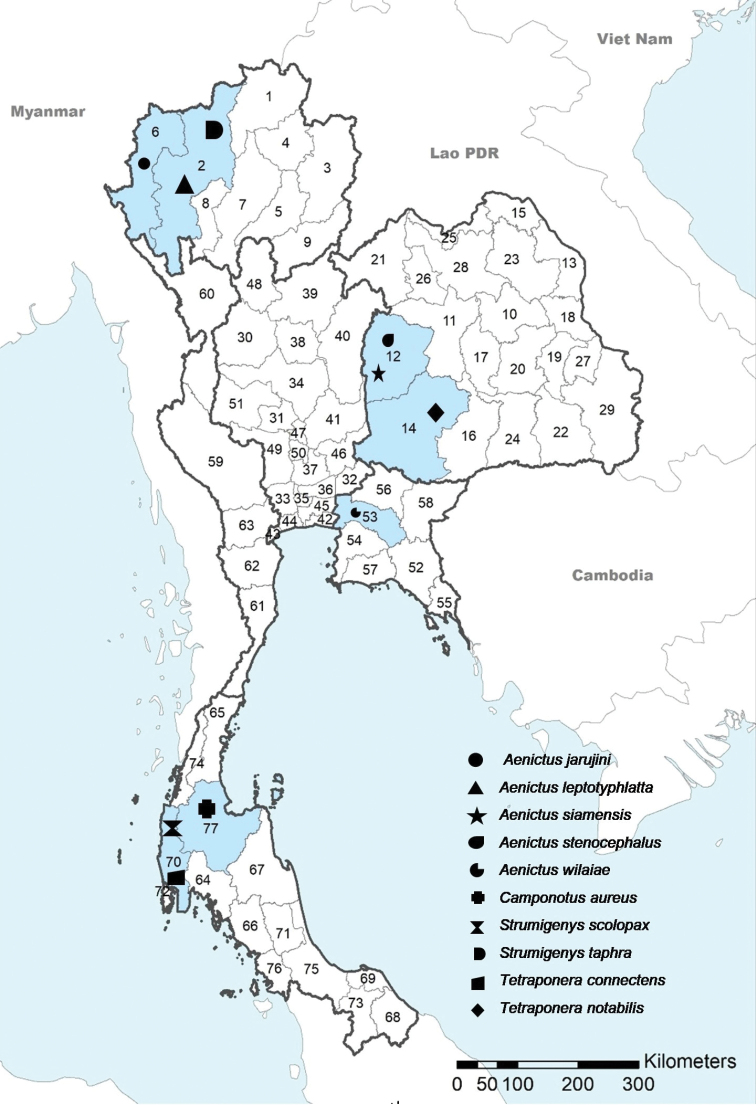
Distribution of ten endemic species in Thailand which are located in the blue areas.

## Supplementary materials

Supplementary material 1 Table S1–S4 Data type species data Explanation note **Table S1.** Numbers and percentages of species within each genus, and its ratio (%) to the total species numbers of ants so far recorded in Thailand. Unidentified species are excluded. * remarks the genus with unidentified species by morphological species identification. **Table S2.** Numbers of ant species by subfamily found among six geographical regions. If records do not mention specific regions, then they are put in ‘Unknown’. N is stranded for north, NE: northeast, C: central, E: east, W: west, S: south. **Table S3.** Numbers of provinces found ant species by genus among six geographical regions. If records do not mention specific regions, then they are put in ‘Unknown’. Nominal species define numbers of species by genus. N is stranded for north, NE: northeast, C: central, E: east, W: west, S: south. **Table S4**. List of ant species described from Thailand and their type localities. * denotes endemic species. https://binary.pensoft.net/file/479604This dataset is made available under the Open Database License (http://opendatacommons.org/licenses/odbl/1.0/). The Open Database License (ODbL) is a license agreement intended to allow users to freely share, modify, and use this Dataset while maintaining this same freedom for others, provided that the original source and author(s) are credited.Salinee Khachonpisitsak, Seiki Yamane, Patchara Sriwichai, Weeyawat Jaitrong
