# Supplementary material for: An updated checklist of the ants of Thailand (Hymenoptera, Formicidae)
Source: Zookeys. 2020 Nov 26;998:1–182. doi: 10.3897/zookeys.998.54902 (PMC7714773; doi:10.3897/zookeys.998.54902)
Supplement: Supplementary material 1 — Table S1–S4 [file zookeys-998-001-s001.docx]

**Supplement Table 1.** Numbers and percentages of species within each genus, and its ratio (%) to the total species numbers of ants so far recorded in Thailand. Unidentified species are excluded. * remarks the genus with unidentified species by morphological species identification.

|  | **Genus** | **Species** | **%** |
| --- | --- | --- | --- |
| 1 | *Polyrhachis* | 62 | 11.72 |
| 2 | *Pheidole* | 51 | 9.64 |
| 3 | *Aenictus* | 39 | 7.37 |
| 4 | *Strumigenys* | 32 | 6.05 |
| 5 | *Crematogaster* | 29 | 5.48 |
| 6 | *Camponotus* | 26 | 4.91 |
| 7 | *Tetramorium* | 26 | 4.91 |
| 8 | *Technomyrmex* | 16 | 3.02 |
| 9 | *Tetraponera* | 16 | 3.02 |
| 10 | *Leptogenys* | 14 | 2.65 |
| 11 | *Dolichoderus* | 13 | 2.46 |
| 12 | *Echinopla* | 11 | 2.08 |
| 13 | *Colobopsis* | 8 | 1.51 |
| 14 | *Gnamptogenys* | 7 | 1.32 |
| 15 | *Prenolepis* | 7 | 1.32 |
| 16 | *Carebara* | 7 | 1.32 |
| 17 | *Pristomyrmex* | 7 | 1.32 |
| 18 | *Myopias* | 7 | 1.32 |
| 19 | *Myrmoteras* | 6 | 1.13 |
| 20 | *Myrmecina* | 6 | 1.13 |
| 21 | *Cataulacus* | 5 | 0.95 |
| 22 | *Anochetus* | 5 | 0.95 |
| 23 | *Diacamma* | 5 | 0.95 |
| 24 | *Platythyrea* | 5 | 0.95 |
| 25 | *Stigmatomma* | 4 | 0.76 |
| 26 | *Acanthomyrmex* | 4 | 0.76 |
| 27 | *Cardiocondyla* | 4 | 0.76 |
| 28 | *Lophomyrmex* | 4 | 0.76 |
| 29 | *Meranoplus* | 4 | 0.76 |
| 30 | *Myrmicaria* | 4 | 0.76 |
| 31 | *Recurvidris* | 4 | 0.76 |
| 32 | *Odontomachus* | 4 | 0.76 |
| 33 | *Dorylus* | 3 | 0.57 |
| 34 | *Monomorium* | 3 | 0.57 |
| 35 | *Vollenhovia* | 3 | 0.57 |
| 36 | *Brachyponera* | 3 | 0.57 |
| 37 | *Ectomomyrmex* | 3 | 0.57 |
| 38 | *Probolomyrmex* | 3 | 0.57 |
| 39 | *Philidris* | 2 | 0.38 |
| 40 | *Tapinoma* | 2 | 0.38 |
| 41 | *Syscia* | 2 | 0.38 |
| 42 | *Acropyga* | 2 | 0.38 |
| 43 | *Cladomyrma* | 2 | 0.38 |
| 44 | *Euprenolepis* | 2 | 0.38 |
| 45 | *Calyptomyrmex* | 2 | 0.38 |
| 46 | *Rhopalomastix* | 2 | 0.38 |
| 47 | *Syllophopsis* | 2 | 0.38 |
| 48 | *Odontoponera* | 2 | 0.38 |
| 49 | *Proceratium* | 2 | 0.38 |
| 50 | *Myopopone* | 1 | 0.19 |
| 51 | *Mystrium* | 1 | 0.19 |
| 52 | *Prionopelta* | 1 | 0.19 |
| 53 | *Xymmer* | 1 | 0.19 |
| 54 | *Iridomyrmex* | 1 | 0.19 |
| 55 | *Ochetellus* | 1 | 0.19 |
| 56 | *Cerapachys* | 1 | 0.19 |
| 57 | *Chrysapace* | 1 | 0.19 |
| 58 | *Eusphinctus* | 1 | 0.19 |
| 59 | *Lioponera* | 1 | 0.19 |
| 60 | *Parasyscia* | 1 | 0.19 |
| 61 | *Simopone* | 1 | 0.19 |
| 62 | *Yunodorylus* | 1 | 0.19 |
| 63 | *Zasphinctus* | 1 | 0.19 |
| 64 | *Anoplolepis* | 1 | 0.19 |
| 65 | *Dinomyrmex* | 1 | 0.19 |
| 66 | *Lepisiota* | 1 | 0.19 |
| 67 | *Oecophylla* | 1 | 0.19 |
| 68 | *Paraparatrechina* | 1 | 0.19 |
| 69 | *Paratrechina* | 1 | 0.19 |
| 70 | *Pseudolasius* | 1 | 0.19 |
| 71 | *Leptanilla* | 1 | 0.19 |
| 72 | *Anillomyrma* | 1 | 0.19 |
| 73 | *Dacetinops* | 1 | 0.19 |
| 74 | *Dilobocondyla* | 1 | 0.19 |
| 75 | *Epelysidris* | 1 | 0.19 |
| 76 | *Erromyrma* | 1 | 0.19 |
| 77 | *Eurhopalothrix* | 1 | 0.19 |
| 78 | *Gauromyrmex* | 1 | 0.19 |
| 79 | *Kartidris* | 1 | 0.19 |
| 80 | *Lasiomyrma* | 1 | 0.19 |
| 81 | *Liomyrmex* | 1 | 0.19 |
| 82 | *Mayriella* | 1 | 0.19 |
| 83 | *Myrmica* | 1 | 0.19 |
| 84 | *Paratopula* | 1 | 0.19 |
| 85 | *Proatta* | 1 | 0.19 |
| 86 | *Solenopsis* | 1 | 0.19 |
| 87 | *Tetheamyrma* | 1 | 0.19 |
| 88 | *Trichomyrmex* | 1 | 0.19 |
| 89 | *Buniapone* | 1 | 0.19 |
| 90 | *Centromyrmex* | 1 | 0.19 |
| 91 | *Cryptopone* | 1 | 0.19 |
| 92 | *Emeryopone* | 1 | 0.19 |
| 93 | *Harpegnathos* | 1 | 0.19 |
| 94 | *Mesoponera* | 1 | 0.19 |
| 95 | *Parvaponera* | 1 | 0.19 |
| 96 | *Pseudoneoponera* | 1 | 0.19 |
| 97 | *Chronoxenus** | 0 | 0.00 |
| 98 | *Ooceraea** | 0 | 0.00 |
| 99 | *Gesomyrmex** | 0 | 0.00 |
| 100 | *Nylanderia** | 0 | 0.00 |
| 101 | *Plagiolepis** | 0 | 0.00 |
| 102 | *Protanilla** | 0 | 0.00 |
| 103 | *Aphaenogaster** | 0 | 0.00 |
| 104 | *Lordomyrma** | 0 | 0.00 |
| 105 | *Temnothorax** | 0 | 0.00 |
| 106 | *Vombisidris** | 0 | 0.00 |
| 107 | *Hypoponera** | 0 | 0.00 |
| 108 | *Ponera** | 0 | 0.00 |
| 109 | *Discothyrea** | 0 | 0.00 |
|  |  | **529** | **100.00** |

**Table 2.** Numbers of ant species by subfamily found among six geographical regions. If records do not mention specific regions, then they are put in ‘Unknown’. N is stranded for north, NE: northeast, C: central, E: east, W: west, S: south.

| **Subfamilies** | **N** | **NE** | **C** | **E** | **W** | **S** | **Unknown** |
| --- | --- | --- | --- | --- | --- | --- | --- |
| Amblyoponinae | 2 | 2 | 3 | 4 | 1 | 4 | 1 |
| Dolichoderinae | 22 | 19 | 15 | 16 | 21 | 20 | 0 |
| Dorylinae | 30 | 23 | 13 | 21 | 29 | 21 | 0 |
| Ectatomminae | 4 | 2 | 2 | 3 | 4 | 5 | 0 |
| Formicinae | 42 | 41 | 29 | 51 | 45 | 89 | 1 |
| Leptanillinae | 0 | 0 | 0 | 0 | 0 | 1 | 0 |
| Myrmicinae | 109 | 89 | 50 | 77 | 111 | 117 | 2 |
| Ponerinae | 24 | 25 | 26 | 35 | 28 | 36 | 3 |
| Proceratiinae | 2 | 2 | 1 | 0 | 2 | 1 | 0 |
| Pseudomyrmecinae | 6 | 8 | 5 | 8 | 9 | 12 | 0 |
| **Total** | **241** | **211** | **144** | **215** | **250** | **306** | **7** |

**Supplement Table 3.** **Numbers of provinces found ant species by genus** among six geographical regions. If records do not mention specific regions, then they are put in ‘Unknown’. Nominal species define numbers of species by genus. N is stranded for north, NE: northeast, C: central, E: east, W: west, S: south.

| **Subfamilies** | **Genera** | **N** | **NE** | **C** | **E** | **W** | **S** | **Unknown location** | **Nominal species** |
| --- | --- | --- | --- | --- | --- | --- | --- | --- | --- |
| Amblyoponinae | *Myopopone* | 0 | 0 | 2 | 3 | 1 | 3 | 0 | 1 |
|  | *Mystrium* | 4 | 2 | 2 | 3 | 0 | 0 | 0 | 1 |
|  | *Prionopelta* | 0 | 0 | 0 | 0 | 0 | 2 | 0 | 1 |
|  | *Stigmatomma* | 1 | 3 | 1 | 2 | 0 | 1 | 1 | 4 |
|  | *Xymmer* | 0 | 0 | 0 | 1 | 0 | 0 | 0 | 1 |
| Dolichoderinae | *Dolichoderus* | 6 | 4 | 5 | 6 | 5 | 12 | 0 | 12 |
|  | *Iridomyrmex* | 3 | 5 | 6 | 6 | 3 | 11 | 0 | 1 |
|  | *Ochetellus* | 1 | 0 | 2 | 0 | 0 | 0 | 0 | 1 |
|  | *Philidris* | 1 | 0 | 0 | 0 | 0 | 0 | 0 | 2 |
|  | *Tapinoma* | 4 | 5 | 6 | 6 | 3 | 12 | 0 | 2 |
|  | *Technomyrmex* | 6 | 8 | 5 | 7 | 4 | 12 | 0 | 16 |
| Dorylinae | *Aenictus* | 6 | 5 | 5 | 7 | 4 | 9 | 0 | 39 |
|  | *Cerapachys* | 1 | 2 | 1 | 2 | 1 | 2 | 0 | 1 |
|  | *Chrysapace* | 1 | 0 | 0 | 0 | 2 | 0 | 0 | 1 |
|  | *Dorylus* | 5 | 5 | 2 | 3 | 2 | 7 | 0 | 3 |
|  | *Eusphinctus* | 1 | 0 | 0 | 0 | 0 | 1 | 0 | 1 |
|  | *Lioponera* | 0 | 1 | 0 | 1 | 0 | 0 | 0 | 1 |
|  | *Parasyscia* | 0 | 1 | 0 | 3 | 1 | 0 | 0 | 1 |
|  | *Simopone* | 1 | 1 | 0 | 0 | 0 | 0 | 0 | 1 |
|  | *Syscia* | 0 | 0 | 1 | 0 | 1 | 1 | 0 | 2 |
|  | *Yunodorylus* | 1 | 0 | 0 | 0 | 1 | 0 | 0 | 1 |
|  | *Zasphinctus* | 1 | 0 | 0 | 0 | 0 | 0 | 0 | 1 |
| Ectatomminae | *Gnamptogenys* | 1 | 3 | 3 | 2 | 4 | 7 | 0 | 7 |
| Formicinae | *Acropyga* | 3 | 4 | 3 | 3 | 2 | 8 | 0 | 2 |
|  | *Anoplolepis* | 6 | 5 | 6 | 4 | 4 | 10 | 0 | 1 |
|  | *Camponotus* | 8 | 5 | 7 | 4 | 4 | 13 | 0 | 26 |
|  | *Cladomyrma* | 0 | 1 | 2 | 4 | 0 | 1 | 0 | 2 |
|  | *Colobopsis* | 3 | 4 | 2 | 3 | 1 | 9 | 0 | 8 |
|  | *Dinomyrmex* | 0 | 0 | 0 | 0 | 0 | 6 | 0 | 1 |
|  | *Echinopla* | 0 | 2 | 2 | 1 | 2 | 11 | 0 | 11 |
|  | *Euprenolepis* | 0 | 0 | 0 | 0 | 0 | 4 | 0 | 2 |
|  | *Lepisiota* | 0 | 0 | 1 | 0 | 0 | 0 | 0 | 1 |
|  | *Myrmoteras* | 2 | 2 | 1 | 3 | 2 | 4 | 0 | 6 |
|  | *Oecophylla* | 7 | 5 | 7 | 4 | 3 | 12 | 0 | 1 |
|  | *Paraparatrechina* | 6 | 5 | 2 | 4 | 3 | 9 | 0 | 1 |
|  | *Paratrechina* | 6 | 5 | 5 | 4 | 4 | 10 | 0 | 1 |
|  | *Polyrhachis* | 7 | 7 | 8 | 6 | 5 | 13 | 1 | 63 |
|  | *Prenolepis* | 1 | 1 | 0 | 0 | 1 | 5 | 0 | 7 |
|  | *Pseudolasius* | 1 | 0 | 0 | 0 | 0 | 0 | 0 | 1 |
| Leptanillinae | *Leptanilla* | 0 | 0 | 0 | 0 | 0 | 2 | 0 | 1 |
| Myrmicinae | *Acanthomyrmex* | 1 | 1 | 1 | 0 | 1 | 5 | 0 | 4 |
|  | *Anillomyrma* | 1 | 0 | 0 | 0 | 1 | 0 | 0 | 1 |
|  | *Calyptomyrmex* | 1 | 1 | 2 | 2 | 2 | 3 | 0 | 2 |
|  | *Cardiocondyla* | 2 | 1 | 2 | 1 | 2 | 4 | 0 | 4 |
|  | *Carebara* | 7 | 5 | 7 | 4 | 4 | 11 | 0 | 7 |
|  | *Cataulacus* | 7 | 5 | 5 | 5 | 4 | 12 | 0 | 5 |
|  | *Crematogaster* | 7 | 6 | 7 | 5 | 4 | 12 | 1 | 29 |
|  | *Dacetinops* | 0 | 0 | 0 | 0 | 0 | 1 | 0 | 1 |
|  | *Dilobocondyla* | 2 | 0 | 0 | 1 | 1 | 0 | 0 | 1 |
|  | *Epelysidris* | 0 | 0 | 0 | 0 | 0 | 1 | 0 | 1 |
|  | *Erromyrma* | 1 | 0 | 0 | 1 | 0 | 1 | 0 | 1 |
|  | *Eurhopalothrix* | 0 | 0 | 0 | 0 | 0 | 2 | 0 | 1 |
|  | *Gauromyrmex* | 1 | 0 | 0 | 0 | 1 | 2 | 0 | 1 |
|  | *Kartidris* | 1 | 1 | 1 | 0 | 0 | 0 | 0 | 1 |
|  | *Lasiomyrma* | 0 | 1 | 0 | 1 | 2 | 0 | 0 | 1 |
|  | *Liomyrmex* | 0 | 1 | 0 | 1 | 1 | 0 | 0 | 1 |
|  | *Lophomyrmex* | 1 | 3 | 1 | 2 | 3 | 10 | 0 | 4 |
|  | *Mayriella* | 0 | 1 | 0 | 1 | 0 | 0 | 0 | 1 |
|  | *Meranoplus* | 7 | 5 | 6 | 5 | 4 | 13 | 0 | 4 |
|  | *Monomorium* | 7 | 5 | 6 | 6 | 4 | 11 | 0 | 3 |
|  | *Myrmecina* | 1 | 1 | 0 | 1 | 1 | 2 | 0 | 6 |
|  | *Myrmica* | 1 | 0 | 0 | 0 | 1 | 0 | 0 | 1 |
|  | *Myrmicaria* | 2 | 2 | 1 | 2 | 1 | 8 | 0 | 4 |
|  | *Paratopula* | 0 | 1 | 1 | 3 | 0 | 0 | 0 | 1 |
|  | *Pheidole* | 7 | 7 | 7 | 6 | 4 | 12 | 0 | 51 |
|  | *Pristomyrmex* | 3 | 3 | 1 | 3 | 3 | 6 | 0 | 7 |
|  | *Proatta* | 0 | 2 | 3 | 3 | 1 | 4 | 0 | 1 |
|  | *Recurvidris* | 1 | 1 | 0 | 3 | 2 | 3 | 0 | 4 |
|  | *Rhopalomastix* | 0 | 0 | 4 | 1 | 1 | 1 | 0 | 2 |
|  | *Solenopsis* | 7 | 5 | 4 | 6 | 3 | 11 | 0 | 1 |
|  | *Strumigenys* | 2 | 3 | 0 | 1 | 4 | 9 | 0 | 32 |
|  | *Syllophopsis* | 0 | 1 | 0 | 1 | 1 | 0 | 0 | 2 |
|  | *Tetheamyrma* | 0 | 0 | 0 | 0 | 0 | 1 | 0 | 1 |
|  | *Tetramorium* | 3 | 2 | 4 | 2 | 2 | 7 | 0 | 26 |
|  | *Trichomyrmex* | 7 | 5 | 3 | 5 | 3 | 11 | 0 | 1 |
|  | *Vollenhovia* | 0 | 1 | 0 | 0 | 2 | 6 | 1 | 3 |
| Ponerinae | *Anochetus* | 4 | 5 | 6 | 5 | 3 | 9 | 0 | 5 |
|  | *Brachyponera* | 6 | 5 | 3 | 5 | 3 | 11 | 0 | 3 |
|  | *Buniapone* | 1 | 0 | 0 | 1 | 1 | 1 | 0 | 1 |
|  | *Centromyrmex* | 3 | 3 | 2 | 3 | 1 | 6 | 0 | 1 |
|  | *Cryptopone* | 0 | 0 | 0 | 0 | 0 | 1 | 0 | 1 |
|  | *Diacamma* | 3 | 7 | 7 | 6 | 1 | 2 | 0 | 5 |
|  | *Ectomomyrmex* | 2 | 4 | 2 | 3 | 2 | 8 | 1 | 3 |
|  | *Emeryopone* | 1 | 2 | 1 | 2 | 2 | 6 | 0 | 1 |
|  | *Harpegnathos* | 2 | 2 | 2 | 1 | 0 | 0 | 0 | 1 |
|  | *Leptogenys* | 7 | 7 | 7 | 6 | 4 | 10 | 1 | 14 |
|  | *Mesoponera* | 0 | 0 | 0 | 1 | 0 | 2 | 0 | 1 |
|  | *Myopias* | 1 | 2 | 0 | 1 | 4 | 5 | 0 | 7 |
|  | *Odontomachus* | 1 | 7 | 8 | 6 | 4 | 12 | 0 | 4 |
|  | *Odontoponera* | 7 | 7 | 8 | 6 | 4 | 12 | 0 | 2 |
|  | *Parvaponera* | 0 | 0 | 0 | 0 | 0 | 0 | 1 | 1 |
|  | *Platythyrea* | 2 | 3 | 2 | 3 | 2 | 5 | 0 | 5 |
|  | *Pseudoneoponera* | 3 | 1 | 1 | 1 | 1 | 4 | 0 | 1 |
| Proceratiinae | *Probolomyrmex* | 1 | 1 | 0 | 0 | 1 | 1 | 0 | 3 |
|  | *Proceratium* | 1 | 0 | 0 | 0 | 0 | 0 | 0 | 1 |
|  | *Procertium* | 0 | 1 | 1 | 0 | 2 | 0 | 0 | 1 |
| Pseudomyrmecinae | *Tetraponera* | 8 | 9 | 7 | 6 | 4 | 13 | 0 | 16 |

**Supplement Table 4.** List of ant species described from Thailand and their type localities. * denotes endemic species.

| **Genera** |  | **Species** | **Type locality** |
| --- | --- | --- | --- |
| ***Acanthomyrmex*** | 1 | *Acanthomyrmex thailandensis* Terayama, 1995 | Chiang Mai (Doi Suthep-Pui NP) |
|  | 2 | *Acanthomyrmex malikuli* Jaitrong & Asanok, 2019 | Tak (Thung Yai Naresuan WS) |
|  | 3 | *Acanthomyrmex mizunoi* Jaitrong & Asanok, 2019 | Nakhon Nayok (Ban Hin Tang) |
| ***Aenictus*** | 4 | *Aenictus changmaianus* Terayama & Kubota, 1993 | Chiang Mai (Doi Suthep-Pui NP) |
|  | 5 | *Aenictus concavus* Jaitrong & Yamane, 2013 | Nakhon Ratchasima (Khao Yai NP) |
|  | 6 | *Aenictus cylindripetiolus* Jaitrong & Yamane, 2013 | Trang (Khao Chong BG) |
|  | 7 | *Aenictus duengkaei* Jaitrong & Yamane, 2012 | Chachoengsao (Khao Ang Reu Nai WS) |
|  | 8 | *Aenictus fulvus* Jaitrong & Yamane, 2011 | Nakhon Si Thammarat (Khao Nan NP) |
|  | 9 | *Aenictus jarujini* Jaitrong & Yamane, 2010*** | Mae Hong Son (Haui Nam Dang NP) |
|  | 10 | *Aenictus leptotyphlatta* Jaitrong & Eguchi, 2010* | Chiang Mai (Chiang Mai University) |
|  | 11 | *Aenictus longinodus* Jaitrong & Yamane, 2012 | Trang (Palian, Khao Chong BG) |
|  | 12 | *Aenictus maneerati* Jaitrong & Yamane, 2013 | Tak (Thung Yai Naresuan WS) |
|  | 13 | *Aenictus nishimurai* Terayama & Kubota, 1993 | Chiang Mai (Doi Suthep-Pui NP) |
|  | 14 | *Aenictus nuchiti* Jaitrong & Ruangsittichai, 2018 | Chiang Mai (Omkoi National Forest) |
|  | 15 | *Aenictus paradentatus* Jaitrong & Yamane, 2012 | Chiang Mai (Doi Suthep-Pui NP) |
|  | 16 | *Aenictus parahuonicus* Jaitrong & Yamane, 2011 | Trang (Thung Khai BG) |
|  | 17 | *Aenictus pinkaewi* Jaitrong & Yamane, 2013 | Chiang Mai (Mueang Chiang Mai Dist) |
|  | 18 | *Aenictus samungi* Jaitrong & Ruangsittichai, 2018 | Tak (Thung Yai Naresuan WS) |
|  | 19 | *Aenictus siamensis* Jaitrong & Yamane, 2011* | Chaiyaphum (Phu Khiao WS) |
|  | 20 | *Aenictus stenocephalus* Jaitrong & Yamane, 2010* | Chaiyaphum (Phu Khiao WS) |
|  | 21 | *Aenictus thailandianus* Terayama & Kubota, 1993 | Chiang Mai (Doi Suthep-Pui NP) |
|  | 22 | *Aenictus watanasiti* Jaitrong & Yamane, 2013 | Chiang Mai (Doi Suthep-Pui NP) |
|  | 23 | *Aenictus wilaiae* Jaitrong & Yamane, 2013* | Chachoengsao (Khao Ang Reu Nai WS) |
|  | 24 | *Aenictus wiwatwitayai* Jaitrong & Yamane, 2013 | Nakhon Ratchasima (Sakaerat) |
| ***Camponotus*** | 25 | *Camponotus aureus* Dumpert, 2006* | Surat Thani (Khao Sok NP) |
|  | 26 | *Camponotus khaosokensis* Dumpert, 2006 | Surat Thani (Khao Sok NP) |
|  | 27 | *Camponotus paraleonardi* Zettel & Yamane, 2018 | Phang-nga (Khao Lak NP) |
|  | 28 | *Camponotus schoedli* Dumpert, 2006 | Surat Thani (Khao Sok NP) |
|  | 29 | *Camponotus schulzianus* Zettel & Balàka, 2018 | Phang-nga (Khao Lak NP) |
|  | 30 | *Camponotus sophiae* Zettel & Balàka, 2018 | Phang-nga (Khao Lak NP*). |
|  | 31 | *Camponotus weiserti* Zettel & Laciny, 2018 | Phang-nga (Khao Lak NP*). |
| ***Colobopsis*** | 32 | *Colobopsis markli* Dumpert, 2004 | Chanthaburi (Khao Chamao-Khao Wong NP) |
| ***Crematogaster*** | 33 | *Crematogaster artifex* Mayr, 1879 | Bangkok |
|  | 34 | *Crematogaster dohrni kerri* Forel, 1911 | Chiang Mai (unknown locality) |
|  | 35 | *Crematogaster fumikoae* Hosoishi & Ogata, 2015 | Chiang Mai (Doi Suthep-Pui NP) |
| ***Diacamma*** | 36 | *Diacamma jaitrongi* Zettel, Pal & Laciny, 2016 | Chiang Mai (Pha Hom Pok NP) |
| ***Dolichoderus*** | 37 | *Dolichoderus siggii* Forel, 1895 | Bangkok |
| ***Echinopla*** | 38 | *Echinopla charernsomi* Tanansathaporn & Jaitrong, 2018 | Nakhon Ratchasima (Sakaerat Environmental Research Station) |
|  | 39 | *Echinopla jeenthongi* Tanansathaporn & Jaitrong, 2018 | Nakhon Si Thammarat (Khao Nan NP) |
|  | 40 | *Echinopla madli* Zettel & Laciny, 2015 | Satun (Thale Ban NP) |
| ***Kartidris*** | 41 | *Kartidris matertera* Bolton, 1991 | Chiang Mai (Nong Hoi) |
| ***Lasiomyrma*** | 42 | *Lasiomyrma wiwatwitayai* Jaitrong, 2010 | Nakhon Ratchasima (Khao Yai NP) |
| ***Leptanilla*** | 43 | *Leptanilla thai* Baroni Urbani, 1977 | Trang (Khao Chong BG) |
| ***Lophomyrmex*** | 44 | *Lophomyrmex striatulus* Rigato, 1994 | Chanthaburi (Pheao NP) |
| ***Myopias*** | 45 | *Myopias minima* Jaitrong, Tasen & Guénard, 2018 | Phatthalung (Riang Thong Waterfall) |
|  | 46 | *Myopias sakaeratensis* Jaitrong, Tasen & Guénard, 2018 | Nakhon Ratchasima (Sakaerat Environmental Research Station) |
|  | 47 | *Myopias sonthichaiae* Jaitrong, Tasen & Guénard, 2018 | Chiang Mai (Doi Ang Khang) |
| ***Myrmoteras*** | 48 | *Myrmoteras jaitrongi* Bui, Eguchi & Yamane, 2013 | Narathiwat (Hala-Bala WS) |
| ***Polyrhachis*** | 49 | *Polyrhachis noonananti* Kohout, 2013 | Surat Thani (Khlong Saeng WS) |
|  | 50 | *Polyrhachis saevissima kerri* Forel, 1911 | Chiang Mai |
|  | 51 | *Polyrhachis sculpturata siamensis* Mayr, 1879 | Thailand |
|  | 52 | *Polyrhachis thailandica* Kohout, 2006 | Kanchanaburi (Mae Klong River) |
|  | 53 | *Polyrhachis watanasiti* Kohout, 2013 | Ranong (Ngao) |
| ***Prenolepis*** | 54 | *Prenolepis fustinoda* Williams & LaPolla, 2016 | Chiang Mai (Doi Inthanon NP) |
| ***Pristomyrmex*** | 55 | *Pristomyrmex leleji* Yamane & Dias, 2016 | Chanthaburi (Khao Khitchakut NP) |
|  | 56 | *Pristomyrmex rigidus* Wang, 2003 | Chanthaburi (Khao Sabab) |
| ***Proceratium*** | 57 | *Proceratium siamense* de Andrade, 2003 | Chiang Mai (Doi Inthanon NP) |
| ***Recurvidris*** | 58 | *Recurvidris chanapaithooni* Jaitrong & Wiwatwitaya, 2015 | Chanthaburi (Khao Soi Dao WS) |
|  | 59 | *Recurvidris lekakuli* Jaitrong, Tokeeree & Pitaktunsakul, 2019 | Western: Kanchanaburi (Thong Pha Phum) |
| ***Strumigenys*** | 60 | *Strumigenys adiastola* Bolton, 2000 | Kanchanaburi (Erawan Waterfall) |
|  | 61 | *Strumigenys arges* Bolton, 2000 | Chiang Mai (Doi Suthep-Pui NP) |
|  | 62 | *Strumigenys atropos* (Bolton, 2000) | Phetchaburi (Kaeng Krachan NP) |
|  | 63 | *Strumigenys benulia* Bolton, 2000 | Phetchaburi (Kaeng Krachan NP) |
|  | 64 | *Strumigenys brontes* Bolton, 2000 | Phetchaburi (Kaeng Krachan NP) |
|  | 65 | *Strumigenys caniophanes* Bolton, 2000 | Chiang Mai (Doi Suthep-Pui NP) |
|  | 66 | *Strumigenys dipsas* Bolton, 2000 | Chiang Mai (Doi Inthanon NP) |
|  | 67 | *Strumigenys nothomopyx* Bolton, 2000 | Chiang Mai (Doi Suthep-Pui NP [Monthathan Waterfall]) |
|  | 68 | *Strumigenys nytaxis* Bolton, 2000 | Phetchaburi (Kaeng Krachan NP) |
|  | 69 | *Strumigenys scolopax* Bolton, 2000* | Phang-nga (Si Phang-nga NP) |
|  | 70 | *Strumigenys strygax* Bolton, 2000 | Chiang Mai (Khun Chang Khian) |
|  | 71 | *Strumigenys taphra* Bolton, 2000*** | Chiang Mai (Doi Inthanon NP) |
|  | 72 | *Strumigenys tritomea* Bolton, 2000 | Chiang Mai (Web Pang An) |
| ***Technomyrmex*** | 73 | *Technomyrmex yamanei* Bolton, 2007 | Chiang Mai (Chiang Dao WS) |
| ***Tetramorium*** | 74 | *Tetramorium aptum* Bolton, 1977 | Chiang Mai (Nong Hoi) |
|  | 75 | *Tetramorium ciliatum* Bolton, 1977 | Chiang Mai (Nong Hoi) |
|  | 76 | *Tetramorium cuneinode* Bolton, 1977 | Chiang Mai (Nong Hoi) |
|  | 77 | *Tetramorium hasinae* Yamane & Jaitrong, 2011 | Nakhon Si Thammarat (Khao Nan NP) |
|  | 78 | *Tetramorium nacta* (Bolton, 1976) | Chiang Mai (Nong Hoi) |
| ***Tetraponera*** | 79 | *Tetraponera connectens* Ward, 2001* | Phang-nga (Khao Lak NP) |
|  | 80 | *Tetraponera notabilis* Ward, 2001* | Nakhon Ratchasima (Sakaerat) |
| ***Zasphinctus*** | 81 | *Zasphinctus siamensis* Jaitrong, 2016 | Chiang Mai (Mae Taeng) |
